# Morphology, Carbohydrate Composition and Vernalization Response in a Genetically Diverse Collection of Asian and European Turnips (*Brassica rapa* subsp. *rapa*)

**DOI:** 10.1371/journal.pone.0114241

**Published:** 2014-12-04

**Authors:** Ningwen Zhang, Jianjun Zhao, Frederic Lens, Joan de Visser, Temesgen Menamo, Wen Fang, Dong Xiao, Johan Bucher, Ram Kumar Basnet, Ke Lin, Feng Cheng, Xiaowu Wang, Guusje Bonnema

**Affiliations:** 1 Wageningen UR Plant Breeding, Wageningen, The Netherlands; 2 Institute of Vegetables and Flowers, Chinese Academy of Agricultural Sciences, Beijing, China; 3 Horticultural College, Hebei Agricultural University, Baoding, China; 4 State Key Laboratory of Crop Genetics and Germplasm Enhancement, Horticultural College, Nanjing Agricultural University, Nanjing, Jiangsu, China; 5 Naturalis Biodiversity Center, Leiden, The Netherlands; The University of Western Australia, Australia

## Abstract

*Brassica rapa* displays enormous morphological diversity, with leafy vegetables, turnips and oil crops. Turnips (*Brassica rapa* subsp. *rapa*) represent one of the morphotypes, which form tubers and can be used to study the genetics underlying storage organ formation. In the present study we investigated several characteristics of an extensive turnip collection comprising 56 accessions from both Asia (mainly Japanese origin) and Europe. Population structure was calculated using data from 280 evenly distributed SNP markers over 56 turnip accessions. We studied the anatomy of turnip tubers and measured carbohydrate composition of the mature turnip tubers of a subset of the collection. The variation in 16 leaf traits, 12 tuber traits and flowering time was evaluated in five independent experiments for the entire collection. The effect of vernalization on flowering and tuber formation was also investigated. SNP marker profiling basically divided the turnip accessions into two subpopulations, with admixture, generally corresponding with geographical origin (Europe or Asia). The enlarged turnip tuber consists of both hypocotyl and root tissue, but the proportion of the two tissues differs between accessions. The ratio of sucrose to fructose and glucose differed among accessions, while generally starch content was low. The evaluated traits segregated in both subpopulations, with leaf shape, tuber colour and number of shoots per tuber explaining most variation between the two subpopulations. Vernalization resulted in reduced flowering time and smaller tubers for the Asian turnips whereas the European turnips were less affected by vernalization.

## Introduction

Plant storage organs, such as grains, fruits, tubers, roots and rhizomes, are important for plant survival and represent the harvested parts of many crops. Since ancient times, roots and tubers have provided food for man and livestock. To date, intensive research on tuber formation has focused on crops like potato (*Solanum tuberosum*) [1], [2],[3],[4],[5],[6], sugar beet (*Beta vulgaris*) [7], [8], [9], [10], cassava (*Manihot esculenta*) [11], [12], [13] and radish (*Raphanus sativus*) [14], [15], [16], [17], and brought some insight in the mechanisms of storage organ formation. Especially in potatoes, tuber formation (tuberization) is well studied, and with the publication of the genome sequence in 2011 [18], [19], the molecular regulation of tuberization can be studied at the gene level.

*Brassica rapa* is an important crop adapted to agricultural settings worldwide, and displays enormous morphological diversity in the organs used for consumption. A variety of different forms has been selected for use as oilseeds, leafy vegetables and turnips [20]. This makes this crop an excellent model to study genetics and mechanisms underlying the morphological characteristics and their inter-relationships. It is a diploid species (AA = 2n = 20) with a relatively small haploid genome (485Mb), which is one of the closest crop relatives of the model plant species *Arabidopsis thaliana*. The *B. rapa* genome sequence facilitates comparative genomic studies to reveal causal genetic elements for its extreme morphological variation [21].

Turnips (*B. rapa* subsp. *rapa*) represent an important morphotype in the species *B. rapa*, and have been cultivated in Europe since 2,500-2,000 B.C. and spread to other parts of the world afterwards [22]. Turnips are either cultivated as fodder crop, where both leaves and tubers are consumed, or as vegetables, where, depending on the region, the leaves (turnip greens) and shoots (turnip tops) are consumed (southern European countries) or the tubers are consumed (northern and eastern Europe and China).The mechanisms underlying storage organ formation in turnips have not been studied so far.

Well described genetic variation and morphological characteristics of germplasm represent an important resource to study traits and a reservoir for breeders to develop new cultivars with desired characteristics. Previous studies on turnip germplasm focused on genetic relationships among different *B. rapa* accessions [23], [24], [25], [26], while data on morphological characterization are scarce, or focused on the composition of phytonutrients such as glucosinolates or sugars [27], [28]. Based on genetic diversity studies using molecular markers (amplified fragments length polymorphism fingerprinting, plus multi allelic microsatellite markers) and metabolite profiling, turnip accessions were clustered into two groups: one cluster consists of Asian turnips together with mainly pak choi (*B. rapa* subsp. *chinensis*); another cluster consists of European turnips with broccolettos (*B. rapa* L. “*Broccoletto* group”)[23], [24], [25].

The objective of the present turnip study, which includes both Asian and European accessions, is to evaluate various morphological traits (turnip anatomy, leaf, tuber and flowering time) and carbohydrate composition of the turnip tuber, and to analyse these in the context of their genetic relationships redefined using sequence-based bi-allelic SNP markers. Controlling flowering time is especially important in crop plants as it determines the geographical range where the crop can be cultivated and ensures high agricultural productivity. Biennial forms of turnips are planted in late summer/early fall and require vernalization (a period with low temperature) during the winter to flower in the following spring, whereas annual forms are planted in spring and flower in late summer. Therefore, both the effects of vernalization and day length on turnip development were measured in this turnip core collection.

## Materials and Methods

### Anatomy of turnip tubers

**Figure 1:**
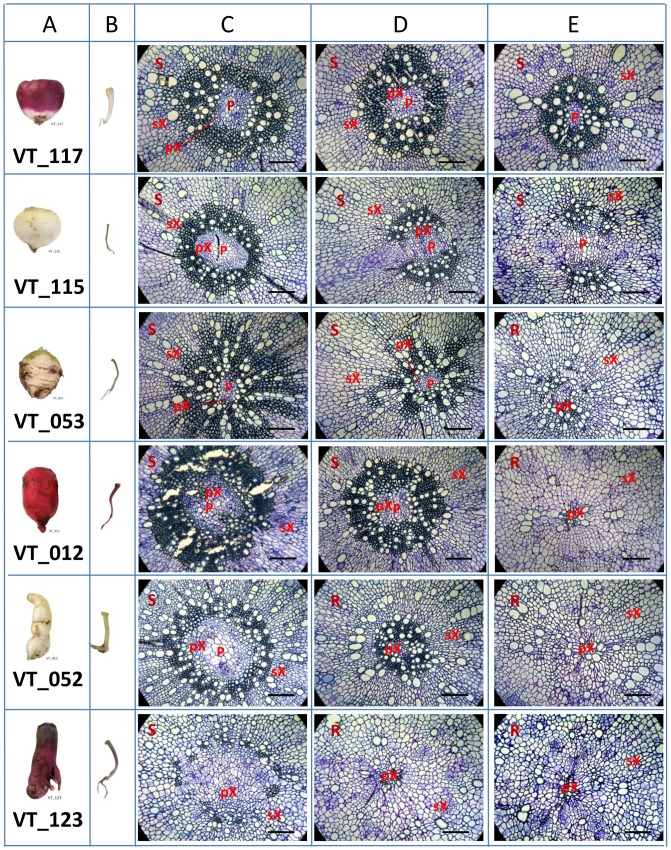
Mature turnips, 28 day old seedling turnips and cross sections of 28 day old turnips of six accessions. The mature turnip tuber and the 28 day old seedling are separated in column A and B of the corresponding accession; followed by sections derived from the turnip tuber of a 28 day old seedling displayed in column C, D and E. All light microscopic images are the middle field of each cross section with the scale bar standing for 100 µm. Column C shows the cross sections of the upper part of the tuber (5 mm below the cotyledons); D shows the middle part of the tuber; E shows the lower part of the tuber (5 mm above the bottom). At the left up corner of each section, S and R stands for stem and root structure respectively. P: Pith; pX: primary xylem; sX: secondary xylem. Most of the turnips show a more dense lignified zone of first formed secondary xylem around the pith (especially in VT_012, 053 and 117), and then the wood parts becomes very weakly lignified. Lignification is the strongest in the upper parts and declines towards the lower parts. Accession VT_123 does not show significant lignification in the wood cylinder.

To explore whether turnip tubers consist of stem, hypocotyl or root tissue, we chose six accessions (VT_052, VT_053, VT_012, VT_115, VT_117 and VT_123, the first two are European turnips and the other four are Asian turnips) based on their morphology and genetic relationships as determined in previous studies, to investigate the turnip anatomy. Three plants per accessions were grown in soil in the greenhouse and one representative plant per accession was used for anatomical sectioning at 28 days after sowing, while the remaining two plants were kept for macroscopic observation. Five millimetre thick pieces were sliced at three positions (upper, middle and lower part) of each turnip tuber (Figure 1). The sliced turnip tubers were first fixed in 5% glutaraldehyde in 0.1 M phosphate buffer (pH 7.2) for 2 h, washed in the phosphate buffer for 4×15 min and then in H_2_O for 2×15 min, dehydrated in a series of ethanol (10, 30, 50, 70, 90, 100% v/v, 2 h per step) under vacuum. Infiltration was carried out using 1∶1 v/v mixtures of ethanol and Technovit 7100 resin (Kylzer and Co. GmbH, D-6393 Wehrheim/Ts) with hardener I (benzyl peroxide) for 2 h and in 100% Technovit 7100 resin with hardener I for 24 h. Polymerization was carried out at room temperature in fresh Technovit 7100 plus hardeners I and II (dimethyl sulphoxide (DMSO)). Cross sections (5-7 µm) were cut with a Ralph knife on a LKB Historange rotary microtome and stained using 0.25% Toluidine blue (in 1% NaB_4_O_7_.10H_2_O). The cross sections were then examined with a bright-field microscope (Zeiss Axiophot) equipped with a digital camera at suitable magnification. Photographs were taken by the digital camera and analysed using software AxioVison LE Rel.4.6 (Carl Zeiss).

### Turnip collection

The turnip (*Brassica rapa* subsp. *rapa*) germplasm used for this study consisted of 56 accessions, mainly landraces from gene banks, including 43 vegetable and 13 fodder turnips, having different phenotypes, originating from 16 different European and Asian countries. Seeds of tested accessions were obtained originally from the Dutch Crop Genetic Resources Centre (CGN), the Vavilov Research Institute of Plant Industry (VIR), and the Russian State Agrarian University (RSAU) (Table 1). Genetic relationship, leaf traits and glucosinolate content of subsets of these accessions were described previously [23], [24], [25], [27] for different research purposes.

**Table 1:** Accessions used in five experiments and the proportion of variation of each accession derived from the different subpopulations.

Accession	Morph^a^	Collection^b^	Cultivar name	Genebank ID	Country of origin	Sub_population	Structure membership fractions		hetero%	2008F	2009F	2010G	2011F	2012F	Ver	C-C
FT_001	FT	WUR	Halflange Witte Blauwkop Ingesneden Blad-Barenza	CGN06669	Netherlands	European	0.989	0.011	10.9	x	x		x	x		
FT_002	FT	WUR	Grote Ronde Witte Roodkop-Norfolk; De Norfolk a Collet Rouge	CGN06673	United Kingdom	European	0.995	0.005	11.8	x	x	x	x	x	x	x
FT_003	FT	WUR	Lange Witte Roodkop	CGN06675	Netherlands	European	0.995	0.005	9.9	x	x			x		
FT_004	FT	WUR	Lange Gele Bortfelder	CGN06678	Denmark	European	0.993	0.007	11.2	x	x	x	x	x	x	x
FT_005	FT	WUR	Ochsenhorner	CGN06688	Germany	European	0.994	0.006	15.5	x	x		x	x		
FT_047	FT	WUR	Moskovskij	CGN06866	Soviet Union	European	0.995	0.005	16.7	x	x	x	x	x	x	x
FT_051	FT	WUR	Krasnaja	CGN07164	Soviet Union	European	0.518	0.482	22.2	x	x	x		x	x	x
FT_056	FT	WUR	Daisy; Bladraap	CGN07179	France	European	0.674	0.326	14.0			x	x	x	x	
FT_086	FT	WUR		CGN07223	Pakistan	European	0.555	0.445	17.4		x	x		x	x	
FT_088	FT	WUR	Blauwkop Heelblad-Oliekannetjes	CGN10985	Netherlands	European	0.991	0.009	14.7	x	x			x		
FT_097	FT	WUR	Buko; Bladraap	CGN11010	Germany	European	0.701	0.299	26.1	x	x	x	x	x	x	
VT_006	VT	WUR	Pusa Chandrina	CGN06709	India	Asian	0.451	0.549	22.4	x			x	x		
VT_007	VT	WUR	Maiskaja	CGN06710	Soviet Union	Asian	0.229	0.771	29.5	x	x	x	x	x	x	x
VT_008	VT	WUR	Pusa Chandrina	CGN06711	India	European	0.539	0.461	15.3	x	x	x	x	x	x	x
VT_009	VT	WUR	Ronde Rode -Tsutsui	CGN06717	Japan	Asian	0.015	0.985	18.8	x	x	x	x	x		x
VT_010	VT	WUR	Platte Ronde Blauwkop Ingesneden Blad- Lila Ker	CGN06718	Hungary	European	0.987	0.013	13.3	x	x	x	x	x		x
VT_011	VT	WUR	Platte Witte Blauwkop Ingesneden Blad-Siniaja	CGN06719	Soviet Union	European	0.994	0.006	7.9	x	x		x	x		
VT_012	VT	WUR	Ronde Rode Heelblad-Yurugu Red	CGN06720	Japan	Asian	0.053	0.947	15.2		x	x	x	x	x	x
VT_013	VT	WUR	Ronde Rode Heelblad-Scarlet Ball	CGN06721	Japan	Asian	0.015	0.985	16.9	x	x	x	x	x	x	x
VT_014	VT	WUR	Platte Witte Blauwkop Heelblad-Milan	CGN06722	Italy	European	0.995	0.005	14.5	x	x	x	x	x	x	
VT_015	VT	WUR	Bianca Lodigiana; Italiaanse Witte	CGN06724	Italy	European	0.996	0.004	15.1	x	x			x		
VT_017	VT	WUR	Platte Witte Meirapen	CGN06732	Netherlands	European	0.996	0.004	9.0	x	x	x	x	x	x	
VT_018	VT	WUR	Goudbal; Golden Ball	CGN06774	Netherlands	European	0.997	0.003	12.5	x	x	x		x	x	
VT_044	VT	WUR	Soloveckaja	CGN06859	Soviet Union	European	0.982	0.018	11.7		x	x	x		x	x
VT_045	VT	WUR	Milanskaja; Italiaanse Witte	CGN06860	Italy	European	0.994	0.006	12.7	x	x		x	x		
VT_052	VT	WUR	Hilversumse; Marteau	CGN07166	Netherlands	European	0.836	0.164	12.8	x	x	x	x	x	x	x
VT_053	VT	WUR	Teltower Kleine	CGN07167	Germany	European	0.979	0.021	13.2		x	x	x	x	x	x
VT_089	VT	WUR	D′Auvergne Hative	CGN10995	France	European	0.981	0.019	16.0	x	x	x	x	x	x	x
VT_090	VT	WUR	De Croissy	CGN10996	France	European	0.841	0.159	21.5	x	x			x		
VT_091	VT	WUR	Snowball; Blanc Rond de Jersey	CGN10999	United Kingdom	European	0.982	0.018	12.0	x	x	x	x	x	x	
VT_092	VT	WUR	Amerikaanse Witte Roodkop Heelblad	CGN11000	Netherlands	European	0.997	0.003	10.1	x	x		x	x		
VT_115	VT	WUR	Kairyou Hakata	CGN15199	Japan	Asian	0.01	0.99	21.4	x		x	x	x	x	x
VT_116	VT	WUR	Nagasaki Aka	CGN15200	Japan	Asian	0.131	0.869	12.6		x			x		
VT_117	VT	WUR	Toya	CGN15201	Japan	Asian	0.009	0.991	21.6	x	x	x	x	x	x	x
VT_119	VT	WUR	Roodkop-Pfalzer	CGN15209	Netherlands	European	0.908	0.092	16.0	x	x			x		
VT_120	VT	WUR	Platte Gele Boterknol	CGN15210	Netherlands	European	0.991	0.009	8.4	x	x	x	x	x	x	
VT_123	VT	WUR	Terauchi-Kabu	CGN15220	Japan	Asian	0.007	0.993	17.0	x	x	x	x	x	x	
VT_137	VT	WUR		CGN20735	Uzbekistan	European	0.659	0.341	19.2	x	x	x	x	x	x	
SM_14	VT	RSAU	JE 4-11		Cross of European and Japanese turnips	Asian	0.479	0.521	8.9			x		x		
SM_15	VT	RSAU	Petrovskaya 1-122		Russia	European	0.995	0.005	15.2			x		x		x
SM_16	VT	RSAU	JJE 2-1		Cross of European and Japanese turnips	Asian	0.383	0.617	11.5			x		x		
SM_17	VT	RSAU	Iz 2-11		Israel	European	0.994	0.006	7.8			x		x		
SM_18	VT	RSAU	Petrovskaya 34-12		Russia	European	0.996	0.004	12.4			x		x		
SM_19	VT	RSAU	Petrovskaya 1-35		Russia	European	0.988	0.012	6.7			x		x		
SM_20	FT	RSAU	ECD 04-0126		European Clubroot Differential Set	European	0.985	0.015	2.2			x		x		
T_1050V	VT	VIR	Volynskij	1050	Ukraine	European	0.991	0.009	10.7	x	x	x		x	x	
T_1283V	VT	VIR	Zolotoj shar	1283	Netherlands	European	0.99	0.01	13.9	x	x	x		x	x	
T_163V	PC	VIR	Local	163	China	Asian	0.016	0.984	20.5	x		x		x		
T_307V	VT	VIR	Osterzundomskij	307	Russia	European	0.996	0.004	15.9	x	x	x		x	x	
T_385V	VT	VIR	Bartfeldskij	385	Ukraine	European	0.997	0.003	10.6	x	x	x		x		
T_738V	VT	VIR	Karelskaya	738	Russia	European	0.737	0.263	17.6	x	x	x		x		x
T_821V	VT	VIR	Grobovskaya	821	Russia	Asian	0.213	0.787	14.1	x		x			x	
T_826V	VT	VIR	Milanskaya belaya	826	Russia	European	0.992	0.008	15.9	x		x		x		
T_830V	VT	VIR	Petrovskaya	830	Russia	European	0.997	0.003	11.2	x	x	x			x	
T_pancai	VT	WUR	Wenzhoupancai		China	Asian	0.2	0.8	2.2		x			x		
T_RR	VT	WUR	Red Round Turnip		China	Asian	0.072	0.928	23.1					x		

The accessions used in each experiment are marked with “x”. “Hetero%” indicates the percentage of heterozygous SNP loci detected from genotyping by 280 SNP markers. Experiment code “2008F, 2009F, 2011F and 2012F” stand for four field experiments carried out between 2008 and 2012. Code “2010G” means the greenhouse experiment in 2010. “Ver” and “C-C” stand for experiment of testing vernalization response and carbohydrate composition of turnip accessions, respectively.

a FT stands for fodder turnips and VT stands for vegetable turnips.

b WUR  =  Wageningen University and Research Center; VIR  =  Vavilov Research Institute of Plant Industry; RSAU  =  Russian State Agrarian University.

### Marker development and genotyping

Pair-end resequencing data of three libraries, with insert sizes of 300 bp, 500 bp and 2,000 bp for four genotypes of *B. rapa*, one oil type rapid cycling DH line L144 (subsp. *oleifera*), line R-o-18 (subsp. *trilocularis*), a leafy type Wutacai (subsp. *narinosa*) and DH_VT_117 (subsp. *rapa*), a DH line of a Japanese vegetable turnip CGN15201, were generated on an Illumina HiSeq 2000 platform. Genome-wide *B. rapa* SNPs were detected by comparing whole genome resequencing data from these four *B. rapa* accessions to the reference genome of Chiifu-401-42 (subsp. *pekinensis*) [21]. A strategy was employed by using the reference genome as a ‘bridge’ to sequentially detect the SNPs. The resequencing data of L144, DH_VT_117, R-o-18 and Wutacai were aligned to the reference respectively, by SOAP with default setting, except no gaps were allowed [29]. To avoid false detection of polymorphisms, multiple-hit reads were filtered out from the dataset. Then, the results of alignment were used to obtain consensus sequences of each *B. rapa* accession using software SOAPsnp [30] with default parameters, and the requirement that each reliable SNP was covered by at least three pair-end reads in each accession. Using the consensus sequence dataset, we then detected SNPs between DH_VT_117 and the three genotypes R-o-18, L_144 and Wutacai. Sequences of 200 bp flanking the SNP site were used to design primers. Genotyping was conducted using the KASPar SNP genotyping technology (http://www.kbioscience.co.uk), with a competitive allele specific polymerase chain reaction system according to the manufacturer's instructions. In total 280 SNP markers randomly distributed over the *B. rapa* genome were selected and screened over the turnip accessions (Table S1).

The number of subpopulations was determined using the software STRUCTURE 2.2.3 [31]. A model with population admixture was tested, which assumes that genotypes can have a mixed ancestry, and assumes independent allele frequencies between subpopulations. The number of subpopulations was set to vary from one to six, and for each fixed number of subpopulations, five independent Markov Chain Monte Carlo processes were run using 100,000 interactions for each with burn in of 10,000. We calculated the statistic ΔK, which indicates the highest level hierarchical structure in the population. To perform ΔK calculations, CorrSieve software 1.6-3 [32] was used which can summarize the K statistic directly from STRUCTURE outputs. The population K that has the highest value of ΔK is considered as the meaningful number of subpopulations [31].

### Phenotyping

Five experiments were conducted between 2008 and 2012 (2008F, 2009F, 2010G, 2011F and 2012F), in which different subsets of the 56 turnip accessions were tested to compare seasonal and environmental effects on growth and development of the accessions (Table 1). The turnip accessions were planted and grown in the field in Wageningen, The Netherlands from May to October in 2008F and 2009F, and from June to November in 2011F and2012F. Experiment 2010G was conducted from February to August 2010 in a greenhouse with controlled conditions (21/18°C day/night and 16 h light) at Unifarm, Wageningen UR. Seeds were germinated in petri-dishes with moist filter paper and then transferred into 5 cm^3^ size peat blocks in the greenhouse and were transplanted to the field or into pots with soil in the greenhouse at the third leaf stage. Details of experimental design, temperature and day length information in the growth period of each experiment are described in Table S2 and Figure S1.

Morphological traits including 16 leaf traits, 12 turnip tuber traits and flowering time (FT) were recorded according to the description in Table 2. Flowering time of the plants which did not flower before harvesting was set to 150 days. Different traits were evaluated per experiment; traits related to the turnip tuber size, tuber shape (tuber width, length and weight) and leaf shape (leaf index) were evaluated in at least three independent experiments (Table 2). In all five experiments, digital pictures were taken from fully expanded leaves of each plant (the fifth or sixth true leaf) and analysed with ImageJ software [33]. Tuber width and length were measured using a digital calliper at the widest and longest position of each tuber.

**Table 2:** List of evaluated phenotypic traits in five experiments.

Trait type	Trait code	Trait name	Trait description	Experiment a					
				2008F	2009F	2010G	2011F	2012F	Ver
Leaf traits	LC	Leaf color	Chlorophyll content of leaves using SPAD meter	x	x	x			x
	LL	Leaf length	Length from base of petiole to tip of lamina (cm)	x					
	LBL	Lamina blade length	Distance from the tip to the fist lobe (cm)	x		x			x
	LBW	Lamina blade width	Lamina width at the widest point (cm)	x		x			x
	LI	Leaf index	Ratio of LBL/LBW	x		x			x
	PL	Petiole length	Distance from the base of the petiole to bottom of lamina (cm)	x					
	PW	Petiole width	The width of petiole (midvein)	x		x			x
	LB	Leaf Lobe	Number of lobes below lamina blade	x					
	LBs	Leaf Lobelets	Number of lobelets formed below the lobes	x					
	LES	Leaf edge shape	1: non serrated; 2: slightly serrated; 3: intermediate serrated; 4: very serrated	x		x			x
	LS	Leaf blade shape outline	1: orbicular; 2: elliptic; 3: obovate; 4: spathulate; 5: ovate; 6: lanceolate; 7: oblong; 8: others	x					
	LD	Leaf division (incision)	1: entire; 2: sinuate; 3: lyrate; 4: lacerate; 5: others	x					
	LAS	Leaf apex shape	1: acute; 2: intermediate; 3: rounded; 4: broadly rounded	x					
	LH	Leaf hairness	1: absent; 2: sparse; 3: intermediate; 4: abundant	x					
	LAT	Leaf lamina attitude	1: extreme deep down curling; 2: deep down curling; 3: little down curling; 4: flat; 5: up curling			x			x
	Lwe	Leaf and stem weight	Fresh weight of stem and leaves 100 days after transplanting (gram)			x			x
Flowering time	FT	Flowering time	Days to flowering from transplanting to appearance of the first open fower (days)	x	x	x			x
Turnip tuber traits	TL	Tuber length	Length from the base of cotyledons till the bottom of the swelling tuber (mm)	x	x		x	x	
	Twi	Tuber width	The widest diamer of the swelling tuber (mm)	x	x	x	x	x	x
	TI	Tuber index	Ratio of Twi/TL	x	x		x	x	
	TS	Tuber shape	1: oval; 2: round to oval; 3: round; 4: round to long; 5: long; 6: slim long	x					
	TC	Tuber color	1: white; 2: light green; 3: cream; 4: yellow or light brown; 5: pink or light purple; 6: purple; 7: dark purple; 8: red; 9: dark red	x					
	Tsh	Tuber shoots number	Number of shoots/stems formed from a turnip tuber		x	x	x		x
	Twe	Tuber weight	Fresh weight of a turnip tuber (gram)		x	x		x	x
	TDW	Tuber dry weight	Dry weight of a turnip tuber (gram)					x	
	Tss	Tuber surface smoothness	1: smooth; 2: a bit wrinkled; 3: very wrinkled			x			x
	Tso	Tuber swelling onset	Number of days from transplanting till an obvious swelling of a turnip tuber			x			x
	Tgd	Tuber growing depth	Depth in the soil at whick the turnip tuber grows. 1: underground; 2: half above ground; 3: above ground			x			x
	TDM	Tuber dry mass%	Percentage of total dry mass in a turnip tuber					x	

The evaluated traits in each experiment are marked with “x”.

a Experiment code “2008F, 2009F,2011F and 2012F” stand for four field experiments carried out between 2008 and 2012. Code “2010G” means the greenhouse experiment in 2010. “Ver” stands for experiment of testing vernalization response of turnip accessions.

### Principal component analysis

Principal component analysis was used to visualize the variation over the accessions for the morphological traits. In this analysis, flowering time data were not included, because many accessions did not flower by the time of harvest and therefore cannot provide informative flowering time data. Bi-plots were used as a tool to visualize the variability of traits and the correlation between traits, as well as their space in relation to groupings of accessions. Analyses were conducted using R statistical software (www.R-project.org).

### Heritability analysis

To better understand the relative contribution of genotype and environment to the observed phenotypic variation, we adapted the classical equation to calculate heritability (*h^2^*). For the traits measured in more than one experiment (environment), to calculate the variance component, a linear mixed model was employed, because of the unbalanced experimental design for the different experiments. The heritability was defined as the proportion of phenotypic variance that is determined by the ratio of genotype variance (σσ^2^_acc_) by total variance (genotype variance (σσ^2^_acc_) plus genotype by environment interaction variance (σσ^2^_acc.env_) plus other causal variance (σσ^2^_e_) (equation 1). Equation 2 was used when calculating heritability within one experiment. For the traits that were measured in only one experiment, ANOVA was used to show the homogeneity of the phenotypes per accession. All analysis was carried out using a statistical package GenStat 16^th^ Edition (VSN International, Hertfordshire).
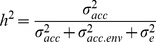
(1)

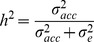
(2)


### Hierarchal cluster analysis

Analysis of the auto-scaled data, correlations between morphological variables and hierarchical clustering using the un-weighted pair group method with arithmetic averages (UPGMA) of the accessions were performed using Genemaths XT (Applied Maths, Belgium). The dissimilarity matrix was calculated based on Euclidean distances between the morphological variables.

### Vernalization response

To investigate vernalization response of the turnip collection in the greenhouse experiment in 2010 (2010G), a subset of 29 accessions that represented genetic diversity of the whole collection was tested for the response to four weeks and eight weeks vernalization treatments (Table 1). Seeds were sown in petri-dishes with moisture filter paper and the germinated seeds were placed at 4°C in the dark for four or eight weeks before transplanting into soil. Transplanting date was synchronized, by sowing seeds in three patches with four weeks' time intervals. Four biological replications were included per accession per treatment with a complete randomized block design. All turnip plants were harvested at 110 days after transplanting and the turnip tubers were immediately sliced to uniform parts including the outer and inner tissues, pooled per accession into a corning tube, and then immediately frozen in liquid nitrogen. The samples were freeze-dried and milled to fine powder, then stored at –20°C for further carbohydrate composition analysis.

### Determination of soluble sugars and starch

Eighteen accessions were included in carbohydrate composition analysis (Table 1). Freeze-dried turnip tuber powder from experiment 2010G was weighed, 5 mg was suspended into 1 ml 80% ethanol and incubated for 60 minutes in a shaking water bath at 70°C. The centrifugation at 13000 rpm for 10 minutes separated the supernatant, which contains soluble sugars. Soluble sugars were quantified using a UV-method for determination of sucrose/d-glucose/d-fructose (Boehringer Mannheim, Kit 716260) following the manufacturer's instructions.

Starch was solubilized from 50 mg freeze-dried powder according to the protocol described in Salehuzzaman et al,. 1992 [34]. The quantity of starch was determined using a UV-method which measured glucose released from starch (Boehringer Mannheim, Kit 207748).

## Results

### Anatomy of turnip tubers

At 28 days after sowing, seedlings had three to four expanded leaves, while the hypocotyl tissue started to expand. Cross sections for the tubers of six turnip accessions were made at three positions to reveal whether the turnip tubers were homologous to stems or roots (Figure 1). In all cross sections made from these six accessions, secondary growth was clearly observed, illustrated by a vascular cambium producing secondary xylem and secondary phloem (Figure S2). Sections from all three positions of VT_117 and VT_115 tubers and the upper part (position a) from the other four accessions showed that the tubers consist of stem like structures, as the pith cells were clearly observed. In the middle position (b) of tubers from VT_012 and VT_053 the pith cell area was reduced and the xylem cells were bigger and started to form a star shape in the centre of the tuber, which is reminiscent of root tissue. A typical secondary root structure was observed in sections from the lower part (position c) of VT_012, VT_053 and the middle and lower part (position b and c) of VT_052 and VT_123. In conclusion, turnip tubers of the six accessions consist of different proportions of hypocotyl and root tissues (Figure 1).

**Figure 2:**
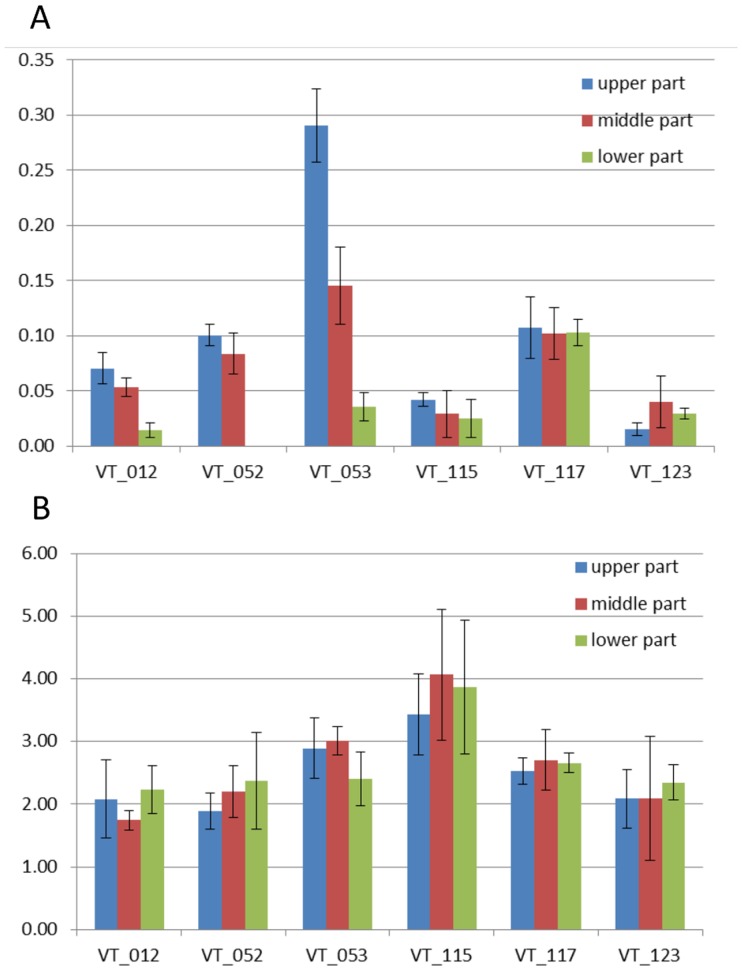
Comparison of lignification ratio (darker stained proportions in the wood cylinder) (A) and xylem/phloem ratio (B) for the six turnip accessions. Error bars stand for standard deviation.

The most lignified cells in the wood cylinder stain dark blue and can be seen in most sections with different proportions for different accessions, indicating that lignification occurs during the growth of turnip tubers (Figure 1). The lignified area decreased from the top to the bottom of the turnip tuber for all six accessions (Figure 2 A). VT_053 showed the highest proportion of lignification, while VT_052, VT_115 and VT_123 showed the lowest proportion of lignification (Figure 1, 2A and Table S3). The diameter of xylem and phloem area was calculated by averaging five measurements taken in the radial plane of transverse sections of each picture. The tuber of VT_115 had the highest proportion of xylem (∼3.5x), while the other five accessions had lower ratios (∼2–2.5x) between xylem and phloem (Figure 2 B, Figure S2 and Table S3). These data illustrate that neither lignification nor the proportion of xylem and phloem are related to the tissue origin of the turnip tuber.

### Genetic variation

**Figure 3:**
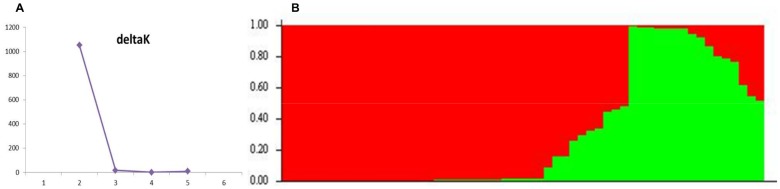
STRUCTURE analysis of 280 SNP markers over 56 turnip accessions. (A) Result of the K calculation, deltaK for K = 2-6 revealed a single distinct peak at K = 2; (B) Plot of the raw STRUCTURE output for two subpopulations (K = 2).

Genotyping result of the 56 turnip accessions is presented in Table S4 and the genetic structure of this turnip collection was inferred using 280 SNP markers randomly distributed over the ten chromosomes of the *B. rapa* genome. The Bayesian model-based clustering implemented in STRUCTURE software revealed two subpopulations. For all K, memberships were consistent between all runs. The ΔK graph, indicates the presence of two subpopulations (K = 2) (Figure 3). Population 1 includes 15 accessions mostly from Asian origin, except for T_821V, which originates from Russia. Population 2 includes the remaining 41 accessions mainly from European origin, except FT_086, VT_008, VT_137 and SM_17, which is derived from Pakistan, India, Uzbekistan and Israel respectively (Table 1).

Of the 56 accessions, 48 were assigned to a group with a probability value of *p*>0.70. Eight accessions have *p*<0.70 probability values with different levels of admixture between subpopulations. Three accessions with admixture were those originating from Asia but classified to the group with mostly European accessions, or vice versa. SM_14 and SM_16 were inbred lines developed from a hybrid between Russian and Japanese turnips, which explains their admixed probability value (Table 1).

The heterozygosity level of each genotyped plant was calculated by dividing the number of SNP markers that were heterozygous over the total number of loci with genotype information. Heterozygosity ranged from 2.2% to 29.5%, and the top ten near-homozygous accessions included one landrace T_pancai, four inbred lines (SM_14, 17, 19 and SM_20) and five European accessions (Table 1).

### Morphological variation in the collection

The morphological variation of the 56 turnip accessions was evaluated based on 16 leaf traits, 12 turnip tuber traits and flowering time, in four independent field experiments and one greenhouse experiment over different years (Table 2 and Figure S3). Supplementary data provides detailed phenotypic values for each trait in the five experiments (Table S5 and Table S6) and the photo displays of leaf and tuber variation of all the 56 accessions (Figure S4 and Figure S5).

The heritability was calculated for the eleven traits which were measured in more than one experiment, to explain the relative contribution of genotype and environment to the total phenotypic variation (Table S7). Three traits, tuber index (TI, 0.62), tuber length (TL, 0.48) and flowering time (FT, 0.46) showed relatively high heritability, while low heritability (0∼0.29) suggesting extensive environmental effects was observed for traits petiole width (PW), leaf blade length (LBL), tuber weight (Twe), tuber width (Twi), tuber shoot number (Tsh), leaf color (LC), leaf blade width (LBW) and leaf index (LI) (Table S7).

**Figure 4:**
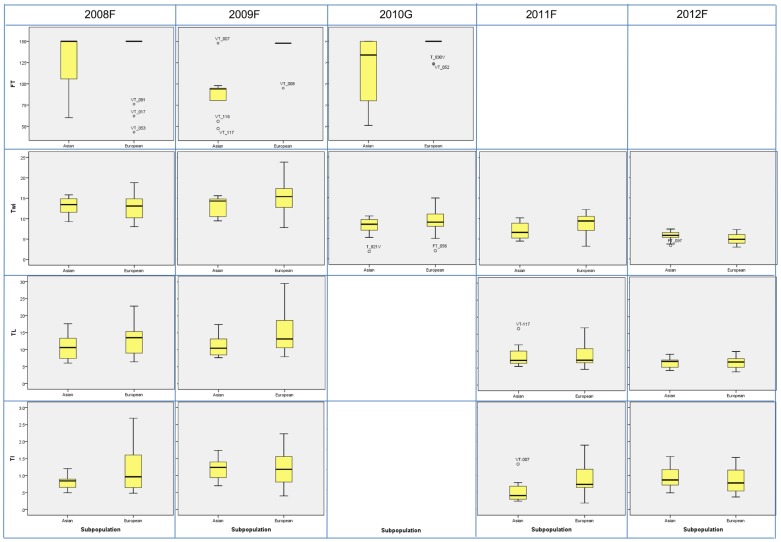
Boxplots for visualizing the comparison between Asian and European subpopulations, for the traits flowering time (FT), turnip width (Twi), turnip length (TL) and turnip index (TI) evaluated in five independent experiments. 2010G was carried out in the greenhouse in 2010 while other experiments were carried out in the open field between 2008 and 2012. Experimental set up and conditions are provided in Table S2 and Figure S1.

The variation per trait within the Asian or European subpopulations is listed in Figure 4 and Table S8. Traits leaf apex shape (LAS), leaf lamina attitude (LAT), leaf division (LD), leaf blade shape outline (LS), leaf length (LL), petiole width (PW), leaf color (LC), tuber length (TL), tuber dry mass% (TDM), tuber growth depth (Tgd), tuber shape (TS) and tuber surface smoothness (Tss) showed similar median values between the two subpopulations, whereas for the traits leaf blade length (LBL), leaf blade width (LBW), leaf index (LI), petiole length (PL), leaf lobes (LB), leaf lobelets (LBs), leaf edge shape (LES), leaf shape (LS), leaf hairness (LH) and leaf and stem weight (Lwe) clear differences were observed between Asian and European turnips (Figure 4 and Table S8). Our data indicated that leaves of accessions belonging to the European subpopulation had higher number of lobes (LB) and lobelets (LBs) and had more leaf hairs (LH), a shorter petiole (PL) and rounder shaped leaf lamina (lower median value in LBL and LI, but higher value in LBW) compared to accessions from the Asian subpopulation. The weight of total leaves and branches (Lwe) was higher for the Asian subpopulation than for the European subpopulation, which might be related to the fact that most accessions from the Asian subpopulation were flowering and setting seed pots when evaluated, while most accessions from the European subpopulation were not yet flowering (Figure 4 and Table S8) and still forming leaves.

Asian turnips flowered much earlier than the European accessions in three experiments (2008F, 2009F and 2010G), however the range of flowering time for the Asian turnips was larger in 2008F and 2010G compared to 2009F: the median value for 2009F was 94 days, which was earlier than in the other two experiments (150 days and 135 days) (Figure 4 and Table S8). Asian turnips tended to form higher number of shoots per tuber than the European turnips in experiments 2009F, 2010G and 2011F, although in 2009F, the range in number of shoots was much larger (5–23) (Table S8). In 2009F, 2010G and 2011F, European turnip tubers were bigger than Asian turnip tubers, while in 2008F tuber size tended to be similar between the two subpopulations and in 2012F, when the tubers were harvested earlier than in the other experiments (75 days after sowing) (Figure 4 and Table S8). For both subpopulations, the shape of the turnip tuber reflected by the trait tuber shape (TS) in 2010G and tuber index (TI) in the other experiments was very similar, although the accessions of the European subpopulation showed a slight tendency of being more flat (higher value of TI) in two (2008F and 2011F) out of four experiments (Figure 4 and Table S8). In conclusion, the European turnips and the Asian turnips mainly differ in flowering time, onset and speed of turnip tuber formation, and the shape of their leaves.

**Figure 5:**
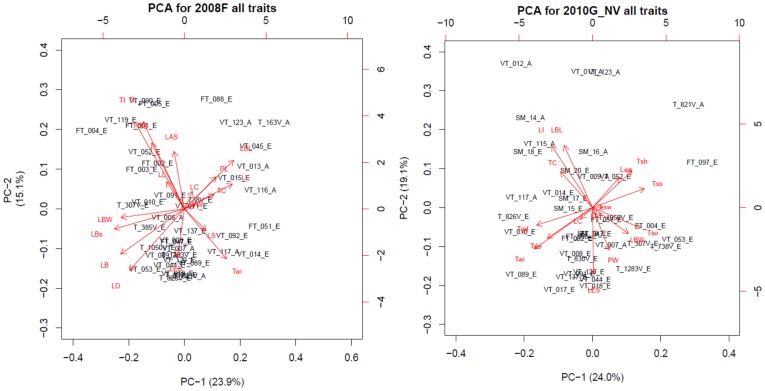
Principal component analysis (PCA) diagram showing the phenotypic variation and the contribution of different traits for the tested turnip accessions of the field experiment in 2008 (2008F) and the greenhouse experiment in 2010 (2010G). The presented traits are leaf color (LC), leaf length (LL), lamina blade length (LBL), lamina blade width (LBW), leaf index (LI), petiole length (PL), petiole width (PW), leaf lobe (LB), leaf lobelets (LBs), leaf edge shape (LES), leaf blade shape outline (LS), leaf division (LD), leaf apex shape (LAS), leaf hairiness (LH), leaf lamina attitude (LAT), leaf and stem weight (Lwe), flowering time (FT), tuber length (TL), tuber width (Twi), tuber index (TI), tuber shape (TS), tuber color (TC), tuber shoots number (Tsh), tuber weight (Twe) tuber surface smoothness (Tss), tuber swelling onset (Tso) and tuber growing depth (Tgd). Red arrows represent the contribution of different traits to the total variation. Percent of variation explained by each dimension is indicated. The name of accession with either _A or _E extension refers to Asian or European subpopulation, respectively.

Principal component analysis (PCA) was used to visualize the contribution of the different traits to the phenotypic variation and to compare the phenotypic variation of the Asian and European subpopulations (Figure 5 and Figure S6). The trait flowering time was left out of the PCA analysis as most of turnip accessions (especially the European accessions) were not flowering by harvest time. In experiments 2008F and 2010G, both leaf traits and tuber traits were scored and included in the analysis; the first dimension explains about 24% and the second dimension explains 15–19% of the variation in both experiments. The Asian subpopulation was not tightly clustered together, however a loose grouping in the upper (2010G) or right (2008F) quadrant was observed, which was explained by the variance in traits leaf lamina length (LBL), leaf index (LI), tuber color (TC) and number of shoots per tuber (Tsh) (Figure 5). Accessions belonging to the European subpopulation did not form a group but were distributed over the whole PCA diagram indicating high variation within this subpopulation (Figure 5). Experiments 2009F, 2011F and 2012F included mainly the tuber related traits and in PCA the first dimension explains 34-50% and the second dimension explain around 30% of the variation. Similarly as shown in Figure 4, the Asian subpopulation tended to form a loose grouping in 2009F and 2011F, with tuber shoot number Tsh as explaining variable (Figure S6). Whereas, when turnips were grown when days shortened and were harvested earlier (75 days, 2012F), no grouping of the two subpopulations was visible, suggesting similar variation of turnip tuber size and dry matter across all turnip accessions tested in this experiment (Figure S6). Again this shows that differences between European and Asian turnips are most obvious when turnips are evaluated after longer growth periods.

To explore the relationship between flowering time (FT) and tuber characteristics, we performed additional correlation analysis only for FT, tuber width (Twi), tuber weight (Twe), tuber shoot number (Tsh) and tuber swelling onset (Tso) data obtained in 2008F, 2009F and 2010G, by transforming FT data into three categories: early flowering (FT<70 days); intermediate flowering (70<FT<140) and late flowering (FT>140). In 2008F and 2010G, no significant correlation was detected between FT and traits explaining tuber size (Twi and Twe). Whereas in 2009F, a moderate correlation between FT and Twi (*r* = 0.306, *p* = 0.046) and a significant negative correlation between FT and Tsh were detected (*r* = −0.430, *p* = 0.004).

### Vernalization effects on flowering and turnip tuber formation

**Figure 6:**
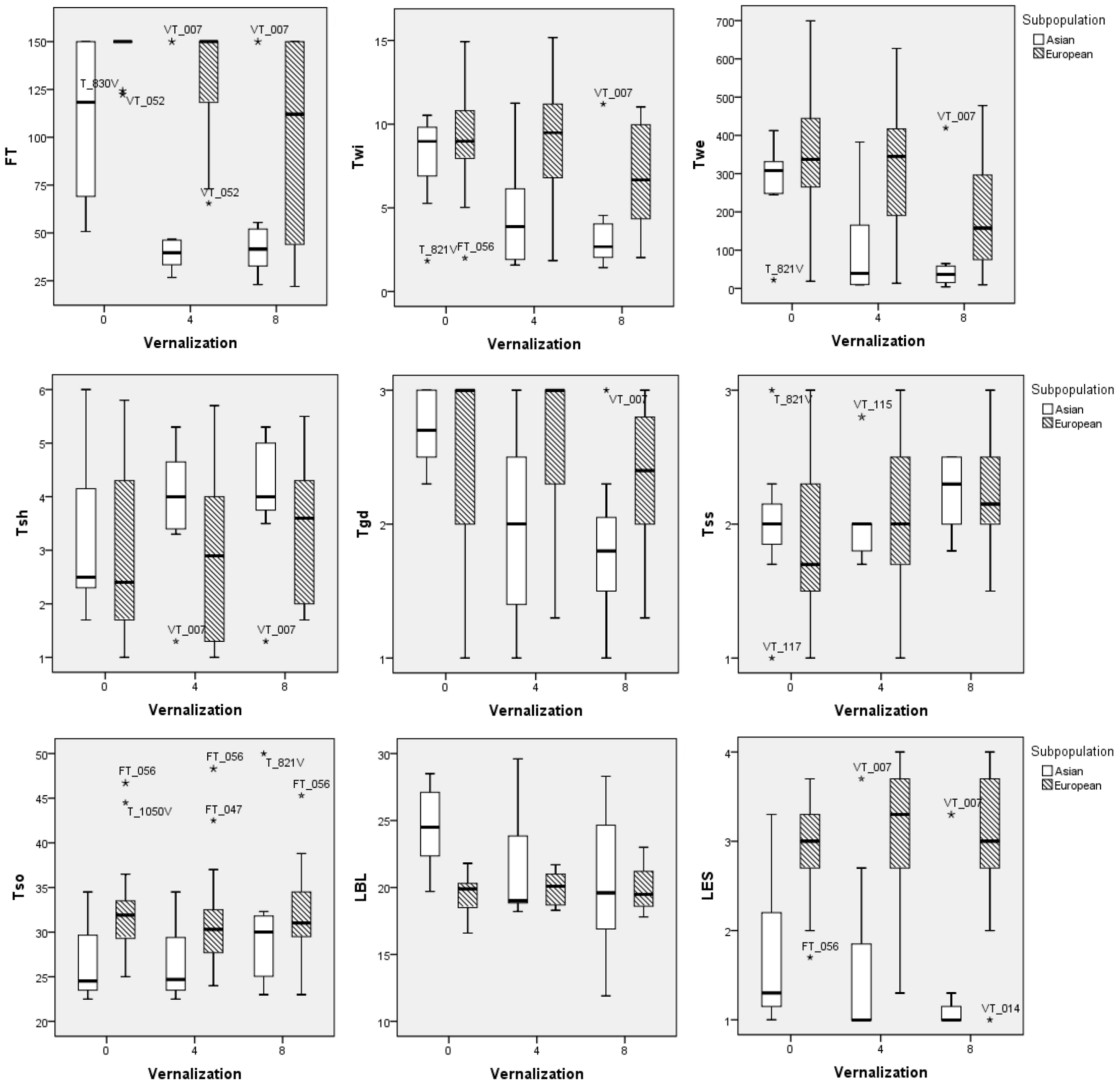
Boxplots showing vernalization responses of 29 turnip accessions for nine different phenotypic traits to no vernalization, four week vernalization and eight week vernalization. The presented traits are flowering time (FT), tuber width (Twi), tuber weight (Twe), tuber shoots number (Tsh), tuber growing depth (Tgd), tuber surface smoothness (Tss), tuber swelling onset (Tso), lamina blade length (LBL) and leaf edge shape (LES). Open and striped boxes stand for Asian and European subpopulation respectively. Outliers are marked using asterisks.

To determine the response of different accessions to vernalization, we performed a greenhouse experiment with 29 turnip accessions (7 Asian and 22 European turnips) selected according to their genetic relationship and morphological variation. Vernalization affected flowering time (FT) and the Asian and European subpopulations responded differently to the length of the vernalization period (Figure 6 and Figure S7). The Asian turnips flowered earlier when vernalized four weeks and extended vernalization time did not further accelerate flowering. However, the European turnips flowered earlier only when eight weeks vernalization was applied. Vernalization influenced turnip tuber width (Twi) and weight (Twe) differently for both subpopulations. Asian turnips formed smaller and lighter tubers after four weeks vernalization, while eight weeks vernalization resulted in an even smaller tuber. In nine European accessions and VT_007, even eight weeks vernalization did not affect turnip tuber size (Twi and Twe) and flowering time, as no significant differences were found for those traits between vernalization treatments (Table S9).

In addition, for both subpopulations, vernalized plants formed more shoots per tuber. The 8-week-vernalized plants formed tubers relatively later (Tso) and deeper (Tgd) in the soil; tuber surface (Tss) also became wrinkled after vernalization. Leaves of the Asian turnips also responded to vernalization with increased lamina length (LBL) and shape (reflected by LI), while leave morphology of the European turnips was insensitive to vernalization. Neither subpopulation showed obvious response to the vernalization for the other recorded leaf traits, leaf blade width (LBW), petiole width (PW), leaf edge shape (LES), leaf color (LC), leaf lamina attitude (LAT) and leaf and stem weight (Lwe) (Figure 6 and Table S9).

### Sugar and starch content in turnip tubers

**Figure 7:**
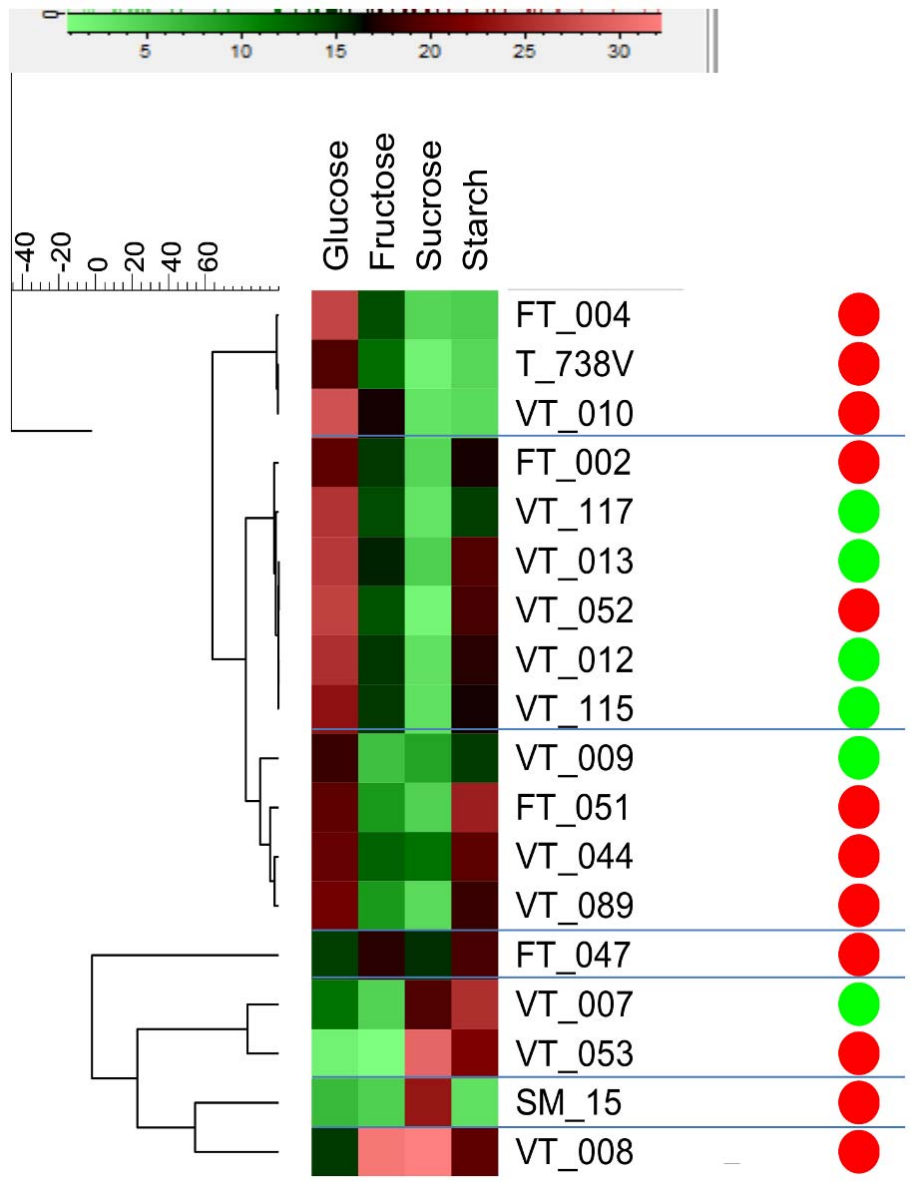
Hierarchical clustering in UPGMA obtained based on sugar and starch content measured on turnip tubers from 18 turnip accessions. Color gradient indicates the percentage of each carbohydrate in the freeze-dried mature tuber samples. Green and red dots on the left side indicate Asian and European subpopulations respectively.

Sugar and starch content of the mature tubers of a selection of 18 turnip accessions (Table 1) (6 Asian and 12 European) was determined. Total sugar content (glucose, fructose and sucrose) ranged from 32.8% (VT_053) to 48.2% (VT_010) of total freeze-dried weight of 17 out of 18 accessions, while VT_008 had a much higher level of 78.7%. Hierarchical clustering using UPGMA revealed three patterns of sugar levels for the tested 18 accessions, and this clustering did not correspond to the subpopulation structures (*p* = 0.713) (Figure 7). For the sugars, three clusters were observed: Cluster I included nine accessions with high glucose, intermediate fructose and low sucrose levels; Cluster II consists of four accession with high glucose, but low in both fructose and sucrose; accessions in Cluster III had high sucrose levels while glucose and fructose levels were low (FT_047, VT_007, VT_053, SM_15 and VT_008) (Figure 7). Starch content in turnip tubers was very low, ranging from 0.36% (SM_15) to 2.54% (VT_007) of the dry weight.

## Discussion

### Anatomical observations reveal that turnip tubers are not homologous structures

Understanding which part of a turnip plant develops into a tuber during the vegetative development is a key for future research investigating this phenomenon. Previous studies often stated that the turnip tuber is a taproot [28], [35], [36], [37], while a few studies mentioned that the thickened part of turnip consists of both hypocotyl and root [38], [39]. Our anatomical observations of six genetically diverse turnip accessions showed that turnip tubers are a combination of hypocotyl and root; both organs take part in forming the fleshy organ through secondary growth by a vascular cambium, while the proportion of hypocotyl/root differs among different accessions (Figure 1) and seems to be independent from the geographic origin of the turnip accession. The anatomy of other tuber crops like radish [15],*B. napus* swede, and sugar beet resemble turnip tubers in this, while tubers from potato and kohlrabi (*B. oleracea* subsp. *gongylodes*) constitute only stem tissue and carrot (*Daucus carota* L.) constitutes root tissue [40], [41]. Although our study on lignification in the tubers is limited and concentrated in the first formed wood of the upper tuber parts, our observation on the presence of different degrees of lignification of the xylem from all six turnip accessions suggests that lignin biosynthesis is an important aspect of turnip tuber development. In a study of sweet potato (*Ipomoea batatas*) it was suggested that lignification was the major limitation to tuberization of both roots and stem [42].

### Carbohydrate composition of turnip tubers

The ratio of the sugars glucose, fructose and sucrose differed among the tested accessions, which did not correspond to the subpopulation structure nor to the tissue composition of the turnip (root or hypocotyl) (Figure 7). Earlier research on one Indian turnip accession reported detailed information for sink development and carbohydrate status during plant development [35]. The authors pointed out that the turnip tuber has a high fructose and glucose content which might be due to high acid invertase activity in the tuber. In our study, 13 out of 18 turnip accessions were comparable to this Indian turnip with the hexoses glucose and fructose as primary sugar compound, while the sucrose level was low. The sugar composition of these turnip tubers is comparable with fruits of domesticated tomato, which accumulate primarily glucose and fructose, whereas some wild tomato species store sucrose [43]. The sugar composition of accessions with relatively high sucrose levels (FT_047, VT_007, VT_053, SM_15 and VT_008) is comparable with that of sugar cane (*Saccharum* spp.) stems [44], sugar beet tubers [45] and carrots [46]. The starch level in the tested 18 turnips ranged from 0.36% to 2.54%, which was higher than reported by [35]. This is probably due to the later harvest in the present study (100 days vs. 66 days). The starch level in the turnip tuber (0.36% to 2.54% dry mass) is 10 times lower than the starch level in potato tubers [47]. Gupta and co-authors reported a steep decline in sucrose synthesis activity in turnip tubers during rapid sink filling and mentioned its possible relation with the negligible starch biosynthesis [35].

### Genetic variation

Based on allele frequency of 280 SNP markers evenly distributed over the *B. rapa* genome, two subpopulations were revealed: European turnips and Asian turnips. The accessions with different levels of admixture between the two subpopulations also showed admixture in previous studies (Table 1). In the previous studies population structure was inferred in a collection comprising all different *B. rapa* morphotypes, including 49 out of 56 turnip accessions from the present study, based on both molecular markers (AFLP, SSR) [23], [24] and total metabolites [25]. For these studies, the European turnip accessions were always grouped with broccolettos, and Asian turnip accessions were grouped either with pak choi in the study of Zhao et al. [23], [24] and Del Carpio et al. [25], or with oil accessions from the Vavilov collection and Pakistani winter oils from the WUR collection in another study using a limited number of SSR markers [24]. Together with a few studies using isozymes from turnip accessions derived from the Nordic area [26] and RAPD markers from the collection of eastern Anatolia in Turkey [48], it has been confirmed that the turnip collection displays high genetic variability and is a source of diversity in breeding programs. In this study the heterozygosity was calculated based on allele composition of the 280 SNP markers and ranged from 2.2% to 29.5%, with the inbred lines having the lowest levels of heterozygosity (Table 1).

### Environmental conditions influence turnip leaf and tuber development

There is ample variation in leaf and tuber morphology between accessions of both Asian and European subpopulations (Figure S4 and Figure S5), but accessions from the Asian subpopulation seem to form longer and narrower leaves than the accessions belonging to the European subpopulation. The heritability of turnip traits in individual experiments ranged from 0.35 to 0.86, while the calculated heritability over all experiments was very low (0.10-0.62). This does not only reflect influence of the environment on these traits, but also the different experimental conditions (field/greenhouse, harvest time, temp and day length affect turnip tuber growth). For the five leaf traits, we only analysed two experiments (2008F, 2010G); heritability is much lower in the field (0.10–0.27) (2008F) than in the greenhouse (0.28–0.64) (2010G), with more uniform conditions. (Table S7).

The PCA analysis visualized the contribution of the different traits to the phenotypic variation among the turnip accessions evaluated in five independent experiments with different sowing and harvesting dates (Table 2 and Table S2, Figure S1). Leaf index (LI) was higher indicating a wider leaf blade for European turnips compared to Asian turnips in two experiments. Moreover the leaves of both subpopulations were also much wider in experiment 2008F compared to the corresponding ones in experiment 2010G for both subpopulations (Table S8). This was apparently caused by the different growing environment as 2008F was carried out in a field and 2010G was a greenhouse experiment. The separate PCA analysis from four out of five experiments illustrated very similar clustering of turnip accessions, although different traits were measured in each experiment. Results showed that the Asian subpopulation tended to form a loose cluster, with the important explaining variables leaf lamina shape (LBL), leaf index (LI) and tuber color (TC) in experiments 2010G and 2008F, and tuber shoot number (Tsh) in four out of five experiments (except 2012F) (Figure 4 and Figure S6). Tuber size (both Twi and TL) and weight (Twe) for both the Asian and European subpopulations were higher in experiments with early sowing in May (2008F and 2009F) than in the other three experiments, with later sowing dates. This observation suggests that (i) turnip tubers develop faster under long day conditions with relatively higher temperatures, as the turnips of experiment 2011F were growing from mid-autumn towards winter, while in 2008F and 2009F they grew from late spring to autumn; (ii) growing in pots limited the size and weight expansion of the tuber, as experiment 2010G was carried out in the greenhouse, where except for growing plants in pots the temperature and day length during growth and plant age at turnip tuber harvest were all comparable with that in 2008F and 2009F. The Asian turnip tubers had similar size and weight range in experiments 2011F and 2012F that were both performed in the late autumn, while European turnip tubers were smaller in 2012F than in 2011F. This observation fits the fact that most European turnips are biennial and need a longer period to reach the mature stage and in 2012F tubers were harvested younger (75 days) than in 2011F (126 days) (Figure 5, Table S2 and Figure S1).

### Correlation between flowering time and turnip tuber formation and influence of vernalization

Flowering is an important step in plant growth and defines the agriculture setting of the crop. Vernalization is the promotion of flowering after exposure to cold, where plants do not necessarily initiate flowering but acquire the competence to do so. In *B. rapa*, many papers reported quantitaive trait loci (QTL) regions or genes that regulate flowering and vernalization [49], [50], [51], [52]. Lou et al. identified one major flowering QTL on *B. rapa* linkage group A02 that colocalized with a major turnip width QTL, using a segregating DH population from a cross between a turnip and a yellow sarson, and the BC_1_ from the same parents [53]. Our results within a turnip collection showed no correlation between flowering time (FT) and turnip size traits (Twi and Twe) in experiment 2008F and 2010G without vernalization; whereas the correlations between FT and Twi, Twe and turnip shoot number (Tsh) were significant in experiment 2009F, in which the temperature after transplanting was only 5–10°C (Figure S1), and in experiment 2010G with four weeks and eight weeks vernalization. This suggested that flowering time is not correlated with turnip tuber traits under non-vernalization conditions, but that after vernalization these traits are negatively correlated.

In a study towards the effects of vernalization and the length of the photoperiod on inflorescence formation of two Japanese turnip cultivars, the percentage of plants with a terminal inflorescence and also the number of lateral inflorescences per plant increased as the chilling duration and the subsequent photoperiods were longer [54]. In research on the flowering response to vernalization in swede (tuber forming type from *B. napus* subsp. *rapifera*), temperatures of 5−9°C for four weeks induced flowering and the date of flowering was advanced by extending the cold period [55]. Takahashi and co-authors studied the interactions between vernalization and photoperiodic effects on the flowering of 12 turnip varieties (9 Asian and 3 European) and implied that, depending on the variety, temperature and photoperiod play important roles in inducing flowering in turnip plants, and that the vernalization effect evoked at the early seedling stage can be totally or partially nullified by the subsequent short day condition. In their study, both Asian and the three European varieties were sensitive to vernalization of 30 days (∼4 weeks) under long day conditions [38]. In our study, most European accessions were not influenced by four weeks vernalization, with a few exceptions that flowered earlier after only four weeks vernalization.

Our results showed that for several morphological traits, the Asian and European subpopulations responded differently to the length of the vernalization period, under the same photoperiodic condition (long day) (Figure 6 and Figure S7). Vernalization shortened leaf length and increased leaf width in turnip accessions, such as T_1050V, FT_086, VT_007 and VT_014 (Figure S7 and Table S9). This finding is in agreement with the study on *Arabidopsis thaliana* ecotypes where extended vernalization led to shorter and more erect leaves [56]. Except for VT_007 (with admixed population structure), turnip accessions that belong to the Asian subpopulation all flowered earlier and formed smaller and lighter tubers after vernalization, and the effect of four and eight weeks vernalization was the same (Table S9). Accessions belonging to the European subpopulation were less sensitive to four weeks vernalization, as measured by flowering and tuber size, while 11 out of 21 accessions were affected in these traits after a prolonged vernalization period (eight weeks) (Figure 6 and Table S9). These findings suggest that tuber sink filling is interrupted by accelerated flowering after vernalization, and this effect of vernalization is different in Asian and European accessions.

## Conclusion

Anatomy and carbohydrate composition of turnip tubers differ among accessions, independent from their geographical origins. The evaluated morphological traits segregated in both Asian and European turnips, with leaf shape, tuber colour and number of shoots per tuber explaining the most variation between the two subpopulations. The effect of vernalization on flowering and tuber formation differed significantly between the two subpopulations.

## Supporting Information

Figure S1 **Day length and temperature profile over the experimental period in the four field experiments.** Frames indicate the period from sowing till harvest for each experiment.
(TIF)

Figure S2 **Cross section of 28 day old turnip tuber at position (a) (5 mm below cotyledons) of VT_052 (100x magnification).** The scale bar stands for 100 µm. P: Pith; C: cortex; Ph: phloem; Ca: vascular cambium; pX: primary xylem; sX: secondary xylem.
(TIF)

Figure S3 **Illustration of turnip shoots from tubers of VT_137 and VT_089 grown in the field experiment of 2008F.**
(TIF)

Figure S4 **Display of leaf and mature turnip tuber for the 15 turnip accessions from the Asian subpopulation.**
(TIF)

Figure S5 **Display of leaf and mature turnip tuber for the 41 turnip accessions from the European subpopulation.**
(TIF)

Figure S6 **Principal component analysis (PCA) diagram showing the phenotypic variation and the contribution of different traits in the tested turnip accessions of the 2009F, 2011F and 2012F experiments.** The presented traits are leaf color (LC), leaf length (LL), lamina blade length (LBL), lamina blade width (LBW), leaf index (LI), petiole length (PL), petiole width (PW), leaf lobe (LB), leaf lobelets (LBs), leaf edge shape (LES), leaf blade shape outline (LS), leaf division (LD), leaf apex shape (LAS), leaf hairiness (LH), leaf lamina attitude (LAT), leaf and stem weight (Lwe), flowering time (FT), tuber length (TL), tuber width (Twi), tuber index (TI), tuber shape (TS), tuber color (TC), tuber shoots number (Tsh), tuber weight (Twe) tuber surface smoothness (Tss), tuber swelling onset (Tso) and tuber growing depth (Tgd). Red arrows represent the contribution of different traits to the total variation. Percent of variation explained by each dimension is indicated. The name of accession with either _A or _E extension stands for Asian or European subpopulation, respectively.
(TIF)

Figure S7 **Boxplots showing vernalization responses of the 29 turnip accessions for 6 different phenotypic traits to no vernalization, four weeks vernalization and eight week vernalization.** Open and stripped boxes stand for Asian and European subpopulation respectively. Outlier was marked using asterisk. LBW: lamina blade width; PW: petiole width; LI: leaf index (lamina blade width/lamina blade length); LC: leaf color; LAT: leaf lamina attitude; Lwe: leaf and stem weight.
(TIF)

Table S1 **List of 280 SNP markers with their physical positions and 100 nucleotides flanking the SNP site.**
(PDF)

Table S2 **Experimental design and performing period for the five independent experiments.**
(PDF)

Table S3 **Comparison of stele and xylem areas in the six turnip accessions.** Numbers indicate mean ± standard deviation.
(PDF)

Table S4 **Results of genotyping 56 turnip accessions using 280 SNP markers.**
(PDF)

Table S5 **Descriptive of phenotypic value of traits that were evaluated in each experiment.** The presented traits are leaf color (LC), leaf length (LL), lamina blade length (LBL), lamina blade width (LBW), leaf index (LI), petiole length (PL), petiole width (PW), leaf lobe (LB), leaf lobelets (LBs), leaf edge shape (LES), leaf blade shape outline (LS), leaf division (LD), leaf apex shape (LAS), leaf hairiness (LH), leaf lamina attitude (LAT), leaf and stem weight (Lwe), flowering time (FT), tuber length (TL), tuber width (Twi), tuber index (TI), tuber shape (TS), tuber color (TC), tuber shoots number (Tsh), tuber weight (Twe), tuber dry weight (TDW), tuber surface smoothness (Tss), tuber swelling onset (Tso), tuber growing depth (Tgd) and tuber dry mass% (TDM). Experiment code “2008F, 2009F, 2011F and 2012F” stand for four field experiments carried out between 2008 and 2012. Code “2010G” means the greenhouse experiment in 2010.
(PDF)

Table S6 **Restults of analysis of variance (ANOVA) for each trait in five experiments.** The presented traits are leaf color (LC), leaf length (LL), lamina blade length (LBL), lamina blade width (LBW), leaf index (LI), petiole length (PL), petiole width (PW), leaf lobe (LB), leaf lobelets (LBs), leaf edge shape (LES), leaf blade shape outline (LS), leaf division (LD), leaf apex shape (LAS), leaf hairiness (LH), leaf lamina attitude (LAT), leaf and stem weight (Lwe), flowering time (FT), tuber length (TL), tuber width (Twi), tuber index (TI), tuber shape (TS), tuber color (TC), tuber shoots number (Tsh), tuber weight (Twe), tuber dry weight (TDW), tuber surface smoothness (Tss), tuber swelling onset (Tso), tuber growing depth (Tgd) and tuber dry mass% (TDM).
(PDF)

Table S7 **Heritability of eleven traits that were evaluated in multiple experiments.**
(PDF)

Table S8 **Descriptive of phenotypic variation between Asian and European subpopulations without vernalization for each trait from five independent experiments.**
(PDF)

Table S9 **Descriptive of phenotypic variation between Asian and European subpopulations with and without vernalization for each trait from five independent experiments.** Within the same trait, same accession, the values accompanied by the common letter show no significant difference between the treatments. The presented traits are flowering time (FT), tuber weight (Twe), tuber width (Twi), tuber shoots number (Tsh) tuber growing depth (Tgd), tuber swelling onset (Tso), tuber surface smoothness (Tss), tuber color (TC), leaf and stem weight (Lwe), leaf length (LL), lamina blade width (LBW), leaf index (LI), leaf color (LC), petiole width (PW), leaf blade shape outline (LS), leaf lamina attitude (LAT) and leaf edge shape (LES).
(PDF)
