# Supplementary figures and images for: Morphology, Carbohydrate Composition and Vernalization Response in a Genetically Diverse Collection of Asian and European Turnips (Brassica rapa subsp. rapa)
Source: PLoS One. 2014 Dec 4;9(12):e114241. doi: 10.1371/journal.pone.0114241 (PMC4256417; doi:10.1371/journal.pone.0114241)

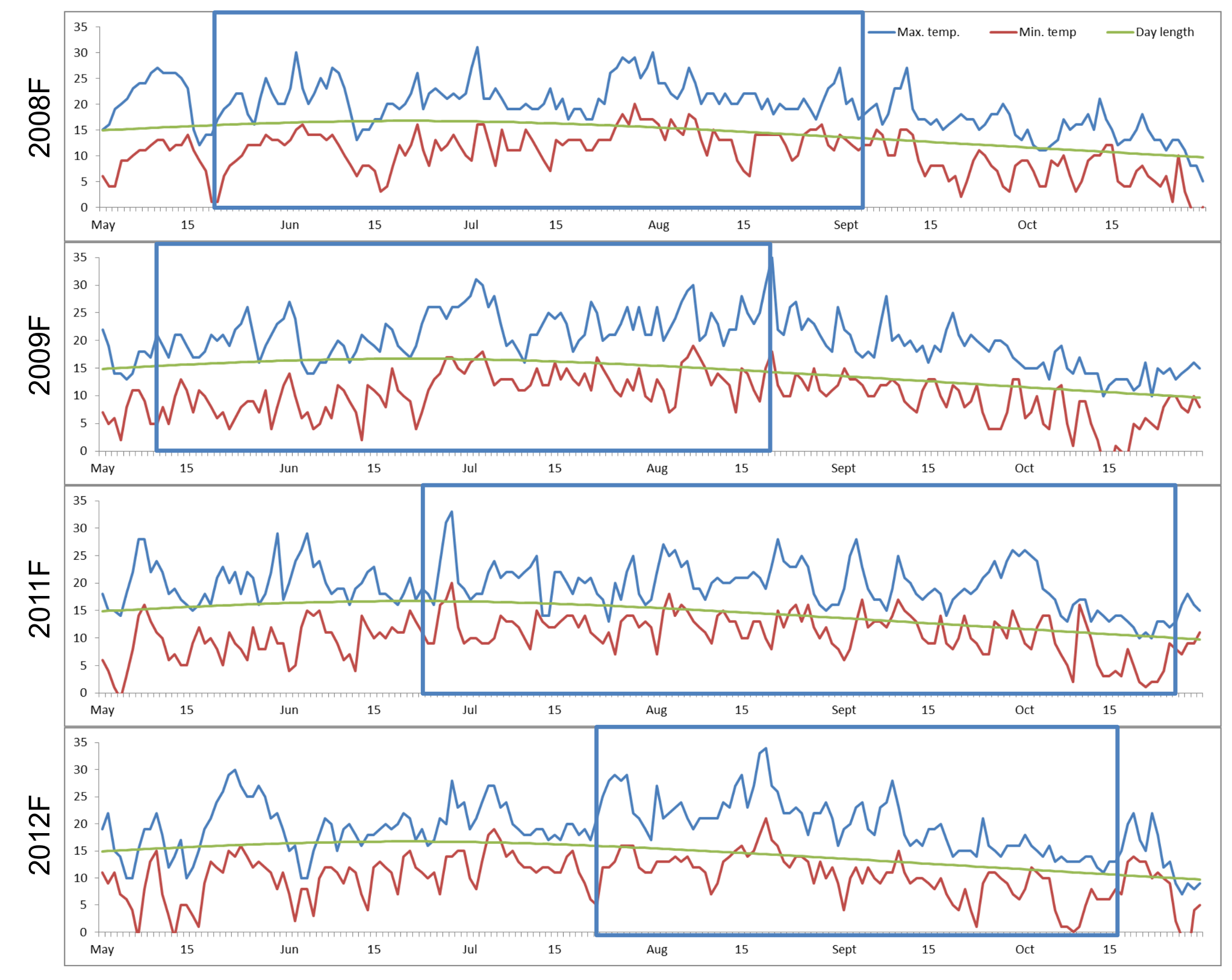

Supplement: Figure S1 — Day length and temperature profile over the experimental period in the four field experiments. Frames indicate the period from sowing till harvest for each experiment. (TIF) [file pone.0114241.s001.tif]

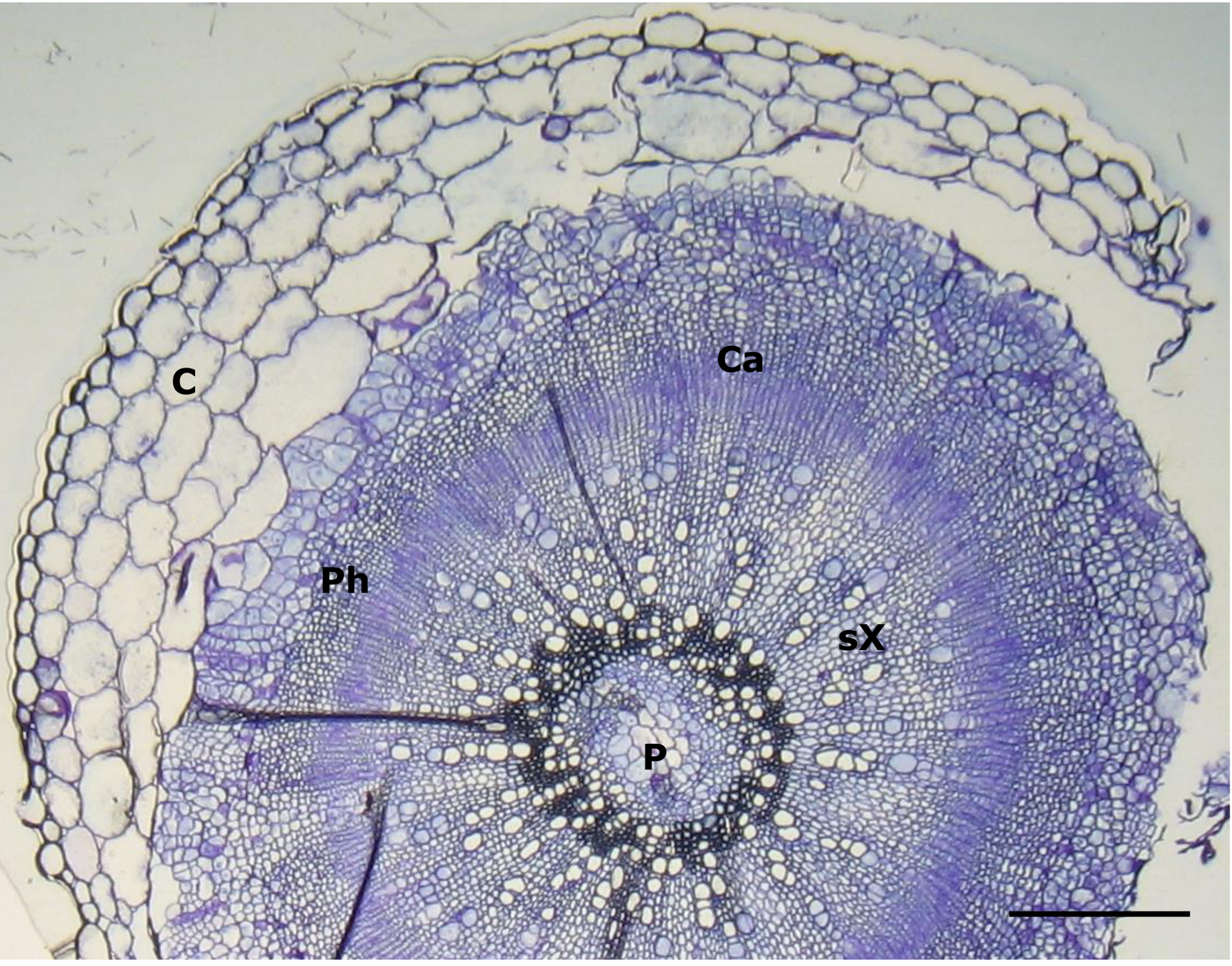

Supplement: Figure S2 — Cross section of 28 day old turnip tuber at position (a) (5 mm below cotyledons) of VT_052 (100x magnification). The scale bar stands for 100 µm. P: Pith; C: cortex; Ph: phloem; Ca: vascular cambium; pX: primary xylem; sX: secondary xylem. (TIF) [file pone.0114241.s002.tif]

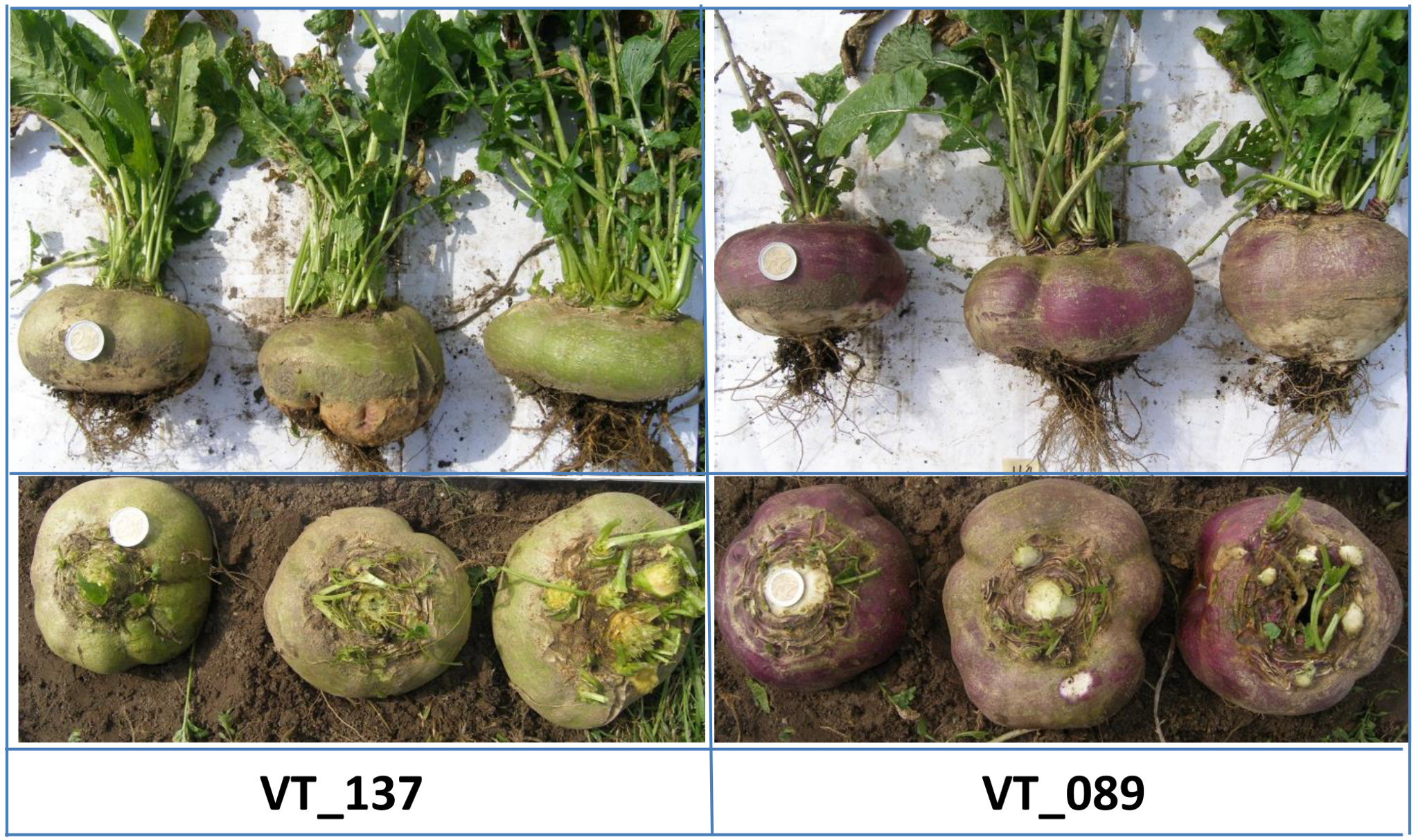

Supplement: Figure S3 — Illustration of turnip shoots from tubers of VT_137 and VT_089 grown in the field experiment of 2008F. (TIF) [file pone.0114241.s003.tif]

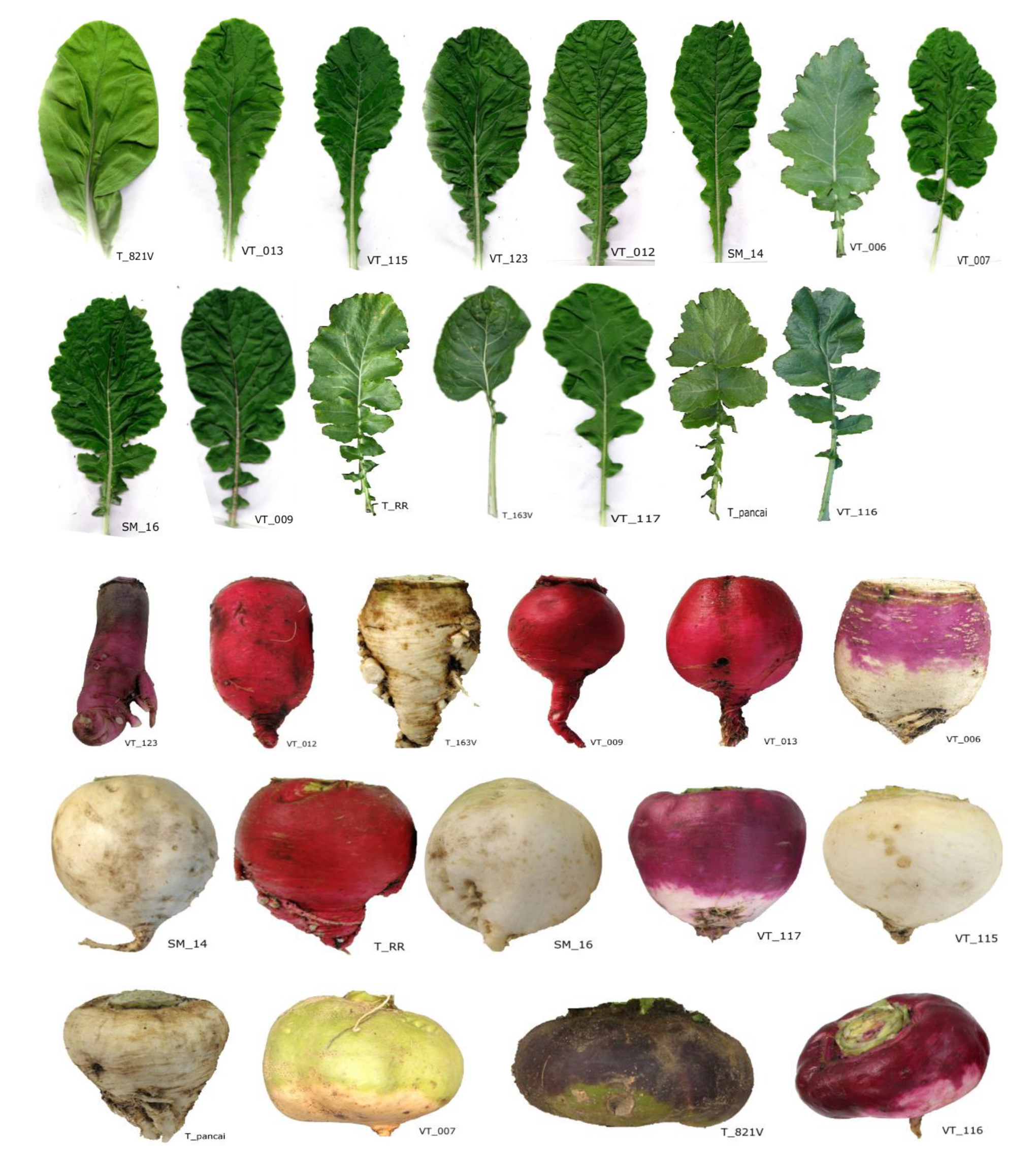

Supplement: Figure S4 — Display of leaf and mature turnip tuber for the 15 turnip accessions from the Asian subpopulation. (TIF) [file pone.0114241.s004.tif]

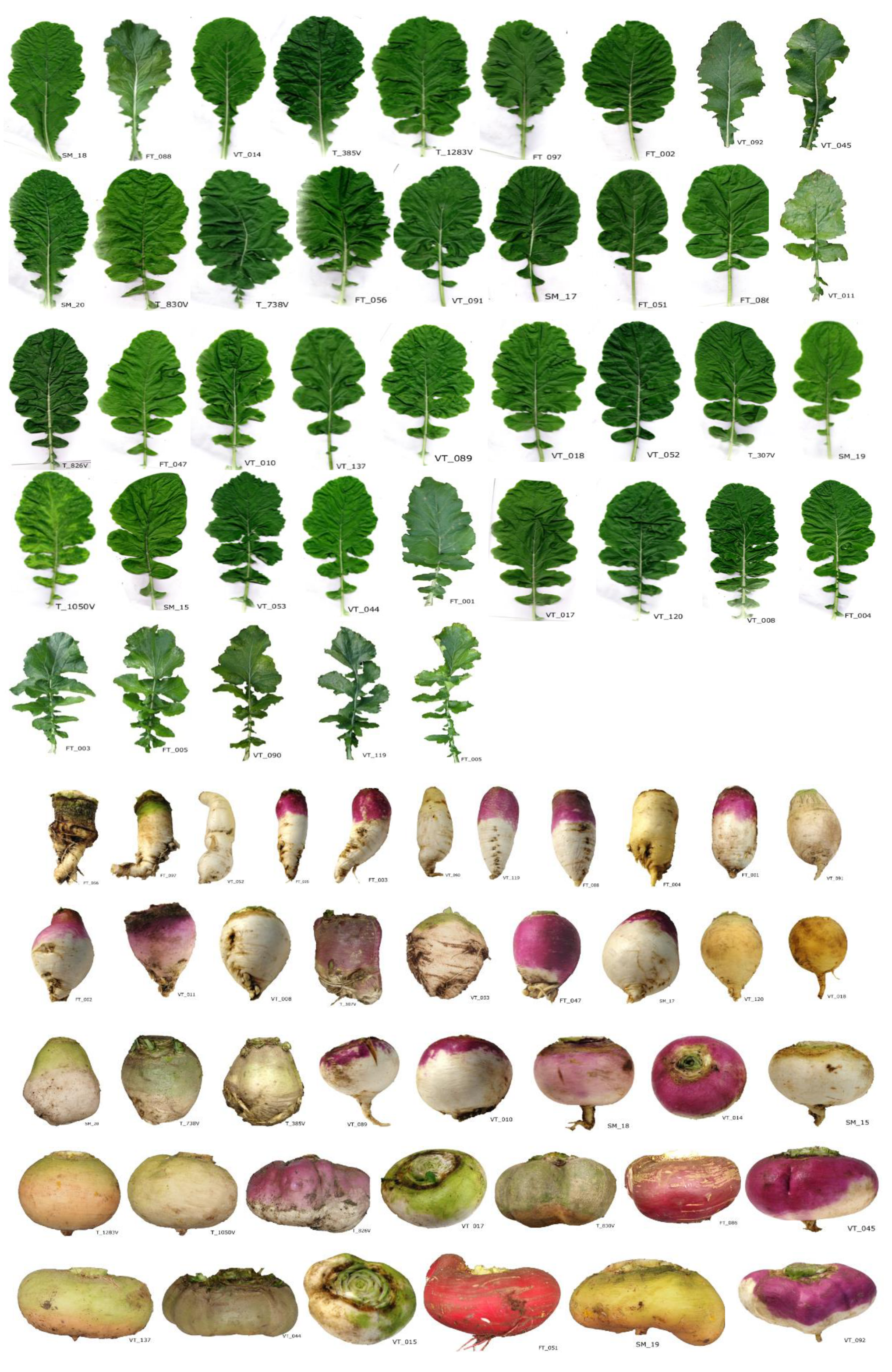

Supplement: Figure S5 — Display of leaf and mature turnip tuber for the 41 turnip accessions from the European subpopulation. (TIF) [file pone.0114241.s005.tif]

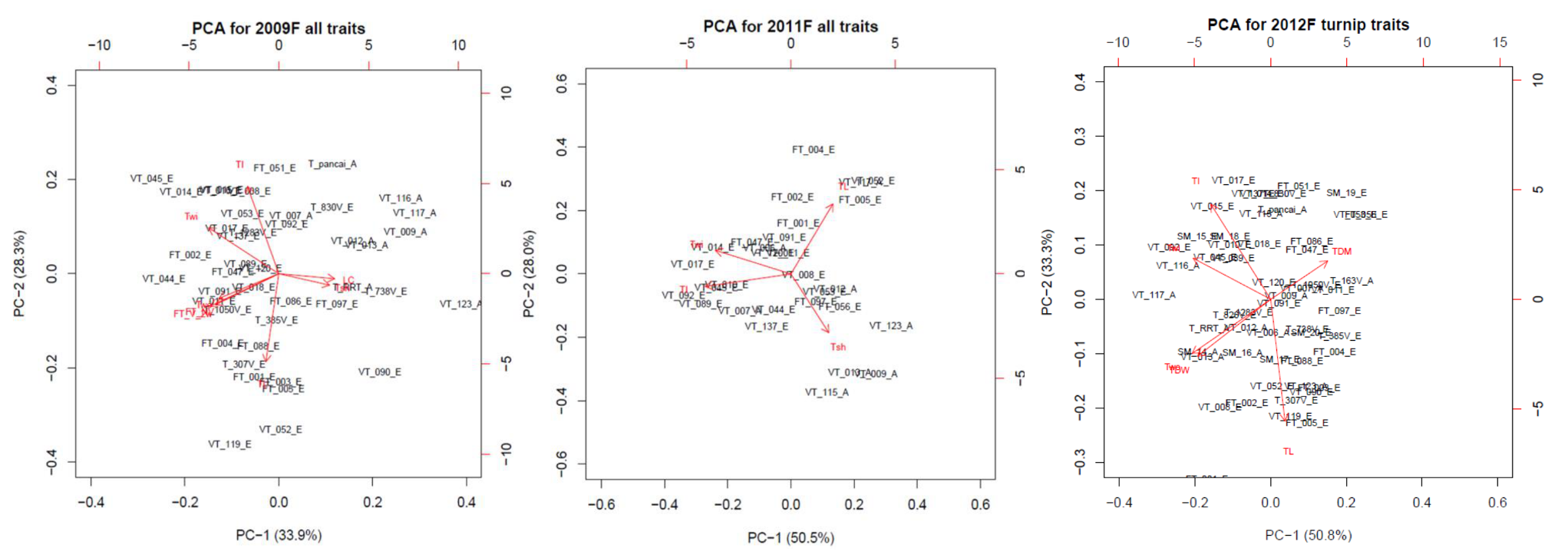

Supplement: Figure S6 — Principal component analysis (PCA) diagram showing the phenotypic variation and the contribution of different traits in the tested turnip accessions of the 2009F, 2011F and 2012F experiments. The presented traits are leaf color (LC), leaf length (LL), lamina blade length (LBL), lamina blade width (LBW), leaf index (LI), petiole length (PL), petiole width (PW), leaf lobe (LB), leaf lobelets (LBs), leaf edge shape (LES), leaf blade shape outline (LS), leaf division (LD), leaf apex shape (LAS), leaf hairiness (LH), leaf lamina attitude (LAT), leaf and stem weight (Lwe), flowering time (FT), tuber length (TL), tuber width (Twi), tuber index (TI), tuber shape (TS), tuber color (TC), tuber shoots number (Tsh), tuber weight (Twe) tuber surface smoothness (Tss), tuber swelling onset (Tso) and tuber growing depth (Tgd). Red arrows represent the contribution of different traits to the total variation. Percent of variation explained by each dimension is indicated. The name of accession with either _A or _E extension stands for Asian or European subpopulation, respectively. (TIF) [file pone.0114241.s006.tif]

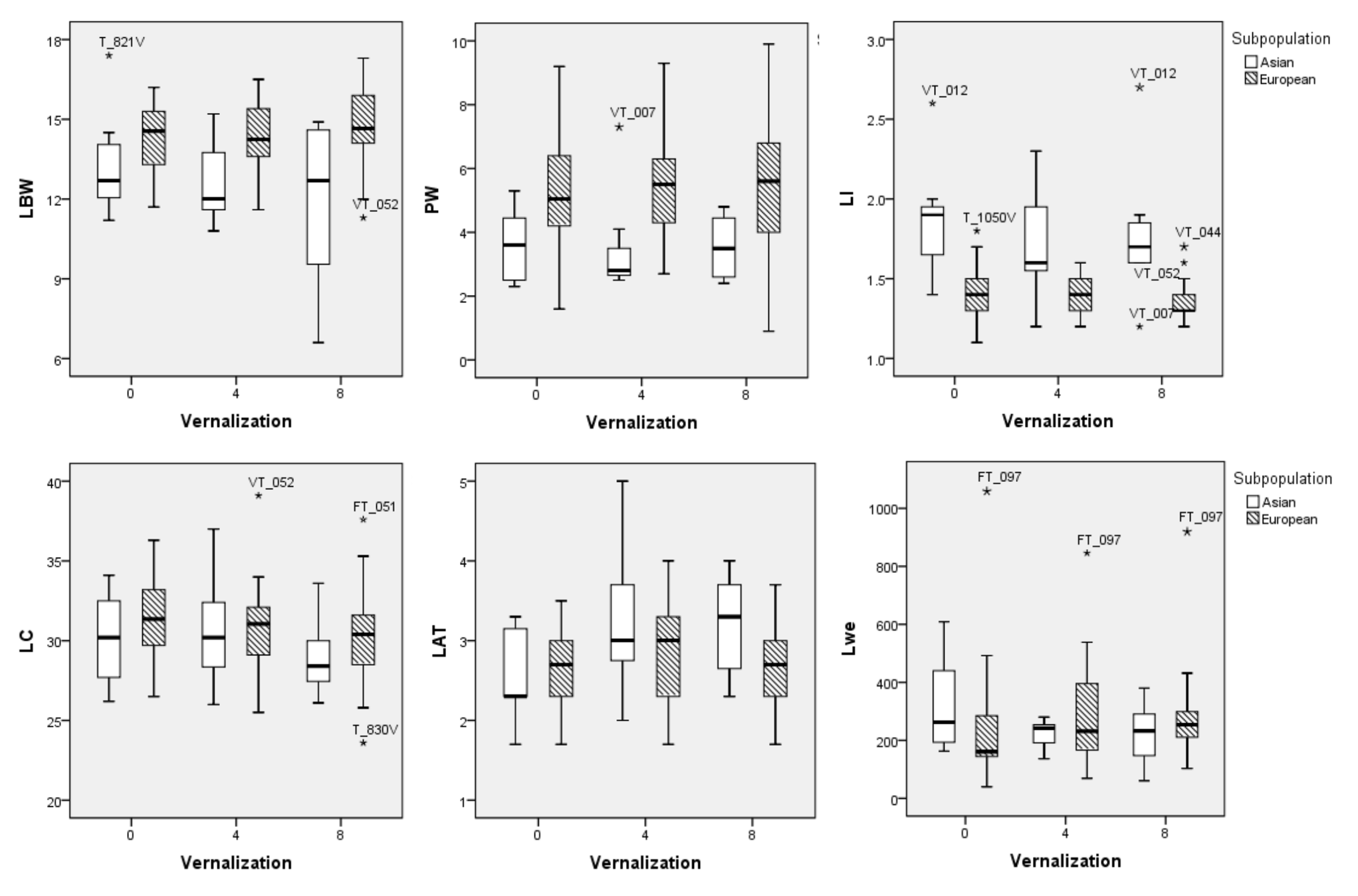

Supplement: Figure S7 — Boxplots showing vernalization responses of the 29 turnip accessions for 6 different phenotypic traits to no vernalization, four weeks vernalization and eight week vernalization. Open and stripped boxes stand for Asian and European subpopulation respectively. Outlier was marked using asterisk. LBW: lamina blade width; PW: petiole width; LI: leaf index (lamina blade width/lamina blade length); LC: leaf color; LAT: leaf lamina attitude; Lwe: leaf and stem weight. (TIF) [file pone.0114241.s007.tif]
