# Supplementary material for: Morphology, Carbohydrate Composition and Vernalization Response in a Genetically Diverse Collection of Asian and European Turnips (Brassica rapa subsp. rapa)
Source: PLoS One. 2014 Dec 4;9(12):e114241. doi: 10.1371/journal.pone.0114241 (PMC4256417; doi:10.1371/journal.pone.0114241)
Supplement: Table S1 — List of 280 SNP markers with their physical positions and 100 nucleotides flanking the SNP site. (PDF) [file pone.0114241.s008.pdf]

Table S1. List of 280 SNP markers with their physical positions and 100 nucleotides flanking the SNP site

Marker ID	Linkage	Position*	Flanking Sequence (100bp flanking the mutation site, and the SNP site is in brackts)
PV_Br_SNP_0002	A01	191796	GTGACCCAGGTTTTGTAGATAATTGCACTAGTTTTCCTTTTATCTTTGATGCAAAGTCCGTTGCTTGGGGTAGTTGTGGGGATACTTACGACCGGCACG(A/G)AGATCCGTTGTTCAGGGAATTGTTGTTTATATCTGGAAGTCATGGTGTTACCGTCCATGCTTTTTGCTGTTTCAAAGATTCAAGTGACGTAGCCAATGGG
PV_Br_SNP_0003	A01	198417	GAACAGGCACAGTAACTCTTCTGCACAGTGCACACTGGAGAATTTGAAACCATTTCTTCAGAGATTCCCTACCTTATGGAGTAAGCTTGTTACTGCTTG(T/C)TTTGGAGAAGATATTTCTGGCAACCTTCTGCGGACCAAGGCTAGGAATGGTAACCATGATCCACGATATGTTTTCGTTTATTTGTGTAATGATATTGATG
PV_Br_SNP_0005	A01	207316	GGTGGATAAAGCCTCATTGTTTCACACAACGCTGCGTGTAAATACACTAGCTTGTTAAACTGCTCCGTTGGATCACAATCACTACTATACTTGGTGGTA(G/A)ATTTGTGTCCATCTCTCGGAGCTGAACATCTCTTAGTGAGATGAGACCAGGATCTGGTACATCAATTAATTCCCTTATGTACCATACCATAATTTTGTGA
PV_Br_SNP_1048	A01	332469	CATTCACAAGTATTGTTGATGATTAGATTACCTCATGTCCTTGATGATTTGGTCGACTTGTTCCTTCTTGGTACCGTTGATGACGTTGTTGGCACGTGA(G/A)CCTTGATTAGCAGCGATGAAATCAGGGTCGTAGATCCCAGGGAGTGGGACAATGTTCTCCATGTAAGGCCTGAGCTGTTTCTGTAAGTCAATGTCAAGAA
PV_Br_SNP_0006	A01	511520	ACCATCAAGTGATTGTGCATGGTACGATTGTATAGGTTATTAGCACCGAGTGGGGAGATTTCAGATCTTGTCATCTTCCTCTAACTGAATTTGATGCTG(C/G)TCTTGATGCGGAAAGCTTGAATCCAGGAAGCCACGTAAGAACACTTATTGGTTAAACAAAGTCATGTTTTGTGTGTTTTGAGAATATTTTTGGTTATGCT
PV_Br_SNP_0014	A01	2609884	ATTCAGAGGACTTTGGAGACTGACCAGCGCGGTTCGACCGTTCTTATCAGTTTCATTAGGGTTTGAGCCTCGTTTCAGTAACTGATGCAGCAGTAAATC(A/G)TCACCTCTTGCTGCTGCGAAACATAAGCTGAGAGGTAAGTCCATTTTCCCTTGAGCCAACATCTGTTCTGTATCAGCCAAAATGCCTTTCATCACTGGAT
PV_Br_SNP_0015	A01	2867228	TATGAGCTGAATAGAGGTAGATGGAGAAGAGGACCCTTGTTCATCGATAGGGCTGGTTTCAGTTCATCTTTTTGTGTGATAGACAACGTGCTGTATCGT(T/C)GTAATAGCTGTATGATCGATTGGTATGACTCTGAGGAAAATTTGTGGGAAACGGTTAAGGGTTTGAAGGGATTGGTTCCCTTTTATTCGGGTTACATCCA
PV_Br_SNP_0016	A01	3049926	ACTACACAGGAGTGTAGGTTCAATGCAACGACTGAGGAAGTGATAAGTTACCGTCATCCTTCGGATATCCCTAGGACTCCATCTCACCAACCTCACAGT(C/A)CAATGTGCAGATAACTTTTTTTGTATGTTTTGATTTGCTTGTTGATTGCTTTGAACCATTCTTCTTCCTCTTCCCCATGTTCCAACTTTTTCACTTGGGG
PV_Br_SNP_1064	A01	3360507	AATGGAAAGTGTTAGTAGAATTAATTGTATAGTTAATATGATAATATTTTAGTAATTATATGGTCCCTAGAGTTGGGTTTAAGACCAATGTAAAGTTAG(T/A)TTGGTAAGAAGAGAGAACCTGGTGACCAAAGCAGATGCCAAGAATTTTCTTCTTCATCTCATCAAGTTTCTTGACGATCTCACACAGCCTAAGGATCCAT
PV_Br_SNP_0017	A01	4993191	TCCGTAGACTGTGTCATTAATTAGGAAAGAGTAGGAGACTGACTGGATGCATTGCATGATTAGATTAATCCATTTGTCGTGAAAACCCAACCTTTGTAG(G/A)ACCTCTGATACAAAAACCCACTCAACTCTATCATATGCCTTTGACATATCCATCTTTACGGCCATTGAGCAGTTGATCTGAGCCTGAGATGTTTTGAGAT
PV_Br_SNP_0029	A01	8038575	TTGAGCTGTGAACTGATCCCACTGGGAGAGATACCCGTTTGAGTAAGTGATGTCTCCAATATGGAAAACAATGTCAATGTTCTTCAAGTCCTTGATGAG(C/T)TGGTCTGTTGTGTTGAGGGAACCAGGCTGGTAATCATTATATTCGTTCGAGCCATCTCGCTCTCCCTGAAAACATATCTCAATGTAAGTAAGTGACCTTT
PV_Br_SNP_0033	A01	8788567	TATTCTTAATGATACAAATGCTTCTAAAGGTTGAGTCTCTATATGTTACTTTATCTCTCCAACTAAAGTTTTGTTTCTAACCAAAAATACAGAGGAACA(C/T)GCTCCCGTGAGACGAACCTTTGGCAAACCTATTTGTTGGACCCAATTTTGGTCAAGATCAAAATGGGCCCCGATCAAAGACATGGGCCAAGACACGCCGA
PV_Br_SNP_0035	A01	9304705	CGATAGCCATCTTCGAGGATGTCGATGTCGCTTGTGGTCTTCACCACGACCTTTGGCGCACGGTTGGCTCTGGTTGATCCACTCATTTCCGTTGCATAA(G/A)CTTCTAGTTTCCTGATACGTGTGAATAGCACAAGAAGTCAGAACGTTTCGGCTTCTGATCATTTTAATTTTTAATGTTTACTTTTAATTTCTAACCTTCT
PV_Br_SNP_0037	A01	9618547	AAGGATTCTCTATGTGAATGGTGGCCATTGAGAGCGTTGCTGGTTTTCTCACAATCTTTGTCAGACAAAAAACCTTTCATCTTGCATTCCAGAAGGACA(A/G)TATGTCGACTTGCCGATGCTTCTTTTGCTGATACTCTTGATGAGATCAAAGGGCTGGTGAGACGTAGTTCTCCGGATGAAATTGCTGGAATCGTTCAGTC
PV_Br_SNP_0055	A01	14045839	CCGCTCTTTCTCTTCTGTCTCAAGATCATACAACACGCCTTTGGTCTGCGGTATGTACCCTTTCTCCTTCATATCTTCTGCTAACTTAACCAAGAGAGC(G/A)TGAATCTGTTCCATTAGAGGATTGAACTCATCCACCGAGACAAACGAGTACATTTTCCTCCCCACTTCCATCCAGCACCGACCAGGCAGCTTCTGCAGTC
PV_Br_SNP_0058	A01	14246865	GTCGTCGTTGATAGATTGAGCAAAGCAGCTCACTTCATGGCGTTATCACACCCTTACAATGCTGCTTCTGTGGCTCAAGTCTTCTTGGATACAGTCTAT(T/C)GTCAACATGGATTCCCTCGTTCAATTGTGAGTGACAGAGATGCTGTATTTCTCAGTGAGTTTTGGCAAGAGTTGTTCACACTACAGGGTTGTTCTCTTAA
PV_Br_SNP_0059	A01	14336973	ACATTCTCAATATTTGATAGTTATATTGTTGAATCTGTTAGTGTTACAATCATGCTATTTGGTGTTTTGGCAGAGAAGTGGAATCCCAGTTTCTGACAT(T/C)TGTCAGCACTGGTTTCTCTTGCACAACAAGGACGCCACGCAACATCTGTTTGAAGTCTCAGCTGGTCTGGACTCTCTTGTCCTAGGGGAAGGTCAGATAC
PV_Br_SNP_0060	A01	14415476	ACGAGTGCAATACACTTCTGCTTCGTCAAACTTCTTGTTGGTCTTCAAATGTGTAGCAAGGAAAACCAAAGCCTCAAACATGTTAGGTCCTTCTAATCC(C/T)TCAGCATCCATTCTCTCTAAATCCTTCTCGAAGTAAAATGCAGCTTCTTCATTACGTCCAAGCTTCTGGTGAAGCTTCGCCAGCTGGTTAAGAGCTATTC
PV_Br_SNP_1055	A01	15365144	TGTCTATGTCTCTCCTGTTGGAAACTACTTCAAAGAAGGTGTGTCACCAATCAATTTGATTGTGGAAAGTGAATTTCACCGTGCAACACCTGGTGGTAC(T/C)GGAGGTGTTAAAACCATTGGCAATTATGCTGCAGTAAGTCAAAGTTGAAGTCGCTAACATCTCTTTGGACTGGCTTATGTACTCACTCACGCATAACAAC
PV_Br_SNP_0080	A01	16957132	TTGCATATGTAAGGAATCTTCTTAGCACCAGGGCCAATGGCGTCTCTATCGAGAGGTCCGTGACAGACAAAGCCAGCCTTGTTCACATGCCGTCTCTCG(T/G)TGTAAACGAGAAGCGCAAGGGAAGGACGAGCGTAGAGACGCTTCACCACGAAATGACACGTGCATCCTCTCATGGACTGAGGTCTCGCGGCTCGGTTCCT
PV_Br_SNP_0084	A01	18014692	TTCCCGAGCTATGAGAAGCCTTGTTTTGGTCGTAAAAACTAACCAAGCTTCTGACTAAACCTCTCGACCACGCCTATGGCGTATCCATGTTGATAATTC(G/A)TCTGAATCTTGACTATGCTTGTCCATTAGACGTATGTCTAGTCCTATGCTGGACTGATGGCGTTACAAATGCAGCCACCAATGCATGGAGACATACCTAG
PV_Br_SNP_0086	A01	18706064	CACAAATTCAGGGTACTTTATTGCTTCCCTCTCCCCGTCTGATATAACACAAACAAGATCAAATACATATTTTCACTGGATCAAAGAAGTGTGGGCAGG(G/A)AATTTCTCACCAAAAATGAGGACGTTCTTATGGGCTGTCTTAAAAGACTCGCTTCCTCTGGGAGCTAACCTACACAGTAGAGGCGGAATCTCAGCATCAT
PV_Br_SNP_0089	A01	19150724	AAGAGAGGGAGACCGAGGAAGTATAACCCCGACGGTTCACTCGCTGTCACACTTTCTCCGATGCCCATCTCCTCCTCCGTTCCTTTGACGACGGAGTTT(T/G)CTTCTCGGAAACGGGGACGAGGGCGCGGAAGAGGAAGAGGACGAGGAAGAGGTCGAGGACTTGTAGAGCCGCCCATCAACAACAACAATTGGGTCAAGAA
PV_Br_SNP_0098	A01	21243792	TGTCTAAGGCATGAGTCAGAAACGCACTGGTCTGGTGAAGATTGGGATGATGATTTCATCACAGAACATGAAGAAGAACAAGATCATCATCATCATCAT(T/A)GCTCCAAGACGACATCCATTAAGGCTAGTAAAACAATAATTGTCACGCAAGACAGTAACTCATCTCATCATGAGATCAAGATCAGGCTCACGAAGAAGCA
PV_Br_SNP_0101	A01	22000107	CACGGAGGTGATCGAATCAATCAGCACCGAGTCAGATCCTTCCTTGATCGAAGCGAGTTTATCATTCCTAATCACGATCGCGAAATCGAAACGAGTGAG(G/A)TCGAAACTAATCACCGCGAAAACTATCACGAAGATCAAAGACATTCTCCTAACGGAGGAGATAACCAGCGTCGCGGTGACGGAGAAATCGCTGAAACTGT
PV_Br_SNP_0104	A01	23766969	AGGCCTTGAATGGGATTTCAAAACCGTGTTTCCTAAACTCAAACCAGTTGAGTATCTAGAGTTGCCCTGGACACCTTGGCCTAACAGAACATGAATTTT(T/C)TGTTTCTTCTGTACTGAATGTAACTATATACTGTATTTAGAGTTCATAATCAGTATGTCACATACAATTTCTTGCAGAGAGATCTATATATACGTTTTAT
PV_Br_SNP_0108	A01	25004591	TAATGCAACGGTAGAGAACATTCATCTCCGAGGATAAGATGTCGTGATCAATCTTGTCTCCATGTTTCTCAATCAGCTTCTTCTGCTCATCCAAGGCAG(C/T)GGACGAGTACTCTTTGTATTTCATCAGATAAGCACCGGTCCACGTGGAAGTTATGGAGCTCGTCTGCTGTCCTGCAAAGAGAGCAGCGATGAGCAAACCC
PV_Br_SNP_1029	A01	25145190	CCCTGCGGCTACTCTATGAACTCCATCGAAGGAGATGCAATCTCCACTATCCATGTGACCCCTGAGGATGGGTTTAGCTACGCGAGCTTCGAAGCTGTG(T/G)GTTATGACTTCAACACTATTGACCTGAGCCAGCTGGTGACAAGGGTTCTGTCTTGCTTTGAGCCCAAACAATTCTCTGTGGCTGTGCACTCGAGCGTTGG
PV_Br_SNP_1030	A01	25145360	ATTCTCTGTGGCTGTGCACTCGAGCGTTGGAGTGAACGCATACAAGCCAGAGATAAGTGTAGACTTGGAAGATTACGGGTGCAGAGAGAGGACATTCGA(G/A)TCTCTGGGAGAAGAGAGCGGAACGGTGATGTATCAGACGTTTGAGAAGCTAGGCAAGTACTGTGGGTCGCCTAGATCTACATTGAAGTGTGAGTGGAGCA
PV_Br_SNP_0118	A01	28347266	TGATTAACCAGGCCCATGGTTTAGCACTTGATATCATTCCGGCCCTAGCAGTTAGCGGTGTCTTGGTAAGCATCTATGAACTCAGGTTTTGGTTGTTAA(C/G)CTTTGGATTGGTCCGGTTTACAGATCGGTTAACTAGGAATTCCATTTTCAGGTCTATATCGGTTCATTTTCGATTGGAATGGGTGCAATCCCATGGGTTA
PV_Br_SNP_0125	A02	488097	TCACAAGAACACCGTCTCCACAAACACCATGATCTCCGGCTACGTCAAGTCCGGTGACGTTTCCTCCGCTAGAGATCTCTTTGACGCCATGGTGGATCG(A/C)ACCGTGGTCACTTGGACTATCTTGATGGGGCTGTACGCTAGGAACAACCGTTTCGACGAAGCCTTCGAGCTTTTCAGACAGATGTGCAGGTCTTGTACCT
PV_Br_SNP_0128	A02	939293	ACCCAACTGTAAAGATTTCGACTGTTTCTTGGAGTTGATCCTTTGTAAGATTGCTTTCTGTGGAACGTCAGCTTTTGGGCTTTCAAGCCCACCTGAGAG(G/C)GTTCTCTGGTAACTACTCCGTTTGATCTCCTCAAGTGTTCCGTTGCCGTTTTCATCCGTTTCAGCCTCTGCGTGGGATGGCGTTGGCTCCTCTTTCTTTA
PV_Br_SNP_0130	A02	3230130	CATTTTTGATTTAGCGTGATCAAACATTAACCAGTAAGAGAATAAGAAACATATTGAGATGTAACTACAGATGCTTGAATAGGTTTTGGTACAAGAACA(G/T)ATTAGGCTAAAAGCTGTGGAGTTGTGTAAGCATGTGGACAAGCTCATCACTTGAAGGCCGGTCTGCAGGAAAGTCAGAAAGGCAGACGATGGTGATCCGT
PV_Br_SNP_0131	A02	3783084	CTTCCTCACAGGGGATGGTGACTCATCTTCGTCAAAGGCTACATCAAGAACTGAAACGGGGCTTGGCTGCTCCACCGTAACTTTCAGAGGTTTTGGCAA(C/T)GACCTCATTCCCAATTCTGGACTCTGCATTGATAAAACAGATGAAACATGTATTAGTGAATAATTTGAGAAGTGAATTTACGTTAGGGAGAGTAAAGTCT
PV_Br_SNP_1035	A02	5879777	CTTGACAGCAACATTCTTGACATTGAATCCGACATTGTCACCTGGAAGCGCTTCCACAAGAGACTCGTGGTGCATCTCAACGGACTTGACCTCAGTGGT(T/C)AACCCTGAAGGAGCGAATGTCACAACCATACCGGGCTTGAGCATACCAGTCTCCACACGACCCACTGGCACCGTTCCAATACCACCGATCTTGTAGACAT
PV_Br_SNP_1036	A02	5880587	AGCCTTAAGTTTGTCCAACACCCACGCGTACTTGAAGGACCTCTTGTTCATCTCAGCAGCCTCCTTCTCGAACCTATCGATGACACGCTTGTCAATACC(T/A)CCAAGCTTGTAGATCAAGTGACCAGTTGTAGTCGATTTGCCAGAGTCGACATGGCCAATGACAACAATGTTGATGTGAAACTTCTCTTTACCCATAGCTG
PV_Br_SNP_0143	A02	6823179	CGAAGAGATGCTTATAAGGGCAAGCAAGAAGGGAACAAAAGATCCAAATCAAGAACAGCAACCGTGAACGAGAAACCTGAGACCAATGTAGCAAATGGA(T/G)TTGGAGGAGATAATGACTTGGTTCACGTCGTCAAGACCATCAGATGCTTAGAGCAAGAAGGGCATATAGACAAGAGTTTCCGGAAAAGGTTCTTGACATG
PV_Br_SNP_0144	A02	6983274	TCTTGTCGCGGCAACTGCGGTTAACTTTACCTCCATAGTTCCCATGATCAGCTTAAGGTCTGGAATCCAGAAACGGTCCACGATCGCAATGAAAATGTT(G/C)CGTTGAACAGCATTCATAGTGTCGACTAGATTAGCTGGTCCGTGCTTCACCAAGAAGAGGGATATGAATATTACTAGACATTTCTGGAGCTTGACAGACT
PV_Br_SNP_0145	A02	7322569	CCGATGAGTTTTGACAAGGCAACAACACCTTTGAGTTTTAGAAACAATGATCATGAGTCCTGTTGTTGCCAAAGAAAAGAGAGAGTCTCCTTGAATCAT(G/C)AAGCGTCTCATCTACTGCAGCGAAGAGCTGCTTCTTCTTCATCAATAGCCATGAGCTTAACCAAGTCTCCCACACGTTTGGATCCAAATCATCCGTTTGA
PV_Br_SNP_0148	A02	7567322	CCGTCATAGGCACAAACACAGCAAATCTTTAGACAGGCATGAGCTGTATGATTCTTCCGACACTGATCGTGAGCACCGATACAGATCTAGTAAGCATAA(C/A)AAAGATGCTGATTATTCTAAGGACAAGCGTAGCTATCGTCACAAATCCAGGAGGCATCATGATAAGCATATTGATTCATCTGATGATGAGCATCGCAGAC
PV_Br_SNP_0156	A02	8448254	TGAGGATTCTCTTTTTACACCATAAGTTTGATAATATGCCCAACTCCTCGAAATTAGAGAAAATAAACCTGTACAACAACATCATTGGTGGAAACACAT(C/G)GCGGCATCTCTTCCCTTGGTAGGATCTGTACGCTAGCTCCAGTTACTCGTGATATCTCAGACACTGATCCTGCTTTCCCTTCCAAACATACTGAGTCTCT
PV_Br_SNP_0157	A02	8484688	TTATGTATATTTACTTTATGTACATAATGTATTTTGCATGTCCAAACGTAAGTATATGTATATTGGAATCTATCAAAATGATATCACTTCTTCACGTAT(T/A)CAATTTAGGTCAAACAGCATCCATTATTATAAGACTAATGTGGAGTCTGAAACTGGGTTTGTATTAGATTTGTGTGTGTGTTTTGAGTTGGCACATTGCT
PV_Br_SNP_0158	A02	8828680	CAAGTTTCGAATCCACATTTGGCTTATGCAGCCCTTGGATACTCTAGTTGAGGGACACAACACGATTCGACTGAAGGGGCAATACTGCTGACATTAACC(G/A)AGGAAACCCGACTGAAGATGATGATGATTCCAACATGGACAAATACACGGATATGCAGGAAGCAGACACTCTGCAAAGAAATCAAGAAACGACTTACATG
PV_Br_SNP_0162	A02	9343146	AAAAGGTAATCAAGACTGTTGGAATAAATAATTCAGGAAGATATTACTTATGCTTTCGAGCAGGACACCGGCTTTCATATTTCTGCCTAGAGAAACTCC(G/A)GCTCAAACCCGGCTCCATCTCTGCCTCCATGTAAAATAGAAGATTTCTACTAAAAGTGATTTTTTCATTTTTAGAATATGGAAGTCTTTAACCTAATTTC
PV_Br_SNP_0172	A02	13543301	TGATTGAGCTTGTGGACCAAAAAACAAACAAAATAAAGAATAATAAATGTCTGGAAAAGCGGGCCAATCATCATCACTCACTCATTAACAGACCCAACC(G/A)TTTTGTTCCCGCGCAATACTAATTTCGGTTTGATTCGGTTTAGGTACCAATCAACCAGCAAATCGGAGCAATCGATTGGCTCCAAGCGCAGAACGAGACT
PV_Br_SNP_0175	A02	14260292	TGTTGTTGTTGCAGGCTTTGATGGAGTTGAGATTCATGGAGCTAATGGCTATCTGATAGACCAGTTCATGAAAGACACGGTGAATGACAGAACAGATGA(G/A)TACGGTGGATCATTACAAAACCGTTGCAAGTTCGCACTAGACATAGTTGAAGCAGTGGCTAATGAGATCGGACCAGACCGTGTTGGCATTAGACTCTCTC
PV_Br_SNP_0177	A02	14947720	ATCATTCTTGCGCAGATCAGCTCAGCTTCAGCGATCCCGCTCGCTGCTATTCTCTTGTTGTTTTTACCTGACGATCCATCTACTACTGCGATCCATGGT(C/T)TGGTCTTAGTGCTATTGGGTCTGTTTGTTTCTTGGAACGCTCCTGCTACCAACAAGTGAAGTCTAAAAACACTCTTTCTCACTACTAGTACGCTTTGATA
PV_Br_SNP_0182	A02	16556107	TTGTCTCACGGAGGAAGAGATCAAACCACGAACCGTCTCAGGGGCAAAGTAGTAAACTTTGAATCTCAGTTCTTGAAAAGTCGATCTCACCAGCGACTC(T/C)AGGTTCGTCTCTGAGACGATGAAGTGGAAGAAGACGTTCTCGGGGCACAGCGAGTGCTGGAGGATGGAATTGACGGCTGCGATTGAGCCGCGTAGGTATA
PV_Br_SNP_0193	A02	21006740	TGCCGAGCGAAGGGTATCTCTCTCTCTCTCTTCCGTAACATAACTCATAAAATCTCTCTACTTTCCTTAACGTATCCGTTTCTGAGCGTTACCGATT(C/A)TCCGATCTATTTAGTACGATTGGTTTGTAGAAGTACTGTGCGATTGGTTTATCAATTTTAGGCATCAGATTTGATAGATAATGCGACTCTTTCCGTGTAG
PV_Br_SNP_0194	A02	21572911	TCACTAAAAACTGCAGCACCAAAGAGCTGAAGGAGCCCAATCTCCCGCAACAAAAGCATTCTTTTCTTGCTATTACTACGCAACGCTTCTAGAATAACA(A/G)ACTCATAGATCGTTGTCTTCGGCTTTGTCCACACAGAATCATCAACATCCTGTAACGCCACACTAGCCTCTCTGATCTTATCAACCGCTACAATCAGCAA
PV_Br_SNP_0196	A02	22448961	TCCACGAAATCTTCAACTCCGTCTTTGTTTTCCTGTTTATGCAAATCAAACATTTGTTCATAGAACGCATCAAGATCTCTCACGCTTCTCTCTCTCCGC(C/T)CTTGTAGTCCAGTGAGCCGGTCCACGATCCAGCCGCCACTTGGGAAAAAATCTGAGGCAGAGAAGCTCCCCAGGAACAGAAAAGCCTCACGGAACAACTT
PV_Br_SNP_0197	A02	22480276	TTCTCTTGTGTAAACAGGGCTGTGATGCATCAATCTTGTTGGATGATAGTGCTACCATAAAAAGTGAAAGGAATGCAGGACCAAACAAGAACTCTATTA(A/G)GGGGTTTGAAGTAATAGACGAGATCAAAGCCAAACTAGAGCAAGTTTGCCCTCAAATAGTCTCTTGCGCTGACATTCTAGCTCTTGCCGCTCGTGGCTCA
PV_Br_SNP_0206	A02	23925152	TGGCTGGTTGAAGCAGGAAGTGTGCTTGTTTTATTGTTTACCTTCACCTTCAGAACACCGTTTCTCCCCTGCACCCTTATCGCCTCATCTGAATCAGAT(T/C)CACTACTCAAACTCATACTACTCCCGCCATTCAAGTACGGTTTTTCCCTCCTAACAAACACACCCTCTCTCCCTGATCCAGACCCTCCCACAACACCATT
PV_Br_SNP_0207	A02	24175586	AAGGAGGACACTATTAGCTAACGCAGGTCTTCTGTTCATCAGCTCCTCCAAGCGAGCCAACCTCAGATCAACATCGTTATCATCGTTCAGCCAAAACCC(G/A)TTCAGAATCTTCCTCTGCAGCTCCTTCACGGATAAAGAAAAGTTAAGCCTCACATCCTCCTCCTCCTCCTCCTCCACATCCTCATCTTCTTCCTCATCAC
PV_Br_SNP_0211	A02	25024347	TTACAAAACCGCAACCATTGTACTAGCCAGTGTACTAACATCAGTAGCTGCCGCTGCATTTCTTGTTGGTTCGTGTTGTTGTTACTTTTCATCTCGGCG(C/T)AGAACAAGAGCACAAAAAGGTTTGAGATTGATATTTGTAGAAGCTGTTTTCACTATACAGAGTTTGTTTATTTTTGTACATTTTTTTGGTTTGTTTACTG
PV_Br_SNP_0213	A02	25488133	CCGGGAAAGCTGGAGGTGTGAGCATCGATGTGGAGATTACTCTCGAAGCACGTGTCATGGAGAAGCTAGAGAAGAGCAAAGAGTTTTTACTCATCGATG(T/A)GGAAGAAGATGGAAACAAGATCACCAATGGCAATGGTCATGGTCATGGTCATGGTCATGGTCATGCTAATGGTAACGGGTTTGCTTGAGCCAGTTTCGTT
PV_Br_SNP_0221	A02	27606980	GCCTTCTCTTCAAAATCTAATGCAATGCCTTCACGTGAAAGACACGTTATCGAGCATCGTCTCTAACCAGTGTAAACCCTTTAGGGCTTCAATGTATCG(G/C)CTCTGGGCGTGCATGCTTGCTCTCTCTCTTATCATGAAGGTTACGGTTCTGCTCTTCCTCGCTAGAGCTTACCAAGAGAGAGGAAAGAGCTTTGCTTGGT

Marker ID	Linkage	Position*	Flanking Sequence (100bp flanking the mutation site, and the SNP site is in brackts)
PV_Br_SNP_0222	A03	390404	ATACTCCATCTATACGTATTAATTAGTTTTTTTTTCACACAATAGACTGATTAGCGAGTAATAATACTTAAATTCAATATCAACAATACCAAACGTTT(T/C)TCATCTCATACATAATTGATCAAATGTAGTACCTGGAGAACAACGTGCCACAGTCGCACTTATACTCACGAGTGCCACAAGTTTTCGCGTGCGCCTTCCA
PV_Br_SNP_0226	A03	1591560	TTTTGGTTCTTCAAGTGCAAATGCAGGAATAACTATGGCAGATCAATCTACCTCTTGTGCAGCTATGAAGGTGAATTAAGTCTGAATTTGGATGTGTAT(C/A)CGTGTAGTACGCCAAATATATGGTGTATGATTAAACAGTTTTTGTTATTCAGAAAAATGAAGTGCGAACTCTGCCTCTTGGGAAGCCCAGAACCTCTAAT
PV_Br_SNP_0229	A03	3159821	GAGCAGTTGGGTTCAGATGTTCCTGCTTTCACCCAGGCTGCTGATGCTATGGTTAGCTGGATCCAGCTCTGGACTTATGTTAACTCCAGCGGTTAACGG(T/C)TGAACCAATGAATCTATCTCCCAAGATCAGGCATTTATCATCATGTTTATTTTCTCTTTTAATAATAATGTTAAAAACCTTTCGACTGATGAAACCCATT
PV_Br_SNP_0231	A03	3543566	TAAACTGTTAAGCATCGATTATGAGATGTTGTTTACCTGCACGAAGAGCTCAAGTTCATTCCCGAGAGGAAGAAGATCCAAATTCCGATCAAAGCAGTA(T/G)ACATGAACACGGCTACCAGAGCTTCCAGCATCAAAAATCACAGCGTAGTTCTTCGGAGGCCTCGAGTTGGGGCCTCCCTTGCGGTTAAGCACCGTGTACT
PV_Br_SNP_1031	A03	4509906	ATACCCTCTCTTAAGATCCTTGACAGCAACATTCTTAACATTGAACCCAACGTTGTCACCTGGAAGCGCCTCCACAAGAGACTCGTGGTGCATCTCAAC(G/A)GACTTAACCTCAGTGGTCAACCCTGAAGGAGCGAAGGTCACAACCATACCGGGCTTGAGCATACCGGTCTCAACACGTCCCACTGGCACCGTTCCAATAC
PV_Br_SNP_1032	A03	4510458	CTTCACCTTGTTACAGCAGCAAATCATCTGCTTGACACCAAGGGTGAAAGCAAGAAGAGCATGCTCACGGGTCTGACCATCCTTGGAGATACCAGCCTC(G/A)AAACCACCAGTGGTGGAGTCAATGATCAAAACAGCACAATCAGCCTGGGAGGTACCAGTAATCATGTTCTTGATGAAATCACGATGTCCAGGAGCGTCAA
PV_Br_SNP_0242	A03	7648839	AGTTAAAAAAAAAACATAGATGATGAAACCAGTTCCAAGGCTTTGGGTTGTCATCCCGGTTACGTTTGTGTTCTGTCTCTTCGTTCTGTTTCAACTCCA(G/A)ATATCAAACTTGTTCGGAAAAAATCTCCAAATCACTCATCAAGTAAACAACTTCTTCATCTCCATCGCCTCTTCTTCACATCATCAAACCCCAAGCCCCA
PV_Br_SNP_0243	A03	7742095	TTGACCACTCTACTACTGCAGACTATGATGAAAACGAACCTCACACACCGCCTCTGACCGGACCATACAAGAGAAGAAGAGTAACAAAGGCAATCAGCA(C/G)CGGAGTTGAATTTGACTATATGGAGGAAACACCCAAAAGAAAAGATAACCACCCCTTCGACCAGCCTGATGGTGAGAACACCATTGAAGCAACTCAGTAC
PV_Br_SNP_0245	A03	8150031	CCTGTAAGTTCCAGAATCTGTAGATTCCTCAACGCGCCAAGCGACTCAGGCAATGTCCCCTCTAAACGGTCGAACATTAGACTGAGAGAGAGCAAGCTG(C/A)TATTTCCTTTGTTCTTGGACAAGGCGTCAAGAAACCTGTGGATTTGACCTTCGAAAGAGTTTTCAGAAAGGTCAAGATGCTTCAGCTGAGGAAGGTCTCC
PV_Br_SNP_0246	A03	8251793	TTGGCGTCTCACTTTTGAAACTTAAGGTTTGAAAAAGTTGCACCTTTGTTAATTAAACTGCAGTATCTAGGTTATATTCATTCATGTCTAATAAGATTT(T/C)TCTGTCCATTTGACAGGACGTAATAGATTTTTGTAAAGATGTTGGACTGCAATCTAGTGCAGCTGCTACCAGAAATGCTACAATCAAACTTTTGGGTGTT
PV_Br_SNP_0253	A03	10871276	CTTCAAAGAAAGCACCTGCTTCAGACAACATCATCCCCTGTTCGCAGAATCGACTAATGATGTTCCTGTAACAAAGCTGTGAGCCATTGATGTTCGTTC(C/T)GACTTGCTGAAAAGTATCGACTGCCTCTCTGCATTAAACTGATTGTCCAGCATCCAATCAAACAATGCCCACGCTTCTCTTTTCTTGTCGTAATTAAGAA
PV_Br_SNP_0255	A03	11342547	CTCCCTGGCTATCTTTTCAGGACCCTAAGCCACCATTAAGTATATTCTCATTCCTTCCAGAACCTCTTTTGCAATCTTTGGTCTTCCTAAGACATGATC(T/C)AATCCTAGATGATTCTCTGAAATGACCCCGTAGAGCGGATCGGTTTCATTTAAACTTTCTGTCACATCCGATCTTTTACTTGTTGTAGATTATACTGATA
PV_Br_SNP_0264	A03	14770684	GGAGTGAGATAGTCTGATTCCTACTACTGCTTACACATTGATGATCCAACATCTGACAACACTGTTTTGAATGTTGAAATAGACAGTGCAAGGGGCTCT(T/C)CTTACCTGTTCATATGCGGTTCTCGACTACGCCCAAACCGGTTTGATTGCAGCCGTTTTTATCTTTAAAGTAAGTACCCATACACTTCACCGTTTATGAT
PV_Br_SNP_0267	A03	15435114	AAAAGGCTGAACGTCTCCTCGCGTACCGACGATTAGCATCACAATCTGCATAGGAGGAACGTACTGAAAGTCTACTCCATCGATAGGCTCATCACCGTT(T/C)TTGGAATCTTCTTCACGGTCTAAAGGAATAGATTGTGGGATGGAATCAGCTGGAACTTCGAACACGACTGTGCCATCAGGTTTCACATTAGCTATACGGT
PV_Br_SNP_0274	A03	17345774	ACTTCAACCGCCGATGACTGATAAAAGTGAAAACAAAATTCATTTTATCATTATGAACAATTTTGTGGTCGGAGTGTAACATATCTGTTTATGTTCTAA(T/C)CAAATCTAACTTCACAGTAAGTACCTTATCATCTGTATTTTTCTCAGAACCTCCATTGTTGTAATGCTGATCGATTATGATCGGGTTTTCCACGTTATCT
PV_Br_SNP_1050	A03	18284950	ATGGCATACAAAGTCTGTGGGCTCCTGATTGAAGACGAAGGGCGTTTTGGTCTGGTCGGAATAGCATAATCCGGCCATCTTCTGTCCTGTAAGCCTTCA(G/A)CCCTTCATATAAACCCTATTCATTCCCAAACCACAAATATTTCAGATTATTTTCCTTGCGCATCACTCAATTCATCATCATCTTTTTCATTTTACATACA
PV_Br_SNP_0284	A03	19709518	ACCATCATGAATCTACTATCTATCCACCTTTCCCCGTTTATCTTCCTCTGCCCTCATCGAATAAGCAAACTCAACCTGCCGAGTTTACAATCTACTTTG(G/T)CTGGACCAAGTTATACACCATCTCAGCCAAATCCAATGCCACAGTGAGGACCCGACTCAGTACAAACCAGTTCTGAGTACGAGGATAACCCTTTGCAGGA
PV_Br_SNP_0286	A03	20138490	CAAAGTGGCAGACTTTGGGTTTGCGAGAGGCGGTCCTACGGACTCTAACCAAACCCACATATTGACACAAGTGAAAGGAACAGTTGGTTATCTCGACCC(G/A)GAGTACATGAGGACTTATCAACTCACTGCCAAAAGCGATGTTTTCTCCTTCGGGATTTTGGTTGTGGAGATACTCACTGGCCGTCGTCCGGTAGAGGCCA
PV_Br_SNP_0288	A03	20356994	CCATGTTGGTCACAACGAATTGGCGGGAAAACTTCCGAGGTCCTTGTCCAAGTGTTCTTATCTAGAGGTTCTAAACATGGAACACAACGCAATCAGCGA(C/T)ACTTTCCCATTCTGGTTGGAATCTTTACAAAGTCTACAAGTATTAGTCCTCCACTCTAACGAGTTTCACGGGTCGTTACAACATCATCATCCCAAGTCTG
PV_Br_SNP_1082	A03	21105376	AGATATGGATCCATTTTCAACATAGAGTGTTCAGAATTTCAAACTAACTTGAAAAGTATTTATGTGCTTAAACAAATCTTTTAAGTTTACTGTTTCCGC(G/A)TAGCTTTATAAGGGTATTCAATTCTTCTTGGGATAAATTAGTCAATCTATTTGTTTTCTGTTTTGGGCAAAGTATAATAATTTCACCTGGTGCTGATTAT
PV_Br_SNP_0294	A03	22408309	ATCCTTTGACTGGTGGAGCCGCCGCCAAAAGAATTGCTCGTATCTGATTTTCCTCTGTCCATTGATATTGATGGTATTCCCCTTTCTTCTTTAAAGCTG(G/A)CCGGATATGAAGATGGGTAATTTGTAGGCTGCTTGAATTCAATACCCGAAGTAGAGTAGCTGCTGTCTTTCTCTACCCTTTTCCCTGTCTCCTGTGCATT
PV_Br_SNP_0296	A03	22821489	CAATATCATCATCATCTCCTACAACTCTTGTGGTTTCCATTTTCTAAGTTCAGCATCGACGATGCAATGTGCTTCCTCTGCGATCTCTGCATTTCTCAA(C/A)GCAGCATCAATCGCATCATTCACCTCTTCCATCTCTTTCAACTTCTCCTCCAACATTCTCTCCCCTTCTACTCTGCTCGCGTTGATCTCTTCCAGCTGCG
PV_Br_SNP_0297	A03	22926755	ACCACAGTTTAGATCTAGTTAGATAAAACAAAACACCCGAGTTTGAATGAAACTGTCGAGTTCATCTCTTTGACGTTTTGATTCTCCGGCGAGATCTCC(G/A)GGAAGCATAGAAGAAGACGAAAGCAGCTGTTCTCCATTCTGTGACTGCTCATGAGATGAACGAAGAAGACCAGTGGAAACCATGTTAAGCGTTTTCCGGT
PV_Br_SNP_0298	A03	23143245	AACGCAAAATAGAGAATTTATGAAAATTTACCAGCTGATTCGCCGGATGAGTACTGAATGTTCCGGTTATCGGAGGAGGAAAGAATGTTCCGGTTACCG(G/A)TGGTCCAACCGGGTAACCCCATGTATTACCATTCAACTGAAACACGATAATAGACAATATAACATTTCTAAACATCAATCATTGATTTAGCTATCATTTC
PV_Br_SNP_0309	A03	26161200	TAGCTAATCTCTTGGACTAGTGAAGATTTTTCTATTATGGGTTTTTCTTGCCAGGTATCTCAGACGATGCAAATACCGTTCTCCTATGCGGAGGATAT(T/C)ATTGGTTTAGAAGGAGCTAATATAGCCTTCATCCGGAGAAGAAGCGGCGCTACCATTACCATTCAAGAGAGTCCACATCCTGATCAAATCACAGTTGAAA
PV_Br_SNP_1065	A03	27864125	CTTGGCTACATCAAGGTTTGTTTTACTTAAATGATCATCATATAACATAACAATGGGAATATAATGTGGTATCACTGGTATGTATCTGACGAGTTATGA(T/A)TTACATTTGCAGCAAGAAATGGCTGATGCGGCAAAGGCCTCGATGGAGGGTAAGGGAGCTGACAGGAAACTTTTGGAGACGATTTGCAAGAACTTCCTCA
PV_Br_SNP_0312	A03	28510242	TGGCTCCTAGTTCCACTCTGCGGAACTGGGAGATAGGATTTACCACATGGGCACCACAGATGAATGTGGTATGTAAGCAGTAGTGAACACATCATGTTT(A/G)GTATGTTTATTAATTGGTTAATAAATGATGCTCATATTTTTTTGTTAACGGTTCAGGTTGTGTACGGTGGTACTTCTCAAGCTCGTACTGTTATTATACA
PV_Br_SNP_0314	A03	29213500	CTTGTTGGTGAGGAGACAAGTGAATGTGACGTGGAGAAATACTCATCTGGAAAGACTCTTTTTCCCTTGGAAACCGTTGTTGAGAACCACAATGAGGAG(A/G)AGAGCATCTTGCAATCCGAGATCTCTGTGACTACTACGGATCATCATCAGAAATCTCTCACCGGTTCCAAGAAGAGATCACGTGCGACATCGGCTGATGT
PV_Br_SNP_0319	A04	601636	GTCGGGATCGTAGTCGAGGCTGTTGCAAGAGAGGTGATGAGCGTTGTGGGTCCACTTCCACCACGCGATGGAGATCCCGGTGATGCAGTTGCCGGAGAG(A/G)AGCTGAGCGAACTTGTTGTACGACTTGTTCTTCATGATGACGTAATGCCCCGAGTCATGGCCAATGTACGCGCTCTGCATCCAGAGGAGGCCGAGTATCG
PV_Br_SNP_0320	A04	934960	TTTGAATCTATCGATGATGTTCTCTTCCTCTTCTTCAAATATTCTGTTGAATCTATCGATGGTCTTTTCGCCTTCAAACCTTGCTGCGAATGCCGCCAA(G/A)TAAATCTTCTTATGTTCTTCCATGTATAAGTACTTCATTGACGTTGTGTATGTAATATCATCTCCACGATCAAGAGCCTTGAGATCTTGAAATTTCAGTG
PV_Br_SNP_0321	A04	1247632	TAGACAGAGTGTGTTCTTGTTTTACTTATTTGATGGTTGGATGCAAAAGGTTATTAGTGTTGAACCTACCGAAAGCGACATACTTTCTGGTGGCAAACC(G/T)GGTAAGAACTCACATACCATACAATCAGTAATGTGGTTTTCTTGGTTTACTATTTTTAGGTATTTATCTTTACTTGAAATTTGAGTATACGACCTCACAA
PV_Br_SNP_0323	A04	1258335	GTGTGAGCCTTGCTGGTCATGCTCAGGGACGATCGCGAAGAAGAAGTTGATTGAGAAAGATGTTGTGATGTTTGTGGATGAGTTTGTGAATGTAACTGG(G/T)TTGCTGTCGAAAGCTTGACCGTGTGTGTGTTTTGTTATGTTGGTTAAAGTGAAGTAGCCGTTGTAGCCGATTCCTACAGATCCTGTTGGGATGAAGTAGC
PV_Br_SNP_0327	A04	2800639	TACTGACTGAGTTTCCAAGAGGCAGAAAATCTTGTTCAGTAAACTGAGGTAAAAGTTGTTGTAGTACTACAAGTTGTCTTTCAACTGTCTGTTCTAAAG(G/A)AACTTGTCTTGTTTCAGGAGCTGAAGGCTGACTTCTGTAGAAACTTTTCTTCCAAGCATCCATGAGAGGTGTTGAAACCGCTTGCTGAAGATGCTTGTAC
PV_Br_SNP_0331	A04	6717123	TAATCATTTAGCAGAGGAAGGAGCGGTCGTGGACTTGCAGGATGTGTTCGTGAGGTTCACATTCGACACAACCTTAGTGATGGTAACTGGGTCCGCCGA(T/C)CCTAGATCTCTCTGCGTTGAAATGCCAGAGGCTGATGAGTTCGCTAAAGCTCTTAACGATGTTGGTGAAGGGGTTATGTATAGGCATGTTAAACCGAGGT
PV_Br_SNP_0332	A04	6848553	GCAGGGTTACCTAGTGAACTGACCAATACCTCGAACGATAAAGTGTAAGTCCTTGTCAGAAACCTCAAGTTGGGCTTTCTCCTGCTTGATTAATCAGT(A/G)TATGAATCTCGAACAACTACTGATGATGAGATAGAAGCTGGTCAAGTACTTGATGCACTCTTGAGTGATGATTTGGTAGATTTCCTGACAGAAGGGACTT
PV_Br_SNP_0333	A04	6927029	GATTTTACCCTCCCCCAATAATATTGATTAAACGCTGCGTTTTGTGTCTTTTTGTTTTTGTTCATTCTCTGTAGCCAAGATGGAGACAGCAATGGAGGT(A/G)TACAATGACGTTGAGATGAGCACACCACCGATCAAGCGACGCAAGAAGAAGTCAAAGGTGTGGGAACACTTCACCATCGGTGTTACAGAGCCTGGTTGCA
PV_Br_SNP_0338	A04	9525656	AGTTCTGTGGGACCAATTGCGCAGTGGAAACAAGAAAGGAGAGGTTGATGGTTTGTTAACTGGTGCACTTAAGAACCTTTCTAGCACCACGGAAGGATT(C/T)TGGTCTGAAACAATTCGAGCTGGGGGAATGGATGTACTTGTGAAGCTGCTTACAAGCGGGAAGGCTAGTACAGTATCTAATGTGTGCTTCCTTCTTGCTT
PV_Br_SNP_0340	A04	9933369	CCCTGTTCTTCTCTCCGCTTCTTTATCCTCTAAAAGCTCACCAACGTACTCGCATATGAAGCTACCTGAAGGGATCGAGCTGAGAGATCTCACTCCCCA(T/G)CCTCTTGACTCGGTTTTGAAGATCTCAAGCGGGAACTTGATCCCTCTCTGCGTGACTCTTAGGTAGCAAGAAGCAGGGCATTTACAAAGCGGGCCACACT
PV_Br_SNP_0341	A04	10430690	CACCGAGGAAGTGTCCGGCGAGTTGGAGGAGACGACGAGGGGTCTAGCCTTCTGCGATAGCTTTGAGGTTAGCGAGGAGAAAGAGAGCTTAGATAACGA(G/A)TCTGTTCTGAGTATGGACTATCCCTCCTCTAGGGTTACTGGCGACTGCGTTAGCGAAACTGGTGGGAAGAAGCAGCAGGTTGTTACTTTCTTAGGTATTG
PV_Br_SNP_0342	A04	10902499	CCGCAATCTTGATTGACTTGTCAGCAGAGATGAGAAGGTTATCCGACTTGAGATCTCTGTGTATCAAGTTGTGTGCATGAACATAAGCCATACCCCTAG(C/T)GACATCCAACGCCTGTTTGACAGCTATCTTCAACGGCACTGCACGGTTCTGTCTTTTTGTCAGAAACTGCCTCACGGAACCTCCTTTGGCATATTCAGTC
PV_Br_SNP_0345	A04	12871772	TTGGTGATTCGCCACCCACTCGTCTCCCAAGACAACTCTTAGATTCGACTTGCGTACCTTGGAGATCACGTAATGAAGATTGTTCGCTAAGAAAAGATA(C/T)GATAGAGCCACATTCTCGTATATACGAGACTTCGTGTCGATCTTACAAAGCAAGACTAGGATCAGCCACCCGAGCCTGGTCGTCACCGTGGAAGCAGTCT
PV_Br_SNP_0348	A04	14057322	CAACGCGTAAGGCAACGCCTCGTTGAAATTCAAAACCGCCACGTAGGCATCAGCAAGACTCCTATTCGCAGCGCCCATCTCTTTACTCCCTTCCTCGAA(A/C)GCCATCTCTTTAATCTCCAAACTCTTCTTCAGATTCTCAATCGCTTCCTCTCTCCTCCCCATAGCGTTCTTGACGTTAGCTAGCTCGAGGTGGACCGCGT
PV_Br_SNP_0349	A04	14135868	CTTATTATTTCAATAATGAAATGTTATTATCATGAAGTTACCAAGCACTTCTTGAACTGTGAGTCTGCTATAAGGGTTAGCATCAAGCATGTCCTTAAC(G/T)AGCTCTTTGGCTTCAGCTGAGACTTTTGGCCAAGGATCTCTCTCGAAATCGATGTTCCCTCTCACGATTGCATGCGCGATTCCTTCCTCAGTTTCTGCCC
PV_Br_SNP_0350	A04	14646618	CTACGCTGGATTCGACGTGAGACGCTTTTGGGTATACAACGTGAAGCTTAGAGATCAACTCGGTGAAGATCTAGCTCAATGGCTTAGGGAGAGACCTGA(G/A)TGGAAGAAAATGTACGGACAAGATCACTTCTTTGTCACGGGACGGGTTGGTCGGGATTTCAGAAGGGTGCCTGATCTAGATTCAGACTGGGGGAACAATC
PV_Br_SNP_0353	A04	16153523	TAGTAACCAAGCGCTGAGCACACTATCCCCTGCATGCATTTTACGTTCCAGCCAGTTAGTTTAAATTACTATGAGATATAGCCTACTAGTGTATATGTG(T/A)GAATCTTACACTATATGTGACGGTAAGAAGTTTGTTATCCCAACCAATGGCCCATGCACCTGGATTTCCTCTCTCCATGACTAATGCCACAGCTGCTCCC
PV_Br_SNP_0363	A04	17626598	GAAGCGTCATTTCTTTGTATGAAGCGTCAGCTGCAGTGTGGAAGGAGATTCTACCCACTTGACGTGTTGTCTCTACGACCTTTCAACAAAACAAAAGAA(A/G)CTAAAGTTAGTATTGAGGTGATGACTTTGTAATCTAATTTCTCGAACTTACAGATTCTAGAATTTGATTGACGCTCAGGAGGTCTGGGAGTTCAGCAACT
PV_Br_SNP_0364	A04	17743027	GATGATATTGTTAGTGTAACTCACGAACATGCTACTCAAGTCACAAGGCAACCCTTCTCTGCAACACTTAGAGACAGACTCGACCAGCTTCAGTTTCTC(C/T)TCCTCACGGATGTCAGAGAACTTCTCAAGCTGCGGAACAGCGAGAAGCTTGGTCATGCAGAGCTTCTTCATGAACCTCCAGTAATCACCGTACTGCGCGA
PV_Br_SNP_0366	A04	17920108	TTGGGCCCTTTCGTATACTTAAGCTTAGATTAATGAGCTTCACGCATAGGCCCATTACGTCGAAACCATTGTTATAAAACCTAGATGAATTTTATACAA(C/G)TACCTTTACGATGACTGGCCATAATTTATAAGGCTAATTCGACTGGTGAATTGTCACATATATTATCTTAGATAATAACCATTGTAGAAGGCCATAGTTC
PV_Br_SNP_0372	A04	18705486	GGCTCTTTGTCTGCATTGGACACTAGGCCAGGTTCTGTCTCGAAAAGACTGGTAGGCACCTGGTTTTGTCTTGCATGCCTCTCTTCTTCCTTGTCGTTT(G/A)TGTAATGTTTGTTTCTTCAATAATGTTGGAACAGGTAGATGACCTAGAAGATGGGGAAATAGGAGCCTCTGGGGATTCTCACATAGATCTCCAACGATCC
PV_Br_SNP_0374	A05	37346	GAAGACGCGTAATTCAGAAACTATGGCCTGTAGTTGTTTTTCTGTTTGCATCAATACTCTCAGTTGAGTACGTTGCCACTTGGAATAACTCATTGCCTT(C/T)CGATCAGGCTCCAAGTGAAACTAGTGTGCATTGCCATGACTGCTGGAGCATTGCAGTTCTCAACTTTAAATTTTGCCGGGACTGTTGGCTTGGTAAGACA
PV_Br_SNP_0375	A05	60311	CAGTGCGTATGGATTCAACAGGAGACTTGGAGGTGTCGAGAAGAAGAATCCTCTGCCTCTCTGTCTGAATCTCCGCCTTGATGGTAACATCCCTGACTT(G/C)GGAAGTGGAGCTGTTGTTGACGCTTATATAGCTACAGAACGTCTCTCCCAGATAGATCGCCCCGAATGACTGCGGCAGAAGCAGCAGACCTGAGAGACCG
PV_Br_SNP_0378	A05	519882	TGGAAAGAAACATGAGTTTGCGGATTTCGTATGGTTGCCAAGCCAAGGCAAAGTAGTGTATCGAATGGACGACCGAGTACCTGTCAACACATCGGGCAA(C/T)GGTTTGTTTGACCTTTTGCCGTTCCGTTCACAGCTCTCGGCGGCATTAGCCATCATAAGATCATCAGGTTCATCTTTGTTTCTAAAGCTTAGGCTTTCGC
PV_Br_SNP_0381	A05	1125894	CAATGTTGTAAAGCAGTATTTGCACGTTCTGATTTATCCTCTTTGATGTATACTCAGGCTGCACAGATTCTTGTCAGAAGCGAAAGCGAAGGACCGTGG(T/C)TGAAGGAGATATTAGAAGAGGTTCACAAGGGTGCAGATAGGCTCTTCAAAATGATTGGAGAGGCATATTCAGTTCTTTCTGACCCAACCAAGGTTTGCTT
PV_Br_SNP_0388	A05	5146576	ACAATTTTGGTAGATGTTTATTTTATGTTTTTGTCCGTAGATTAAAGGAACAGACTACAAGACCCTTGATGGGACTTGTGTACGTGACTACATAGATGT(A/G)ACAGACCTTGTTGATGCTCATGTTAAAGCTCTTGAAAAGGCGAAACCTAGCAGAGTTGGAATCTTTAATGTTGGTACCGGGAAAGGTATGCAAGTGGTCT
PV_Br_SNP_0393	A05	6473191	ATCAAGACCAACCTTTCCTCTATATAAGCTACACCGAGAAAAAAGGTAGTGAGATTTTAATAGCTGAATCTTGCAATGCAAGAGACACTTACTCAGGTA(T/C)AAGGGTTTTGAAACCAGGGTCAAGCTGTGAGATTTTAATAGCGTGGTTCTTGATCTCAACTTCAACACCCCACCATTCTTGAATCACAACAATCCCAGGC
PV_Br_SNP_0394	A05	6646646	GCAGAACATGATTCAGCTGTGGAGTGCCATTAAGCAACCTAATGTTTTCCTCCCAACTTTATTTATCTTCTTATGGCAAGCCACGCCGCACTCTGATTC(C/A)GCAATGTTTTACTTCACGTATGTTTCTCTCTTCCCACTGAGATGGTAGTAGTTACTTAAGTTGCTGCTAGCTAATGCTAATGCTAAAACTATCTCTCAGA
PV_Br_SNP_0396	A05	6749255	GGTATCTCATCTTGGATTTTCATCTTCTTCTGCGACTGACTAGAGGAGTCCTTGTTCTTTTGCGACTAAGTAGACAAGCCCTGAGTCGATGGCTTTCTT(C/G)TCCCTTCACGAATCACAGATTTCATAGGAACATACTTCTTATGACTACATGCCTGTGCGGACGAGGCACTACCACCATCTAAAGTGGATTGGAACATGGG
PV_Br_SNP_1057	A05	8925567	TGATACTAGTGATATTGCTAGTGGTATTGCTAATGTTCATATTAATGATGACGCCAATATTGGTGCTACGGCTAATGGCGACATTGGTGCTGATGTTGA(T/C)GCTAAAACTAGTGATTTTGCTAGTGTTGAGTCTAAAGCCGACGTTAGTACCAATGTTAATGCTAATGCTGATGCCAATGCTAATACTGGTACTCTCAGTA
PV_Br_SNP_1081	A05	8964613	GTAACGACGGTGACCCTCGCGGACACGGTAGGGAACAACATCCCTCAAGAATTTGGAGAAATGGTGACCTATCTCAAAACAAACACTCCTGGAATTGAT(G/A)ATGTTGTCTTCAGCCTTCATTGTCACAACGACCTTGGTCTTGCTACCGCCAACACAATCTCCGTAATTAACTATTTTGTGTTAGTCTAATTAAGAAAATC
PV_Br_SNP_0413	A05	10244748	TTCAGTTTCATCTCTGGTAATTTCCTCTGCAGCTTGTTTCAGGAAGGACCAAGAACTGATCGGAACACCCGCTCACCGGAACCGGCATTGACCCGATTC(T/G)CACCTGAAAGAATATGCTTCGATATCCACAAACCCAACAATGAATCTACTAATTTTGCTAATTTCAAAACAGGTTTAGAGAGTATAGTATTGAAAAATCT
PV_Br_SNP_0415	A05	10368006	GGCCTCGACGACAAGCCCAACGACTCTAAGACCACTCAGCTTCAGCCCAAAATCATTCAACGGTCATCTCCAAGCTTAATTACAAGCGACAAAGAAAGC(C/A)TGAGAGTGATGACGTTCGGAAAGGTACAATCTGGTCCCCTCCAGTTTGGAAACAGATTCGCAGCCCTTGATTCCTTGGAGCTTCCATGATGTTGCCACCT
PV_Br_SNP_0417	A05	10731008	TCACCCACTGACCTATTGTACAGAACCTCGAAATCTCGGACTTCTCTTCTGTTGGATTCTCCTTGGACACGTTCTTTCACAAGGAAGATCACCAAAGGC(C/T)CGTCTACGTGGACCTGCGTCGTGCTGAAACACGAGTACATCTCCTCCACCTCTACTTGGAACTTCTTGAACATCTCTCTAGTGTAGACTCTCGAGAGCTG
PV_Br_SNP_0418	A05	10865607	GTTTTCACTCCTCAATCTGTTACCATCTTTTCGTAATGTCTCCATCTGAAACAAAAACAAAAAAAAGTAAAGAAAATCAAGATTCAGTTCTTGTTTTTA(A/T)AGAAAGTTTGACAAGTACCTCCTCTGGATCCTCCAGCTTAGAACTTGCTATGATTTGAACAGACTCCAGCTTCTCAGCAAGTTCATAATTCTCAAAATGT
PV_Br_SNP_0424	A05	11496787	TACCTTCAACTCCTTGATTTTCTCCTGGAGGTTTGAGTTCAAGTCTTTCAATCTCTGTGCTTCGTCTCTTGCTTGATTCACCATGCGGATAGCGTCATT(G/A)ATGATAGCAACCTTGTCTGTTTTCGGAGCTCTCCCAGGTTCCAGTACGGAACTCAACTCCGTAAATCTACAGTTAATATTAAAAGATTAAAGAACTTAGA
PV_Br_SNP_0426	A05	11626502	TAAGAATGACTTGGCCACAACGAATATTTTCTTGCAGATACTCCAAATTGGCTGAATCCATATGTGCATAACTCAAGATAAACATTTCAAATATTTCTA(G/A)GGAAATCTGCGGTGATTCGTATTGAAATGTTTTCATGGTCTTGCATCTGATGGATGAGTGACCTCTCCATCCTTCTAGACATGTTAAGCATGTGATCTTC
PV_Br_SNP_0430	A05	12088226	GGTGGCCTGGGCTTCAGAGACATTCAAAGTTTCAATGACGCCCCCTTGGCAAAGATCAGTTGTCGCATCCTATCGTCACCATCTTCCCTCCTAGCTCGC(T/G)TCCTTGCTGGCAAGTACTTTCACAACCAAGAGTTTCTCCATGTTAAAGCTCCATCTGCATGTTCCCACGGCTGGAGAGACATTCTCATTGGGCGCGACCT
PV_Br_SNP_0432	A05	12171714	TCAAGCGATTACTCAATCGTTTCACGACCAGACACCGGTTCTGCTTCTGATCACGAACCAGTTGCGGAAGGATCTGAACAGTGTCAACGAGTACGAGGT(T/A)AGTCTAGCTCTGGAGTGTTTGTCTAGGATTGGAACTCGTGACTTAGCTAGGGATTTGTCTCCTGAGGTGTTTACTCTCTTGGGTAGTAGCAAGTCTTTCG
PV_Br_SNP_0444	A05	12922255	ATACCTGCTCGCTGATGTGTTAGTGCGCGTCGGTCGATTCTGGTGTGGTAATGTCGATCGATGTCAACCTGGCCTCATCTGTCGATAGATGGGGAAATC(G/C)TCTCCTCGGGTTTCGTTTGGTTTCTTTTAACCTGCAATAATTTAAATGCGAAAATAAGTAACAATTTTGAAATAAAATTAAGTAAAATTACCTAATAG
PV_Br_SNP_0446	A05	12998177	GTCTACTCTTTAATGCTATCTCCCGAAGACCGTAGCTGAACCATTTCACGCTTCTCGTTATTCGAAGTCATCAATCAAACTTTACGATAAAAACACACA(A/G)AAAGTTTGTTTTTATCGAAGAAACCGTAAAAAATGTTTCGAGTCAAAAGACGGTCCAAAGAGACCTAAGACGTGACTCGAAGCCCACTTACGATTTCTTA
PV_Br_SNP_0448	A05	13282738	TAATGGTATCAGAACCAAGTATTACAAAATTTAAAGCTTACGCTTGGAAAATCAACGCTCCCAGAAGATGTTGCACCTAATATGGAAAACGGCCTCAGG(G/A)TATTTGGCAGTGACAGGAAACTTAACACGTCGCCATATGCGAAGCGATAATCACTGTCCTCAATGTACAGAGATGACGAAACAATCAATCATGCAATTT
PV_Br_SNP_0451	A05	13486515	ATTTCCTAGGGAAGGAGGAATTGAACCGGTAAGGTTATTTGTATGAAGGGTAAGGGAATCTAGACTGGTCATGTCACCGATCTCAGGGGGTATTTCACC(C/T)GAAAGTTTGTTTTCAAACAAGTTGAGAAGAGTTAAGTTCTTCAACTTTCCTAAACTGGAAGGAATGTGGCCGGTCATGCTGTTTCTATCAAGGCACAGCT
PV_Br_SNP_0464	A05	15526140	TTTGGATCCAATTTTATGCCTCCACATCCACGAAACCCACAATGATCCCCCTGCTGCAAACAGAAGCCATATAAGCTTCAGTGCCAGCACCTGATTCCA(A/G)TCTATTAATTTTCTCAATCCTAAGCCACCTTCATTCTTCGGTGTGCAGATAATGTCCCAGCTTATCTTCGCACCTCTCGCAGACTGTGGAGCTCCACTCC
PV_Br_SNP_0465	A05	15526505	CTTTGTCGAGCAAAGGTTTGAAATCTGACTTCTTCATTTTCTTGGAGGACAATGGAACTCCGAGGTATCTCACCGGCAGTGCCCCTTGCTTTATTCCCA(T/A)AGACTCAGCCAATTCTTGACACAAAGAGTGACTTCCACCATCAATCATAAGCTCAGTCTTATCTCTGTTGATACTCAGACCCGAGATTTGCTTAAACTCA
PV_Br_SNP_1042	A05	15800094	ACCATGCATGGCTCGTGAACAAGACCAATACATGCCAATCGCTAACGTCATAAGGATCATGCGTCGGACCTTACCGCCGCACGCCAAAATCTCTGATGA(T/C)GCCAAAGAAACGATCCAAGAATGCGTCTCAGAGTACATCAGCTTCGTGACCGGTGAAGCTAACGAGCGTTGCCAACGTGAGCAACGCAAGACCATAACTG
PV_Br_SNP_0471	A05	15949259	TGCTTTGACTCACCAGGTTACTCACAACAACCTCTATGCTTGTTAAGAAGTAACTGTTTGAATTTTCGCACTTTGTACTAATCAAAACATTCCTTATAA(C/T)TCTTATCTGATTGTCATGGGTTCTCTTCTATAGGATCAATCTTTAACCAAACACTATGATAGCAAAACTGAAGCTGTATACTCTTAAATCTTACATATAA
PV_Br_SNP_0476	A05	16404787	GTCTTATGGACTTAGCTAGTAGAGCCGGCAGAGAACTTAGCAGAAAGAAACGATTAACATCACATCAAATGAGACAGAACAATCGAACAAAACATTACC(C/T)TGCCTAATGGTCTCCAATTTCGATGCAGCAACCGAGAAAGCGTTAGATATATTATAAACAGCCCGACCCTAAATGAGAAGGAAACAAAAAAAAAAACAGT
PV_Br_SNP_1052	A05	17215318	ATACATGGACAAAGCTTTTCCCTTTCCCTTGTCACGACCACTTGGATTCACTTGTATATTCCTGATCATCAGCATATCGAAGAAGACTTGTTACTGTAC(G/C)ACAGTATACTTCAACTAAGCATATCAAACGAGGTTATTTGGACTCACCAACGTCTTCCTCCGATGGAGAAAGTATCAGACAAGTAAGTATCTTCGAGCAA
PV_Br_SNP_1051	A05	17589777	TTTGTATATATAGATTCCTCCTCCGGGGAGAGGAACATTGTATATCAGACCAATCTTGTTTGGTAGTGGTCCTATACTTGGCTCACTTCCTGTTCCCGA(T/G)TACACCTTCACAGTGTTTGCATGTCCCGTTGGACGTTATCACAAGGTGAGTGTGTTCTTCTCATTCTCAACATCATCTTTATTATTCTAAACTATAATGT
PV_Br_SNP_0492	A05	18313091	CTCTACATGTTACAATAAAATTAATCTGTTATGAAACGTTGCACAGATGAAAACTGCCTTGAGAGCTCATTTTAGTGCTTGTGGAGAGATCAAAAGGAT(T/C)TCTCTTCCTTATTACCAAGAGACTGGTGATAGCAAGGGGTTAGTAACTCTTCTTTGCTAGTTTGGTGAATGATGATGATGAAATATATGTTTTCTTAATC
PV_Br_SNP_0493	A05	18351619	CGCCGCATGGTGGAGTCAACTAAAACTATCACGTACTCGACGAGGGAAAGAGAAGATCTCATCATGGGATAAGTTAAAAAAACATATGCGCAAGACATT(C/A)ATTCCCTACAACTATGAGAGTCTTCTTTTGCAAAAATTCCACAACATTCGTCAAGGCTCGCGTTCCGTAGAAGACTATTCGAATGAATTTTATCAGATGC
PV_Br_SNP_0494	A05	18458313	CATCTCACTGTTACTAATCCCATGAAGACTCAACGTAGCAGTACCGATGTTGTCAGTGCCAAGCATCTCTTCTCTGTAGCATTTCACTTTCAGATATTC(T/C)TCTCCTGCTAACTCCTCAAACTCAAACTTCTCGTTCCACACACCATCTGCCGTCTTTGTCTTCTGTATAGTCTATTACACAAACAAGAAGAATATTAAAC
PV_Br_SNP_0500	A05	20060453	TGAGGATTTATATTGTAAACAACATAGTATTAACAAAACGCATAGAGTTTCTTTGGGCCACTTGTGACGGTCTTATATTAGTGGAAATTAGTGTGCCTG(A/T)GTTTGGCGTAGAAGGAAAAAGAAGTTTTAACTTTAAATGAAATGTAGTGTGTGCGCAATATAAAATTTCAAACCAGGGAAACAAAGGGGGAAAGATTCAT
PV_Br_SNP_0509	A05	22293235	GGAGGTGGAGAAGCATTACCTGCATTAAAGAAGTTTAAGACAGGTCCAGTTTCTTTTGGTTGTTAGGTCTAGCTTGTATAGACATGACTGTATTTGACA(T/G)CTCATTTGATTTCAAATGCAGCATCAGGATCTGCAAATATACATGCCAAAGAGGGTTCTCTACTCTCAAGACTGCGGTTAGACCCTAAGAAAGATGATGA
PV_Br_SNP_0510	A05	22623229	GTCCAAAGCCCTTCATCGGTTTTCCACGACCTAGAATCCGAGTTCCAGTCCCCATCCCAATATGACTCAGTGCCATTGGTCTTAGTCCGCCTCGATGAA(A/T)CCCCTAAGCACTCAGATTTCTTGAAATACTCTGAAAGCAAATGTGTCATTGAGGATGATGATGAGAAGAGGTTGATGCCATTGCGGAGCCCCAGTTCGGG
PV_Br_SNP_0512	A05	23153672	CTTGGCCAACCCTTTCGTTATAGCTCCCACAGCGAGCTTAACCTTCTTCTTCCTCTTCGCTTTCTTATCCTCTTCATCTTCTGCTGAACACGGTGTTTC(T/C)TTAGCAAACCGGATCCATGCATCGTCGAGATCGTCCTCCGCGTGAGCAGGTGCAGTAGCCAATCCAAAGAAGAACTTTCCCTCATTGTGTTCTGAAACAA
PV_Br_SNP_0513	A05	23581175	CCCACAAAGAAACAAGATTCTTTTATTAGCTCCAGTTATTTTACCGCCCATGGCCTTGACAAAAAGCTGGATAAACTCAGGATGTCCCTCATAGGTAGC(C/T)GCAGTGATCAAACTACCATCAACAACACAGACATCAGGTGTCACTGGTTCCACCCACTTGGCCCCTGCAGCGATAAGAGCAGGTCCAACAGTTGCATAAG

Marker ID	Linkage	Position*	Flanking Sequence (100bp flanking the mutation site, and the SNP site is in brackts)
PV_Br_SNP_0517	A06	251018	TTTTACCTTTTCAAAGAAAATTTAGCAAATGCCGACTGTAAAATTGGCACAAGCACGGCCTCAGCTGGATATATGAACTTTTTTTCACACATCGATGCT(A/T)TATCTGGGTGTCCTTTCCGGTGATCTTTACTCCCAACTGATGTTTGAGGAACATTCACCGCAAAGGTTGAATTAGTTTGACTTGTGTTGTCATTTATATT
PV_Br_SNP_1043	A06	427379	GGTTGTGGGGAAATATATACTGATATTTGGAGGAGAGACCTGGCCGGACCCAAAGGCACATTTGGGACCAGGGACGTTGTCTGATGAAGGGTTTGCGTT(G/A)GACACTGAGACACTGGTGTGGGAGAGGTTTGGAGGAGGAGCTGAACCGGGTCAACTTGGTTGGCCCGGTTACACGACTGCCACCGTGTATGGAAAGAAAG
PV_Br_SNP_0519	A06	796251	TCACTATTTCCATTCTCCGGCTTATAAACATATGAGTATCTCCTTCACTCTCAGACACAAACATAATCTACTCAACTTTAGAAAATGGTCCAAACACAC(A/G)AAGGCAAATGCGTTGGGTTTATTGTTCTCTTGCTATGCCTCACTTCGACATTAGCACGCACCCTGATCCTTAAGGAACAACTCCCTATGATGAAGCTGCA
PV_Br_SNP_0520	A06	877234	GCGTTTGCATTGCTTCAAACTCGCTCTTGAGCTTTGCCATATCCTCTTTCAATCTCTTAATCTCCATCTGCTGGACATTCATCTCGCGTTTGGACATGC(A/G)CCGAGCTGGACCTGACTCTGAATAGTGTGAACTAGAAGGGTTTGGACTGAACGTTAAGCCACTCCAATCATTGTTGTTGTGGCGGCTCCGACTCATCTTC
PV_Br_SNP_0521	A06	1074004	TTCTAATGGAAAATGGGGTATCCAGTTCGCGTTGGATTATCCGGATGAGTATATCACAGCTGTTGGTGGAACCCACCAATTTGATATCAAGCATAAAAC(A/G)ACGCTGATCAGATCTTTATTCTTCAAAACCTCCCGTGGAAGGACATCTGACACACTCGGTCATAAGAAGAATCCAATGATACCATCATTAGTATCATCTG
PV_Br_SNP_0526	A06	2011195	TCTCCAAAGACCATCTATTAGGACAAGGCGGGTTCGGATATGTTCACAAAGGAGTCTTGCCTAATGGTAAAGAGATCGCGGTGAAGAGCCTTAAAGCGG(G/A)AAGCGGACAAGGAGACAGAGAGTTCCAAGCAGAGGTTGACATCATCAGCCGTGTCCATCATCGACATCTCGTGTCTCTTGTTGGATATTGCAGTAATTCA
PV_Br_SNP_0528	A06	2238277	ACTTGTAGACAAAGGCAGGGAACATTTCTACACGATGACTCAAGACTATGGTGTAACACCAAACTCGCAGCACTACGCTTGTATGGTGGATCTTCTTGG(T/A)CGAGCTGGGCTTTTAAAAGAAGCTCACAGTCTAATGAAAGCAATGCCCTTTGAGCCAGACGGTGCAATATGGGGAACTTTACTTGGTGCAAGCAGAGTCC
PV_Br_SNP_0530	A06	2691656	CACGATGATGAAACTCTGTAGTGTGACTAACGTTTCGTTTGATCATCTCATGGTTTCAGGGAGAAACGATTGACGAATGCTTTAACGTTCGGGAGGATA(A/T)CACAAGAGGCAGCTTGTGAGTTGCATTTTCCTTTCTCCTTTTATGTATTTTATGTTTCTCAGTGTAATCTGAAATCGACCTATTTTGCTTTCAGATGTTA
PV_Br_SNP_1033	A06	2706689	GCGTGAGCGTGGTATCACCATTGATATTGCTCTTTGGAAGTTTGAGACCACCAAGTACTACTGCACGGTTATTGACGCTCCTGGTCATCGTGATTTCAT(T/C)AAGAACATGATCACTGGTACCTCCCAGGCTGATTGTGCTGTTTTGATTATTGACTCCACTACTGGTGGGTTTGAGGCTGGTATCTCTAAGGATGGTCAGA
PV_Br_SNP_1034	A06	2707359	TGGTATGGTTGTGACCTTTGCTCCTTCAGGCTTGACCACTGAGGTTAAGTCTGTTGAGATGCACCACGAGTCACTTGTGGAGGCGCTTCCAGGTGACAA(T/C)GTCGGGTTCAACGTGAAGAATGTTGCTGTCAAGGATCTCAAGCGTGGGTATGTTGCATCCAACTCCAAGGATGATCCTGCCAAGGGTGCTGCTAACTTCA
PV_Br_SNP_0534	A06	3924739	CACCGGTGCTCTGGAAGTACTTGGAAGAAGTTACAATCGTTGTAATGATTATAACATTGATGATGACGAAGAGGTAAGGTGGTTTCAGCCACGAGAGAA(A/C)TGAACTCCAAAGGATTGGGAACCGAGATGAGGAGAGATCTAGTGCGGTAGGAACACAAGACATTACAAACAGAGACATAGCGGTGATTACGGTTGAGACA
PV_Br_SNP_1039	A06	6303418	AGACAGATTCACAAACTTGTTAGTGTTCATTGCTAACGAGCACACAACTCTCTCTCTACTCACACACACAAACATCATGACAATGATGATGAGCCTTAC(C/A)ACATCATTACCATACCCTTTTCAAATCCTACTAGTCTTTATCTTGTCCATGGCATCAATCAGTTTGCTTGGCCGAATACTATCAAGACCCACAAAAACCA
PV_Br_SNP_1040	A06	6304099	CATGAGATTGTTGTTTGGAAGGAGACATATCACGAAAGAAAATGTTTTCTCCGACGAGGGAAGGTTAGGACAAGCGGAGAAAGATCATCTTGATGCGAT(T/C)TTCGAAACCTTAAACTGTTTGCCGAGTTTCTCTCCGGCGGATTACTTGGAAAAATGGTTTAGAGGTTGGAACATTGATGGTCAAGAGGAGAGGGTGGTAA
PV_Br_SNP_0545	A06	8198336	TGCGTATGGTGGGTTTAGCGCAAGCATGACGCCTTTTTTCAGTGCAACACGTATTGTGCTTAGTAGACATCTTGGTGCTGTGTTCTGTTTTGCAAACAT(T/A)CGTGGTGGTGGTGAGTATGGTGAGGAATGGCACAAAGCTGGCGCGCTTGGAAATAAACAGAACTGCTTCGATGATTTTATTGCTGGAGCAGAGTATTTGG
PV_Br_SNP_0549	A06	10126924	TTCTTAGGTGCCAAAAGACAAATCCTATACCCATATCTAAAGAGTTTTTAAAGACATGTAAAAAAACATAGAACATGCGAGGGAAAGAAAAGACGAAGT(G/A)AGAGAAAAGACAAACAGACACAGTTTGGGAAAAGACACATCCTCTTTACTTCATGACCTGCAAAACATGCTAGAGGAACGAGCAAGCCGAATAGTCAGAC
PV_Br_SNP_0551	A06	10741922	TAAGTTTCTGCTTGGTCTAGACGTTTCGTACAACAAACTGTCAGGACAGATTCCACGCTCCTTGGGGAATTGTCTCTCACTAGAATATCTTTGGCTGCA(A/T)GGTAATTCATTTGTTGGTTCTATTCCAGATATGAGAAGGTTGACTGGCCTCAGGTTCCTTGATCTGTCCAAGAACAATCTCTCTGGCACGATACCTGAAT
PV_Br_SNP_0554	A06	11039171	ACGGCTCCCATGTTGTAGATCCATGGCCGCCCGAGTATCATATTGTATGATGACTTACATTCGACGACAAAGAACTTGTGGACATGTTGATCCCTTCAG(T/C)GTATACCGGGAGAATGACTTTTCTGGCGGTCTGCTGGACTTCTCCGCTGAATAATATGAGCGGGGTGATGTTTCACGTTAGTGCATTCTCTTCCAGCCCT
PV_Br_SNP_0565	A06	14855293	TTCATCACCACGAGCTGTGGCTAGACTCGCTCTGCTAGCTCTTGAAATCTTGGAACCTATTGTTGGGGAAGTAAGATAGCAGTTTGGACTACTAGACGC(G/T)GAGGAAGCACTAGAGAGTTTCCTTGAGAGGTAAAGATCCGCCATGTCATCGTCATCATCCAGCAACTGTTCGAGCTCATCTCTTACCTTTTGAACTCGAG
PV_Br_SNP_0567	A06	15627812	TTGTTATCGATTTGTCACCTCCGAACCGTTACGAGTTGGATTGATTCAACCATTAATTACTTATTAATTAACTCTTTCGCCCGCTACAACCACGATCCC(G/A)GCCACGATCCTTCATTCAACGATTCTGATTCCTCTTGCAGTCGGTGATGCTCCACCCTATAAAAAGGTAAGCTTCATCCCTAGCATCCTCCTCACAATTC
PV_Br_SNP_0571	A06	17743723	CAAAGTGAAAAGACTTGTGCAGAAAACGCTGACTTCAGATTCCAAGCTTCGTGGAATCATCATCAGGAGAAAAAAGACAACGAGTTTGGCCCAACTTAC(A/C)ACAACAGTTCTCAGCAGAAGCAGGTGTTTACAAGCAGCAGCAGCGATCCAGTGAGGTTCCAGCTTCTTGGAGCATCAGATTCAATGAAGCTACCTCTCAA
PV_Br_SNP_0573	A06	18594484	CAGTTATAGCATTATTACTGCATATAGGACTTTCCAAGAAGATGTATTGGGGCAACAAAGGAGACATAAACTTGTAGTAGTTGTTGTTGAGTTTCTTGA(T/A)CTTTCTTTGACCTCCTAACCCTCTAGTCAAAAGAGAAGTGACTCTCTTGAACTTGATAACTGCTTCTCTTGTTTCAACCAGTAGGGATTTAGGATCACTG
PV_Br_SNP_0578	A06	19646308	AGAGGAAGAGCTTGGAGAAGATGCGTGGGTGATGCGTCGTCGTCTGCTGCAGTGTTCTTTTGTGGTGGCATCATACCTCTGCTGTTCATTACAGTCTTA(C/T)CAGAGCGATTTGATAGGATACAGTTTCTCCTGGGGGTTACATTTGTCATGGTCGGAGCCATCCTAGTTGTTTATCTCGTGATGAGGGATAGGAAGATGAT
PV_Br_SNP_0585	A06	20214699	ATTACAGATGTGAGATCACATATTCTTTAGCAAGGGATGCACGTGAATATGTGGTCAATGCAGTTAGAGGTATTCTGGAGACAAGTTATGGGAAAATGC(C/T)GAAATACTTGCACAAGTTGAGAGAGGCTAATCCGGATACACATTCATCGTATGAGATCGACGACAGCAATGGGAGATTTCGGTACATGATTATTGCATTTGGG
PV_Br_SNP_0586	A06	20344798	ACCCAGGCTTCGCTGTGCAACTGCAAGACGCTTGGATTCAAGCTGGAAACATGTGCCAAACACTCACTCAACTGTGCTGGAAACTGAAATTAATAAAGA(G/T)TGACTTAAAGCTTCTGAACAAAAACAACTACCCAAAAATTTAGGAGAGAGTGTGTGAGACTAACCGTTTGTTACAAAGTGCGCAGGTACAAGCATTGGAA
PV_Br_SNP_0587	A06	20423475	TGAACCAAAAGAGGAAATGAGAGCTCTTCTTGCAGTCTCCAAGTCGATCCCACGAGCTTGGAAATAGAAGAGCTGGTCTTCTTCAAGATCGCTGATGGC(A/C)GCTCCGTGTGAGCACTTGACATCGTCTGCAATGATCTGAAGGTTCGGTTTTATGTTCACGGTGGCTCGAGGTTTCAAAAGAAGGCTTCTTGTTAGTTGTC
PV_Br_SNP_0597	A06	23816726	GCCTTCTAGTTTTAGAGGGATGAGAGCTATTGTTGTTGATGCTAAGCCTGTTCGAGCTGCGGTGACTAGGTATCATATGAAGAGACTTGGGATCAGTGT(T/A)GATGTCATGACAAGTCTCAGAACCGCTGTTTCTACTGCGTCTGGAAGAAACGGTTCTCCTCTTCCTTCAGGGTAAGTAAAAGGTTTAAATCTCTTTTGAT
PV_Br_SNP_0600	A06	24527870	TCCTCTCGCATTGTCTCTCCAACACGTCGAGCCAACGGCTAGCTCCAAAAGCGATTCCGCTTTTCACATACTCAGCGAATATCTCGTGCACGTGCCTCT(C/T)GTTAACTTCCACGTGCTCGACCCACTTGACTTGTGAGTATCCATTAGGCATGTCTTGAATGATGCAGCCTGAAGGCTTTCTCTTATACTCATGAGGAGTG
PV_Br_SNP_0601	A06	24904044	TTCCATGTCGCTGGATGTAACCTTTTGCAACTAATCTCGCCTTGTACTTGTTGATGCTTCCATCAGAGTTACGTTTGATTTTAAAAATCCATTTGAGAC(T/C)AATAGCTCTAGCTCCAGCCGGAAGCTCCACCAAGTTCCATGTCTTGTTCTTAACTATTGAGCTTATCTCATCCTCACAAGCATCTCTCCAGACTTTCTGA
PV_Br_SNP_0602	A06	24995754	GTTTCCTTGACACATGCGCAGAGGCAAACGAGTTTTTCGAGAGGAGGCATATCAATAAAACAAGGCAGGATGAGAAAGAGAACCTGGTGAAAGAAGCTA(G/C)CCGAGCTATTCTCTCTGTCAAAACATATGTTGAGCCTATGGATGTGAAAGGAGACCGAAGCAAATCTGTGCTGTTTGATGGAAGCGTGCTTGCACAGGAG
PV_Br_SNP_1069	A06	26158880	TCCCGCTATAAATCAGGGACTGAATCATGTATTCAACCACAACAATATTATCTCAAAGCTAACCAACAATGGCGTTCCCAAAGGTGTTCTTCGACATAA(G/A)CATCAACGGCCAGGCAGCGGGAAGGATCGTGATGGAGTTGTATACGGATAAGACGCCGAAGACGGCTGAGAATTTCAGAGCTCTGTGCACTGGAGAGAAG
PV_Br_SNP_1047	A07	107888	CAAGAAACCCGAGTCTTGCACTTATCTATTGGTAAGACTTTAGTACCTGATTAATAGATAAAAGCATTCTCCATATTAACACTTTCACTTACTGCAGAC(G/C)AAGCTTGAGCTGTCATTGTTGGATGACATCAAGGATGACATTGATTTTTGCGTGAAGCTGGCCAGAGAGGAAAACCTCGTGTTTCTACCAGGTAAAGCTA
PV_Br_SNP_0607	A07	286182	CAGCCAATCCAATGATCAAGCGTATTGTCTCAATGCGAGCCACTGGTGCGAAGACCTCATCATAATCAGCACCATGCTTCTGGACGTATCCCTTTGCCA(C/T)GAGTCGAGCTTTAAACTTATTGATACTACCATCAGCGTTACGTTTTACCTTAAAGACCCACTTTAGGCCAATAGCTTTCGCTCCTGCAAGTAAACAGACG
PV_Br_SNP_0609	A07	611531	GTTTCCACGACGGTCAAGAGTATACTTCCGCTGCTACGTACGGATACAGAGTCTCTGATTGAACGGTTTAAAGCGGTGTGTCTCGAGAATCACGGTTCG(G/A)ACAGAGGGAAGCTTCCGTTGCCGCCGACTTCTGGACCGTGGAGTCCGACAGAAGCAAACACGCTGTTGAGGGTTTTGTGTTACCGTTATGATGAGTCTGC
PV_Br_SNP_0610	A07	797437	GCAGAAAACTGTAATATGAGCTCATGAATTTGTTTTCCCTTTGTAAGAGGAGATCTTCGGTTAGATGTTCGAGATGGGAACGTTGGCTAAGCGGTCCGT(G/A)AACAACTTCAGGACCGATCTGGTGACTCGGTGGATCTTTGATTTCATCAACTAGAGATAGGTTGTAGCTTGGGATAATGTTTGTTTCAAGATGATGATCT
PV_Br_SNP_0611	A07	931243	GTTTAAAATGTGCGGAGTTTCGGTGCCGAGATAGACCGGATCTTAAAGAAGAGGTTATACCGGTTTTGAAACGACTTGTAGAGACTGCAAATTCAAAGA(T/C)AAAGAAAGAACGAAGCAATTTACGTGCACCAAGTCATTACTTTTGTCCCATCTTACGAGTGAGTACAAGCTTACCCGGTTCTGTACATGATTTTTAGTAA
PV_Br_SNP_0616	A07	1592883	TTCGTATGTCTTGTCCATGTCGATGCTCAGAACAACTTTTTGTCTTCCCACGAAACGAAACTGTGGCGCATTGTTGTAGAAACCCGTTACTCGTAAAAC(G/A)TCCCCCAAACGGTATCTATAAAGACCTGTTAATTATATATACGTATTATTATAACAAGTTCATAATCATAATCATGGCCTAAACACAAGTAGGAACTTAC
PV_Br_SNP_0620	A07	4705134	CACAGTTTCTGTAGTTACCTGGTTTGCTCTCTCAATATCTTTTTGTGATGCAGTGGACATTGATGATGTCCTCAAGCTTTTCCTGCTATTTCTAACCTT(T/G)GGAGAGACGCTCTCAGTGGGAGTTATTAGTAGAGGAGAACCATTTACTGGATCTATGCTGAGGCAAGGTGACCCAGGATTTTCTGAGAAGTGATTTGTAT
PV_Br_SNP_0622	A07	5385095	TCTCTCTCTCATGTCGGAACCGCCGCGTTTCTCGATGTATCCTCATGCCGCTATTAGTGATGGGCCTAACTCACATCCAAAAGCTAGTTCAAGAGTGGA(T/G)GATTGCACATACACATATATGTTACTCCCATTCACTCATATTTCTCGATGTGGGACTAATACACCCCCTCACGTTCAGGATTGGACATCTGAAACGTGAA
PV_Br_SNP_0629	A07	6945368	AACTCTAGGTCACCGTTGATTTCATAGACGTCGTTTACTTGGTTGGTGTATTCGAAAAGCAAAGTATCTCCAACTTGAAATTGTTTCCCCTCGCTCCAC(T/G)TGTTATAAAAGTCACTGTCATAGACTTTCCATCCTTCCGAGTCTCCGACATTGTAGGTCTTTCCGACAGGAAGTATCTTGCTCGGTGGTGGTGGAGGAAT
PV_Br_SNP_0637	A07	11626771	AGAGGTGCTACTAGTCTACTACAGAAGGTCCACAACAATATATCATTGGAAAATGTATATAATAAGGTTTTTCTGTTTGAAATATTGAACACAGAATTA(A/G)TATCGAATTTTCGAGTAGAAGTTATTACCATAAGGTAGATGATGGTTGCAAGTAGGACAGTGGCATAACGGCCAAGATAAGCATCGGCGACGAAGCCTCC
PV_Br_SNP_0641	A07	12369740	AAATGGCAAGAAACCGTTAATCAGAGGAATAACCAACATAATCGCTACAAGTAATGAACTTGCCCGTTCCCAATACGGAACCATAGTGCGTGTTTTGGT(T/C)TCTTGAGTCACATATCGTGGACCGTACACTAGACCAAACATAGCCAAAGTGATGGTATCTGCAACGTATCGAAGAAGGCAAACCGTTGCCCAAAGAAACA
PV_Br_SNP_0645	A07	13144591	ATAATCGTTTGGCCTCGATCCCGTTCCCTTCTTCTCCGCCTCGAGCCTTACTCTACAACTCCTATTGTGGTGGGTACTGATATCTACATTGTGGTCGGC(T/G)CGGAGAAACATACGGATCCTGAACACTCGGTCTGGAAAGTTGCGTACTGGAAAGTTGCTTCATGGTCCAAAGTCGCCTGTGTTGAGACTTAGATGAAGCA
PV_Br_SNP_0646	A07	14186738	TCCCGACCTTGTTTCTTTACCTTCACTCAAAGATATAAATTTAATCCTTGTTGAGTTTACCGACGAATGGGCACTAGAAAAGTTCATATCACAATGCCC(A/G)TCTCTCAAAAACTTATGTATTGAGAGGAGTTTCGGTGATGATATACCAATGTTACGTGTGCGTTCTCAATCTCTGTTGACATTTATTCATATTGTTGACA
PV_Br_SNP_0651	A07	14974921	TCAGCTGAGGCGAGACCGTGTTGATAAGAAATCAGAGGAAGCTACTTTCATCAGCACGGACAACATCAGGTTATCTGGTAGTGTGAAGTTCGAGGTATG(T/C)GACAAAGAGGAGCTTGTTTTGTCTGGGACGTTGGAGATGTCTGGTAGTAATGGCTTCACAGGGGAGTCTGCTAAGCACAGTGTGAAGCGGTGGGGGATGA
PV_Br_SNP_0653	A07	16550579	TGTCCCGGTTCATTTCCCCAGACCAAAACCTGTGGCCGAGACCAAAAAAGAACATTACCAGAGTTACTTCAGTTTAAACATTTCTAGAGATCTGCCAGG(G/T)GTTATGACATAGAAGACTTGCCAGGGCAGTTCCTGCAAGTGCGACAAGTGCAGAGAGGAAGTTGAAGAAGAGTGCTTTTGATACAGTGAAGCCTGACCTC
PV_Br_SNP_0656	A07	16780120	GAAATGTTATTCTGGATAGAAAAGTGGTTGATATCAATGGTGATGACGACGATAGCGAAGAAGAAGAGAAGAAGCTGCAACTTAGGCGTCCAGTTTGGT(T/C)ATCTCTATCGTGTAAAGCTAGTAGGAAACGCAAAGGCTGAGTCAAGGATCGCAATCTGATGAAACTCTTGCTTGAAATGATTTCTTGCCATGATTTAGAT
PV_Br_SNP_0657	A07	16977664	CAACCACGGTTCCTCGGTTTGGTGATACCTCCTCGATGCCACTGGCAGATTCTGGATTTCAGAACACTTTGCAAGAAACTACAGAGGTAGCAGCACATG(G/C)GGGGGTTGAACACACAAATAACTTTGTTAAGGTGCTGTGTTTTTTGTCTGACTTTTCCAATCCATCTCGTTTGTTTTTATTGTAAAATTAATATAGTGAT
PV_Br_SNP_0663	A07	17756345	GTCACTAGAATTGCAATGTACATTCAGAGTTTGCTTGTTGCTTACAACGTTGTGGATTACGACATGCTTTTTTGCTAGAGGCCAGAAGCCGTCACCGCC(T/A)GATCCTGGTCCTGGTGCTTCCGCCGGAGAATCTGAGACATCTAACGCTGCAACAAAAAGAGTAAGAATGAAAAATGAGACCATAAACATCAATATGCAAT
PV_Br_SNP_0664	A07	17798150	GAAGGCAAAGAGTTGAAATGTTTGCATTTGTACCATACGAGCTGCATTGTGTCTTGGTTGAATATTCATAATACTTGTCCCATTTGTCGCTTTGAGGTT(C/T)GCTTAGGCGTTTCGGGAAGTGACATTGACGGAGGAGGGTCTCGCCATGTCGACCATGATAGATCAAATCGGTCGTCTGGGCCTAGGGTTTGTTCTTTGTG
PV_Br_SNP_0665	A07	17919819	GCCCAGCGGTTAGGATTGAAGGGTTCGATTGTCTCGACAGGGACAGTAGAAACTGCAGCATCCCTGCCTTGGCATGAGAACCCCAACCTCAAAAACCAC(C/T)AATCACATACTCTGTTGAATTAGCTCACATAAACCAATTAGTGCGTCGTACATAATGAGTTAAGTATATGGAGAATTTGTGGAAATCTTTGAGGGAAGTT
PV_Br_SNP_0666	A07	18314620	GTGTGGTTGAAGGATGTGGTGAGGTTGGATGCTTGAGAACGGCCAAGTCAGTTCATGGAATGATAACGAGGAAGATGTTTGATTTTGATGAAACGTTAT(G/T)CAACTCTCTGCTTTCTATGTATAGTAAGTGTGGGGACTTGCTTAGCGCGGAGAGGATCTTTGAATTGATAGTTAATAAGAGTGCTGTTTCGTGGACTGCT
PV_Br_SNP_0677	A07	20018101	TCAAACCAAGCCAGAAGATGGGAAATTGTTAGCCATTGGTCACTCAATGGGTGGTATGTTGTTATACGCCATGCTCTCAAGATGTGGTAAGCCTTCTCA(T/A)ATACATCATAAAAGACTAATATTCATTCTCTTATGTGTTGTAAATGTAATTCAAGATGTGTTAAGCCTTCTCATATACACCACTTTTTTTCTTCACCAG
PV_Br_SNP_1046	A07	20143408	AGAACGGTACAAAGCTCTAATCTCCACGTTTACTCACGAGAAAGGCTGGAGGCAGAAAGAAGCCTATATCGAGTACGGTGGTAGCTGGTGGACACAATT(T/C)GTTCTCGAAGGCTGCCTTCACGCGCAAGAATTCTTTAAAGCACGACCCAACCACTTCTTCGTCTGTAGCTACCCAAAGACAGGTACCACTTGGCTCAAAG
PV_Br_SNP_0678	A07	20498940	CCACCAAACATTCGCTCTCGTCAGAGAATCCTCTCTCTCTGACCCCGTCAAGGGCAAAATCGTCCAGAACTTCAAAGATCTCGGCGTTACAATATTATA(T/C)GTACGTAAAAAATCTTCTCTATAATGTTCTATGAAATGAGCAGATCCATGATGTGTATTAGTCAACCATATGTTATACGCAGGGAGACTTAAGCGACAAG
PV_Br_SNP_0687	A07	22175659	AGGTTATTGTACGTAGACATGTCGTTTGTGTGACTAACATTAACATGACGAGAAAAGACCCGTAGATTACTTTATTCATCAACTTCTGATTCTTAAACA(A/C)ACACTAACGTGAAAAGACAAGTATAAACACATACTTTACATTATAGAAACAAGAGATTTAGCCAGTGCCAGTAACACCAAGATCCTGCTTCTTGTCGTCC
PV_Br_SNP_0688	A07	22214476	CAGGGCGGCCAAAACAGAGAACGCATCAGCTTCAGCAATTGATATCAGATGAAAAGGTATCAATTACTTTTGCAATTGTACAGGTACAAAATCAACTAC(A/G)CAAACGTTGTCATGCCAACCTGTATTCAGGTGCAGATGTCTGATTTGACCGGTGAGTTCTTCCAAATTGCTGAATCGCACGATCAGCACTCCAAGGAAGC
PV_Br_SNP_0691	A07	22577772	CAAAGCTCGACATCTTCTCCTCTATCGTAGCCCTGGGTGTACTGAGGGATCTGTTTGAGGTCGATGCGGTTGCCCACAAAAAAACTCTTCTTGTCGGAA(A/T)GATCGCCTGTTCCATACCCACTTAATTGAATCATCTCTAAAATTCATTAACAAACATTGAAGCATACAGGTAGCCAGTATATCTTACAAAGGGCGTCTTC

Marker ID	Linkage	Position*	Flanking Sequence (100bp flanking the mutation site, and the SNP site is in brackts)
PV_Br_SNP_0696	A08	368385	ATGTTCACCGTTTTACCTAGAGTGTGTTGAATGCACGTGGTTTTTGGGTCTTAAGTGTGCTATGTTACCAAATATTGCTTACTACAAATTTGATAGGCA (C/T) CCACTCACACTTTGTTACGGAGAAAAAGGTACAAGAAGTGGTCAATACTGGTGTGAGCTATGTGAAACAACATTACATTCAAGTGAATGGTTCTATACAT
PV_Br_SNP_0697	A08	481952	TTGGAGATTTTGGTTTAGCGCGGTGGCAGCCTGATGGAGAGCTCGGTGTAGACACACGCATAATAGGAACTTTCGGGTAAAATCTAAATCAACATTCTC (T/C) ATCTCAAAAGTTCTACAGCTCCTACATTACTTACTATGCTTGGTTTGTTTAGCTACTTAGCTCCAGAGTATACTCAAAGTGGTCAGATCACAGAGAAAGC
PV_Br_SNP_0698	A08	734955	TGAAGGTGACATGGAGGAAAGTCATCAAGGACCTCTCATGTTCAAATTTCAGCAACCATGGTTCGTTTTTTGTGAAAGTTTGGTTGCCTTTGTTTCATG (C/T) GTCTGTGGTTGGCTAGTAAGGGCCTCGAGGATTGGTTGATCGTGGTCTCTTCAGAAACTGTTCACAGAGGCTTCACTCACCAGATATTAGCCTATTTTGA
PV_Br_SNP_0699	A08	912720	GTCAGAAATCGTATGATGAGACACTCGGTGTAAGGTTCCAACAGATATTGCTGGGAAACAGTTAGTCGGTGAGATGAACAGACTCATCGTTTCTTCGGC (G/A) TTGACGAACTTTTTCGTGTTATTAGTTACGACGGAGAATCTTAGACGATGAGGATGAATGGCCTACCGTTCAATTAAGGCTGAAAAATATCAATCGACAA
PV_Br_SNP_0709	A08	3109891	GAGCTAGCAGAGGAAATCCTGGTATTGAGACTGAGCCTAACTGGGGGAAGTGATTCCTATTTTTGGTACCCTGTCGCATCTGGAGCTTACTCAGAAAAA (T/A) CTGGCTACGCGGCAGCATCTGAATTTGCAATAGAAAACATTCCACAACATGAGATCATAACGCCTATCAACGGAAGAAATCTGTGCGGAATGTGGAGTGC
PV_Br_SNP_0710	A08	3527840	GAGAACAAGACCTTACCGAAATTACGACAACAATGGCCGATGTGAAAGAAGATATCGCAGTGAGCACCAAAGACACGGGACCCTCGTCCATAAAACTTC (C/G) AATGCTAACTTCGTCAAACTACACGGTTTGGGCAATGCGGATGAAGATAGCTCTCAAGGTCAATAAAGTTTGGGAAACCATTGATCCAGGCAACAAGCAT
PV_Br_SNP_0714	A08	3798695	GTATGCAGAACCTGATGTAGTTGAAGAGCTCTTTGAAGAGGGGTCTGCTATGCTTTCAGAGAGGTTTGATTCTCTGAGAACAATAGTTAATACAGAATA (C/A) TATATTTTCATTCATTTTGATATGTGAAACTTAACTTCGTTCTTGTTGTTTATTGCAGATTAGACACGAAGTATCCACTTTGCAATATGTTTAAGTTGTT
PV_Br_SNP_0715	A08	4073494	CTCCAGGTACCTTAAACGATATTAATGTCCTCGATCGATCTCCTGTTGTTGATGACATTTTCCAAGGCCGAGCTCCCAGGTTAAAGTACGTGGTCAACA (G/A) AAACCTGTATAAGTTGGCGTACTACCTCACAGATGGTATATATCCCAAATGGTCAACATTTATCCAATCTATCTCGCACCCTCAAGGTCCTAAAGCAGAG
PV_Br_SNP_1066	A08	5049303	TACTAAATGCTATTTTTTCTAAGTGAAACTATTATTATTTCCTTTCTTTTGTTAGGTCAGCCTTTTTTGGTTTATAGGTTTACTTTTGATGAAAGATTT (T/A) AAAATAACTTTGTCCCAATTTCAACAGGATATTGTTGTACCGGGAACTGACACTGCGGCAGCAGTGGTAGTATGGGCCATGACTTACCTTATAAAGTACC
PV_Br_SNP_0721	A08	6333089	TTCTTCCATTAAGAAAGTCTCCCTCAACTGTATAAACTATGTTCATCCAGATCATTTGGACCGTTGGATATGTAACGTGCTGCGTCGTGGTGTTTCAGA (A/C) CTTGAAACTGCCATCTATGATCAAGATGGTGATGATGATTTTAATTATCTTCTGCCTCAAGAGATGTTCGTCAGTAGGACATTAGTCAAGTTGAAATTAA
PV_Br_SNP_0722	A08	6547066	GATGACAGTCACCGCATATTTTTTTCGCTGCTCGATGATTTATCACATGAAACCAGAGACTTTCTCTCTCTCCAACCGATAAAAGAAAGCAACAACCAA (C/T) TCATATATTATTTAAATCGTTATTGATCATCTGCAAAACAGTTGTGGTGCAGGAGATGGACGACTTGCCGATTCGTTGCGTGGGATATCTAGCAGTGAGG
PV_Br_SNP_1061	A08	6622868	GCTAATTACATATGGCCTTACTTGAACTGAACTATAGAAACATTAGTGAAACAATGTAAAACCAAGTTGTGATCGATGATGAGTTACCGGAAGCATAAC (G/A) TAGACGGGAACATAGTTGAGAAGAAGCTTTTCGTTGTAGTTTGCAGCCATTTTTAAAGCTTTTTAGGAATGAATAAGCAGTAAGTGATACTAGTTGCTGA
PV_Br_SNP_0725	A08	6780592	CCTGACGGTGACTTTACCTGATACGTCACAAACGTCATCTCCTGGCCGTTAACGTCACCTTTAATTGAGGGAGTGCATGATTTTAAAAGCTCAAGCAAC (G/A) AGACGTTAACTCCTCCTATATTTTTGTTGCCTAGGACACGGCGACAGGTCAATCTCATGGCGAGAGTCAAACGACTGTCACGAGCTAATCTCTCGTCGAC
PV_Br_SNP_0729	A08	7381378	ACCCTGTTGCAATGTGGCATGTCCGCGAGGAGATTGCTTTATCCATTCTACTAGTTGTGGATTTTGGAGGATGGTTCAGGCAAGATTCTAAGTCTTTCA (A/G) CGGTACTAGCTTCCCTGAGAGGATAGAGCATACTCAAGTCTCTGTCATTAAGGATGTGATTGTTCCATACACGCATCTTCTTCCTAGGCTAGATTTGTCT
PV_Br_SNP_0743	A08	8978452	CTCTCCAACCATTTTTTAAAATAGTTATCTCTTCACAAATATGGATCATCTCTTTGAGAGAGTTCTCCCACATATGGATGATCACCAGTTTGCATGGAT (G/A) CTGTGTACATCTGGAAATGAAGGAATAATAAAGTCTTTAGTAATTTAGACATCGTTCCTAGAAAAACGCTTAAATTAGCAGAAACAGAATCCACACTTTG
PV_Br_SNP_0744	A08	9046309	TTGATTGTTAGCTTCCCTGCATTGCTCTTTTGACTGAAGAAATAAAGTTTTGTTTTTTTCTCTTCTCTAATTGCAGAAATTCTCAGCGTTTCTGCTTCT (G/A) CAGCTTTCATTTCAGAGTCTAGGCGTTGTGTATGGAGACTTAGGAACCTCTCCTCTATATGTTTTCTACAATACATTTCCCCACGGGATCAAAGACCCTG
PV_Br_SNP_0745	A08	9356818	TGAATTCCATTTGTTATACTTTAAAACTAATATGTAACACCGTTTGACTTTCTAACATCGATTATGTGAAGGGATACAACTGTATGTGTTAGTATGTTT (A/G) ACTCCTTGGCTCTTGCATTCCATAGCAAGCTCGATGGCTACAGGAGAGAGCCAAGGATTGTTATTGTCACCGGCGTCAATCCTAAGATGGTTTCGGGTAA
PV_Br_SNP_0746	A08	10076323	GTGCCAGTAGAACTGGCATTAGAGTTAGAGGTAGCTGGTTATCAATCTGTAAATTGCCCAGAATGACTTTGAACCTGTAAACCATAGAAAAGAGTTGCT (A/T) GTTCAGATTTCCGAAGGTATTTCATGCGGTTGCATCGCTTCCTAATACGAGAGAACATATGACACTGAAAAATTTACACGAATGTGCAACCTAAAGACCA
PV_Br_SNP_0754	A08	11236942	GCAAGGAGCTTCCTGAGCTGGCGTTAAAGAAGGAGAACTGTACGGAGATGACTTGGTTTCAGTCTGCTCTATGGTGGGACAATCGCCTCAACGCAACTC (T/A) GATTGATCCTAAAGTGTTTCTTGATCGGAATCTTGATTCCTCTAGGTTCTTGAAGAGGAAGTCGGATTACGTCGCGACGGTGATTCCTAGAGATGGGATT
PV_Br_SNP_0760	A08	12781945	GCGAGTAATTCGCAGTTTCGGGGATACTATCCCATTCTTCAGCAGAAACATTATCTAGCTTCCTCTTCAAATCCGAAAACTGCTCGCAGATTTTCGGAT (T/A) CGAAGCTCTGTAATTCTCGATTTCATCTTTAATTTTTGCTTCTTTAACATCTTTCCTTCTCGAATCCATTCTCTTATCAATAGCTTCCCATATCGCATCA
PV_Br_SNP_0761	A08	13413655	TCTTCAACTAATTGACCACGACTTGAGTCAGATCAAAAAAACAATATTTTAACTTCAGATAGTGCAAAAAATCAAAAAGAGAACAACAAATTGAGAATG (T/C) AATATCCGTACCTCGTCAGAGAGTGAGCGAGAAGTCCAGTAGAATAAGTCTTCAACATCTCAGAATCTGAGGCCTCACTGCTACCAGAGTGTTCACCAGG
PV_Br_SNP_0762	A08	13487463	CATTATTCTCAAACATTAGATTGAGCGGTTATAGGTTAACAATGTCATAAGAGATCTTGTTACCTCTTACTACCATTCGTTGAACCTCGGTTCACCCAC (G/A) TATTAAAAACCGTAATGCAAGTGAAGCTATAGTTCTTGAATCATCACAGTTTTTCCTCTCAGATGTTTTCCATTTCATTGGACTAGATGCAGATATTAAA
PV_Br_SNP_1062	A08	13615880	CTTCCCACTGTTCCTCCTCTTACTCTGGCTATGATCTAAAGGAGATCGGAAATTTTCTGGTTAGATGAGTCTCGTAATCTCAATCGAAAATATTATATT (C/A) AGTGGTTAGTGAGTTTCATACTAGTAGATCCTATAACTTTTATTGACATGCTTCTTCACTAGTTCACTCGTAAATATATTTCACCTACTTATCGGAAACA
PV_Br_SNP_1072	A08	15753089	AAGAAGTCTCGCGAGCATCATCGTCGTCAACGCCGTACCCAAAGTTGGTGCAGCACAACCTCTTTTACCGGTACTAAACGAGATAAACCGGAGATCGTT (T/C) TCCGTCAATGTAACTTCGGAGCACTCGTTGAGATGTCTCTCCGGTTTAAAGCTAAGTGGGTCGGCCCAAACTTTTGGATTACGGCCCAGCCCATATCGGC
PV_Br_SNP_0776	A08	15885956	ATTACTTGCCCTTGTCAGAGGACCAAGTTGCAGCTAAACTTGCGGCTAACAACAAGTGATCTTGGTTGCTTCTTGTGGTGATTTCATGTCATTGTTTTC (G/T) TAGCAGGCTTCTTATGGAATTTAGCTCAGTGCGGACAAAAGTTGAGGACTTGGTTTGGCTATTATCTAGTGTTAGAACGCAGGAGAAGACTTTTATGTTT
PV_Br_SNP_0778	A08	16910347	GAAAGTGCAAACGTGTGAGAAGATTCCCCATTTCACAGCTTCATAAAAGCCTTTCTCTGATGGTTCATCGATGGGTTCATAAGTGTCGTCGTGTGAGAG (G/T) TGGAGACTTGGAGGACACGTAGAGAGGTGTTGCAAGTGGATTGCTAGATGATCTCCTTTCTTCCCCTCAAGGAATAAACGTAGTCCCGTTACTGGTCTCT
PV_Br_SNP_0779	A08	16953278	AGATCCTCTCTCCGAGCATCCTCGATTTCCCGTCTCTCGTACTCAGCCCGGTTACTCCTCTTGTACCCGACCCGTTTAACCGGTCCGGATCATCGAATC (A/G) GAGCTCCAACGACGAGGAAGAGAAGGCTATGAAAGAGAAGGGGTTTATTTGCACCCGTCTCCGGCGACAACTCCGATGGATTCTGAGCCTCGTCTTCTT
PV_Br_SNP_0783	A08	17556729	CAACATGGTGCTCGCAGCACTGGTGGAAGCCAAGCGGTTGCGAATCGCTGAGGAACACGGGCTCATTGACAAACCGAACGTTACCATTCCAATGTCCAT (T/A) TGGTGGTTCGTCCCTCAGTATATGTTGGTCGGTATGATCGAAGTGTTTGACCTGGTAGGTGCACAAGAGTTCTTCTACGACCAGGTCCCCACAGAGCTGA
PV_Br_SNP_1041	A08	19024222	TCAGTCTGCAGCTTTTGCCACATCTTTTGAGTCCTAAATCATTTTACAAAATAATTTTATATTAAAAATCATTGATCAATAATGACCAGAAAGGCCAAA (C/A) ATTGAATATTCAAATAATGCGGAATAAAAAAAAATACCAGCTTTTTCCGGGAGAGTTCGCCATCCACCTTCACCGTGACAATTGACGTAGGAGACAAGCT
PV_Br_SNP_1037	A08	20713105	AATATTTCAGAAACAAAAAGCAAATGAAAAAAAAAAACAGAGGAAGCTAGCATGCAATGTGGCTCCGCCACCTCCGATCAAGAACCCAGTTACGAGAAC (G/A) TAGTTCTGACGGAGACGAAACGAAGCTGCAACACAAACAAGCAACCAAATACCAAACTAACAAAACAAAACTAAAATAGTCTTTCGATTCAACTGCGGAT
PV_Br_SNP_0790	A08	20974107	CGCATCTTCCTGATTCAAAGGATCAGATACAGGTTGAATAACGCTCTTATCAGTAGGAGCCAAACTCAAACTAGTATCATCCTGAGGTTCAGCTTCAGT (C/A) AATGCTTCCATAAGAGGCTTGTCAATCACCTTCTCAGAAGGTGCTTCAGCTGAGTCTTTAGCCACACTGGACACAGGACTCTTCTGAGACTCTTGAGCTA
PV_Br_SNP_0792	A08	21364188	CACTCTTCGAATCTCCCACTCAGAAGCTTTAAACTCCAATCTCTGATCATGGACCTGAAAGCAATCAACTAAATGGTTACCGAGTCTCATCCATGGAAG (C/T) ATTTGTACATGTGGATGTTGTGGTTAAGAAGTTAGAAGCAGTGTCAACTTACAACTAAAATGGTGGTTCGAGTGGTTCATCCGCACTCCCATTTGAGACT
PV_Br_SNP_0795	A09	489884	CAGATAAGGCTGTTATTCTTGACCATGGCACAGATGTATTTATTTGGTTGGTAAGTTTCTCTTGTCTTCATAAGGTTCATGCAATAATATATATTTGTC (A/G) GAAGCGTTTTTGTTAATCTTTGTTGGTTTAGGGAGCGGAACTGTCTGCTGATGAGGTGAAGAGCGCTGCGGTTTTAGCAGCTTGCAGAACGTTGGCTGAA
PV_Br_SNP_0796	A09	731769	AATACTGACCCTCCCATCTCTCTCAGGTTCTTTCCTCTTCCAACCCCTTCCTATACTCGGTGGCATCCTCTCCATCTGACAAGACCCTGTTGCTGGTCT (G/A) TACGTAACCCTCTGCGGTCCGTTTCTGAGAACCTCCTCCTCCTCAGCTTTGACTCGTTCCATCAACATCCAGACCGGTAAAAACTCCTCTTCCATCAACC
PV_Br_SNP_0798	A09	966111	AGTTTCTTGCTTCACAAGGAACTCGTTTAAACTTGTTTTATTTACTTCATGAACAATAAAAGATTTCAGAAGACCATAGTCTGCTTCAACACATCAAGT (C/T) TTGCTTGTTGGAGACTTCAAAACCTGATTGCGCTTCATACACCTTCTCTGCTCGCCGAGACAGCGATGATGAGAAGTATCAAACTAAAATCAAGACCAGT
PV_Br_SNP_0800	A09	1112513	AAACACCTCCCCGGTTCCAGTGACTGATCTGGTGACTATTCTCGAGAGGTTGTTGAAAGTCAAGGCAAAGAGGAACATTGTGAAGATGCCTAGGAATGG (C/G) GATGTGCCCTTCACTCATGCTAACATCAGTTCCGCGCAAAGAGAGCTCGGTTACAAGCCCACCACCGATCTTCAGACTGGTCTTAAGAAGTTTGCCAGAT
PV_Br_SNP_1083	A09	2867279	TAGAACTTGATTTCTTAAAACGTTTCTTGCGACTCAAGGAATCGTCCTCATGAACTGGTGTCCCATCTTTGCTGGTATTCTCGAAAACCGTGTTGAAAC (C/A) GGAGGAAGGAGGACAAGACATGGTAGGCATTGAGCTTGACCGGGAGTATTGCTTGTCGGAACTAGATGCATCTAGGGAATGCAAATTCTCAGGTACTGTA
PV_Br_SNP_0809	A09	4162713	TTCATATCTATAGCACGAAATATGTAAACGCACACTCATGTTAACTGTATGCAGACATGGTTAAGGGTGTCTCAGAAGCTTCACACGACCAGTCAAGTT (A/G) TGGTTGGTGCGATCGTGGGGTCTGTTTTCTCCACCTTATGGTACATCACTTGGAACTCACTTGTTCTCCAAGCTTTTGCCTCGTCACAGATAGCCGTCTT
PV_Br_SNP_0810	A09	4305557	GAAGACTTACATGTTCAGAATCTCAAATGTTGGGTTGTCAAGCACTTTCAACTTCAGAATCCAAGGACATACGATGAAAGTAGTTGAAGTTGAAGGCTC (T/G) CACGTCATGCAAACGGACTACGACTCTCTTGATGTTCACGTTGGCCAGTCTTTATCACTCCTTGTGACGTTAAACCAATCTCCTAAAGATTATTACATCG
PV_Br_SNP_0811	A09	4460206	CTCTGCATCTTCATCCCCCGTCTCAGCCGTTACACTCTCATCCTGTGTCGCTGATAGTTCTGTCATTTCTATCTCTTCCTGACTTTCCTTCCCCTCCTC (T/A) ATATCAGCTAATGAGGAGTCTACATGCGTTTCCGCATCAGTCATGTTTTCATCTTTCTCCTCATCCTCATCTTCGGTCCTTGCCTCTGTTTCTTCTGACA
PV_Br_SNP_1071	A09	4475177	CGCCGGGAACGGAACCGGAGGAGAGTCGATCTACGGCATGAAGTTCAAAGACGAGAACTTTGTCAAGAAGCACACCGGTCCGGGTATTCTCTCCATGGC (T/C) AACGCTGGTTCGAACACGAACGGATCTCAGTTTTTCATTTGCACTGAGAAGACATCGTGGCTCGATGGGAAGCATGTTGTGTTCGGTCAAGTTGTCGAAG
PV_Br_SNP_0812	A09	4959336	CATTTTCAAAATCCCAAACGCTAATATCATTGAAGGAATCGCTGCGAGTCCAAGCATCAATCTCCAGCCGAGTTTCAACGTCAATTTCCCGAAGAAGTA (G/A) TTCGAAACATAGCCTAGTAAGATCCCTAGACTAATACAAAGCTCGGGGAGCGAAGTGAGAAAGCCTCTGTGTGAAGCAGAGGCTATCTCGGCGGAGTAGA
PV_Br_SNP_1049	A09	5177858	AACGTTTTGATTTATGTTGCAGTATGTGCCTTATGTTGGGGATAGTAAGAGAGCCATGGACGAGTACACCTCGGAGATATTCATGGGAGGGAAGAATAC (G/A) ATTGTTATGCATAACACTTGTGAGGATTCTCTATTGGCTGCTCCAATCATCTTGGATCTTGTTCTTCTCGCTGAGCTCAGCACCAGAATCCAATTCAAAG
PV_Br_SNP_1044	A09	7789464	TTCAAGAAATGGAAACTTATACTTGTACCTGGACAGTAAACCAAGATATAAGAAATATAATGAATCCATCCACATTTTACATATGATATCTCGCTATAT (T/A) TAACCATAAAATATTGAAAATGATTAAAGAACATACCCGGTACAATACACAACAGTATCTGCATGAACCGCCTTCCCATTTTTGAAAACAATTGAACCAT
PV_Br_SNP_1045	A09	7789468	AGAAATGGAAACTTATACTTGTACCTGGACAGTAAACCAAGATATAAGAAATATAATGAATCCATCCACATTTTACATATGATATCTCGCTATATTTAA (G/C) CATAAAATATTGAAAATGATTAAAGAACATACCCGGTACAATACACAACAGTATCTGCATGAACCGCCTTCCCATTTTTGAAAACAATTGAACCATCATC
PV_Br_SNP_0826	A09	8726675	GACGGAACTCGCTAGAAACATGGAAGTGATGAAGAAAGTTCAAGGCGAGATCCGTGACCGCCTTGGAAACAGTAAGGAGAGAGTCACCGAAGAAGATAT (C/A) GGCAAGGTTCCTTACTTGTACCTCGTGATCATGGAAACATTCAGGTTGCACCCAGCACTTCCACTTCTGCTTCCAAGGGAAACTATGACTCACATCAAGG
PV_Br_SNP_0827	A09	9563894	GAGAAAGTTTTGTGGCAGATTTATTTCATCGCCTCCTGCTAATTTCCAAGCAATGGTGTTGTTATGTGCTCAGTTCCAGGGTTCTCATTCTTCTCAGGT (G/C) AAGGTTCTCTTCAGGTTACTCTTACAGGTGATTGTATATAACATATGGCGTGAACGAAATGCTCGTATCTTCCGTAATCTCTCGCGTTCTCCTTTGGCTT
PV_Br_SNP_0828	A09	9652821	CTCCATCTTGAGAGGAGCGCGCAGAAGGAACATGAGCGTGAGAAAGAGGCAAGGCTCCGCAAACCCTAGATTCGAGACGGTGTACCACTTGCAGACGTT (C/T) TCTTGCCATCTGAGATCCAACCCGCTCAGTATCCTCCTGTGATACCGAAACAAACTCAAACGGAGAATCTCTCCGACAGCCCAGCAGATAGCGAAGAGGA
PV_Br_SNP_0839	A09	11475911	TATACTTCTCTACATACGTTCCAGTGGGTATGTATGTAAACATGAACTTTGAGAGAAGAATGTGATCAAACTCTAGTTCTTGTATTACTGATAATGTGC (T/A) TGTTGTGGTTTGCAGATCGCCTGTTGTGAAGATACAACCTTCTAAAACACTCATTGGACAACCAGTTGGTACAAAGGTGGCAGAGTTCTCATCAAGAGGA
PV_Br_SNP_0848	A09	13502218	AGGATTGGGATGTTAAGCTGTCGGAAACCACCGGGCGGTCTAAAAGGAGACAAGGGAAGAGGTTTATACGTAGATCGAGATTGTAGCATCTGAGTTGTG (C/T) GTACCATTTGTGTTTCTTCAACATACTTTTGCAGTCGAATTGTATTTGTTTTTTGCCCTCGCAGAGACTCTAAATAAAATCAATAAAATAAGATCCATTT
PV_Br_SNP_0850	A09	15420007	AAAAGGCTGAATTTTTGGACGCTGCCGCAAGACGTGGCGGTTCTGAAACGAACAGGGCTCATGTCGATAAATATTGGTTTGACAAATATACATCATTTT (T/C) AGTGAAGATTTATAGATGGGTTGTGGTCGAACAAATAGTTAGAATGCAATAAAATATTTTTTAGTTTGTTGTAGTATTTTTTTTTGACGAAAATTTTGCT
PV_Br_SNP_0851	A09	15638572	ACGTATGTATCTACTTTTACAGGCAATGTACCAACCAAAACAGTCGTGGAATCATCTTGCTAATATGCCTATATGCAGCTTTAACAACAACTGAGCTCA (T/G) TACTAATTGGCCCAAAAACTAATTGTCAGAGAAAGTATCCCATCACTTCTGGCCTAACCCAGTTGATAAGAAAGCGAATATTTCGGCCATATTTAACACA
PV_Br_SNP_0855	A09	17812533	TATGTGGGATAAGTCTTTGAGTGTGGTAATGGGATCCTTGGAGTCGCACACATTTCGATACGCCTTGAGAAATGTCCTCTCCAGGTTCTCGGACAGACC (C/T) TTGGGCTTATCTGCGTACTCTCGCTTCTTCATCAACACGTGATCCGCTAATCCTTGGTTCTCTCGACACACCACCCTTCTCTCCTCATCCATCACTACCC
PV_Br_SNP_0865	A09	19518904	GTTCTTGGAACAAAACGTAACGCTCTCGATCTTCTGGATCGCACCACAGTTTCTTATATTCGGTTTATCAGAGATGTTTACAGCCGTTGGTTTGGTGGA (G/A) TTTTTTTACAAACAGTCTTCACAGAGTATGCAGTCTTTTCTCACAGCAATGACTTACTGTTCTTACTCTTTCGGGTTCTACCTTAGCTCTGTTCTTGTCT
PV_Br_SNP_0867	A09	21654202	TGTGATTCAAATTTGTTTCGTAGCTGGTCATCAATGAGATCGACGAAGCGTCGGCTACTCCTCTTCAGGCAGTGAAGCTCCTTGCTATGTACCTTTCGA (G/C) CCCTGAAAACAAGGTAATGCTTGAGAATTTGAGATTATCACTTACGCAATCGCATCGATTCAATTCATGTTAGCTTAGCGGAATTGAAGATATCTTCGTG
PV_Br_SNP_0868	A09	22147801	CCAGCTGTAAGCATACTCTCTAACTCGAAGTAGTTTCCTGATTGCATAGGAGTGTGATTGCTTTTCCTTGATTGTCCAGAAATTACTCTTGCAATCTCT (G/A) AGTATGTGTTTTGTAAACCAGGCTACCCATATAGATCCTGATCGAAAGAACAGGAGCCAGACAAGCTTGAGAGTGCATGCTTTGTTCCAGAGGTGGAGAT
PV_Br_SNP_0869	A09	22335708	AACCTCTGAGATCTCATCATGAGGCAAGGTTAGGCCCTTGCGTTTTATCGACCTATGCAACTCACAGAAGAATTTAGGGTCTTGCAGCTTGATGTACTC (C/G) TGCAAAACTTGCTCATGGTTTGGTATCCAGTTCCTAGGAAATGGAGTATAATCACAAGATGGAATAAAAGACATGCGAGAGAACTGAACTCCATCTATAG
PV_Br_SNP_0879	A09	25766864	TGTGTCGGTTCAAAGCAACGGGATCATCACTGGCTTCTGTGTTGATGTCTTTGTGGCCGCTCTCAGTTTACTACCTTATGCAGTTCCCTATGAGCTTGT (A/G) GCTTTGGGGGATGGTCATGATAATCCAAGTAACTCTGAGCTAGTGCGCTTGATAACTGCTGGGGTGAGTGTACATATGATATGCACTCTTTTTAGAATCT
PV_Br_SNP_0882	A09	26952124	AAAAACTTCGCAATGACAAAATCCTCCCACAGATGATTAGATTTTTCAATGATCTCCGATGGAACTTCCACTGATTGCTTTCCCTCTGAAACCTCAATG (C/T) CGAAATCATACTTCTTCAGAACTTGTTTCTTCTGCACCGCCGAGACCCAGGATCTTTTCTGATCGATCGGAGGTGGCGGATCTTCCTGTATCGCCGTTGG
PV_Br_SNP_0883	A09	27132534	TCTTGCTGAAGCCAAACAAGAAGTGGAAGCTAAAGAAGAGCCTACAGAAGAAGTGAAGCAGTGTGTCGAAAAAGAAGAACCGCAAGAAGCTAAAGAAGA (G/C) AAGACTGAGAAAAAACAACAACAACAAGAAGTGGAGGAGGCGGTGGTCACTTGCTGCATTGATTCTTCGGAGAGCAATGAGCTGGCTTGGGACTTCTGTA
PV_Br_SNP_0889	A09	27875429	ATGACTTGACTTGAGTTCACACACAGGAGAATGTGTTCACTGCTAGACTAGAGAGTGTGACAACTTATTTTTATATGAGCAGGGTCTTAAGTCAAGCTC (T/A) ATCTTTGGAGATTCACTCAGAGTAGCACCAAGATCGCAGTTGAAAGCCACCAAGGCTAAGAGCAATGGTGCTTCATCTGTGACCAAATGTGAAATTGGTC
PV_Br_SNP_0902	A09	32116439	TGCGTTTTGAAGCGAGAGCTTTGTTACTTCATCAGCTAGATTTTTCAGCTCAATTGCAGCTGCAGAGGCCAATTCCTTAGCGTACGAAGCTTCTTCAGC (A/T) AGTTTTTGATTTTGCACTCGTAATCCACTTGTCTCCTCCACAGTCTGCACATGCTCTAACTTCAACTTTTCATTCTCTATCTCCTGAAGTATAAAAACAT
PV_Br_SNP_0904	A09	32560861	CTTCAAGTAAAGGGATGCCATACAGCGAAAGCATCCCTTTTTTAGACAAAACCGGGTGCTCAGGCGACGCACTGCCTTATACCAACACAGGGGACTGTA (G/A) AATTACCGAAGATGATGAAGAAACAAGCTGCAATGGTAACGGATGCTGCCGTGCTAGTCTACCGAATGATCCTCAACAGGTGATCGGCGTCAGAATAGAG
PV_Br_SNP_0906	A09	33028118	GATGCCACGATGATTGAAGAAATAGTTCAAAACATTTCAAGCCGGTTGTTATCTATGTTGCCAATACGTTTCCGTGATGTTGTCGGGATGAGAGCTCAC (G/A) TGAAAGTTCTCAGCCCTCTCTTGGACATGGACTCCAAGGATGATGCTCGTATCATAGGAATCGTTGGAACAGGAGGCATTGGTAAAACCACTATAGCAAA
PV_Br_SNP_0907	A09	33253584	GCGGTTTGATAGAAACCCACTTGCCATCTTTCTTGATTTGGAGACCTTCAACTTCATTCACCTGCAACAATATGGTGAGGGCCACCGCATCCGAATGAG (G/A) AGTTAGACCAATAACCTGATGAGGGTCCGGACAGGGAGGATAGTAATTCATCCTCATGCTCTGTAGCATATCATCACCAAATAAATCTTCATTTTCCTCA
PV_Br_SNP_1038	A09	35628753	GTAGTAGCATCCATCTGCAAAACATTCAAAACACAGATTGGTTAGCAACAAATCAAAAGGGTATAACCACAGCAAGTAATGAAAAAAGAACAGCTAGTT (C/A) ACCTTGTTACAGCAGCAAATCATCTGCTTGACACCAAGGGTGAAAGCAAGAAGAGCATGCTCACGGGTCTGACCATCCTTGGAGATACCAGCTTCAAAAC
PV_Br_SNP_0918	A09	36554283	AAGAAACTTACTTTCTCAAGTTTCTCAAAGGTGCTTGCTCGACGTGGAGCCCAGTCTACTAACGCCTCACCAGCAACAGAGACTAGCCAAGTAGGCCAT (G/T) CTTGCTTCAACTCTATGTCTCTTGAAACAATAACACTCTGGTTCTTTTTCACTTCCCAAGGAACAGCTTGAACCGAAGCTTCAGCCCCAAGATTCTCTGG
PV_Br_SNP_0919	A09	36612655	CCTCTTGGGGTATTCTTCAATCAATTTTCAGATCCCTGTCGTTGTTGTTTTTTCCCTGTTATTCTCTCTCACCCCTTTCTCGTTCTTCATGAATTGTAA (T/C) TGAACAGGCTGCGTGGAACTTCTGTTGGAGATAATTTACCCCTTTCTTCCGGGTTCATCTCCATTTCTTTCTTATGCTTTGAAGATTGTGAAAATTCTTG
PV_Br_SNP_0920	A09	37061247	TGGTCGTAAAGTGTATATATGACATATATGCTAGGTTCCCAGTAAAACTGGTGGGCCTCCGGAGAAAAAGGAGATAGTGCTTGAAGATCATGATCCAGT (G/T) TGGCTTGAGCTTCGACACACACATATAGCAGATGTATATGTTACACAGTTACTTACAAAGTATCTTCCCCGCAGAATAGCTCACCATTTTTTTGATTTG

Marker ID	Linkage	Position*	Flanking Sequence (100bp flanking the mutation site, and the SNP site is in brackts)
PV_Br_SNP_0921	A10	592464	GTCATTACAGGTGTTGAGAGATCTTGGTGGCAGTTGCTTCAGCCAGTAAGTTGCATCCCTAGATAAAGAGTGTTCAAAGAGGTTACAGAACAAAATAGT(C/T)TTCAGAGACTTCATTGCACTTGATGCTTGATGTTAGATCCTCGAACCTCTCAAGATTATCCATAGGGTTCTCAAGGAGAAGACCATGAAAAGGATGCTGA
PV_Br_SNP_0924	A10	1550777	GAAAAGGAGACAAATGTTTGATGTTAAAGACGTTTGTAGTACGCAGATTAGGAGGTAATTTGATACGATAGGCATTAGGGTTGATGCGCTCAAGGACCT(A/C)GACCAGACCAATCTTTTTTTACTTTAGTTTGTTGTATTCACGCAATGGCAGACGTTCCTTCGTTAAGTACACCCAAACGAGATCCCCGGGTTCGAATATA
PV_Br_SNP_1067	A10	3312620	CGCATCACCTGTTATTGTTTTCGAGAAGAGGAATTTATATAGGCAACCAACAGGGCATAGTACAATTAAGATTAGCAGGGAAGAAGAATATTGGACCTT(G/C)CAAGATGTAACAGTGCCATAAGGTGTAAAAAGCTCCTTCAGTTTCTCGTCAGAGATACTGTCATCTAAATTCGTGACATACAAGTTAGAGCTTTGAAACT
PV_Br_SNP_1068	A10	3313214	AGCCATAGGTTTCTGGGCAGATTCATCAGTTTCATATTGCATAAACCCATAGCCTTTCGATTGCCCTGAGGAATCCACAGCTACCTTGCACGAGATAAT(G/A)TTCCCGAACGCAGAAAACGTATCATGCAACGCTTTGTGGTCTATGGACTTGTCAAGATTCTGCATATATATAACCAAGTTTAGCACAGGAAAAAAATAGA
PV_Br_SNP_0931	A10	3904244	TACTTGGACTGAGACTGGGTGAAGAAACAAAGCTCTGTGGGCTTAGGCGCCCTGTTTTCATGGCCATGGCACTGTTGGAAGCATATGCAAGCCACCTTG(G/T)ACCTACAGCCATCGGACCATAGCCAACATTGACCCTAGTTGTCCCTTGTCTCACTGGCTGAGGAACAGGATAGGTGAGAACACTGAACTTGTTTTCCAGA
PV_Br_SNP_0939	A10	6064111	TTTGAGGAGTCTTACGACCCATGGCATGAGCTGGTTGAGACGCAGGATGAAAGAGGTAGGTACAAAACATCCTAGTGCTTAGCAAAGCAAGCCAGGCCT(G/A)AAACCCATTGTTGGTTTGACAGGTGGTTGAAGGAGAAGCTCAAGTACATAGATGCCAAGCGGATATAGATATGCATGAAGAAGATCATCCTATGTATCCA
PV_Br_SNP_0951	A10	9526129	GCTTCCACCTCCTGGCTTGGTAGAAGAACTCGAGAACAGAAAAGGAAGAAAGCCAAAACCAAAGAGAAAGAAAGGAAAAAATGAGTCACCAACAAAGAA(G/A)AAGGTCAATCGAAGTAGGAGAGTGATGCATTGTGGTCGATGTGGCGTTGCTGGACACAATGTAACCAAGTGCCCAAATGTAGGCGTGCCAGTGAATAGAC
PV_Br_SNP_0952	A10	9623892	CTCTTAGCTCATGATTTCTATTCTGAATCCGATCAGTTCCGGGTTGTGCAATTACCGGACTACCCTGATTACAACAAGGACTACAAACGAACGTTAACA(C/A)CATCAGGAGGCTTTGTCGTGTATGTCAGAACACTAGCAAAACATGACGAAACTGTTTTGAAGATTTGGAGGCTGAATAATGATGATGATACTTGGCAAAT
PV_Br_SNP_0954	A10	10451842	CTTAGGTGTACTTAACAATTTTCTGTTGCCGGATTCAAATGAAGAAACTGAGGACGAGTTGTCAGATGAAGAATTTGACTTTGATAAAGTTGAAGGTGC(G/A)TAAGTGGTTCAGTTATAAATCTTAGTTGTTTATACTGATCACTCGTTATATAACGGTCTTTTTTTTCTTGCCAGGTGAAGCCTGTTCACGAGGTGAACTG
PV_Br_SNP_0955	A10	10551658	TCTTTTGTTCTGAAGAAGAGGCGGCCTATCACGCTTCTGTAGCCTCAACCAGGAGAGGTCGGTCTAGAACAGTACACCACGTCCCAATCTGCAGCAAAA(G/T)ATTCAGCAAGTTCACGTTTTGATAAAAGGAAGTAGCTTTCGCAGTGTTTGCTTAGATTATTTCTTACCTCCGAAAATGAGAGGGCACTTAACAGGCTCGC
PV_Br_SNP_0958	A10	11191128	ACAACAACAAAAGCAATGTGTAAATAGGAACTTGTCTTTTTCTGGCCACGGGTCAGGTTCTTGGGGATATAAGTTCGACACGGTTCGTGGGTTGGAGCT(T/G)GGTCTCGGTACGTCAAATGAGGCACGTGGTTCGTGGTCGGGAGGTTTTAGTTTTCTACAAGAAGATGATTATCATAATTCTAATCGGTACGTATCTTCAA
PV_Br_SNP_0959	A10	11287063	TGTATCTGGTTCGCAGTGGAGGCCTGGGAGTCCCTTTCAGAGTCAGAGTGAAACGGTGCGTGTTTTGAATTAGAATATAATCCAAAGGATCTCCAGGAC(C/T)TTTGAACTATATTAAGTTGGTATTCTGACCAATATCGTGGCTTTGCTTGTTAGGTTTTCTCCTATTTTAGAACATACTTCAGAAAAAAAAATCCCGGTTT
PV_Br_SNP_0960	A10	11370494	CAACCAAAGATTCACTAATTTCTAGCGCTCTGGTATCTACAGCCTCTTTCACTTCTTCATGAATCTCTTCCAGGTCTTCTTGACTAGAAACAGACAAAC(A/G)GTCATCCTCCTGGTGATCAATATTCTCGTTCTTATCCAAAGGTAGAGAGGAATCAAGTAAGGCTTCCTCAAATTCAGACACGGAAATTGAGTTCTTTTGA
PV_Br_SNP_0962	A10	11690581	GAGCCCTTCTAAACCCAAATGCTGCACTCCTTGTCTAGACTTTAACTTAGCTCTCTCTTCCAACGAAAGCGAATCCCATTCAGGCTTCCACACGCAGTG(T/C)GTCCCTGGACCGTATGTCTCCGTCAAGCCGTAAAGATGAGACACATTGAATCCCAACTCTTCCATCTTAGCCAATATCTGCGGCATAGGCGGGGATCCTC
PV_Br_SNP_0966	A10	11950431	CTCTCAGAGCCCTTGCTCGTTCTGGCATGAAGATTGGTCGTATTGGTAAGTAAATCTCTCTCTGCCTCTTTTGATTATACAGAGAACTGTGCGGTTTGG(C/T)TAAGAATAATCGTGTTATTGAATGTATTTTGCAGAGGATGTGACTCCGGTTCCCACCGACAGTACACGTAGAAAGGGTGGACGAAGAGGAAGAAGACTCT
PV_Br_SNP_0968	A10	12106839	TCCGCGCATCAAAGAAGCACAAAAGTGTGATGTTCGTGGCTCGCTTGCCCTTCCTGTGTTTGAAAGATGTAGTGGCACTTGTTTGGGTGTTGTTGAGGT(T/C)GTCACAACAACGCAGAAGATGAATTACCGGCCAGAACTTGAGAATATCTGTAAAGCTCTTGAGGTTCTACTCTTCCTCCTAAATCATACCTTGTCTTGAA
PV_Br_SNP_0969	A10	12179004	GCCATGATACGCCTGTCTCAAGGTCTCTCGACCTGACTGGTGCGTCTCCTCGCACCATGGCCCGTCCGGAAGATCCTATCTAGGATCGGGGACATGACA(T/G)TGGAACCCAATATATTAAGTGCATGAGCAACCTTTATCTTTTCAAATGTGAGCTATCTTCCTCCTAGTGCTGCCTGATGTGACATAAGATTTAACCAGAT
PV_Br_SNP_0970	A10	12295861	ATTACTTTTGTAGATTTTTGGCGTTGGAGTTATGGACTTTGATGAGTATGAGTACTTAGAGAAGACCGTTGAGAATCCTCACCTGGAGAACAACGAAGT(C/T)GAAAATGGTGGGGGTGATGAGAAACCCAAATCTGAAGTGAAAGAGCGGAGTAGAAGCTCAAGGCATAGGAATGATGAAGACGAAGATGGTCGGCGTTCTA
PV_Br_SNP_0974	A10	13246082	AATCGACCTCTTACTAAATCTTATTAGTACTTTTTGTTTTCTTTTTCACACGGATGATCTATGTGCTAATATTGGTGTACGATCTTGTTTTATAATCAT(C/A)TACTTTCCGTACATAACAGGTTCTCTGCATCCGGAAGCCTGAGGAAGGTATTGATCTGTTTATGGTGGAGATAATGCACATGCGCCACAAATTCGATGTT
PV_Br_SNP_0975	A10	13638161	CTCACCGCAAGGTATCGTTGATTTACAACAAGATGATAACTTTCGGTGTTTCACCTGACGTCTTTGCTACCAATTCGTTGATTCATTCGTTCTGCAAAG(T/C)GGGTCGATTGAGATTAGCTGTTAGTTTACTTAGAAGTAGAGTAGTCAGCGTCGATACTGTTACTTACAACACTGTGATTCAAGGCTTATGCCAACACGGT
PV_Br_SNP_0976	A10	13973332	TCTACGTGGTCGAGGAGCCGGATGCTTTGATATCCATGACTTCAACGACCTGCATATGTTTGTAAATCTCTAATGCTATGCACCAATAATAGCTATGTG(G/A)ACAAAATAAAAAGGCACAACTACTCACGCAAGAAAAGGTGCCACCATATACAACTTGAGACCAGCAACTCCATGAGTTATCACCTCATTACTTGCTGGCT
PV_Br_SNP_0980	A10	14708300	TTCTTCCTCTTATTCCTTTTTGTGTGTTAAGAGCTTTCGAGGTTGAAGACAAAGGACCAAGAATGGTACTTCTTCTGTCCTCTGGAGAAGAGGCAAAAC(A/T)GCTCTACGGTTATAAACCGAGCAACGAAGGAAGGCTACTGGAAGAAAACTGGAGATGACAAGAAGATCAAGCGAGAAGGAGACGATGAGTTGATCGGTGT
PV_Br_SNP_0985	A10	15707133	ACTAAACATGTAGATATTTTTACCAGATAAGGCTACTAGATCATTTGTATTTAGCCCGACAGCTGAAAATTTTGATGTGATGTTAGTAAGGCTTTCTAC(T/G)GGAGAAGGAATTGACGAATTTGCCCCGGCTTGGTTTGCTGTGAGACTATCTCTTCTGCCCAATAATACAGTCCATGAAGGCCCTCCTGACTGACCATGTT
PV_Br_SNP_0986	A10	15817352	TTGAAAAACTTTGCGCCTTTTCCCCCAATACCAGCACCTGCAAGGCTTGAAAAGAGATGCGATCTGGTGTCTGGTCTCCACTGCAACTCTACCAAGTCC(G/C)AGAACAAAGCTGACATCAACGGAGATATCTCTGAATCTTTTACGTTCTTGCAAGAAACCACTTTCAGATGCTCGAGCTCGTGCCACTGTAGAATCACCGA
PV_Br_SNP_0987	A10	15853544	ATCGCCGTTTCTGCCTCTTGCCACCGTCGTCCTCACCGGTCTATACGCATACCTGCACCTATGTTTTCCCTCAGATTGCCGGAGCTGCTCCGGCGCATG(A/G)GCAAAGAAACACACTTTCCTCTGGCACATGTTTCCGGCGTTGCATGCGCGCGTCCGGTAACGCGCCGGGTGGAGCCAGTACTCGAACACACCGTGTGCGA
PV_Br_SNP_0989	A10	16000038	ATAATTCACCAGAACTTGCCAACTATTCAATGTTAAAATCAATACCACAAGCCCGATCACTGTCACTAACGCCACACCTCATCTATTGTTATGATAAAA(T/C)GACAATACAAACATTTCAAGACTGAAAATGATAATAACTGAGCTGGTCTAGTTTAGGCTAGTCTTGAATGCGTGGAAAGTGGTCTTGATACATATTAGAA
PV_Br_SNP_0996	A10	16818623	GAGCCTTTAGTCACGGCCATAAACAAACCTAAGAGTTTTTCAAACCTCGAGGGTCATCAACAATAACAAGAGAAAGCCCACAAAAGACTAAAACGAGAG(A/G)CATTTCGTAGCGTTTCAACTAAACATGGGCTTATAGACTAGATAGAGAGATAGTGAGACCTACTTACAAGTCACTCCAGGACTTTTATTACCATCCGGA
PV_Br_SNP_0998	A10	17237508	GGGGAACCGGTTGTAGAATCTGTCTTCAGCTAGCCTTATGTAAGCTGCATCTTTCTCAACTAAGAGTATGAACATAGCATCACTCTGCATGTCTCCAAC(T/G)CGATCGATGTTCGGTGGAATGGCTTTCCCACCCATCCCCATCTTTGTGCAGTCTATCATGTCTCCGTTGTCACTGAATATGAGCCTTCCCACCACCACAC
PV_Br_SNP_0999	A10	17368173	ATCAAGGCACTTGTAATAGAGGACAAGTCTAAGAAGAAAGGTCGTCACCACAGAAGAAGCTCTACTTATCCTGTCCAATCAAACCCAAAAGAGAAGGAC(T/C)CATTGAGTGAGTTGGTGGAATCTTCTGATAAGAACTCCTCAAACGGTGATGAGCGTAACCGAGTCTTTAACCAGACTATTGGCATCTCACCCGCTATTGG
PV_Br_SNP_1053	Scaffold(	8568	GTCATTTTGATTCTGTATTGAAACATTTTAAAGTAACATCCTCTATTTTCTGTCTCCAAACAGAGGAGAATGGAGCAGGACCAAATCCAAACGTGGGTG(G/A)AGCAGATCACTTACATCAACATCAAGAGATCTCATCACGTGATCACAAAGTCTCAGTTTCAAGTACGGTGAATGGTCTAAGAGATCGTATTCCATCTTCA
PV_Br_SNP_1054	Scaffold(	9355	CGGTCCGGGTCGGGTATAAAAACAATGATGCTATGTTACTTCAGATACCAATGTGTGGCGTTTTAATGTCTTCAAAACGATGTGGGGATTCTCTCAAGT(G/C)CTCCCTGTTGCTACATTCAAAGACCCGGCAAACGGATACCTCTACGATGGAGACCACTGCGAGTTTGGTGTTGATGTGACCATTCCCACTACGTATCAAA

* Refering to Wang et al.(2011) and http://brassicadb.org/brad/
