# Supplementary material for: Morphology, Carbohydrate Composition and Vernalization Response in a Genetically Diverse Collection of Asian and European Turnips (Brassica rapa subsp. rapa)
Source: PLoS One. 2014 Dec 4;9(12):e114241. doi: 10.1371/journal.pone.0114241 (PMC4256417; doi:10.1371/journal.pone.0114241)
Supplement: Table S2 — Experimental design and performing period for the five independent experiments. (PDF) [file pone.0114241.s009.pdf]

Table S2. Experimental design and performing period for the five independent experiments

Experiment	# Accessions	# blocks	# plots /accession/ block	# plants / plot	# plants / accession	Condition	Sowing	Taproot harvesting	Plant Age (day)
2010G	42	4	1	1	4	greenhouse	9/Feb/2010	26/May/2010	106
2008F	41	4	1	20	80	open field	20/May/2008	5/Sep/2008	107
2009F	42	2	2	5	20	open field	10/May/2009	18/Aug/2009	100
2011F	29	2	2	5	20	open field	22/Jun/2011	26/Oct/2011	126
2012F	53	3	1	3	9	open field	20/Jul/2012	15/Oct/2012	75
