# Supplementary material for: Morphology, Carbohydrate Composition and Vernalization Response in a Genetically Diverse Collection of Asian and European Turnips (Brassica rapa subsp. rapa)
Source: PLoS One. 2014 Dec 4;9(12):e114241. doi: 10.1371/journal.pone.0114241 (PMC4256417; doi:10.1371/journal.pone.0114241)
Supplement: Table S3 — Comparison of stele and xylem areas in the six turnip accessions. Numbers indicate mean ± standard deviation. (PDF) [file pone.0114241.s010.pdf]

Table S3. Comparison of stele and xylem areas in the six turnip accessions. Numbers indicate mean ± standard deviation.

Section position	Accession	Stele diameter (μm)	Pith ratio	Xylem ratio	Phloem ratio	Lignification ratio	Xylem / Phloem
a	VT_115	2047.88 ± 156.93	0.04 ± 0.01	0.74 ± 0.03	0.22 ± 0.03	0.04 ± 0.01	3.43 ± 0.52
5mm under	VT_117	1282.19 ± 64.85	0.05 ± 0.01	0.68 ± 0.01	0.27 ± 0.01	0.11 ± 0.02	2.53 ± 0.17
cotyledon	VT_012	2270.07 ± 174.66	0.03 ± 0	0.65 ± 0.05	0.32 ± 0.05	0.07 ± 0.01	2.08 ± 0.5
	VT_053	734.36 ± 66.82	0.05 ± 0.01	0.7 ± 0.03	0.25 ± 0.02	0.29 ± 0.03	2.89 ± 0.39
	VT_052	800.36 ± 86.03	0.16 ± 0.02	0.55 ± 0.02	0.29 ± 0.03	0.1 ± 0.01	1.89 ± 0.23
	VT_123	1668.69 ± 132.32	0.06 ± 0.01	0.63 ± 0.03	0.31 ± 0.04	0.01 ± 0	2.08 ± 0.37
b	VT_115	2109.54 ± 159.88	0.03 ± 0.01	0.78 ± 0.04	0.2 ± 0.03	0.03 ± 0.02	4.06 ± 0.84
middle position	VT_117	1298.63 ± 193.13	0.03 ± 0.01	0.7 ± 0.03	0.26 ± 0.02	0.1 ± 0.02	2.7 ± 0.39
	VT_012	2163.14 ± 135.17	0.03 ± 0	0.62 ± 0.02	0.35 ± 0.02	0.05 ± 0.01	1.74 ± 0.13
	VT_053	842.82 ± 49.2	0.04 ± 0.01	0.72 ± 0.01	0.24 ± 0.01	0.15 ± 0.03	3.01 ± 0.18
	VT_052	870.69 ± 109.59	0.03 ± 0.01	0.66 ± 0.03	0.31 ± 0.03	0.08 ± 0.01	2.2 ± 0.33
	VT_123	1418.42 ± 383.51	0 ± 0	0.65 ± 0.12	0.35 ± 0.12	0.04 ± 0.02	2.09 ± 0.79
c	VT_115	1902.95 ± 142.7	0.05 ± 0.01	0.75 ± 0.04	0.2 ± 0.04	0.03 ± 0.01	3.87 ± 0.86
5mm above	VT_117	1120.67 ± 130.99	0.03 ± 0.01	0.7 ± 0.01	0.26 ± 0.01	0.1 ± 0.01	2.66 ± 0.12
the bottom	VT_012	2169.04 ± 213.47	0 ± 0	0.69 ± 0.03	0.31 ± 0.03	0.01 ± 0.01	2.23 ± 0.31
	VT_053	765.46 ± 50.71	0.04 ± 0	0.68 ± 0.03	0.29 ± 0.03	0.04 ± 0.01	2.4 ± 0.35
	VT_052	856.34 ± 182.92	0 ± 0	0.69 ± 0.06	0.31 ± 0.06	0 ± 0	2.37 ± 0.62
	VT_123	1556.45 ± 147.11	0 ± 0	0.7 ± 0.02	0.3 ± 0.02	0.03 ± 0	2.35 ± 0.23
