# Supplementary material for: Morphology, Carbohydrate Composition and Vernalization Response in a Genetically Diverse Collection of Asian and European Turnips (Brassica rapa subsp. rapa)
Source: PLoS One. 2014 Dec 4;9(12):e114241. doi: 10.1371/journal.pone.0114241 (PMC4256417; doi:10.1371/journal.pone.0114241)
Supplement: Table S4 — Results of genotyping 56 turnip accessions using 280 SNP markers. (PDF) [file pone.0114241.s011.pdf]

Table S4 Results of genotyping 56 turnip accessions using 280 SNP markers

SNP marker ID	Chromosome	Physical position (bp)	Polymorphic site	Accessions																																																			
				T_1050V	T_1283V	T_163V	T_307V	T_385V	T_738V	T_821V	T_826V	T_830V	FT_001	FT_002	FT_003	FT_004	FT_005	FT_047	FT_051	FT_056	FT_086	FT_088	FT_097	SM_14	SM_15	SM_16	SM_17	SM_18	SM_19	SM_20	T_panca	T_RR	VT_006	VT_007	VT_008	VT_009	VT_010	VT_011	VT_012	VT_013	VT_014	VT_015	VT_017	VT_018	VT_044	VT_045	VT_052	VT_053	VT_089	VT_090	VT_091	VT_092	VT_115	VT_116	VT_117	VT_119	VT_120	VT_123	VT_137
PV_Br_SNP_0002	A01	191796	G/A	AA	AA	AA	AA	AA	AA	AA	AA	AA	AA	AA	AA	AA	AA	AA	AA	AA	AA	AA	AA	NN	NN	NN	NN	NN	NN	NN	NN	NN	AA	AA	AA	AA	AA	AA	AA	AA	AA	AA	AA	AA	AA	AA	AA	AA	AA	AA	AA	AA	AA	AA	AA	AA	NN	AA	AA
PV_Br_SNP_0003	A01	198417	C/T	TT	TT	TT	NN	TT	TT	NN	TT	TT	TT	TT	TT	CT	NN	CT	CT	TT	TT	TT	NN	TT	CT	CT	TT	TT	TT	TT	CT	TT	CC	CC	TT	TT	TT	TT	CT	TT	TT	CT	TT	TT	TT	TT	TT	TT	TT	NN	NN	TT	NN	CT	TT	TT	TT	TT	TT
PV_Br_SNP_0005	A01	207316	A/G	GG	GG	GG	GG	GG	GG	NN	GG	GG	GG	GG	GG	GG	GG	GG	GG	GG	GG	GG	NN	GG	GG	AG	GG	GG	GG	GG	AG	GG	GG	GG	GG	GG	GG	GG	GG	GG	GG	GG	GG	GG	GG	GG	GG	GG	GG	NN	GG	GG	GG	GG	GG	GG	GG	GG	GG
PV_Br_SNP_1048	A01	332469	A/G	GG	GG	GG	GG	GG	GG	GG	GG	GG	GG	GG	GG	GG	GG	GG	GG	GG	GG	GG	GG	NN	NN	NN	NN	NN	NN	NN	NN	NN	GG	GG	GG	GG	GG	GG	GG	GG	GG	GG	GG	GG	GG	GG	GG	GG	GG	GG	GG	GG	GG	GG	GG	NN	NN	GG	GG
PV_Br_SNP_0006	A01	511520	C/G	GG	GC	CC	CC	CC	CC	NN	GC	GC	CC	CC	CC	CC	CC	CC	GC	CC	NN	GC	NN	CC	CC	CC	GC	CC	GC	CC	CC	CC	GG	NN	CC	CC	CC	CC	GC	CC	CC	GC	CC	CC	CC	GC	CC	CC	CC	NN	GG	GG	CC	CC	CC	CC	CC	CC	GG
PV_Br_SNP_0014	A01	2609884	G/A	AA	GG	GA	GA	AA	GA	GG	GA	GG	AA	GG	GA	AA	GA	AA	GG	GG	GA	GA	GA	NN	NN	NN	NN	NN	NN	NN	NN	NN	AA	GA	AA	AA	GA	GG	AA	AA	GA	AA	AA	GG	AA	AA	AA	GA	AA	GA	AA	GG	GG	AA	GA	GA	NN	GA	GA
PV_Br_SNP_0015	A01	2867228	C/T	TT	TT	TT	TT	TT	TT	NN	TT	TT	TT	TT	TT	TT	TT	TT	TT	TT	TT	TT	NN	TT	TT	TT	TT	TT	TT	TT	TT	TT	CC	NN	TT	CT	TT	TT	TT	TT	TT	TT	TT	TT	TT	TT	TT	TT	TT	NN	TT	TT	TT	TT	CT	TT	TT	TT	TT
PV_Br_SNP_0016	A01	3049926	A/C	CC	CC	CC	CC	CC	CC	NN	CC	CC	CC	CC	CC	CC	CC	CC	CC	CC	CC	CC	NN	CC	CC	CC	CC	CC	CC	CC	CC	CC	CC	CC	CC	CC	CC	CC	CC	CC	CC	CC	CC	CC	CC	CC	CC	CC	CC	NN	CC	CC	CC	CC	CC	CC	CC	NN	CC
PV_Br_SNP_1064	A01	3360507	A/T	AA	AA	TT	AA	AA	AT	TT	AT	AT	AT	AT	TT	AA	AA	AA	AA	AA	AT	AA	AT	NN	NN	NN	NN	NN	NN	NN	NN	NN	AA	AT	AA	AT	AA	AA	AA	TT	AA	AA	TT	AT	TT	AA	AT	AA	AT	AA	AA	AT	AA	AT	TT	NN	NN	AA	TT
PV_Br_SNP_0017	A01	4993191	A/G	NN	NN	GG	NN	NN	GG	NN	NN	NN	NN	NN	NN	NN	NN	NN	NN	GG	GG	NN	NN	NN	NN	GG	GG	GG	NN	GG	NN	GG	AA	AA	NN	AA	NN	NN	GG	NN	GG	NN	GG	NN	NN	NN	NN	NN	NN	NN	NN	NN	NN	GG	GG	NN	GG	NN	NN
PV_Br_SNP_0033	A01	8788567	C/T	TT	TT	TC	TT	TT	TT	NN	TT	TT	TT	TT	TT	TT	TT	TT	TT	TC	NN	TT	NN	TT	TT	TT	TT	TT	TT	TT	CC	TC	TT	TT	TT	TT	TC	TT	TT	TT	TT	TT	TT	TT	TT	TT	TT	TT	TT	NN	TT	TT	CC	CC	CC	TC	TT	NN	TT
PV_Br_SNP_0035	A01	9304705	A/G	GG	GG	AA	GG	GG	GG	NN	GG	GG	GG	GG	GG	GG	GG	GG	AG	AG	GG	GG	NN	GG	GG	GG	GG	GG	GG	GG	GG	GG	GG	AG	GG	GG	GG	AG	GG	GG	GG	GG	GG	GG	GG	AG	GG	GG	GG	NN	GG	GG	AA	GG	AG	GG	GG	GG	GG
PV_Br_SNP_0037	A01	9618547	A/G	AA	AA	GA	AA	AA	AA	NN	AA	AA	AA	AA	AA	AA	AA	AA	AA	AA	AA	AA	NN	AA	AA	AA	AA	AA	AA	AA	AA	AA	AA	GA	AA	GA	AA	AA	AA	AA	AA	AA	AA	AA	AA	AA	AA	AA	AA	NN	AA	AA	AA	AA	AA	AA	AA	AA	AA
PV_Br_SNP_0055	A01	14045839	A/G	GG	GG	NN	GG	GG	AG	GG	AG	GG	GG	GG	GG	GG	GG	AG	GG	GG	GG	NN	GG	NN	NN	NN	NN	NN	NN	NN	NN	NN	GG	GG	GG	GG	GG	GG	GG	GG	AG	AG	GG	NN	GG	AG	GG	GG	GG	AG	AG	GG	GG	GG	AG	GG	NN	GG	GG
PV_Br_SNP_0058	A01	14246865	T/C	CC	CT	CT	TT	CT	CC	NN	TT	TT	TT	TT	TT	CT	TT	CC	CT	TT	CT	CC	NN	TT	CT	TT	TT	TT	CC	TT	TT	CT	NN	CT	CC	TT	TT	TT	TT	TT	TT	CT	CC	CT	TT	TT	CT	TT	TT	NN	TT	TT	CT	CC	TT	CT	CT	TT	CT
PV_Br_SNP_0059	A01	14336973	T/C	CC	CC	CT	CC	CC	CC	NN	CC	CC	CC	CC	CC	CC	CC	CC	CC	TT	CC	CC	NN	TT	CC	CC	TT	CC	CC	TT	TT	CT	CC	CT	CC	TT	CC	CC	TT	TT	CC	CC	CC	CC	CC	CC	CT	CC	CC	NN	CC	CC	CT	CC	TT	CC	CC	TT	CC
PV_Br_SNP_0060	A01	14415476	T/C	TT	TT	CC	TT	TT	TT	NN	TT	TT	TT	TT	TT	TT	TT	TT	TT	CC	TT	TT	NN	TT	TT	TT	TC	TT	TT	CC	CC	TC	TT	TC	TT	CC	TT	TT	TT	TT	TT	TT	TT	TT	TT	TT	TC	TC	TT	NN	TT	TT	CC	CC	TC	TT	TT	NN	TT
PV_Br_SNP_1055	A01	15365144	T/C	TT	TT	CC	TT	CC	TT	NN	CC	TT	TT	TT	TT	CC	TT	TT	TT	CC	CT	TT	NN	TT	TT	TT	TT	TT	TT	TT	CC	CC	TT	TT	TT	CC	TT	TT	TT	TT	TT	TT	TT	TT	CC	TT	TT	TT	TT	NN	TT	TT	CC	TT	CC	TT	TT	CC	TT
PV_Br_SNP_0080	A01	16957132	T/G	TT	TT	TT	TT	GT	TT	TT	TT	TT	TT	TT	TT	GG	TT	TT	TT	TT	TT	TT	TT	NN	NN	NN	NN	NN	NN	NN	NN	NN	TT	TT	TT	TT	TT	GT	GT	TT	TT	TT	GT	TT	TT	TT	TT	TT	TT	TT	TT	TT	TT	GT	TT	GT	NN	TT	TT
PV_Br_SNP_0084	A01	18014692	A/G	GG	GG	GG	GG	GG	NN	NN	GG	GG	GG	GG	NN	GG	GG	GG	AG	GG	GG	GG	NN	GG	GG	GG	GG	GG	GG	GG	GG	GG	AG	AG	GG	GG	GG	GG	GG	GG	GG	GG	GG	GG	GG	GG	GG	GG	GG	NN	GG	GG	GG	GG	GG	GG	GG	GG	AG
PV_Br_SNP_0086	A01	18706064	A/G	AG	GG	AA	NN	GG	GG	NN	GG	AG	GG	GG	GG	GG	GG	AG	GG	GG	GG	AG	NN	GG	GG	AA	GG	AG	GG	GG	GG	AG	AG	GG	GG	AA	AG	AA	AG	AG	GG	GG	GG	GG	AG	GG	GG	GG	GG	NN	GG	AG	AG	GG	GG	GG	GG	GG	AG
PV_Br_SNP_0089	A01	19150724	T/G	TT	TT	GT	TT	TT	NN	NN	TT	TT	TT	TT	TT	TT	TT	TT	GT	TT	TT	TT	NN	TT	TT	TT	TT	TT	TT	TT	GG	TT	TT	TT	TT	TT	TT	TT	GT	TT	TT	TT	TT	TT	TT	TT	TT	TT	TT	NN	TT	TT	TT	TT	GT	TT	TT	TT	TT
PV_Br_SNP_0098	A01	21243792	A/T	AA	AT	AT	AA	AA	AA	AT	AT	AT	AT	AA	AA	AA	AA	AA	AT	AA	AA	AA	AT	NN	NN	NN	NN	NN	NN	NN	NN	NN	AA	AT	AT	TT	AA	AA	AA	TT	TT	AA	AA	AA	AA	AT	TT	AT	AA	AT	AA	AA	TT	TT	TT	AA	NN	AT	AT
PV_Br_SNP_0101	A01	22000107	A/G	AA	AA	GG	AG	AG	AA	NN	GG	GG	AG	AG	GG	GG	AG	AG	AG	GG	AG	AG	NN	GG	AA	GG	AA	AA	AA	AA	GG	GG	GG	AG	AG	GG	GG	NN	GG	AG	AG	AA	AG	AG	GG	AA	AG	GG	AA	NN	GG	AA	GG	AA	AG	AG	GG	GG	AG
PV_Br_SNP_0104	A01	23766969	T/C	TT	TT	CT	TT	TT	NN	NN	TT	TT	TT	TT	NN	TT	TT	TT	TT	TT	NN	TT	NN	TT	TT	TT	TT	TT	TT	TT	TT	CT	TT	TT	TT	TT	TT	TT	TT	TT	NN	TT	NN	TT	TT	TT	TT	TT	TT	NN	TT	TT	TT	TT	CT	TT	TT	NN	CT
PV_Br_SNP_0108	A01	25004591	T/C	TT	CC	NN	CC	CC	CC	NN	CC	CC	CC	CC	CC	CC	CC	CC	CC	CC	CC	CC	NN	CC	CC	CC	CC	NN	CC	CC	CC	CC	TT	CC	CC	CC	CC	TC	CC	CC	TT	CC	CC	CC	CC	CC	CC	CC	TC	NN	CC	CC	TT	CC	CC	CC	CC	CC	TC
PV_Br_SNP_1029	A01	25145190	T/G	GG	GG	GG	GG	GG	GG	NN	GG	GG	GG	GG	GG	GG	GG	GG	GG	GG	GG	GG	NN	GG	GG	GG	GG	GG	GG	GG	GG	GG	GG	GG	GG	GG	GG	GG	GG	GG	GG	GG	GG	GG	GG	GG	GG	GG	GG	NN	GG	GG	GG	GG	GG	GG	GG	GG	GG
PV_Br_SNP_1030	A01	25145360	A/G	GG	GG	GG	GG	GG	GG	NN	GG	GG	GG	GG	GG	GG	GG	GG	GG	GG	GG	GG	NN	GG	GG	GG	GG	GG	GG	GG	GG	GG	GG	AG	GG	GG	GG	GG	GG	GG	GG	GG	GG	GG	GG	GG	GG	GG	GG	NN	GG	GG	GG	GG	GG	GG	GG	GG	GG
PV_Br_SNP_0118	A01	28347266	C/G	CC	CC	CC	CC	GC	CC	CC	GC	CC	CC	CC	GC	CC	CC	CC	GC	CC	GC	CC	CC	NN	NN	NN	NN	NN	NN	NN	NN	NN	CC	CC	CC	GG	CC	CC	GG	GG	GC	GC	CC	CC	CC	GC	GC	CC	GC	CC	GC	GC	GG	GC	GC	GC	NN	CC	GC
PV_Br_SNP_0125	A02	488097	C/A	AA	AA	CC	AA	AA	AA	AA	AA	AA	AA	AA	AA	AA	AA	AA	CA	AA	AA	AA	CA	NN	NN	NN	NN	NN	NN	NN	NN	NN	AA	AA	AA	AA	AA	AA	AA	CA	AA	AA	AA	AA	AA	AA	AA	AA	AA	AA	AA	AA	AA	AA	CA	NN	NN	CC	CA
PV_Br_SNP_0128	A02	939293	C/G	GG	CC	CC	CC	CC	GG	NN	CG	CC	CC	CC	CG	CC	CC	CG	GG	CG	GG	CC	NN	CG	CG	CG	CC	CG	CC	CC	NN	CG	GG	CC	CG	GG	CC	CG	CC	GG	CG	GG	CG	CC	CC	CG	CG	CG	CC	NN	CC	CC	CG	GG	GG	CG	CC	NN	CG
PV_Br_SNP_0130	A02	3230130	T/G	GG	GG	NN	NN	GG	NN	NN	GG	GG	GG	GG	GG	NN	GG	GG	TG	GG	GG	GG	NN	GG	GG	GG	GG	NN	GG	GG	GG	TG	GG	NN	GG	GG	GG	GG	GG	GG	GG	GG	GG	GG	GG	GG	GG	NN	NN	NN	GG	GG	TT	GG	GG	GG	GG	NN	GG
PV_Br_SNP_0131	A02	3783084	T/C	CC	CC	CC	CC	CC	NN	NN	CC	CC	CC	CC	CC	CC	CC	CC	CC	CC	CC	CC	NN	CC	CC	TC	CC	CC	CC	CC	CC	CC	CC	CC	CC	TT	CC	CC	TC	TT	CC	CC	CC	CC	CC	CC	CC	CC	CC	NN	CC	NN	CC	CC	TT	CC	CC	TT	CC
PV_Br_SNP_1035	A02	5879777	C/T	CC	CT	CC	CT	CT	CC	CC	TT	TT	CT	TT	CT	TT	CT	CT	TT	CT	CT	CT	CT	NN	NN	NN	NN	NN	NN	NN	NN	NN	CT	TT	CT	TT	CT	CC	CT	CC	CT	TT	CT	CC	CT	TT	CC	CT	TT	CT	CT	TT	CT	TT	CT	CT	NN	CT	CT
PV_Br_SNP_0143	A02	6823179	T/G	TT	TT	TT	TT	TT	TT	TT	TT	TT	TT	TT	TT	TT	TT	TT	TT	TT	TT	TT	TT	NN	NN	NN	NN	NN	NN	NN	NN	NN	TT	TT	TT	TT	TT	TT	TT	TT	TT	TT	TT	TT	TT	TT	TT	TT	TT	TT	TT	TT	TT	TT	TT	TT	NN	TT	TT
PV_Br_SNP_0144	A02	6983274	C/G	GG	GG	GG	GG	GG	CC	NN	CC	GG	GG	GG	GG	CG	GG	GG	GG	GG	GG	GG	NN	GG	GG	GG	GG	GG	GG	GG	GG	GG	GG	GG	GG	CG	GG	GG	GG	GG	GG	GG	GG	GG	GG	GG	GG	GG	GG	NN	GG	GG	GG	GG	GG	CG	GG	GG	GG
PV_Br_SNP_0145	A02	7322569	C/G	CC	GG	CG	CG	CG	GG	NN	GG	GG	GG	GG	GG	CC	GG	CC	CG	GG	CG	GG	NN	GG	GG	GG	GG	CG	GG	GG	CC	CC	NN	CG	GG	CG	GG	CG	GG	CC	GG	GG	CC	CG	CG	CG	GG	GG	CG	NN	GG	GG	CG	GG	GG	GG	GG	NN	CG
PV_Br_SNP_0148	A02	7567322	A/C	CC	CC	CC	CC	CC	CC	NN	CC	CC	CC	CC	CC	CC	CC	CC	CC	CC	NN	CC	NN	CC	CC	CC	CC	CC	CC	CC	CC	AC	CC	CC	AC	CC	CC	CC	CC	AC	CC	CC	CC	CC	CC	CC	AC	CC	CC	NN	CC	CC	AC	CC	CC	CC	CC	NN	AC
PV_Br_SNP_0156	A02	8448254	C/G	NN	NN	CC	NN	CC	NN	CC	NN	CC	NN	CC	NN	CC	CC	CC	CC	CC	CC	CC	CC	NN	NN	NN	NN	NN	NN	NN	NN	NN	CC	CC	CC	CC	NN	CC	NN	CC	CC	CC	CC	CC	CC	CC	CC	CC	CC	CC	CC	CC	CC	CC	CC	CC	NN	CC	CC
PV_Br_SNP_0157	A02	8484688	A/T	TT	TT	AA	TT	TT	TT	NN	TT	TT	TT	TT	TT	TT	TT	TT	AT	TT	NN	TT	NN	TT	TT	AT	TT	TT	TT	TT	TT	TT	TT	AT	TT	AT	TT	TT	TT	AT	TT	TT	TT	TT	TT	TT	TT	TT	TT	NN	TT	TT	TT	TT	TT	TT	NN	TT	TT
PV_Br_SNP_0158	A02	8828680	A/G	GG	GG	GG	GG	GG	GG	NN	GG	GG	GG	GG	GG	GG	GG	GG	GG	NN	GG	GG	NN	GG	GG	GG	GG	GG	GG	GG	GG	GG	AG	AG	GG	GG	GG	GG	AG	GG	GG	GG	GG	GG	GG	GG	GG	GG	GG	NN	GG	GG	GG	GG	GG	GG	GG	GG	GG
PV_Br_SNP_0162	A02	9343146	A/G	GG	GG	GG	GG	GG	GG	NN	GG	GG	NN	NN	GG	GG	GG	GG	GG	GG	GG	GG	NN	GG	GG	GG	GG	GG	GG	GG	GG	GG	GG	GG	GG	GG	GG	NN	GG	GG	GG	GG	GG	NN	GG	GG	GG	GG	GG	NN	GG	NN	GG	GG	GG	GG	GG	GG	GG
PV_Br_SNP_0172	A02	13543301	A/G	GG	GG	AA	GG	GG	AA	GG	GG	GG	GG	GG	GG	GG	GG	GG	GG	GG	GG	GG	GG	NN	NN	NN	NN	NN	NN	NN	NN	NN	GG	GG	AG	AG	GG	GG	AA	GG	GG	GG	GG	GG	GG	GG	GG	GG	GG	GG	GG	GG	AG	GG	GG	NN	NN	AA	GG
PV_Br_SNP_0175	A02	14260292	A/G	GG	AG	AG	AG	AG	AG	NN	GG	GG	AG	AG	GG	AG	AG	AG	AG	AG	GG	AG	NN	GG	AG	GG	AG	GG	GG	AA	GG	GG	AG	AG	AG	GG	AG	GG	AG	GG	AG	AG	AG	AG	GG	GG	AG	NN	GG	NN	AG	AG	GG	GG	GG	GG	AG	AG	GG
PV_Br_SNP_0177	A02	14947720	T/C	CC	CC	NN	TC	CC	CC	NN	CC	CC	TC	TC	CC	CC	CC	TC	CC	NN	TT	TT	NN	CC	CC	CC	CC	TC	CC	TT	TT	NN	CC	NN	CC	TT	CC	CC	CC	CC	CC	CC	CC	CC	CC	CC	TC	CC	TC	NN	CC	CC	TC	CC	CC	CC	CC	TT	NN
PV_Br_SNP_0182	A02	16556107	T/T	TT	TT	TT	TT	TT	TT	NN	NN	NN	TT	TT	NN	NN	TT	TT	NN	TT	NN	NN	NN	TT	TT	TT	TT	TT	CT	TT	TT	TT	TT	TT	TT	TT	TT	NN	TT	TT	TT	NN	TT	NN	NN	NN	TT	TT	TT	NN	TT	NN	TT	TT	TT	NN	TT	NN	NN
PV_Br_SNP_0193	A02	21006740	A/C	AA	CC	NN	AA	AC	CC	CC	CC	AC	CC	AA	AA	AC	AA	CC	AA	AC	AC	CC	CC	NN	NN	NN	NN	NN	NN	NN	NN	NN	CC	AC	AC	CC	AC	AC	CC	CC	AA	AC	CC	AC	AC	CC	CC	CC	CC	CC	CC	AC	CC	CC	NN	NN	NN	CC	CC
PV_Br_SNP_0194	A02	21572911	A/G	AA	AA	AA	AA	AA	GA	NN	AA	NN	GG	GG	AA	AA	AA	AA	NN	GG	AA	GG	NN	GG	AA	GG	AA	AA	GG	AA	NN	AA	GA	GA	GG	NN	GG	AA	AA	AA	AA	NN	AA	AA	AA	GG	AA	AA	AA	NN	NN	GG	GA	AA	AA	AA	AA	AA	GG
PV_Br_SNP_0196	A02	22448961	T/C	CC	CC	CC	CC	CC	CC	NN	CC	CC	CC	CC	CC	CC	CC	NN	TC	CC	CC	CC	NN	CC	CC	CC	CC	CC	CC	CC	CC	CC	CC	CC	TT	CC	CC	CC	CC	CC	CC	CC	CC	CC	CC	CC	CC	CC	CC	NN	CC	CC	TC	CC	CC	CC	CC	CC	CC
PV_Br_SNP_0197	A02	22480276	A/G	AA	AA	AA	AA	AA	AA	NN	AA	AA	AA	NN	AA	AA	AA	AA	AA	AA	AA	AA	NN	GG	AA	AA	AA	AA	AA	AA	AA	NN	AA	AA	AA	AA	AA	AA	AA	AA	AA	AA	AA	AA	NN	AA	AA	AA	AA	NN	AA	AA	AA	AA	AA	AA	NN	AA	AA
PV_Br_SNP_0207	A02	24175586	A/G	AG	GG	AG	GG	GG	AA	NN	AA	GG	AA	AA	AA	AA	AA	AA	AA	AA	AA	AA	NN	AA	AA	AA	AA	GG	GG	AA	GG	AA	AA	AA	AA	AA	GG	GG	AG	AA	AG	AA	AG	AA	GG	GG	AA	AA	AA	NN	AA	AA	AG	GG	GG	AA	AA	AA	AA
PV_Br_SNP_0211	A02	25024347	T/C	CC	CC	TC	CC	CC	CC	NN	CC	CC	CC	CC	CC	CC	CC	CC	CC	CC	CC	CC	NN	CC	CC	TC	CC	CC	CC	CC	CC	CC	CC	CC	CC	CC	CC	CC	CC	CC	CC	CC	CC	CC	CC	CC	NN	NN	CC	NN	CC	CC	CC	CC	CC	NN	CC	NN	NN
PV_Br_SNP_0213	A02	25488133	A/T	AT	AT	AT	AT	AT	AA	NN	AA	TT	AA	AA	TT	AT	TT	AT	AT	TT	AT	AT	NN	AA	AA	AT	TT	AT	TT	AA	TT	AT	AT	TT	AT	AA	AT	TT	AA	AT	AT	AT	NN	TT	TT	AT	TT	TT	AA	NN	AT	TT	AT	TT	AT	TT	TT	NN	AA
PV_Br_SNP_0221	A02	27606980	C/G	GG	GG	NN	GG	NN	GG	NN	GG	GG	GG	GG	GG	GG	GG	NN	GG	NN	GG	GG	GG	NN	NN	NN	NN	NN	NN	NN	NN	NN	GG	GG	GG	GG	GG	NN	NN	GG	GG	GG	GG	GG	GG	GG	GG	GG	GG	GG	GG	GG	GG	GG	CG	GG	NN	GG	GG
PV_Br_SNP_0222	A03	390404	T/C	CC	CT	TT	CT	CT	CT	TT	CC	CC	CC	TT	CC	CC	CC	CC	CC	TT	CC	CC	CT	NN	NN	NN	NN	NN	NN	NN	NN	NN	CC	CC	CC	CC	CC	CT	CT	CT	CT	CC	CC	CC	CC	CC	CC	CC	CC	CC	CC	CC	CC	TT	NN	NN	NN	CC	CC
PV_Br_SNP_0226	A03	1591560	A/C	CC	CC	CC	AA	CC	CC	NN	CC	CC	CC	CC	CC	CC	CC	CC	CC	NN	NN	AA	NN	CC	AA	CC	CC	AC	CC	AA	CC	NN	CC	CC	CC	CC	AC	AA	CC	CC	CC	AC	CC	CC	AA	AC	NN	AC	CC	NN	CC	CC	CC	CC	CC	AC	CC	NN	CC
PV_Br_SNP_0229	A03	3159821	T/C	TT	TT	TT	TT	TT	TT	NN	TT	TT	TT	TT	TT	TT	TT	TT	TT	TT	TT	TT	NN	TT	TT	TT	TT	TT	TT	TT	TT	TT	CC	CC	TT	TT	TT	TT	TT	CC	TT	TT	TT	TT	TT	TT	TT	TT	TT	NN	TT	TT	TT	TT	TT	TT	TT	TT	TT
PV_Br_SNP_0231	A03	3543566	T/G	GT	TT	TT	TT	TT	GT	NN	TT	GT	TT	TT	TT	GT	TT	GT	GG	TT	TT	GT	NN	GT	TT	TT	TT	TT	GT	GG	GG	GG	GG	GT	TT	TT	TT	TT	TT	TT	GT	TT	TT	GG	GT	TT	TT	TT	GT	NN	TT	GG	TT	TT	TT	TT	TT	TT	GG
PV_Br_SNP_1031	A03	4509906	A/G	AG	AG	AG	AG	AG	AG	NN	AG	AG	AG	AG	AG	AG	AG	AG	AG	AG	NN	AG	NN	AG	AG	AG	AG	AG	AG	AG	AG	AG	AG	AG	AG	AG	AG	AG	AG	AG	AG	AG	AG	AG	AG	AG	AG	AG	AG	NN	AG	AG	AG	AG	AG	AG	AG	AG	AG
PV_Br_SNP_1032	A03	4510458	A/G	GG	GG	AA	AA	AG	AG	NN	AG	AA	GG	GG	GG	AA	AG	AG	GG	GG	GG	GG	NN	AA	AA	AA	AA	AA	AG	GG	AA	AA	AA	AG	AG	AA	AA	AA	AG	AG	AG	AG	AA	GG	GG	AG	GG	AA	AA	NN	AA	AG	AG	AA	AA	AG	AA	AA	AG
PV_Br_SNP_0242	A03	7648839	A/G	GG	GG	AA	GG	GG	GG	GG	GG	GG	GG	GG	GG	GG	GG	GG	GG	GG	GG	GG	GG	NN	NN	NN	NN	NN	NN	NN	NN	NN	GG	GG	GG	GG	GG	GG	AG	GG	GG	GG	GG	GG	GG	GG	GG	GG	GG	GG	GG	GG	GG	GG	GG	NN	NN	AA	AG
PV_Br_SNP_0243	A03	7742095	G/C	GC	GG	CC	CC	CC	CC	NN	GG	GG	CC	CC	NN	CC	GG	GG	GG	GC	CC	GC	NN	GG	GC	CC	CC	GC	GG	GG	CC	CC	GG	NN	GG	CC	GG	GG	GG	CC	GC	GC	GC	GG	GG	GG	GG	GG	GC	NN	CC	GG	GC	CC	CC	GC	GG	CC	GC
PV_Br_SNP_0245	A03	8150031	A/C	CC	CC	CC	CC	CC	CC	NN	CC	CC	CC	CC	CC	CC	CC	CC	CC	CC	CC	CC	NN	CC	CC	CC	CC	CC	CC	CC	CC	CC	AC	CC	CC	CC	CC	CC	CC	AC	CC	CC	CC	CC	CC	CC	CC	CC	CC	NN	CC	CC	CC	CC	CC	CC	CC	AC	AC
PV_Br_SNP_0253	A03	10871276	T/C	CC	CC	CC	TT	CC	CC	TC	TC	TC	TT	CC	TC	TC	CC	TT	TT	CC	CC	TC	CC	NN	NN	NN	NN	NN	NN	NN	NN	NN	TC	TT	CC	CC	CC	TC	CC	TT	TT	TT	TT	CC	CC	CC	TT	CC	TT	TC	CC	TC	TT	CC	CC	CC	NN	CC	TC
PV_Br_SNP_0255	A03	11342547	T/C	CC	CT	TT	TT	TT	TT	NN	CT	TT	TT	TT	TT	TT	TT	CT	TT	TT	CC	TT	NN	TT	TT	CC	CT	CT	TT	TT	TT	CT	TT	TT	TT	TT	TT	TT	TT	TT	TT	TT	TT	TT	TT	TT	TT	TT	TT	NN	TT	CT	CT	TT	TT	CT	TT	CC	CT
PV_Br_SNP_0264	A03	14770684	T/C	TT	TT	TT	TT	TT	TT	NN	TT	TT	TT	TT	TT	TT	TT	TT	TT	TT	TT	TT	NN	TT	TT	CC	TT	TT	TT	TT	TT	TT	NN	TT	TT	TT	TT	TT	TT	TT	TT	TT	TT	TT	TT	TT	TT	TT	TT	NN	TT	TT	TT	TT	TT	TT	TT	NN	TT
PV_Br_SNP_0267	A03	15435114	C/T	TT	TT	CC	TT	TT	TT	NN	TT	TT	CT	CT	TT	CT	CT	TT	TT	CT	CT	CT	NN	TT	TT	CT	TT	TT	TT	TT	CC	TT	CT	TT	TT	TT	CT	TT	TT	TT	CT	TT	CT	TT	TT	CC	TT	CT	TT	NN	TT	CT	CC	CT	CT	TT	TT	CT	TT
PV_Br_SNP_0274	A03	17345774	T/C	TT	TT	TT	TT	TT	TT	CT	TT	TT	TT	TT	TT	TT	TT	TT	TT	TT	TT	TT	CT	NN	NN	NN	NN	NN	NN	NN	NN	NN	TT	TT	TT	TT	TT	TT	TT	TT	TT	TT	TT	TT	TT	TT	TT	TT	TT	TT	TT	TT	TT	CC	NN	TT	NN	TT	TT
PV_Br_SNP_1050	A03	18284950	A/G	GG	GG	AG	GG	GG	GG	GG	GG	GG	GG	GG	GG	GG	GG	GG	GG	GG	GG	GG	AG	NN	NN	NN	NN	NN	NN	NN	NN	NN	GG	GG	GG	GG	GG	GG	GG	GG	GG	GG	GG	GG	GG	GG	GG	GG	GG	GG	GG	GG	GG	GG	GG	GG	NN	GG	GG
PV_Br_SNP_0284	A03	19709518	T/G	GG	GG	GG	GG	GG	GG	NN	GG	GG	GG	GG	GG	GG	GG	GG	GG	GG	GG	GG	NN	GG	GG	TG	GG	GG	GG	GG	GG	GG	TG	TG	GG	GG	GG	GG	GG	GG	GG	GG	GG	GG	GG	GG	GG	GG	GG	NN	NN	GG	TT	GG	GG	GG	GG	GG	GG
PV_Br_SNP_0286	A03	20138490	A/G	AA	AG	GG	GG	AG	AG	NN	AA	GG	AA	AA	AG	GG	AA	GG	GG	GG	AG	AG	NN	GG	GG	GG	AA	AG	AG	GG	GG	GG	AG	AG	AA	AG	AG	GG	AG	AG	GG	GG	AA	AG	AG	AG	AA	AG	AG	NN	GG	AG	AG	GG	AG	AG	GG	AG	GG
PV_Br_SNP_0288	A03	20356994	T/C	CC	CC	TT	CC	CC	CC	NN	CC	CC	CC	CC	CC	CC	CC	CC	CC	TC	TC	CC	NN	CC	CC	CC	CC	CC	CC	CC	TT	CC	TC	TC	CC	TC	CC	CC	TC	TC	CC	CC	CC	CC	CC	CC	CC	CC	CC	NN	CC	CC	CC	CC	CC	CC	CC	CC	CC
PV_Br_SNP_1082	A03	21105376	A/G	GG	GG	GG	GG	GG	AG	GG	GG	GG	GG	GG	GG	GG	AG	AG	GG	GG	AG	GG	AG	NN	NN	NN	NN	NN	NN	NN	NN	NN	GG	AG	GG	GG	AG	GG	GG	GG	GG	GG	GG	GG	GG	GG	GG	GG	GG	GG	AG	GG	AG	AG	AA	NN	NN	GG	GG
PV_Br_SNP_0294	A03	22408309	A/G	GG	AG	GG	GG	GG	AA	GG	GG	GG	GG	GG	GG	GG	AG	GG	GG	AA	AG	GG	GG	NN	NN	NN	NN	NN	NN	NN	NN	NN	GG	GG	AA	GG	GG	GG	GG	GG	GG	GG	GG	GG	GG	GG	GG	GG	GG	AG	GG	GG	GG	GG	GG	GG	NN	GG	GG
PV_Br_SNP_0296	A03	22821489	A/C	CC	CC	AC	CC	CC	AC	NN	AC	AC	CC	CC	CC	CC	CC	AC	AC	CC	AC	CC	NN	CC	CC	CC	CC	AC	AA	CC	AA	AC	CC	CC	AC	CC	AA	AC	CC	CC	CC	CC	CC	AC	AA	AC	CC	CC	AC	NN	AA	CC	AC	AA	CC	CC	CC	CC	CC
PV_Br_SNP_0297	A03	22926755	A/G	AA	AA	GG	AG	GG	AA	NN	AA	AA	AA	AA	AA	AG	AG	AG	AA	AG	AA	AA	NN	AA	AA	AA	AA	AA	GG	AA	GG	AG	AA	AG	AA	AG	AG	AA	AA	AA	AA	AA	AA	AA	AA	AA	AA	GG	AA	NN	AA	GG	AA	GG	AG	GG	GG	AG	AA
PV_Br_SNP_0298	A03	23143245	A/G	GG	GG	GG	GG	GG	GG	NN	GG	AA	AA	AA	AG	GG	GG	GG	AG	AG	GG	GG	NN	AA	AA	AG	AA	AA	AA	AA	GG	GG	AA	AA	GG	GG	AG	GG	GG	GG	AG	AA	GG	AG	AA	GG	AA	GG	GG	NN	GG	GG	AG	NN	GG	GG	GG	AA	GG
PV_Br_SNP_0309	A03	26161200	C/T	TT	CC	TT	TT	TT	TT	TT	TT	TT	TT	CT	TT	TT	TT	TT	TT	TT	TT	TT	TT	NN	NN	NN	NN	NN	NN	NN	NN	NN	TT	CT	TT	TT	CT	TT	TT	TT	TT	TT	TT	CC	TT	TT	TT	CT	CT	TT	TT	TT	CT	TT	TT	NN	NN	CT	CT
PV_Br_SNP_1065	A03	27864125	A/T	TT	TT	TT	AT	AT	AA	AT	AT	AT	AT	AA	TT	AT	AA	AT	AT	TT	AT	TT	AT	NN	NN	NN	NN	NN	NN	NN	NN	NN	AA	AA	TT	TT	AT	AA	AT	AA	AA	AT	AT	TT	TT	TT	AT	AT	AT	AT	TT	AT	AA	TT	AT	AT	NN	AA	AT
PV_Br_SNP_0312	A03	28510242	A/G	GA	GG	GA	GG	GG	GG	NN	GG	GG	GG	GG	GG	GG	GG	GG	GG	GG	GA	GA	NN	AA	GG	AA	GG	GG	GG	GG	AA	AA	GG	GG	GA	GA	GG	GG	AA	AA	GG	GG	GG	GG	GG	GA	GG	GG	GA	NN	GG	GG	GA	AA	AA	GG	GG	NN	GG
PV_Br_SNP_0314	A03	29213500	A/G	AA	GA	AA	GA	AA	GA	NN	AA	AA	GA	GA	AA	GA	AA	AA	GG	AA	GG	AA	NN	GA	AA	GA	AA	AA	AA	AA	AA	AA	AA	AA	GA	AA	GA	AA	AA	AA	GA	AA	AA	GG	GA	AA	AA	AA	AA	NN	AA	AA	AA	AA	AA	AA	AA	AA	GG
PV_Br_SNP_0319	A04	601636	A/G	AA	AA	GG	AA	AA	AA	GA	AA	AA	AA	AA	AA	AA	AA	AA	GA	AA	AA	AA	GA	NN	NN	NN	NN	NN	NN	NN	NN	NN	AA	AA	AA	GA	AA	AA	AA	GG	GA	AA	AA	AA	AA	AA	AA	AA	AA	GA	GA	AA	GG	AA	GA	AA	NN	GG	AA
PV_Br_SNP_0320	A04	934960	A/G	GG	AG	GG	GG	GG	GG	NN	GG	AG	GG	GG	GG	GG	AA	AA	AA	GG	GG	GG	NN	GG	AG	GG	GG	AA	GG	GG	AA	GG	AA	GG	GG	AG	GG	AG	GG	AG	GG	AA	AA	GG	AG	AA	NN	GG	AG	NN	GG	NN	AG	GG	GG	AA	AA	NN	AG
PV_Br_SNP_0321	A04	1247632	T/G	GG	GG	TG	GG	GG	GG	NN	GG	GG	GG	GG	GG	GG	GG	GG	GG	TG	GG	GG	NN	TT	GG	GG	GG	GG	GG	GG	GG	GG	TG	TG	GG	TG	GG	GG	TT	GG	GG	GG	GG	GG	GG	GG	GG	GG	GG	NN	GG	GG	TT	GG	GG	GG	GG	GG	GG
PV_Br_SNP_0323	A04	1258335	T/G	TG	TT	TG	TT	TT	GG	NN	GG	GG	TG	TG	GG	TT	TG	TG	GG	TG	TG	GG	NN	TT	GG	TT	TG	TG	GG	GG	GG	TG	TG	TT	TT	TG	GG	GG	TG	GG	GG	GG	TT	TT	GG	GG	GG	GG	GG	NN	TT	GG	TT	GG	GG	TG	TT	GG	GG
PV_Br_SNP_0327	A04	2800639	A/G	GG	AA	AG	GG	GG	GG	GG	GG	GG	GG	GG	AG	GG	GG	GG	GG	GG	GG	GG	AG	NN	NN	NN	NN	NN	NN	NN	NN	NN	GG	GG	GG	GG	GG	GG	AG	GG	GG	AG	AG	AA	GG	GG	GG	GG	GG	GG	GG	AG	GG	GG	GG	GG	NN	GG	GG
PV_Br_SNP_0331	A04	6717123	T/C	CC	CT	CT	CC	CT	CT	NN	CC	TT	CC	CC	CT	TT	CT	CT	TT	TT	TT	TT	NN	TT	CT	CC	CC	CT	TT	CT	TT	TT	CC	CT	CC	CC	TT	TT	TT	TT	TT	CC	CC	CT	CT	CC	CC	CT	CC	NN	CC	CT	CC	TT	TT	CC	CC	TT	TT
PV_Br_SNP_0332	A04	6848553	A/G	AA	AA	GG	AA	AA	AA	NN	AA	AA	AA	AA	AA	AA	AA	AA	GA	AA	AA	AA	NN	AA	AA	AA	AA	AA	AA	AA	AA	AA	GA	GA	AA	AA	AA	AA	AA	AA	AA	AA	NN	AA	AA	AA	AA	GA	AA	NN	AA	AA	AA	NN	NN	AA	AA	NN	NN
PV_Br_SNP_0333	A04	6927029	G/A	GG	GG	GA	GG	GG	GG	NN	GG	GG	GA	GA	AA	GG	GA	GA	GG	AA	GG	AA	NN	GG	GA	AA	GA	GA	GG	AA	GG	AA	GG	GG	GA	GA	GA	GA	AA	GG	GG	GG	GG	GA	GA	GG	AA	AA	GA	NN	GG	GG	AA	AA	GA	GA	GA	GG	GG
PV_Br_SNP_0338	A04	9525656	T/C	CC	CC	NN	CC	CC	CC	CC	CC	CC	CC	CC	CC	CC	CC	CC	CC	CC	CC	CC	CC	NN	NN	NN	NN	NN	NN	NN	NN	NN	CC	CC	CC	CC	CC	CC	CC	CC	CC	CC	CC	CC	CC	CC	CC	NN	CC	CC	CC	CC	CC	CC	CC	TT	NN	CC	CC
PV_Br_SNP_0340	A04	9933369	T/G	GG	GG	TT	GG	TT	TT	NN	TT	TT	TT	TT	TT	GG	GG	GT	GG	TT	NN	TT	NN	TT	GT	TT	TT	TT	TT	TT	TT	TT	TT	TT	TT	GG	GG	GG	TT	GG	GG	GT	GG	GG	TT	GG	TT	GG	GG	NN	TT	GG	GG	TT	TT	TT	TT	GG	TT
PV_Br_SNP_0341	A04	10430690	A/G	GG	GG	GG	AA	AA	GG	NN	AA	AA	AA	AA	AA	AA	AA	AG	AG	GG	AG	GG	NN	GG	AA	AA	AA	AG	AA	AA	GG	AG	AA	GG	GG	GG	AA	AA	GG	GG	AA	AA	GG	AA	AA	AA	AG	AG	AG	NN	AA	GG	GG	GG	GG	AA	AG	GG	AG
PV_Br_SNP_0342	A04	10902499	T/C	CC	CC	CC	CC	CC	CC	NN	CC	CC	CC	CC	CC	CC	CC	CC	CC	CC	CC	CC	NN	CC	CC	CC	CC	CC	CC	CC	CC	CC	CC	CC	CC	CC	CC	CC	TT	CC	CC	CC	CC	CC	CC	CC	CC	CC	CC	NN	CC	CC	CC	CC	CC	CC	CC	CC	CC
PV_Br_SNP_0345	A04	12871772	T/C	CC	CC	NN	CC	TT	TC	CC	TT	TC	TC	TT	TC	CC	TC	TC	TT	TC	NN	CC	TC	NN	NN	NN	NN	NN	NN	NN	NN	NN	TC	CC	CC	CC	TC	TC	CC	CC	CC	TC	TC	NN	CC	TT	TT	CC	CC	TC	TT	CC	NN	CC	NN	TC	NN	TC	TC
PV_Br_SNP_0348	A04	14057322	C/A	AA	CC	CA	CA	CA	AA	NN	AA	AA	AA	AA	CC	CC	CC	CC	AA	AA	AA	AA	NN	AA	AA	AA	CA	CA	AA	AA	AA	AA	CC	CA	CA	CC	AA	CA	AA	AA	AA	AA	CC	CC	AA	CA	CC	CC	CA	NN	AA	CA	CA	CA	AA	CA	CA	AA	NN
PV_Br_SNP_0349	A04	14135868	T/G	TT	TT	GG	TT	TG	TG	NN	TT	TT	TG	TG	TT	TT	TT	TT	TT	TT	TG	TT	NN	TT	TT	TT	TT	TT	TT	TT	GG	TG	TT	TG	TT	TG	TT	TT	TT	TT	TT	TT	TT	TT	TG	TT	TT	TT	TG	NN	TT	TT	TT	GG	NN	TT	TT	TG	TG
PV_Br_SNP_0350	A04	14646618	A/G	GG	GG	GG	GG	GG	GG	NN	GG	GG	GG	GG	GG	GG	GG	GG	GG	GG	GG	GG	NN	GG	GG	GG	GG	GG	GG	GG	AA	GG	AG	GG	GG	GG	GG	GG	GG	GG	GG	GG	GG	GG	GG	GG	GG	GG	GG	NN	GG	GG	GG	GG	AG	GG	GG	GG	GG
PV_Br_SNP_0353	A04	16153523	A/T	AT	AT	AA	AA	TT	AT	AA	AA	AA	AA	AA	AT	TT	AT	AT	AA	AA	AA	AA	AA	NN	NN	NN	NN	NN	NN	NN	NN	NN	TT	AA	AT	AT	TT	TT	AT	TT	TT	AA	AA	AT	TT	AT	AA	TT	TT	AT	AT	AA	TT	TT	NN	TT	NN	TT	AA
PV_Br_SNP_0363	A04	17626598	G/A	AA	AA	AA	AA	AA	GG	NN	AA	AA	AA	AA	AA	AA	AA	AA	GA	AA	GA	AA	NN	AA	AA	AA	AA	AA	AA	AA	AA	GA	GA	GA	AA	GA	AA	AA	AA	AA	AA	AA	AA	AA	AA	AA	AA	AA	AA	NN	AA	AA	AA	AA	AA	AA	AA	NN	AA
PV_Br_SNP_0364	A04	17743027	T/C	TC	TC	CC	CC	CC	CC	NN	CC	CC	NN	NN	NN	CC	CC	CC	CC	CC	NN	CC	NN	CC	CC	CC	CC	CC	CC	CC	CC	CC	CC	CC	CC	TC	CC	CC	TT	CC	CC	CC	CC	CC	NN	CC	CC	CC	CC	NN	CC	CC	TT	CC	CC	CC	CC	NN	NN
PV_Br_SNP_0372	A04	18705486	A/G	GG	GG	GG	GG	GG	GG	GG	GG	GG	GG	GG	GG	GG	GG	GG	GG	GG	GG	GG	GG	NN	NN	NN	NN	NN	NN	NN	NN	NN	GG	GG	GG	GG	GG	GG	GG	GG	GG	GG	GG	GG	GG	GG	GG	GG	GG	GG	GG	GG	GG	GG	GG	GG	NN	GG	GG
PV_Br_SNP_0374	A05	37346	C/T	CC	CC	CC	CC	CC	CC	CC	CC	CC	CC	CC	CC	CC	CC	CC	TC	CC	CC	CC	CC	NN	NN	NN	NN	NN	NN	NN	NN	NN	CC	CC	CC	TC	CC	CC	CC	CC	CC	CC	CC	CC	CC	CC	CC	CC	CC	CC	CC	CC	CC	CC	CC	NN	NN	CC	TC
PV_Br_SNP_0375	A05	60311	C/G	GG	GG	GG	GG	GG	CG	NN	GG	GG	GG	GG	GG	GG	GG	GG	NN	GG	CC	GG	NN	CG	GG	CC	GG	GG	GG	GG	GG	GG	NN	NN	CG	CG	GG	GG	CC	GG	GG	GG	GG	GG	GG	GG	GG	GG	GG	NN	GG	GG	CC	GG	GG	GG	GG	NN	CC
PV_Br_SNP_0378	A05	519882	C/T	CC	CC	CC	CC	CC	CC	NN	CC	CC	CC	CC	CC	NN	CC	NN	CC	CC	CC	CC	NN	CC	CC	CC	CC	CC	CC	CC	CC	CC	NN	NN	CC	CC	CC	CC	CC	CC	CC	CC	CC	CC	CC	CC	CC	CC	CC	NN	CC	CC	CC	CC	CC	CC	CC	TT	CC
PV_Br_SNP_0381	A05	1125894	C/T	TT	TT	TT	TT	TT	TT	NN	TT	TT	TT	TT	TT	TT	TT	TT	TT	TT	TT	TT	NN	TT	TT	TT	TT	TT	TT	TT	CC	TT	CC	CC	TT	TT	TT	TT	TT	TT	TT	TT	TT	TT	TT	TT	TT	TT	TT	NN	TT	TT	TT	TT	CC	TT	TT	CC	TT
PV_Br_SNP_0388	A05	5146576	A/G	AA	AA	AA	AA	AA	AA	AA	AA	AA	AA	AA	AA	AA	AA	AA	AA	AA	AA	AA	AA	NN	NN	NN	NN	NN	NN	NN	NN	NN	AA	AA	GA	AA	GA	AA	AA	AA	AA	AA	AA	AA	GA	AA	AA	AA	AA	AA	AA	AA	AA	AA	NN	AA	NN	AA	GA
PV_Br_SNP_0393	A05	6473191	T/C	TT	TT	TT	TT	TT	TT	NN	TT	TT	TT	TT	TT	TT	TT	TT	TT	TT	TT	TT	NN	TT	TT	TT	TT	TT	TT	TT	TT	TT	TT	TT	TT	TT	TT	TT	TT	TT	TT	TT	NN	NN	TT	NN	TT	TT	TT	NN	TT	TT	TT	TT	TT	TT	TT	NN	TT
PV_Br_SNP_0394	A05	6646646	C/A	CC	CC	CC	CC	CC	CC	NN	CC	CC	AC	NN	CC	CC	CC	CC	CC	CC	CC	CC	NN	CC	CC	CC	CC	CC	CC	CC	CC	CC	CC	CC	CC	CC	CC	CC	CC	CC	CC	CC	CC	CC	CC	CC	CC	CC	CC	NN	CC	CC	CC	CC	CC	CC	CC	CC	CC
PV_Br_SNP_1057	A05	8925567	C/T	TT	TT	TT	TT	TT	TT	TT	TT	TT	TT	TT	TT	TT	TT	TT	TT	TT	TT	TT	TT	NN	NN	NN	NN	NN	NN	NN	NN	NN	TT	TT	TT	TT	TT	TT	TT	TT	TT	TT	TT	TT	TT	TT	TT	TT	TT	TT	TT	TT	TT	CT	TT	NN	NN	TT	TT
PV_Br_SNP_1081	A05	8964613	A/G	GG	GG	GG	GG	GG	GG	GG	GG	GG	GG	GG	GG	GG	GG	GG	GG	GG	GG	GG	GG	NN	NN	NN	NN	NN	NN	NN	NN	NN	GG	GG	AG	GG	GG	GG	GG	GG	GG	GG	GG	GG	GG	GG	GG	GG	GG	GG	GG	GG	GG	AG	GG	NN	NN	GG	GG
PV_Br_SNP_0413	A05	10244748	G/T	TT	TT	TT	TT	TT	TT	TT	TT	TT	TT	TT	TT	TT	TT	TT	TT	TT	TT	TT	TT	NN	NN	NN	NN	NN	NN	NN	NN	NN	TT	TT	TT	TT	TT	TT	TT	TT	TT	TT	TT	TT	TT	TT	TT	TT	TT	TT	TT	TT	TT	TT	TT	TT	NN	TT	TT
PV_Br_SNP_0415	A05	10368006	C/A	AA	CC	CC	AA	AA	AA	NN	CC	CC	NN	NN	AA	NN	CC	AA	NN	CC	NN	AC	NN	CC	CC	CC	AA	CC	CC	CC	CC	CC	AA	NN	CC	CC	NN	CC	NN	NN	AA	CC	CC	AA	AA	AC	AA	AC	AC	NN	CC	CC	CC	CC	CC	AA	CC	AC	AC
PV_Br_SNP_0417	A05	10731008	T/C	CC	CC	CC	CC	CC	CC	NN	CC	CC	CC	CC	CC	CC	NN	CC	CC	CC	NN	CC	NN	CC	CC	CC	CC	CC	CC	CC	CC	CC	CC	CC	CC	CC	CC	CC	CC	CC	CC	CC	CC	CC	CC	CC	CC	CC	CC	NN	CC	CC	CC	TC	TC	CC	CC	CC	CC
PV_Br_SNP_0424	A05	11496787	G/A	GG	GG	NN	GG	GG	GG	GG	GG	GG	GG	GG	GG	GG	GG	GG	AA	GG	GG	GG	GG	NN	NN	NN	NN	NN	NN	NN	NN	NN	GG	GG	GG	GG	GG	GG	GG	GG	GG	GG	GG	GG	GG	GG	GG	GG	GG	GG	GG	GG	GG	GG	GG	GG	NN	GG	GG
PV_Br_SNP_0426	A05	11626502	G/A	GG	GG	GG	GG	GG	GG	NN	GG	GG	GG	GG	GG	GG	GG	GG	GG	GG	NN	GG	NN	GG	GG	GG	GG	GG	GG	GG	GG	GG	GG	GG	GG	GG	GG	GG	GG	GG	GG	GG	GG	GG	GG	GG	GG	GG	GG	NN	GG	GG	GG	GG	GG	GG	GG	GG	GG
PV_Br_SNP_0430	A05	12088226	T/G	TT	TT	TT	GT	GT	TT	NN	TT	TT	TT	TT	TT	TT	GT	GT	TT	TT	GT	GG	NN	GG	TT	GG	TT	NN	TT	GG	TT	GT	TT	GG	TT	TT	TT	GG	GT	TT	TT	TT	TT	NN	TT	GT	TT	NN	GT	NN	NN	GT	TT	TT	TT	TT	TT	NN	GT
PV_Br_SNP_0444	A05	12922255	G/C	GG	GG	NN	GG	GG	GG	GG	GG	GG	GG	GG	GG	GG	GG	GG	GG	GG	GG	GG	GG	NN	NN	NN	NN	NN	NN	NN	NN	NN	GG	GG	GG	GG	GG	GG	GG	GG	GG	GG	GG	GG	GG	GG	GG	GG	GG	GG	GG	GG	GG	GG	GG	GG	NN	GG	GG
PV_Br_SNP_0448	A05	13282738	A/G	GG	AG	AG	AA	GG	AA	NN	GG	GG	AA	AA	GG	AG	GG	AA	GG	GG	NN	AA	NN	AA	GG	AA	AG	GG	GG	AA	GG	GG	AA	AA	GG	GG	AA	AA	AG	GG	AA	GG	GG	NN	AA	AG	AG	GG	AG	NN	AA	AG	GG	GG	GG	GG	GG	NN	AG
PV_Br_SNP_0464	A05	15526140	G/A	AA	AA	AA	AA	AA	AA	AA	AA	AA	AA	AA	AA	AA	AA	AA	AA	AA	AA	AA	AA	NN	NN	NN	NN	NN	NN	NN	NN	NN	AA	AA	AA	AA	AA	AA	AA	AA	AA	AA	AA	AA	AA	AA	AA	AA	AA	AA	AA	AA	AA	AA	AA	NN	NN	GA	AA
PV_Br_SNP_0465	A05	15526505	T/A	TT	TT	TT	TT	NN	TT	NN	TT	TT	TT	TT	TT	TT	TT	TT	TT	TT	NN	TT	NN	TT	TT	TT	TT	TT	TT	TT	TT	TT	AT	AT	NN	TT	TT	TT	TT	TT	TT	TT	TT	TT	TT	TT	TT	TT	TT	NN	NN	TT	NN	TT	TT	TT	TT	NN	TT
PV_Br_SNP_1042	A05	15800094	T/C	CC	CC	CC	CC	CC	CC	CC	CC	CC	CC	CC	CC	CC	CC	CC	CC	CC	CC	CC	CC	NN	NN	NN	NN	NN	NN	NN	NN	NN	CC	CC	CC	CC	CC	CC	CC	CC	CC	CC	CC	CC	CC	CC	CC	CC	CC	CC	CC	CC	CC	CC	CC	NN	NN	CC	CC
PV_Br_SNP_0471	A05	15949259	C/T	CC	CC	CC	CC	CC	CC	NN	CC	CC	CC	CC	CC	CC	CC	CC	CC	CC	NN	CC	NN	CC	CC	CC	CC	CC	CC	CC	CC	CC	CC	CC	CC	CC	CC	CC	CC	CC	CC	CC	CC	CC	CC	CC	CC	CC	CC	NN	CC	CC	CC	CC	CC	CC	CC	CC	CC
PV_Br_SNP_0476	A05	16404787	C/T	CC	CC	CC	TT	CC	TT	NN	NN	CC	CC	CC	CC	TT	TC	TT	TT	CC	TT	TC	NN	CC	CC	CC	NN	CC	CC	CC	CC	CC	TT	TT	CC	CC	TC	TC	TC	CC	CC	TC	TC	TT	CC	CC	TC	TT	NN	NN	CC	TC	CC	CC	CC	CC	TT	NN	CC
PV_Br_SNP_1051	A05	17589777	T/G	GG	GG	NN	GT	GT	GG	GT	GT	GG	GG	GG	GG	GT	GG	GT	GG	TT	GT	GG	TT	NN	NN	NN	NN	NN	NN	NN	NN	NN	TT	TT	GG	TT	GT	GG	GG	GG	GT	TT	GG	GT	GG	TT	GT	TT	GT	TT	GT	TT	GT	TT	TT	NN	NN	TT	GG
PV_Br_SNP_0492	A05	18313091	C/T	CT	CC	NN	CT	CC	CT	CT	CT	CC	CC	TT	TT	CC	CT	CT	CC	TT	CT	CT	CT	NN	NN	NN	NN	NN	NN	NN	NN	NN	CC	TT	CC	CT	CT	CC	CT	CC	TT	CC	NN	CC	CC	CT	CT	TT	CC	TT	CT	CC	TT	TT	TT	CT	NN	TT	CT
PV_Br_SNP_0493	A05	18351619	C/A	CC	CC	CC	CC	CC	CC	NN	CC	CC	NN	NN	NN	NN	CC	CC	NN	CC	NN	CC	NN	CC	CC	CC	CC	CC	CC	CC	CC	CC	NN	NN	CC	CC	CC	CC	CC	CC	CC	CC	CC	CC	CC	CC	CC	CC	CC	NN	CC	CC	CC	CC	CC	CC	CC	CC	CC
PV_Br_SNP_0500	A05	20060453	A/T	AA	TT	AA	AA	AA	AA	NN	AA	AA	AA	AA	AA	AA	AA	AA	AA	AA	TT	AA	NN	AA	AA	AA	AA	AA	TT	AA	AA	AA	NN	NN	AA	AA	AA	AA	AA	AA	AA	AA	AA	AA	NN	NN	AA	AA	AA	NN	NN	AA	NN	NN	AA	AA	AA	NN	AA
PV_Br_SNP_0509	A05	22293235	T/G	TT	TT	TT	TT	TT	TT	NN	TT	TT	TT	TT	TT	TT	TT	TT	TT	TT	TT	NN	NN	TT	TT	NN	TT	TT	TT	TT	TT	GG	GT	GT	TT	NN	TT	TT	GG	TT	TT	TT	NN	NN	TT	TT	NN	TT	TT	NN	TT	TT	TT	TT	TT	TT	TT	TT	TT
PV_Br_SNP_0510	A05	22623229	A/T	AA	AA	AA	TT	AA	TT	NN	TA	AA	TT	TT	AA	TT	TA	AA	TA	AA	NN	AA	NN	AA	TA	TT	AA	AA	AA	AA	AA	TA	NN	TA	AA	TA	AA	AA	TT	AA	AA	AA	TT	TT	TA	AA	TA	TA	TT	NN	AA	TA	TA	AA	AA	TA	AA	AA	TA
PV_Br_SNP_0512	A05	23153672	T/C	CT	CT	CC	CC	CC	TT	NN	TT	CC	CC	CC	TT	TT	TT	CC	CC	CT	TT	TT	NN	TT	CT	TT	TT	TT	TT	TT	TT	CC	TT	CC	CC	CC	TT	TT	TT	CC	TT	TT	CC	TT	TT	TT	TT	TT	TT	NN	TT	CC	CC	TT	TT	CT	TT	CC	CT
PV_Br_SNP_0513	A05	23581175	C/T	TC	TC	CC	CC	CC	CC	NN	TC	CC	TC	TC	TC	TT	TC	TC	TC	TC	CC	TC	NN	TC	TC	CC	CC	CC	CC	CC	CC	CC	TT	CC	TC	TC	CC	CC	CC	CC	TC	CC	CC	TT	CC	CC	TC	TT	CC	NN	CC	TT	CC	TC	CC	CC	CC	TC	TC
PV_Br_SNP_0517	A06	251018	A/T	AA	AA	AA	AA	AA	AA	TA	AA	AA	AA	AA	AA	AA	AA	AA	AA	AA	AA	AA	AA	NN	NN	NN	NN	NN	NN	NN	NN	NN	AA	AA	AA	AA	AA	AA	AA	AA	AA	AA	AA	AA	AA	AA	AA	AA	AA	AA	AA	AA	AA	AA	AA	AA	NN	AA	AA
PV_Br_SNP_1043	A06	427379	G/A	GG	GG	AG	GG	GG	GG	AA	GG	GG	GG	GG	GG	GG	GG	GG	AA	GG	GG	GG	NN	NN	NN	NN	NN	NN	NN	NN	NN	NN	GG	GG	GG	AG	GG	GG	AA	AA	GG	GG	GG	GG	GG	GG	AG	GG	GG	GG	AG	GG	AA	AA	AA	GG	NN	AA	AG
PV_Br_SNP_0519	A06	796251	A/G	AA	AA	AA	AA	AA	AA	NN	AA	AA	AA	AA	AA	AA	AA	AA	AA	GA	AA	AA	NN	GG	AA	AA	AA	AA	AA	AA	GG	AA	AA	AA	AA	AA	AA	AA	GG	AA	AA	AA	AA	AA	AA	AA	AA	AA	AA	NN	AA	AA	AA	AA	GA	AA	AA	NN	AA
PV_Br_SNP_0520	A06	877234	A/G	AA	AA	AA	AA	AA	AA	NN	AA	AA	AA	AA	AA	AA	AA	AA	AA	AA	AA	AA	NN	AA	AA	AA	AA	AA	AA	AA	AA	AA	GA	GA	AA	AA	AA	AA	AA	AA	AA	AA	AA	AA	AA	AA	AA	AA	AA	NN	AA	AA	AA	AA	AA	AA	AA	NN	AA
PV_Br_SNP_0521	A06	1074004	A/G	AA	AA	GA	AA	AA	AA	NN	AA	AA	AA	AA	AA	AA	AA	AA	AA	AA	NN	AA	NN	AA	AA	AA	AA	AA	AA	NN	AA	AA	AA	AA	AA	AA	AA	AA	AA	AA	AA	AA	AA	AA	AA	AA	AA	AA	AA	NN	AA	AA	AA	AA	AA	AA	AA	NN	AA
PV_Br_SNP_0526	A06	2011195	A/G	GG	GG	GG	GG	GG	GG	GG	GG	GG	GG	GG	GG	GG	GG	GG	AG	GG	GG	GG	GG	NN	NN	NN	NN	NN	NN	NN	NN	NN	GG	GG	GG	AG	GG	GG	GG	GG	GG	GG	GG	GG	GG	GG	GG	GG	GG	GG	GG	GG	GG	AA	AA	AA	NN	GG	GG
PV_Br_SNP_0528	A06	2238277	A/T	TT	TT	TT	TT	TT	TT	NN	TT	TT	TT	TT	TT	TT	TT	TT	AT	AT	NN	TT	NN	AA	TT	TT	TT	TT	TT	TT	TT	TT	TT	TT	TT	TT	TT	TT	TT	TT	TT	TT	TT	TT	TT	TT	TT	TT	TT	NN	TT	TT	TT	AA	AT	TT	TT	NN	TT
PV_Br_SNP_0530	A06	2691656	A/T	AA	AA	AA	AA	AA	AA	NN	AA	NN	AA	AA	AA	AA	AA	AA	AA	AA	AA	AA	NN	AA	NN	AA	AA	AA	AA	AA	AA	AA	AA	AA	AA	NN	AA	NN	AA	AA	AA	AA	AA	AA	AA	AA	AA	AA	NN	NN	AA	AA	AA	NN	AA	AA	AA	AA	NN
PV_Br_SNP_1034	A06	2707359	C/T	CT	CC	CC	TT	CC	CC	NN	CC	CT	CC	CC	CC	CT	CT	TT	CC	CT	TT	CC	NN	TT	CC	TT	CC	CC	CC	CC	CC	CC	CT	CT	CT	TT	CT	CC	TT	TT	CC	CC	CC	CT	TT	CC	TT	CC	CC	NN	CC	CT	CT	CT	CC	CT	CC	TT	CC
PV_Br_SNP_0534	A06	3924739	A/C	CA	AA	CA	CA	AA	AA	NN	AA	AA	AA	AA	AA	CA	CA	AA	CA	AA	AA	AA	NN	CC	AA	AA	AA	CA	AA	AA	AA	AA	AA	CA	AA	AA	AA	CC	AA	AA	AA	AA	AA	AA	AA	AA	CA	AA	CA	NN	AA	AA	AA	AA	AA	AA	AA	AA	AA
PV_Br_SNP_1039	A06	6303418	A/C	AA	AA	AA	AA	AA	AC	NN	AA	NN	AA	AA	CC	AA	AA	AA	AA	AA	AA	AA	NN	AA	AA	AA	AA	AA	AA	AA	AA	AA	AA	AA	AA	AA	AA	AA	AA	AA	AC	AA	AA	AA	AA	AC	AC	AC	AA	NN	AC	AA	AA	AA	AA	AC	AA	AA	AA
PV_Br_SNP_1040	A06	6304099	T/C	TT	TT	TT	TT	TT	TT	TT	TT	TT	TT	TT	TT	TT	TT	TT	TT	CC	CT	TT	TT	NN	NN	NN	NN	NN	NN	NN	NN	NN	TT	CT	TT	TT	TT	TT	TT	TT	TT	TT	TT	TT	TT	TT	TT	TT	TT	CT	TT	TT	TT	TT	TT	TT	NN	TT	TT
PV_Br_SNP_0545	A06	8198336	A/T	TT	TT	TT	TT	TT	AT	TT	TT	TT	TT	AT	TT	TT	TT	TT	AT	TT	TT	TT	TT	NN	NN	NN	NN	NN	NN	NN	NN	NN	TT	TT	TT	TT	TT	TT	TT	TT	TT	TT	AT	TT	TT	TT	AA	TT	TT	TT	TT	TT	TT	TT	TT	TT	NN	AT	AT
PV_Br_SNP_0549	A06	10126924	A/G	GG	GG	GG	GG	GG	GG	NN	GG	GG	GG	GG	GG	GG	GG	GG	GG	GG	GG	GG	NN	GG	GG	GG	GG	GG	GG	GG	GG	GG	GG	GG	GG	GG	GG	GG	GG	GG	GG	GG	GG	GG	GG	GG	GG	GG	GG	NN	GG	GG	GG	GG	GG	GG	GG	NN	GG
PV_Br_SNP_0551	A06	10741922	T/A	AA	AA	AA	AA	AA	AA	NN	AA	AA	AA	AA	AA	AA	AA	AA	TA	AA	AA	AA	NN	AA	AA	AA	AA	AA	AA	AA	AA	AA	TA	TA	AA	AA	AA	AA	AA	AA	AA	AA	AA	AA	AA	AA	AA	AA	AA	NN	AA	AA	TT	AA	TT	AA	AA	NN	TT
PV_Br_SNP_0554	A06	11039171	C/T	CC	CC	NN	CC	CC	CC	NN	CC	CC	CC	CC	CC	CC	CC	CC	CC	CC	CC	CC	NN	TT	CC	TT	CC	CC	CC	CC	CC	CC	CC	CC	TT	CC	CC	CC	TT	TT	CC	CC	CC	CC	CC	CC	CC	CC	CC	NN	NN	CC	NN	NN	TT	CC	NN	TT	CC
PV_Br_SNP_0565	A06	14855293	T/G	GG	GG	NN	GG	TG	TG	GG	GG	GG	GG	TT	TT	GG	TT	GG	TG	GG	GG	GG	TG	NN	NN	NN	NN	NN	NN	NN	NN	NN	GG	TG	TT	TG	GG	GG	TT	GG	GG	GG	GG	TG	GG	TG	TT	TT	TG	TG	TG	GG	GG	GG	NN	TG	NN	GG	GG
PV_Br_SNP_0571	A06	17743723	A/C	CC	AA	AA	CA	AA	AA	NN	AA	AA	AA	AA	AA	CA	CA	AA	AA	AA	AA	AA	NN	AA	AA	AA	AA	AA	AA	AA	AA	AA	AA	AA	CA	CA	AA	AA	CC	CA	AA	AA	AA	AA	AA	AA	CC	AA	CA	NN	AA	AA	AA	CA	CA	AA	AA	CA	AA
PV_Br_SNP_0573	A06	18594484	A/T	TT	TT	AA	TT	TT	TT	NN	TT	TT	TT	TT	TT	TT	TT	TT	AT	TT	TT	TT	NN	TT	TT	TT	TT	TT	TT	TT	TT	AT	TT	TT	TT	AT	TT	TT	TT	TT	TT	TT	TT	TT	TT	TT	TT	TT	TT	NN	TT	NN	TT	AT	AT	TT	TT	TT	TT
PV_Br_SNP_0578	A06	19646308	T/C	CC	CC	CC	CC	CC	CC	CC	CC	CC	CC	CC	CC	CC	CC	CC	CC	CC	CC	CC	CC	NN	NN	NN	NN	NN	NN	NN	NN	NN	CC	CC	CC	TT	CC	CC	CC	CC	CC	CC	CC	CC	TC	CC	CC	CC	CC	CC	CC	CC	CC	NN	TT	CC	NN	CC	CC
PV_Br_SNP_0585	A06	20214699	T/C	CC	CC	CC	CC	CC	CC	NN	CC	CC	CC	CC	CC	CC	CC	CC	CC	CC	CC	CC	NN	CC	CC	CC	CC	CC	CC	CC	CC	CC	CC	CC	CC	CC	CC	CC	CC	CC	CC	CC	CC	CC	CC	CC	CC	CC	CC	NN	CC	CC	CC	CC	CC	CC	CC	CC	CC
PV_Br_SNP_0586	A06	20344798	T/G	GG	GG	GG	GG	GG	GG	NN	NN	GG	GG	GG	GG	GG	GG	GG	GG	GG	GG	GG	NN	GG	NN	GG	GG	GG	NN	GG	GG	GG	GG	GG	GG	GG	GG	GG	GG	GG	GG	NN	NN	GG	NN	GG	GG	NN	GG	NN	GG	GG	GG	GG	GG	GG	GG	GG	NN
PV_Br_SNP_0587	A06	20423475	A/C	AA	AA	AA	AA	AA	AA	NN	AA	AA	AA	AA	AA	AA	AA	AA	AA	AA	AA	AA	NN	AA	AA	AA	AA	AA	AA	AA	AA	AA	AA	AA	AA	AA	AA	AA	AA	AA	AA	AA	AA	AA	AA	AA	AA	AA	AA	NN	AA	AA	AA	AA	AA	AA	AA	AA	AA
PV_Br_SNP_0597	A06	23816726	A/T	TT	AT	TT	TT	AT	AT	TT	AT	AA	TT	AT	NN	AT	AT	AT	TT	TT	AT	AT	NN	NN	NN	NN	NN	NN	NN	NN	NN	NN	AT	TT	TT	TT	TT	TT	AT	AT	TT	AT	TT	AT	TT	AT	AA	AT	TT	AT	TT	AT	AT	TT	TT	AA	NN	AT	AT
PV_Br_SNP_0600	A06	24527870	T/C	CC	CC	CC	CC	CC	CC	NN	CC	CC	CC	CC	CC	CC	CC	CC	CC	CC	CC	CC	NN	CC	CC	CC	CC	CC	CC	CC	CC	CC	CC	CC	CC	CC	CC	CC	CC	TC	CC	CC	CC	CC	CC	CC	CC	CC	CC	NN	CC	CC	CC	CC	CC	CC	CC	CC	CC
PV_Br_SNP_0601	A06	24904044	T/C	CC	CC	CT	NN	NN	NN	NN	NN	NN	NN	NN	NN	NN	NN	CC	CC	CT	NN	NN	NN	NN	NN	NN	NN	NN	NN	NN	TT	NN	CC	CC	CC	CC	NN	CC	CC	CC	CC	CC	NN	CC	NN	NN	CC	NN	NN	NN	NN	NN	CT	CC	CT	NN	NN	NN	CC
PV_Br_SNP_0602	A06	24995754	G/C	GG	GG	GG	GG	GG	GG	NN	GG	CG	GG	NN	CG	NN	GG	NN	NN	GG	NN	GG	NN	GG	CG	GG	GG	GG	GG	GG	CG	GG	NN	GG	NN	NN	NN	GG	NN	GG	GG	GG	GG	NN	GG	GG	GG	GG	GG	NN	GG	NN	NN	NN	GG	GG	GG	NN	NN

SNP marker ID	Chromosome	Physical position (bp)	Polymorphic site	T_1050V	T_1283V	T_163V	T_307V	T_385V	T_738V	T_821V	T_826V	T_830V	FT_001	FT_002	FT_003	FT_004	FT_005	FT_047
PV_Br_SNP_1069	A06	26158880	A/G	AG	AG	AA	AG	AG	AA	AA	AG	AA	GG	AG	AA	AG	AA	AG
PV_Br_SNP_1047	A07	107888	C/G	CC	GG	CC	CG	CC	CG	CC	CG	CG	CG	CC	CG	GG	CG	GG
PV_Br_SNP_0607	A07	286182	T/C	CC	CC	CC	CC	CC	CC	CC	CC	CC	CC	CC	CC	CC	CC	CC
PV_Br_SNP_0609	A07	611531	A/G	GG	GG	GG	GG	GG	NN	NN	GG	GG	GG	GG	GG	GG	GG	GG
PV_Br_SNP_0610	A07	797437	A/G	GG	GG	AG	GG	GG	GG	NN	GG	GG	GG	GG	GG	GG	GG	GG
PV_Br_SNP_0611	A07	931243	C/T	TT	TT	TT	TT	TT	TT	NN	TT	TT	TT	NN	CT	TT	NN	NN
PV_Br_SNP_0616	A07	1592883	A/G	GG	GG	NN	GG	GG	AA	GG	AA	GG	GG	GG	GG	GG	GG	GG
PV_Br_SNP_0620	A07	4705134	T/G	GG	GG	GT	GG	GG	GT	NN	GT	GT	GG	GG	TT	TT	TT	GG
PV_Br_SNP_0622	A07	5385095	G/T	GT	GG	TT	GT	GG	NN	NN	TT	GG	NN	GT	NN	TT	TT	GT
PV_Br_SNP_0637	A07	11626771	A/G	AA	AA	AA	AA	AA	AA	AA	AA	AA	AA	AA	AA	AA	AA	AA
PV_Br_SNP_0641	A07	12369740	T/C	CC	CT	CT	CT	CC	CT	NN	CT	CC	NN	NN	TT	NN	CT	CC
PV_Br_SNP_0645	A07	13144591	T/G	GG	GT	GT	GT	TT	TT	NN	TT	TT	TT	TT	TT	GG	GG	TT
PV_Br_SNP_0646	A07	14186738	G/A	GA	GA	AA	AA	AA	AA	NN	AA	AA	AA	AA	AA	AA	AA	AA
PV_Br_SNP_0651	A07	14974921	T/C	NN	NN	NN	NN	TT	NN	CT	TT	NN	TT	TT	TT	NN	TT	TT
PV_Br_SNP_0653	A07	16550579	T/G	GG	GG	GG	GG	GG	GG	NN	GG	GG	GG	GG	GG	GG	GG	GG
PV_Br_SNP_0656	A07	16780120	T/C	TT	TT	TT	TT	TT	TT	NN	TT	TT	TT	TT	TT	TT	TT	TT
PV_Br_SNP_0657	A07	16977664	C/G	GG	GG	GG	GG	GG	GG	NN	GG	NN	NN	NN	GG	GG	GG	GG
PV_Br_SNP_0663	A07	17756345	A/T	TT	TT	TT	TT	TT	TT	TT	TT	TT	TT	TT	TT	TT	TT	TT
PV_Br_SNP_0664	A07	17798150	T/C	CC	CC	CC	CC	CC	CC	NN	CC	CC	CC	CC	NN	CC	CC	CC
PV_Br_SNP_0665	A07	17919819	T/C	CC	CC	CC	CC	CC	CC	NN	CC	CC	CC	CC	CC	CC	CC	CC
PV_Br_SNP_0666	A07	18314620	T/G	GG	GG	TG	GG	GG	GG	NN	GG	GG	GG	GG	GG	GG	GG	GG
PV_Br_SNP_1046	A07	20143408	C/T	CT	CC	CC	CT	CT	CT	CC	CC	CC	CC	CT	CC	CT	CC	CC
PV_Br_SNP_0678	A07	20498940	C/T	TT	CC	TT	CT	CT	TT	NN	CT	TT	CC	CC	CT	NN	NN	NN
PV_Br_SNP_0687	A07	22175659	C/A	AA	AA	CC	CA	AA	CA	NN	CA	AA	AA	AA	NN	AA	NN	NN
PV_Br_SNP_0688	A07	22214476	G/A	AA	AA	AA	GA	AA	AA	NN	AA	AA	GA	AA	AA	GA	AA	AA
PV_Br_SNP_0691	A07	22577772	A/T	TT	TT	AA	TA	AA	TA	TT	TT	TT	AA	TA	AA	AA	AA	AA
PV_Br_SNP_0696	A08	368385	C/T	TT	TT	TT	TC	TT	CC	TT	TT	CC	TT	TT	TT	TT	TC	TC
PV_Br_SNP_0697	A08	481952	C/T	NN	NN	CC	TT	TT	NN	NN	NN	TT	NN	NN	NN	TT	TT	NN
PV_Br_SNP_0698	A08	734955	T/C	CC	CC	CC	CC	CC	CC	NN	CC	CC	CC	CC	CC	CC	CC	CC
PV_Br_SNP_0699	A08	912720	A/G	GG	GG	AG	GG	GG	GG	NN	GG	NN	GG	GG	GG	GG	GG	GG
PV_Br_SNP_0709	A08	3109891	T/A	TT	TT	TT	TT	TT	TT	TT	TT	TT	TT	TT	TT	TT	TT	TT
PV_Br_SNP_0710	A08	3527840	C/G	CC	CC	GC	CC	CC	CC	NN	CC	CC	CC	CC	CC	CC	CC	CC
PV_Br_SNP_1066	A08	5049303	A/T	AA	AA	AA	AA	AA	AA	AA	AA	AA	AA	AA	AA	AA	AA	AA
PV_Br_SNP_0721	A08	6333089	C/A	AA	AA	AA	AA	AA	AA	AA	AA	AA	AA	AA	AA	AA	AA	AA
PV_Br_SNP_0722	A08	6547066	C/T	TT	TT	TC	TT	TT	TT	NN	TC	TT	TT	TT	CC	TT	TT	TC
PV_Br_SNP_1061	A08	6622868	G/A	GG	GG	AA	AG	GG	GG	GG	AA	GG	GG	AG	AG	GG	AG	GG
PV_Br_SNP_0725	A08	6780592	A/G	GG	GG	GG	GG	GG	GG	NN	GG	GG	GG	GG	GG	GG	GG	GG
PV_Br_SNP_0729	A08	7381378	G/A	AA	AA	AA	AA	AA	AA	NN	AA	AA	AA	AA	AA	AA	AA	AA
PV_Br_SNP_0743	A08	8978452	A/G	GG	GG	GG	GG	GG	GG	GG	GG	GG	GG	GG	GG	GG	GG	GG
PV_Br_SNP_0744	A08	9046309	A/G	GG	GG	GG	GG	GG	GG	NN	GG	GG	GG	GG	GG	GG	GG	GG
PV_Br_SNP_0745	A08	9356818	A/G	GG	AA	GA	AA	AA	GG	NN	GG	AA	AA	AA	GG	AA	AA	GA
PV_Br_SNP_0746	A08	10076323	T/A	AA	AA	TA	AA	AA	TA	NN	AA	TA	AA	AA	AA	AA	AA	AA
PV_Br_SNP_0754	A08	11236942	T/A	TT	TT	TT	AT	TT	AT	TT	TT	AA	AT	AT	TT	AT	AT	AT
PV_Br_SNP_0760	A08	12781945	A/T	TT	TT	TT	TT	TT	TT	NN	TT	TT	TT	TT	TT	TT	TT	TT
PV_Br_SNP_0761	A08	13413655	C/T	CT	TT	TT	CC	CT	CC	NN	TT	CC	CC	CC	TT	TT	TT	CT
PV_Br_SNP_0762	A08	13487463	G/A	GG	GG	AA	GG	GG	NN	NN	GG	GG	GG	GG	GG	GG	GG	GG
PV_Br_SNP_1062	A08	13615880	A/C	AA	AA	AC	AC	AA	AA	AA	AA	AA	AA	AA	CC	AA	CC	AA
PV_Br_SNP_1072	A08	15753089	C/T	CC	CC	CC	CC	CC	CC	CC	CC	CC	CC	CC	CC	CC	CC	CC
PV_Br_SNP_0776	A08	15885956	G/T	GG	GG	GG	GG	GG	GG	GG	GG	GG	GG	GG	GG	GG	GG	GG
PV_Br_SNP_0778	A08	16910347	G/T	TT	GG	GG	TT	TT	GG	NN	TT	TT	TT	TT	TT	TT	TT	TT
PV_Br_SNP_0779	A08	16953278	A/G	AA	AA	GG	GG	AA	AA	NN	GA	AA	AA	AA	AA	AA	AA	AA
PV_Br_SNP_0783	A08	17556729	T/A	AT	AT	AT	AA	TT	AA	NN	AT	AT	AT	AT	AA	TT	TT	AT
PV_Br_SNP_1041	A08	19024222	C/A	AA	AC	CC	AA	AA	AC	AA	AA	AC	AC	AC	AA	AA	AA	AA
PV_Br_SNP_1037	A08	20713105	G/A	GG	GG	GG	GG	GG	AG	GG	GG	GG	GG	GG	GG	AG	GG	GG
PV_Br_SNP_0790	A08	20974107	A/C	CC	AA	CC	CC	CC	AC	AC	AC	AA	AA	AA	AC	AC	AC	AC
PV_Br_SNP_0792	A08	21364188	C/T	CC	CC	CC	CC	CC	CC	NN	CC	CC	CC	CC	CC	CC	CC	CC
PV_Br_SNP_0795	A09	489884	A/G	AA	AA	AA	AA	AA	AA	AA	AA	AA	AA	AA	AA	AA	AA	AA
PV_Br_SNP_0796	A09	731769	A/G	GG	GG	GG	GG	GG	GG	NN	GG	GG	GG	GG	GG	GG	GG	GG
PV_Br_SNP_0798	A09	966111	C/T	CC	CC	CC	CC	CC	CC	NN	CC	TC	CC	CC	CC	CC	CC	CC
PV_Br_SNP_0800	A09	1112513	C/G	CC	CC	CC	CC	CC	GC	NN	CC	CC	CC	CC	CC	CC	CC	CC
PV_Br_SNP_1083	A09	2867279	C/A	CC	CC	CC	CC	CC	CC	AC	CC	CC	CC	CC	CC	CC	CC	CC
PV_Br_SNP_0809	A09	4162713	A/G	AA	AA	AA	GA	AA	GG	AA	GA	GG	GA	AA	GG	GA	GA	AA
PV_Br_SNP_0810	A09	4305557	G/T	GT	TT	GG	TT	TT	TT	NN	TT	TT	GT	GT	TT	TT	TT	TT
PV_Br_SNP_0811	A09	4460206	A/T	TT	TT	TT	TT	TT	TT	NN	TT	TT	TT	TT	TT	TT	TT	TT
PV_Br_SNP_1071	A09	4475177	T/C	CC	CC	CT	CC	CC	CT	TT	CC	CC	CC	CC	CC	CC	CC	CC
PV_Br_SNP_0812	A09	4959336	A/G	AA	AA	GG	GG	AA	NN	NN	GG	AA	GG	GG	NN	GG	AA	GG
PV_Br_SNP_1049	A09	5177858	A/G	AG	AA	AG	AG	AG	AG	AA	AG	AG	AA	AA	AG	AG	AG	AG
PV_Br_SNP_1044	A09	7789464	A/T	TT	AT	TT	TT	TT	TT	NN	AT	AA	NN	TT	AA	AA	AT	AA
PV_Br_SNP_0826	A09	8726675	C/A	CC	CC	CC	CC	CC	CC	CC	CC	CC	CC	CC	CC	CC	CC	CC
PV_Br_SNP_0827	A09	9563894	C/G	GG	CG	GG	GG	CC	GG	NN	CG	GG	CC	CC	GG	CC	CG	GG
PV_Br_SNP_0828	A09	9652821	T/C	TC	TC	TC	TC	TC	TC	NN	TC	TC	TC	TC	TC	TC	TC	TC
PV_Br_SNP_0839	A09	11475911	A/T	NN	NN	TT	NN	NN	NN	NN	NN	NN	NN	NN	NN	NN	NN	AA
PV_Br_SNP_0848	A09	13502218	T/C	CC	CC	CC	CC	CC	TT	CC	TT	TT	CC	CC	TC	CC	CC	CC
PV_Br_SNP_0850	A09	15420007	T/C	TT	TT	TT	TT	TT	TT	NN	TT	TT	TT	TT	TT	TT	TT	TT
PV_Br_SNP_0851	A09	15638572	T/G	TT	TT	TT	TT	TT	TT	NN	TT	TT	TT	TT	TT	TT	TT	TT
PV_Br_SNP_0855	A09	17812533	C/T	CC	CC	CC	CC	CC	CC	NN	CC	TT	CC	CC	CC	CC	CC	CC
PV_Br_SNP_0865	A09	19518904	A/G	GG	AG	GG	GG	AG	GG	AA	AG	GG	GG	GG	GG	AG	GG	GG
PV_Br_SNP_0867	A09	21654202	C/G	CC	CC	GG	CC	CC	CC	NN	CC	CC	CG	CG	CC	CC	CC	CC
PV_Br_SNP_0868	A09	22147801	A/G	GG	GG	NN	GG	GG	GG	NN	GG	GG	GG	GG	GG	GG	GG	GG
PV_Br_SNP_0869	A09	22335708	C/G	CC	CC	CC	CC	CC	CC	NN	CC	CC	CC	CC	CC	CC	CC	CC
PV_Br_SNP_0879	A09	25766864	A/G	GG	GG	GA	GG	GG	GA	AA	GA	GA	AA	GG	GG	GG	GG	GG
PV_Br_SNP_0882	A09	26952124	T/C	TC	TC	TC	CC	TT	CC	NN	TC	TT	CC	CC	CC	TT	CC	TC
PV_Br_SNP_0883	A09	27132534	G/C	GG	GG	GG	GG	GG	GG	NN	GG	GG	GG	GG	GG	GG	GG	GG
PV_Br_SNP_0889	A09	27875429	A/T	AT	TT	AA	TT	TT	AA	NN	TT	AT	AT	AT	TT	AT	AT	TT
PV_Br_SNP_0902	A09	32116439	T/A	TA	TA	TA	TA	TT	TT	TT	TA	TT	TA	TT	TA	TT	TT	TT
PV_Br_SNP_0904	A09	32560861	A/G	NN	GG	GG	GG	GG	NN	NN	GG	GG	GG	GG	NN	GG	GG	GG
PV_Br_SNP_0906	A09	33028118	G/A	GG	AG	AG	AG	GG	AG	NN	AG	AG	GG	GG	GG	GG	GG	GG
PV_Br_SNP_0907	A09	33253584	A/G	GG	GG	GG	GG	GG	GG	NN	GG	GG	GG	GG	GG	GG	GG	GG
PV_Br_SNP_1038	A09	35628753	A/C	AA	AA	CC	AA	AA	AA	CC	AA	AA	AA	AA	AA	AA	AA	AA
PV_Br_SNP_0918	A09	36554283	T/G	GG	GG	GG	GG	GG	GG	GG	GG	GG	GG	GG	GG	GG	GG	GG
PV_Br_SNP_0919	A09	36612655	T/C	TT	TT	TT	TT	TT	TT	NN	TT	TT	TT	TT	TT	TT	TT	TT
PV_Br_SNP_0920	A09	37061247	G/T	GG	GG	GG	GG	GG	GG	NN	GG	GG	GG	GG	GG	GG	GG	GG
PV_Br_SNP_0921	A10	592464	C/T	CC	CC	CC	CC	CC	CC	CC	CC	CC	CC	CC	CC	CC	CC	CC
PV_Br_SNP_0924	A10	1550777	C/A	CA	CC	CA	CC	CC	CC	NN	CC	CC	CC	CC	CC	CC	CC	CA
PV_Br_SNP_1067	A10	3312620	C/G	GG	GG	GG	GG	GG	GG	GG	GG	GG	GG	GG	GG	GG	GG	GG
PV_Br_SNP_1068	A10	3313214	A/G	GG	GG	GG	GG	GG	GG	GG	GG	GG	GG	GG	GG	GG	GG	GG
PV_Br_SNP_0931	A10	3904244	T/G	GG	GG	GG	GG	GG	GG	NN	GG	GG	GG	GG	GG	GG	GG	GG
PV_Br_SNP_0939	A10	6064111	A/G	AG	AG	AG	AG	AG	AG	NN	AG	AG	AG	AG	AG	AG	AG	AA
PV_Br_SNP_0951	A10	9526129	A/G	GG	GG	GG	GG	GG	GG	GG	GG	GG	GG	GG	GG	GG	GG	GG
PV_Br_SNP_0952	A10	9623892	A/C	AA	AC	CC	AC	AA	AA	NN	AA	AA	AA	AA	AA	AA	AC	AC
PV_Br_SNP_0954	A10	10451842	A/G	GG	AA	GG	NN	NN	AA	NN	GG	GG	AA	AA	AG	AA	AG	AG
PV_Br_SNP_0955	A10	10551658	T/G	GG	GG	TT	TG	GG	TG	NN	TG	GG	TT	TT	GG	TT	TG	GG
PV_Br_SNP_0958	A10	11191128	T/G	TT	TT	TT	TT	TT	TT	TT	TT	TT	TT	TT	TT	TT	TT	TT
PV_Br_SNP_0959	A10	11287063	T/C	CC	CC	CC	CC	CC	CC	NN	CC	CC	CC	CC	CC	CC	CC	CC
PV_Br_SNP_0960	A10	11370494	G/A	NN	NN	AA	NN	AA	NN	NN	NN	AA	NN	NN	GG	AA	AA	GG
PV_Br_SNP_0966	A10	11950431	T/C	TT	TC	TT	TT	TT	TC	TC	TC	TT	TT	TT	TT	TT	TT	TT
PV_Br_SNP_0968	A10	12106839	T/C	TT	TT	TT	CC	TT	TT	NN	TT	CC	TT	TT	CC	TT	TT	CC
PV_Br_SNP_0969	A10	12179004	G/T	NN	TT	GG	GG	TT	NN	NN	NN	NN	TT	NN	NN	GG	NN	NN
PV_Br_SNP_0970	A10	12295861	T/C	TT	CC	TC	TC	TC	TC	NN	CC	TC	TC	TC	TT	CC	TC	TT
PV_Br_SNP_0974	A10	13246082	C/A	CC	CC	CC	CC	CC	CC	NN	CC	CC	CC	NN	CC	CC	CC	CC
PV_Br_SNP_0975	A10	13638161	C/T	TT	TT	CT	TT	TT	TT	NN	TT	TT	TT	TT	TT	TT	TT	CT
PV_Br_SNP_0976	A10	13973332	A/G	GG	GG	GG	GG	GG	GG	NN	GG	GG	GG	GG	GG	GG	GG	GG
PV_Br_SNP_0980	A10	14708300	T/A	AA	AA	AA	AA	AA	AA	NN	AA	AA	AA	AA	AA	AA	AA	AA
PV_Br_SNP_0985	A10	15707133	T/G	TT	TT	TT	TT	TT	TT	TT	TT	TT	TT	TT	TT	TT	TT	TT
PV_Br_SNP_0986	A10	15817352	G/C	GG	CG	GG	GG	GG	GG	NN	GG	GG	GG	GG	GG	GG	GG	GG
PV_Br_SNP_0987	A10	15853544	G/A	AA	AA	GA	AA	AA	GG	NN	AA	AA	AA	AA	AA	AA	AA	AA
PV_Br_SNP_0989	A10	16000038	T/C	TT	TT	TT	TT	TT	NN	NN	TT	TT	TT	TT	TT	TT	TT	TT
PV_Br_SNP_0996	A10	16818623	A/G	AA	GG	AA	AA	AA	AA	AA	AA	GA	AA	GA	AA	AA	AA	AA
PV_Br_SNP_0998	A10	17237508	T/G	TT	TT	GG	TT	TT	TT	NN	TT	TT	TT	TT	TT	TT	GT	TT
PV_Br_SNP_0999	A10	17368173	C/T	TT	TT	CT	TT	TT	TT	NN	TT	TT	TT	TT	TT	TT	TT	TT
PV_Br_SNP_1053	Scaffold00030[illegible]	8568	G/A	GG	AG	AG	AG	GG	AG	AG	AG	AG	AA	AG	GG	AA	AA	GG
PV_Br_SNP_1054	Scaffold00030[illegible]	9355	G/C	GG	CG	CG	CG	GG	CG	CG	CG	CG	CC	CG	CG	CC	CC	GG

SNP marker ID	FT_051	FT_056	FT_086	FT_088	FT_097	SM_14	SM_15	SM_16	SM_17	SM_18	SM_19	SM_20	T_panca[illegible]	T_RR	VT_006	VT_007	VT_008	VT_009	VT_010	VT_011	VT_012	VT_013	VT_014	VT_015
PV_Br_SNP_1069	AA	GG	AG	AG	AA	NN	NN	NN	NN	NN	NN	NN	NN	NN	AA	AA	AA	AA	AA	AA	AA	AA	AG	AA
PV_Br_SNP_1047	CG	CC	CG	CC	CC	NN	NN	NN	NN	NN	NN	NN	NN	NN	CC	CC	CC	GG	CC	CC	GG	CG	CG	CG
PV_Br_SNP_0607	CC	CC	CC	CC	CC	NN	NN	NN	NN	NN	NN	NN	NN	NN	CC	CC	CC	CC	CC	CC	CC	TC	CC	CC
PV_Br_SNP_0609	GG	GG	NN	GG	NN	GG	GG	GG	GG	GG	GG	GG	GG	GG	GG	GG	GG	GG	GG	GG	GG	GG	GG	GG
PV_Br_SNP_0610	GG	NN	AA	GG	NN	GG	GG	GG	GG	GG	GG	GG	GG	AA	GG	GG	GG	GG	GG	GG	AG	GG	GG	NN
PV_Br_SNP_0611	TT	TT	CC	TT	NN	NN	TT	NN	TT	TT	TT	TT	NN	TT	NN	TT	TT	NN	TT	TT	TT	TT	NN	TT
PV_Br_SNP_0616	AA	GG	AA	GG	GG	NN	NN	NN	NN	NN	NN	NN	NN	NN	GG	AA	GG	AA	AA	GG	AA	AA	GG	GG
PV_Br_SNP_0620	GG	GT	GG	GT	NN	GT	GG	TT	GG	GG	GT	GG	TT	TT	GG	GG	GG	GT	GT	GG	GG	TT	GG	GG
PV_Br_SNP_0622	TT	NN	TT	GG	NN	TT	GG	TT	GG	NN	TT	GG	TT	TT	GG	GG	GT	TT	GT	TT	TT	TT	GG	GT
PV_Br_SNP_0637	AA	AA	AA	AA	AA	NN	NN	NN	NN	NN	NN	NN	NN	NN	AA	AA	AA	AA	AA	AA	AA	AA	AA	AA
PV_Br_SNP_0641	CC	CT	NN	CC	NN	CT	CC	CT	CC	CC	CT	TT	CC	CT	CC	CC	CT	TT	CC	CC	CT	CT	CT	CT
PV_Br_SNP_0645	TT	NN	TT	TT	NN	GT	GT	GT	TT	TT	TT	TT	TT	TT	GT	GT	GT	TT	TT	TT	TT	TT	GT	GT
PV_Br_SNP_0646	AA	GA	AA	GA	NN	GA	AA	AA	AA	AA	AA	GG	AA	AA	AA	GG	GG	AA	GA	AA	AA	GA	AA	AA
PV_Br_SNP_0651	CT	TT	TT	NN	TT	NN	NN	NN	NN	NN	NN	NN	NN	NN	TT	NN	CC	CC	TT	TT	CC	CC	TT	TT
PV_Br_SNP_0653	GG	GG	GG	GG	NN	GG	GG	GG	GG	GG	GG	GG	GG	GG	GG	GG	GG	GG	GG	GG	GG	GG	GG	GG
PV_Br_SNP_0656	TT	TT	TT	TT	NN	TT	TT	TT	TT	TT	TT	TT	CT	TT	TT	NN	TT	CC	TT	TT	TT	TT	TT	TT
PV_Br_SNP_0657	CG	GG	GG	GG	NN	GG	GG	GG	GG	CC	GG	GG	GG	GG	NN	CG	GG	GG	GG	GG	GG	NN	GG	GG
PV_Br_SNP_0663	TT	TT	TT	TT	TT	NN	NN	NN	NN	NN	NN	NN	NN	NN	TT	TT	TT	TT	TT	TT	TT	AT	TT	TT
PV_Br_SNP_0664	CC	CC	CC	CC	NN	CC	CC	CC	CC	CC	CC	CC	CC	CC	NN	NN	CC	CC	CC	CC	CC	CC	CC	CC
PV_Br_SNP_0665	NN	CC	CC	CC	NN	CC	CC	CC	CC	CC	CC	CC	CC	CC	TC	TC	CC	CC	CC	CC	CC	CC	CC	CC
PV_Br_SNP_0666	GG	GG	TT	GG	NN	GG	GG	GG	GG	GG	GG	GG	GG	GG	GG	GG	GG	GG	GG	GG	TT	TT	GG	GG
PV_Br_SNP_1046	CC	CC	CC	CC	CC	NN	NN	NN	NN	NN	NN	NN	NN	NN	TT	CC	CT	TT	CC	CC	CC	TT	CC	CC
PV_Br_SNP_0678	CT	CT	NN	CC	NN	TT	CT	CT	CT	CT	TT	CT	CT	CT	NN	NN	CT	TT	NN	NN	NN	NN	TT	TT
PV_Br_SNP_0687	NN	AA	NN	AA	NN	AA	CA	CA	AA	AA	AA	CA	AA	AA	NN	NN	NN	NN	NN	NN	NN	NN	NN	AA
PV_Br_SNP_0688	GA	AA	AA	GA	NN	GA	AA	GA	AA	AA	AA	AA	AA	AA	GA	AA	GA	AA	GA	AA	GA	AA	AA	AA
PV_Br_SNP_0691	NN	AA	AA	TT	TA	NN	NN	NN	NN	NN	NN	NN	NN	NN	AA	TA	TA	AA	TA	AA	TA	AA	TT	TA
PV_Br_SNP_0696	CC	TC	TT	TT	TT	NN	NN	NN	NN	NN	NN	NN	NN	NN	TT	TT	CC	CC	TT	TT	TT	TT	TT	TT
PV_Br_SNP_0697	NN	TT	TT	NN	NN	NN	NN	NN	NN	NN	NN	NN	CC	CC	CC	CC	CC	NN	TT	NN	CC	CC	NN	NN
PV_Br_SNP_0698	CC	CC	TC	CC	NN	CC	TC	CC	CC	CC	CC	CC	TC	TC	CC	TC	TT	TT	CC	CC	TC	CC	CC	CC
PV_Br_SNP_0699	AA	GG	GG	GG	NN	GG	GG	GG	GG	GG	GG	GG	GG	GG	AG	AG	GG	GG	GG	GG	GG	GG	GG	GG
PV_Br_SNP_0709	NN	TT	TT	TT	TT	NN	NN	NN	NN	NN	NN	NN	NN	NN	TT	TT	TT	TT	TT	TT	TT	TT	TT	TT
PV_Br_SNP_0710	CC	CC	CC	CC	NN	CC	CC	CC	CC	CC	CC	CC	CC	CC	GC	GC	CC	CC	CC	CC	CC	CC	CC	CC
PV_Br_SNP_1066	AA	AA	AA	AA	AA	NN	NN	NN	NN	NN	NN	NN	NN	NN	AA	AA	AA	AA	AA	AA	AA	AA	AA	AA
PV_Br_SNP_0721	AA	AA	AA	AA	AA	NN	NN	NN	NN	NN	NN	NN	NN	NN	AA	AA	AA	CA	AA	AA	AA	CA	AA	AA
PV_Br_SNP_0722	TT	TT	TT	TT	NN	TT	TT	TT	TC	TC	TT	TT	TT	TC	TT	TT	TT	TT	CC	TT	TT	TC	TC	TT
PV_Br_SNP_1061	GG	GG	AG	GG	AG	NN	NN	NN	NN	NN	NN	NN	NN	NN	GG	GG	GG	GG	GG	GG	GG	AG	GG	GG
PV_Br_SNP_0725	AA	GG	AA	GG	NN	GG	GG	GG	GG	GG	GG	GG	GG	GG	GG	GG	GG	GG	GG	GG	GG	GG	GG	GG
PV_Br_SNP_0729	GA	AA	AA	AA	NN	AA	AA	AA	AA	AA	AA	AA	AA	AA	GA	GA	AA	AA	AA	AA	AA	AA	AA	AA
PV_Br_SNP_0743	AG	GG	GG	GG	GG	NN	NN	NN	NN	NN	NN	NN	NN	NN	GG	GG	GG	GG	GG	GG	AG	GG	GG	GG
PV_Br_SNP_0744	GG	GG	GG	GG	NN	GG	GG	GG	GG	GG	GG	GG	GG	GG	AG	AG	GG	GG	GG	GG	GG	GG	GG	GG
PV_Br_SNP_0745	GA	AA	AA	GG	NN	GG	GA	AA	AA	AA	AA	NN	GA	GG	GA	GG	GG	AA	AA	AA	AA	AA	GA	GA
PV_Br_SNP_0746	AA	AA	TT	AA	NN	TA	AA	TT	AA	AA	TT	AA	TT	TA	TT	TA	TT	TA	AA	AA	TA	TT	TA	AA
PV_Br_SNP_0754	TT	TT	AT	TT	TT	NN	NN	NN	NN	NN	NN	NN	NN	NN	TT	AT	AT	TT	TT	AT	AT	AT	AT	AT
PV_Br_SNP_0760	TT	TT	TT	TT	NN	TT	TT	TT	TT	TT	TT	TT	TT	TT	AT	AT	TT	TT	TT	TT	TT	TT	TT	TT
PV_Br_SNP_0761	CT	CC	TT	CC	NN	CC	CT	CC	TT	CT	CC	CC	CC	TT	TT	CT	TT	TT	NN	NN	NN	NN	CT	CT
PV_Br_SNP_0762	NN	NN	GG	GG	NN	GG	GG	GG	NN	GG	GG	NN	GG	AA	AG	AG	AA	NN	GG	NN	NN	NN	GG	GG
PV_Br_SNP_1062	AC	AA	AA	AC	AC	NN	NN	NN	NN	NN	NN	NN	NN	NN	AA	AA	AC	AC	AA	CC	AA	AC	CC	AA
PV_Br_SNP_1072	CC	CC	CC	CC	CC	NN	NN	NN	NN	NN	NN	NN	NN	NN	CC	CC	CC	CC	CC	CC	CC	CC	CC	CC
PV_Br_SNP_0776	GG	GG	GG	GG	GG	NN	NN	NN	NN	NN	NN	NN	NN	NN	GG	GG	GG	GG	GG	GG	GG	GG	GG	GG
PV_Br_SNP_0778	TT	GG	TT	TT	NN	GG	GG	TT	TT	GG	GG	TT	GG	TT	TT	TT	TT	TT	NN	NN	NN	NN	TG	TT
PV_Br_SNP_0779	AA	AA	AA	GG	NN	AA	AA	AA	AA	AA	AA	AA	AA	AA	AA	AA	AA	AA	AA	AA	AA	AA	GG	GA
PV_Br_SNP_0783	TT	AA	TT	AT	NN	AT	AA	AA	AT	AA	TT	TT	AA	AA	AA	AT	TT	TT	TT	TT	AT	TT	TT	AA
PV_Br_SNP_1041	AA	AC	AA	AA	AA	NN	NN	NN	NN	NN	NN	NN	NN	NN	AA	AA	AA	AA	AA	AA	AA	AA	AA	AA
PV_Br_SNP_1037	GG	GG	GG	GG	GG	NN	NN	NN	NN	NN	NN	NN	NN	NN	GG	GG	AG	GG	GG	AG	AA	GG	GG	GG
PV_Br_SNP_0790	CC	AC	AC	AA	CC	NN	NN	NN	NN	NN	NN	NN	NN	NN	AA	NN	CC	CC	AC	AA	CC	CC	CC	AC
PV_Br_SNP_0792	CC	CC	CC	CC	NN	TT	CC	CC	CC	CC	CC	CC	CC	CC	CC	CC	CC	CC	CC	CC	CC	TC	CC	CC
PV_Br_SNP_0795	AA	AA	AA	AA	AA	NN	NN	NN	NN	NN	NN	NN	NN	NN	AA	AA	AA	AA	AA	AA	AA	AA	AA	AA
PV_Br_SNP_0796	GG	AG	AG	GG	NN	GG	GG	GG	GG	GG	GG	GG	AG	GG	AG	AG	GG	GG	GG	GG	GG	GG	GG	GG
PV_Br_SNP_0798	CC	CC	CC	CC	NN	CC	CC	CC	CC	CC	CC	CC	CC	TC	CC	TT	TC	CC	CC	TT	TT	TC	CC	TC
PV_Br_SNP_0800	CC	CC	CC	CC	NN	CC	CC	CC	CC	CC	CC	CC	CC	CC	CC	CC	CC	CC	CC	CC	CC	CC	CC	CC
PV_Br_SNP_1083	CC	CC	CC	CC	CC	NN	NN	NN	NN	NN	NN	NN	NN	NN	CC	CC	CC	CC	CC	CC	CC	CC	CC	CC
PV_Br_SNP_0809	AA	AA	AA	GA	GA	NN	NN	NN	NN	NN	NN	NN	NN	NN	AA	GA	AA	AA	GG	AA	AA	GA	GA	GA
PV_Br_SNP_0810	GT	GT	GT	TT	NN	TT	TT	GT	TT	TT	TT	TT	GG	GT	GT	GT	GG	GT	TT	TT	GG	GT	TT	TT
PV_Br_SNP_0811	TT	TT	TT	TT	NN	TT	TT	TT	TT	TT	TT	TT	TT	TT	TT	TT	TT	TT	TT	TT	TT	TT	TT	TT
PV_Br_SNP_1071	CC	CC	CC	CC	CC	NN	NN	NN	NN	NN	NN	NN	NN	NN	CC	CC	CC	CT	CC	CC	CC	CT	CC	CC
PV_Br_SNP_0812	AA	GG	NN	NN	NN	NN	NN	GG	NN	AA	NN	GG	GG	GG	GG	AA	GG	GG	NN	NN	AA	GG	GG	GG
PV_Br_SNP_1049	AA	AA	AG	AG	AG	NN	NN	NN	NN	NN	NN	NN	NN	NN	AG	AA	AA	AG	AG	AG	AA	AG	AG	AA
PV_Br_SNP_1044	AA	AT	NN	AT	NN	AA	AA	AA	AA	NN	AA	AA	AT	AT	AA	NN	TT	TT	AA	AT	TT	AT	AT	AT
PV_Br_SNP_0826	CC	CC	CC	CC	CC	NN	NN	NN	NN	NN	NN	NN	NN	NN	CC	CC	CC	CC	CC	CC	CC	CC	CC	CC
PV_Br_SNP_0827	CG	GG	GG	CG	NN	GG	CG	GG	GG	GG	CG	GG	CG	GG	CG	GG	GG	GG	CC	CC	CG	GG	CC	GG
PV_Br_SNP_0828	TC	TC	TT	TC	NN	TC	TC	TC	TC	TC	TC	TT	TC	TC	TC	TC	TC	TC	TC	TC	TC	TC	TC	TC
PV_Br_SNP_0839	TT	TT	AT	TT	NN	NN	NN	TT	AA	NN	NN	NN	TT	TT	AA	AA	NN	TT	TT	NN	TT	TT	TT	NN
PV_Br_SNP_0848	TT	NN	CC	TT	CC	NN	NN	NN	NN	NN	NN	NN	NN	NN	CC	TT	TC	TC	CC	TT	CC	CC	CC	TT
PV_Br_SNP_0850	TT	TT	NN	TT	NN	TT	TT	TT	TT	TT	TT	TT	TT	TT	TT	TT	TT	TT	TT	TT	TT	TT	TT	TT
PV_Br_SNP_0851	NN	TT	TT	GT	NN	TT	TT	TT	TT	TT	TT	TT	TT	TT	TT	TT	TT	TT	TT	TT	NN	TT	TT	TT
PV_Br_SNP_0855	CC	CC	CC	CC	NN	CC	CC	CC	CC	CC	TC	CC	CC	CC	CC	CC	TT	CC	CC	CC	CC	CC	CC	CC
PV_Br_SNP_0865	AA	GG	AG	AG	GG	NN	NN	NN	NN	NN	NN	NN	NN	NN	AG	AA	AG	AA	GG	GG	AA	AA	GG	GG
PV_Br_SNP_0867	CG	CG	CC	CC	NN	GG	CC	CG	CC	CC	CC	CC	GG	CG	CC	CG	CC	GG	CC	CG	CC	CG	CC	CC
PV_Br_SNP_0868	GG	GG	GG	GG	NN	GG	GG	GG	GG	GG	GG	NN	GG	GG	AG	AG	GG	GG	GG	GG	GG	GG	GG	GG
PV_Br_SNP_0869	CC	CC	CC	CC	NN	CC	CC	CC	CC	CC	CC	CC	CC	CC	GC	GC	CC	CC	CC	CC	GC	CC	CC	CC
PV_Br_SNP_0879	GA	GG	GG	GA	GA	NN	NN	NN	NN	NN	NN	NN	NN	NN	AA	AA	AA	GA	GG	GG	AA	GG	GA	GA
PV_Br_SNP_0882	TT	TT	TT	TC	NN	TT	TT	TT	TC	TT	TC	TT	CC	TC	TC	TT	TT	CC	CC	CC	TC	TT	CC	TT
PV_Br_SNP_0883	GG	GG	GG	GG	NN	GG	GG	GG	GG	GG	GG	GG	GG	GG	GG	GG	GG	GG	GG	GG	GG	GG	GG	GG
PV_Br_SNP_0889	AT	TT	AT	TT	NN	TT	AT	TT	TT	TT	TT	TT	TT	TT	TT	TT	TT	TT	TT	AA	TT	AA	TT	AT
PV_Br_SNP_0902	TT	TT	TT	TT	TT	NN	NN	NN	NN	NN	NN	NN	NN	NN	TA	TT	TT	AA	TT	TT	AA	AA	TA	TA
PV_Br_SNP_0904	GG	GG	NN	GG	NN	NN	GG	GG	NN	GG	NN	NN	GG	AA	AG	AA	GG	GG	AG	GG	AG	AG	GG	GG
PV_Br_SNP_0906	AG	AG	GG	GG	NN	GG	GG	AA	GG	GG	GG	GG	GG	AG	AG	AG	AG	AG	AA	GG	AG	GG	AG	GG
PV_Br_SNP_0907	GG	GG	GG	GG	NN	GG	GG	GG	GG	GG	GG	GG	GG	GG	GG	GG	GG	GG	GG	GG	GG	GG	GG	GG
PV_Br_SNP_1038	AC	AA	AA	AA	AC	NN	NN	NN	NN	NN	NN	NN	NN	NN	AA	AA	AA	AA	AC	AA	AA	AA	AA	CC
PV_Br_SNP_0918	GG	GG	GG	GG	GG	NN	NN	NN	NN	NN	NN	NN	NN	NN	GG	GG	GG	GG	GG	GG	GG	TT	GG	GG
PV_Br_SNP_0919	TT	TT	TT	TT	NN	TT	TT	TT	TT	TT	TT	TT	TT	TT	CT	CT	TT	TT	TT	TT	TT	TT	TT	TT
PV_Br_SNP_0920	GG	GG	GG	GG	NN	GG	GG	GG	GG	GG	GG	GG	GG	GG	TG	TG	GG	TT	GG	GG	GG	TG	GG	GG
PV_Br_SNP_0921	CC	CC	CC	CC	CC	NN	NN	NN	NN	NN	NN	NN	NN	NN	CC	CC	CC	CC	CC	CC	CC	CC	CC	CC
PV_Br_SNP_0924	CC	CC	CC	CA	NN	CC	CC	AA	CC	CC	CC	CC	AA	CA	CC	CC	CC	CC	CC	CC	CA	CC	CC	CC
PV_Br_SNP_1067	GG	GG	GG	GG	GG	NN	NN	NN	NN	NN	NN	NN	NN	NN	GG	GG	GG	GG	GG	GG	GG	GG	GG	GG
PV_Br_SNP_1068	GG	GG	GG	GG	GG	NN	NN	NN	NN	NN	NN	NN	NN	NN	GG	GG	GG	GG	GG	GG	GG	GG	GG	GG
PV_Br_SNP_0931	GG	GG	TG	GG	NN	GG	GG	GG	GG	GG	GG	GG	GG	GG	GG	GG	GG	GG	GG	GG	GG	TG	GG	GG
PV_Br_SNP_0939	AG	AG	AG	AG	NN	AG	AG	AA	AA	AA	AG	AA	GG	AG	AA	AG	GG	AG	AG	AA	AA	AA	AG	AG
PV_Br_SNP_0951	GG	GG	GG	GG	GG	NN	NN	NN	NN	NN	NN	NN	NN	NN	GG	AG	AG	GG	GG	GG	AA	AG	GG	GG
PV_Br_SNP_0952	AA	CC	CC	AA	NN	AA	AC	CC	AA	AA	AA	AA	AA	AC	NN	CC	AC	AA	AA	AA	CC	CC	AA	AA
PV_Br_SNP_0954	AA	AG	AA	AG	NN	GG	GG	AA	GG	GG	GG	GG	GG	AG	AG	AG	AA	AG	AG	AA	AA	AA	AA	AG
PV_Br_SNP_0955	GG	TG	GG	TG	NN	GG	GG	GG	GG	TG	GG	GG	GG	TG	TG	TG	GG	GG	GG	TT	GG	TG	TG	TG
PV_Br_SNP_0958	TT	TT	TT	TT	TT	NN	NN	NN	NN	NN	NN	NN	NN	NN	TT	TT	TT	TT	TT	TT	TT	GT	TT	TT
PV_Br_SNP_0959	CC	CC	TC	CC	NN	CC	CC	TT	CC	CC	CC	CC	CC	CC	TC	TC	TT	TC	CC	CC	TT	TC	CC	CC
PV_Br_SNP_0960	AA	AA	NN	AA	NN	AA	GA	GG	GG	AA	AA	AA	AA	GA	GG	GG	GG	GA	AA	NN	GG	GA	AA	AA
PV_Br_SNP_0966	TC	TT	TT	TT	TT	NN	NN	NN	NN	NN	NN	NN	NN	NN	NN	TT	CC	CC	TT	CC	CC	CC	TT	TT
PV_Br_SNP_0968	TT	TT	TT	TT	NN	TT	TT	TT	TT	TT	TT	TT	TT	TT	TT	TT	TT	TT	TT	TT	TT	TT	TT	TT
PV_Br_SNP_0969	TT	TT	GG	GG	NN	GG	NN	GG	GG	GG	GG	TT	TT	TT	GG	GG	NN	TT	GG	GG	GG	GT	TT	NN
PV_Br_SNP_0970	CC	CC	TT	TC	NN	TC	TC	TT	TC	NN	TT	CC	CC	CC	TC	TT	TT	TC	TC	TC	CC	CC	TT	TC
PV_Br_SNP_0974	CC	CC	CC	CC	CC	NN	NN	NN	NN	NN	NN	NN	NN	NN	CC	NN	CC	CC	CC	CC	CC	CC	CC	CC
PV_Br_SNP_0975	CT	TT	TT	TT	NN	TT	TT	TT	TT	TT	TT	TT	TT	TT	CT	CT	TT	CT	TT	TT	TT	TT	TT	TT
PV_Br_SNP_0976	AG	GG	GG	GG	NN	GG	GG	GG	GG	GG	GG	GG	GG	GG	GG	AG	GG	GG	GG	GG	GG	GG	GG	GG
PV_Br_SNP_0980	AA	AA	NN	AA	NN	AA	AA	AA	AA	AA	AA	AA	AA	AA	AA	AA	AA	TA	AA	AA	AA	AA	AA	AA
PV_Br_SNP_0985	TT	TT	TT	TT	TT	NN	NN	NN	NN	NN	NN	NN	NN	NN	TT	TT	TT	TT	TT	TT	TT	TT	TT	TT
PV_Br_SNP_0986	GG	GG	GG	GG	NN	GG	GG	GG	GG	GG	GG	GG	GG	CG	CG	CG	GG	CC	GG	GG	CC	CC	GG	GG
PV_Br_SNP_0987	AA	GA	AA	AA	NN	AA	AA	AA	AA	AA	AA	AA	GG	GA	NN	NN	AA	AA	AA	AA	AA	AA	AA	AA
PV_Br_SNP_0989	TT	TT	TT	TT	NN	TT	TT	TT	TT	TT	TT	TT	TT	TT	CT	CT	TT	TT	TT	TT	TT	TT	TT	TT
PV_Br_SNP_0996	AA	AA	AA	GG	AA	NN	NN	NN	NN	NN	NN	NN	NN	NN	AA	AA	AA	GA	AA	AA	AA	AA	AA	AA
PV_Br_SNP_0998	TT	TT	TT	TT	NN	TT	TT	GT	TT	TT	TT	TT	TT	GT	GT	GT	TT	GT	TT	TT	GT	GT	TT	TT
PV_Br_SNP_0999	CT	TT	CC	TT	NN	TT	TT	TT	TT	TT	TT	TT	TT	TT	CT	CT	TT	TT	TT	TT	TT	CT	TT	TT
PV_Br_SNP_1053	AG	GG	AG	GG	AG	NN	NN	NN	NN	NN	NN	NN	NN	NN	GG	AG	AG	AA	GG	GG	GG	AA	GG	AG
PV_Br_SNP_1054	CG	CG	CG	CG	CG	NN	NN	NN	NN	NN	NN	NN	NN	NN	GG	CG	CG	CC	GG	GG	GG	CC	GG	CG

SNP marker ID	VT_017	VT_018	VT_044	VT_045	VT_052	VT_053	VT_089	VT_090	VT_091	VT_092	VT_115	VT_116	VT_117	VT_119	VT_120	VT_123	VT_137
PV_Br_SNP_1069	AA	AA	AA	AA	AA	AG	AA	AA	AA	AA	AA	AG	AA	AA	NN	AA	AA
PV_Br_SNP_1047	CC	CC	CC	CG	CC	CG	CC	CC	CC	CC	GG	GG	CC	CG	NN	GG	CC
PV_Br_SNP_0607	CC	CC	CC	CC	CC	CC	CC	CC	CC	CC	CC	CC	TC	NN	NN	CC	CC
PV_Br_SNP_0609	GG	GG	GG	GG	GG	GG	GG	NN	GG	GG	GG	GG	GG	GG	GG	GG	GG
PV_Br_SNP_0610	GG	GG	GG	GG	GG	GG	GG	NN	NN	GG	NN	AG	AG	GG	GG	GG	GG
PV_Br_SNP_0611	TT	TT	TT	TT	TT	TT	TT	NN	TT	TT	TT	CT	CT	TT	TT	NN	NN
PV_Br_SNP_0616	GG	GG	GG	GG	GG	AA	GG	AA	AA	GG	AA	GG	AA	GG	NN	AA	GG
PV_Br_SNP_0620	GG	GG	GG	GG	GG	GG	GG	NN	GT	GG	GT	GT	GT	GT	GG	NN	GG
PV_Br_SNP_0622	GG	GG	GG	GG	GT	NN	GT	NN	TT	GT	TT	TT	TT	GT	GT	NN	NN
PV_Br_SNP_0637	AA	AA	AA	AA	GA	AA	AA	AA	AA	AA	AA	AA	AA	AA	NN	AA	AA
PV_Br_SNP_0641	CC	NN	CC	NN	CC	NN	NN	NN	NN	CC	TT	NN	NN	CC	NN	NN	CC
PV_Br_SNP_0645	TT	TT	TT	TT	TT	GT	TT	NN	TT	TT	TT	TT	GT	TT	TT	TT	TT
PV_Br_SNP_0646	AA	GA	AA	AA	GA	AA	AA	NN	AA	AA	GA	AA	AA	AA	GG	AA	AA
PV_Br_SNP_0651	NN	TT	NN	TT	CC	NN	TT	TT	TT	NN	TT	CC	CC	TT	NN	CT	CC
PV_Br_SNP_0653	GG	GG	GG	GG	GG	GG	GG	NN	GG	GG	TG	GG	GG	GG	GG	GG	GG
PV_Br_SNP_0656	TT	TT	TT	TT	TT	TT	TT	NN	TT	TT	TT	TT	TT	TT	TT	TT	TT
PV_Br_SNP_0657	GG	GG	CC	GG	GG	GG	GG	NN	GG	GG	GG	GG	GG	GG	GG	GG	CG
PV_Br_SNP_0663	TT	TT	AT	TT	TT	TT	TT	TT	TT	TT	AT	TT	TT	NN	NN	TT	TT
PV_Br_SNP_0664	CC	CC	CC	CC	CC	CC	CC	NN	CC	CC	CC	CC	CC	CC	CC	CC	CC
PV_Br_SNP_0665	CC	CC	CC	CC	CC	CC	CC	NN	CC	CC	CC	CC	CC	CC	CC	CC	CC
PV_Br_SNP_0666	GG	GG	GG	GG	GG	GG	TG	NN	GG	GG	GG	TG	GG	GG	GG	GG	GG
PV_Br_SNP_1046	CC	CT	CC	CC	CC	CC	CC	CT	CT	CC	CT	CT	CC	CC	NN	CC	CC
PV_Br_SNP_0678	NN	CC	NN	CT	CC	NN	NN	NN	CT	NN	CT	NN	TT	CT	NN	TT	CT
PV_Br_SNP_0687	AA	AA	AA	AA	AA	AA	NN	NN	AA	AA	AA	AA	AA	CA	AA	CC	CA
PV_Br_SNP_0688	AA	AA	AA	AA	AA	AA	AA	NN	AA	AA	AA	GG	AA	AA	AA	AA	AA
PV_Br_SNP_0691	TT	TT	TA	AA	TT	AA	TT	TT	TT	AA	TA	TA	AA	TT	NN	AA	AA
PV_Br_SNP_0696	CC	NN	TC	TT	TT	CC	TC	TT	TT	TT	TC	TT	TT	NN	NN	CC	CC
PV_Br_SNP_0697	NN	NN	TT	NN	NN	NN	NN	NN	NN	NN	CC	NN	NN	NN	NN	CC	NN
PV_Br_SNP_0698	CC	TC	CC	CC	CC	CC	CC	NN	TC	CC	TC	TC	TC	TC	CC	TT	CC
PV_Br_SNP_0699	GG	GG	GG	GG	GG	GG	AG	NN	GG	GG	GG	GG	GG	GG	GG	GG	GG
PV_Br_SNP_0709	TT	TT	TT	TT	AA	TT	TT	TT	TT	TT	TT	TT	TT	NN	NN	TT	TT
PV_Br_SNP_0710	CC	CC	CC	CC	CC	CC	CC	NN	CC	CC	CC	CC	CC	CC	CC	CC	CC
PV_Br_SNP_1066	AA	AA	NN	AA	AA	AA	AA	AA	AA	AA	AA	AA	AA	NN	NN	AA	AA
PV_Br_SNP_0721	AA	AA	AA	AA	AA	AA	AA	AA	AA	AA	CA	AA	AA	AA	NN	CA	AA
PV_Br_SNP_0722	TT	TT	TC	TT	TT	TT	TT	NN	TT	TT	TC	TT	TC	CC	TT	TC	TT
PV_Br_SNP_1061	AG	GG	GG	GG	GG	GG	GG	AG	GG	AG	GG	GG	AG	GG	NN	GG	GG
PV_Br_SNP_0725	GG	GG	GG	GG	GG	GG	GG	NN	GG	GG	AA	GG	AG	GG	AG	AG	GG
PV_Br_SNP_0729	NN	AA	AA	AA	AA	AA	AA	NN	AA	AA	AA	AA	AA	AA	AA	AA	AA
PV_Br_SNP_0743	GG	GG	GG	GG	AA	AA	GG	GG	NN	GG	GG	GG	GG	GG	NN	GG	GG
PV_Br_SNP_0744	GG	GG	GG	GG	GG	GG	GG	NN	GG	GG	GG	GG	GG	GG	GG	GG	GG
PV_Br_SNP_0745	AA	AA	GG	GG	GA	AA	GA	NN	GG	AA	AA	AA	AA	AA	GG	GG	AA
PV_Br_SNP_0746	AA	TA	AA	AA	AA	AA	AA	NN	AA	AA	TA	AA	TA	AA	AA	AA	AA
PV_Br_SNP_0754	AA	AA	AT	TT	AA	AT	AT	TT	TT	AT	AA	TT	TT	AT	NN	AA	AA
PV_Br_SNP_0760	TT	TT	TT	TT	TT	TT	TT	NN	TT	TT	TT	TT	TT	TT	TT	TT	TT
PV_Br_SNP_0761	CT	CT	CT	CT	CC	CC	TT	NN	CT	TT	CT	CT	CT	NN	TT	CC	CT
PV_Br_SNP_0762	GG	GG	GG	NN	GG	GG	GG	NN	GG	GG	AA	AA	AA	GG	GG	AG	GG
PV_Br_SNP_1062	AA	AA	CC	AA	CC	AA	AC	AC	AA	AA	AA	CC	AA	NN	NN	AA	CC
PV_Br_SNP_1072	CC	CC	CC	CC	CC	CC	CC	CC	CC	CC	CC	CC	NN	NN	NN	CC	CC
PV_Br_SNP_0776	GG	GG	GG	GG	GG	GG	GG	GG	GG	GG	GG	GG	TG	NN	NN	GG	GG
PV_Br_SNP_0778	TT	TT	GG	GG	TT	GG	TT	NN	TT	GG	GG	GG	GG	TT	TT	NN	GG
PV_Br_SNP_0779	AA	AA	AA	AA	AA	AA	AA	NN	AA	AA	AA	AA	AA	AA	AA	AA	AA
PV_Br_SNP_0783	AA	TT	AT	AA	AT	AA	AT	NN	TT	AA	AT	TT	TT	AT	TT	TT	AA
PV_Br_SNP_1041	AA	AA	NN	AA	CC	AA	AA	AA	AC	AA	AA	AA	AA	AA	NN	AA	AA
PV_Br_SNP_1037	GG	AG	GG	GG	GG	GG	GG	GG	GG	GG	GG	AG	GG	NN	NN	GG	GG
PV_Br_SNP_0790	CC	CC	CC	CC	AC	AC	CC	AA	AA	CC	CC	AC	AA	CC	NN	AC	CC
PV_Br_SNP_0792	CC	CC	CC	CC	CC	CC	CC	NN	CC	CC	CC	CC	CC	CC	CC	CC	CC
PV_Br_SNP_0795	AA	AA	AA	AA	AA	AA	AA	AA	AA	AA	AA	AA	AA	AA	NN	AA	AA
PV_Br_SNP_0796	GG	GG	GG	GG	GG	GG	GG	NN	GG	GG	AG	AA	AA	GG	GG	AG	GG
PV_Br_SNP_0798	CC	CC	TT	CC	CC	TC	CC	NN	TC	CC	TT	CC	TC	TC	CC	TC	CC
PV_Br_SNP_0800	CC	CC	CC	CC	CC	CC	CC	NN	CC	CC	CC	CC	CC	CC	CC	CC	CC
PV_Br_SNP_1083	CC	CC	CC	CC	AC	CC	CC	CC	CC	CC	CC	CC	AC	AC	NN	AC	CC
PV_Br_SNP_0809	GG	GA	GA	GA	GA	GG	GG	AA	GA	AA	GG	GG	GA	NN	NN	GA	GG
PV_Br_SNP_0810	TT	TT	TT	TT	GT	TT	TT	NN	TT	TT	GG	TT	TT	TT	TT	GT	GG
PV_Br_SNP_0811	TT	TT	TT	TT	TT	TT	TT	NN	TT	TT	TT	TT	TT	TT	TT	TT	TT
PV_Br_SNP_1071	CC	CC	CC	CC	CC	CC	CC	CC	CC	CC	TT	TT	CT	NN	NN	CT	CT
PV_Br_SNP_0812	GG	AA	NN	NN	NN	GG	NN	NN	NN	AA	AA	GG	AG	NN	GG	GG	NN
PV_Br_SNP_1049	AA	AG	AG	AG	AA	AG	AG	AG	AG	AG	AA	AA	AA	AG	NN	AA	AG
PV_Br_SNP_1044	AA	NN	NN	NN	AA	AT	AA	NN	AT	TT	AA	AA	AT	AA	AA	NN	AA
PV_Br_SNP_0826	CC	CC	CC	CC	CC	CC	CC	CC	CC	CC	AC	CC	CC	NN	NN	CC	CC
PV_Br_SNP_0827	GG	CG	CC	GG	GG	CG	CC	NN	GG	CC	GG	CG	GG	CC	CG	GG	GG
PV_Br_SNP_0828	TC	TC	TC	TC	TC	TC	TC	NN	TC	TC	TC	TC	TC	TC	TC	TC	TC
PV_Br_SNP_0839	NN	NN	NN	NN	TT	NN	NN	NN	TT	NN	TT	TT	TT	TT	NN	TT	NN
PV_Br_SNP_0848	CC	TT	CC	CC	TC	CC	CC	CC	CC	CC	CC	CC	TC	CC	NN	CC	TC
PV_Br_SNP_0850	TT	TT	TT	TT	TT	TT	TT	NN	TT	TT	TT	TT	TT	TT	TT	TT	TT
PV_Br_SNP_0851	TT	TT	TT	TT	TT	TT	TT	NN	TT	TT	TT	TT	TT	TT	TT	TT	TT
PV_Br_SNP_0855	CC	TC	CC	CC	CC	TC	CC	NN	TC	CC	TC	TC	TC	CC	TC	NN	CC
PV_Br_SNP_0865	AG	GG	GG	AA	AG	GG	AG	GG	GG	GG	AG	GG	AG	GG	NN	AA	AG
PV_Br_SNP_0867	CC	CC	CC	CC	CC	CC	CC	NN	CC	CC	GG	CC	CC	CC	CC	CC	CC
PV_Br_SNP_0868	GG	GG	GG	GG	GG	GG	GG	NN	GG	GG	GG	GG	GG	GG	GG	AG	GG
PV_Br_SNP_0869	CC	CC	CC	CC	CC	CC	CC	NN	CC	CC	CC	CC	CC	CC	CC	CC	CC
PV_Br_SNP_0879	GA	GA	GA	GA	GG	GG	GG	GA	GA	GG	GA	GA	GG	NN	NN	AA	GA
PV_Br_SNP_0882	CC	TC	CC	TT	TT	CC	TC	NN	TC	TT	TC	CC	CC	CC	TC	NN	TT
PV_Br_SNP_0883	GG	GG	GG	GG	GG	GG	GG	NN	GG	GG	GG	GG	GG	GG	GG	NN	GG
PV_Br_SNP_0889	AT	AA	TT	AT	AT	TT	TT	NN	AT	TT	TT	TT	AT	AA	AT	NN	TT
PV_Br_SNP_0902	TT	TT	TT	TA	AA	TA	TA	TT	TT	TT	TT	AA	AA	NN	NN	TT	TT
PV_Br_SNP_0904	GG	GG	GG	GG	GG	GG	GG	NN	GG	GG	AA	GG	GG	GG	GG	NN	GG
PV_Br_SNP_0906	GG	GG	GG	GG	GG	GG	AG	NN	GG	GG	AG	GG	GG	GG	AA	GG	GG
PV_Br_SNP_0907	GG	GG	GG	GG	GG	GG	GG	NN	GG	GG	GG	GG	GG	GG	GG	GG	GG
PV_Br_SNP_1038	AC	AA	AA	AA	AA	AA	AC	AC	AA	AA	AA	CC	AC	AA	NN	CC	AA
PV_Br_SNP_0918	GG	GG	GG	GG	GG	GG	GG	GG	GG	GG	GG	GG	GG	GG	NN	GG	GG
PV_Br_SNP_0919	TT	TT	TT	TT	TT	TT	TT	NN	TT	TT	TT	TT	TT	TT	TT	CT	TT
PV_Br_SNP_0920	GG	GG	GG	GG	GG	GG	GG	NN	GG	GG	GG	GG	GG	GG	GG	GG	GG
PV_Br_SNP_0921	CC	CC	CC	CC	CC	CC	CC	CC	CC	CC	CC	TT	TT	CC	NN	CC	TC
PV_Br_SNP_0924	CA	CC	CC	CC	CC	CC	CC	NN	CC	CC	CC	AA	CA	CC	CC	CC	CC
PV_Br_SNP_1067	GG	GG	GG	GG	GG	GG	GG	GG	GG	GG	GG	GG	CG	CC	NN	GG	GG
PV_Br_SNP_1068	GG	GG	GG	GG	GG	GG	GG	GG	GG	GG	GG	GG	AG	NN	NN	GG	GG
PV_Br_SNP_0931	GG	GG	GG	GG	GG	GG	GG	NN	GG	GG	TG	GG	GG	GG	GG	GG	GG
PV_Br_SNP_0939	AG	AG	AA	AG	AG	AA	AG	NN	AG	AA	AG	AG	GG	AA	GG	GG	AG
PV_Br_SNP_0951	GG	GG	GG	GG	GG	GG	GG	GG	GG	GG	AA	GG	GG	GG	NN	AA	GG
PV_Br_SNP_0952	CC	AA	AA	AC	AA	AC	AA	NN	AA	CC	CC	AA	AC	AA	AA	AC	CC
PV_Br_SNP_0954	AA	GG	AA	AA	AG	GG	AG	NN	GG	GG	GG	GG	GG	AA	GG	AG	AA
PV_Br_SNP_0955	GG	TG	GG	TT	GG	GG	GG	NN	GG	GG	TG	TG	TG	GG	TT	TT	GG
PV_Br_SNP_0958	TT	TT	TT	TT	TT	TT	TT	TT	TT	TT	TT	TT	TT	TT	NN	TT	TT
PV_Br_SNP_0959	CC	CC	CC	CC	CC	CC	CC	NN	CC	CC	TC	CC	CC	CC	CC	TC	CC
PV_Br_SNP_0960	AA	NN	AA	NN	AA	GA	NN	NN	AA	AA	GA	AA	AA	NN	GA	GA	AA
PV_Br_SNP_0966	TT	CC	TT	TT	TT	TT	TT	NN	CC	TT	TC	CC	TT	TT	NN	CC	TT
PV_Br_SNP_0968	CC	TT	CC	TT	TT	TT	TT	NN	TT	TT	TT	TT	TT	TT	TT	TT	TT
PV_Br_SNP_0969	TT	TT	NN	NN	TT	GG	TT	NN	GT	TT	TT	TT	TT	TT	GG	GG	TT
PV_Br_SNP_0970	CC	TC	TT	TC	TT	TT	TC	NN	TC	TT	CC	CC	TC	TC	TC	CC	TC
PV_Br_SNP_0974	CC	CC	CC	CC	CC	CC	CC	CC	CC	CC	CC	CC	CC	CC	NN	CC	CC
PV_Br_SNP_0975	TT	TT	CT	TT	TT	CT	TT	NN	TT	TT	TT	TT	CT	TT	TT	CT	TT
PV_Br_SNP_0976	GG	GG	GG	GG	GG	GG	GG	NN	GG	GG	GG	GG	GG	NN	GG	GG	GG
PV_Br_SNP_0980	AA	AA	AA	AA	AA	AA	AA	NN	AA	AA	TA	AA	AA	AA	AA	AA	AA
PV_Br_SNP_0985	TT	TT	TT	TT	TT	TT	TT	TT	TT	TT	TT	TT	TT	NN	NN	TT	TT
PV_Br_SNP_0986	GG	GG	GG	GG	GG	GG	GG	NN	GG	GG	NN	GG	GG	GG	GG	CC	GG
PV_Br_SNP_0987	AA	AA	AA	AA	AA	AA	AA	NN	AA	AA	GA	GA	AA	AA	AA	GA	AA
PV_Br_SNP_0989	TT	TT	TT	TT	TT	TT	TT	NN	TT	TT	TT	TT	CC	TT	TT	TT	TT
PV_Br_SNP_0996	GA	GG	AA	AA	AA	AA	AA	AA	AA	AA	AA	AA	AA	NN	NN	AA	AA
PV_Br_SNP_0998	TT	TT	TT	TT	TT	TT	TT	NN	TT	TT	TT	GT	GT	TT	TT	GT	TT
PV_Br_SNP_0999	TT	TT	TT	TT	TT	TT	TT	NN	TT	TT	TT	TT	TT	TT	TT	TT	TT
PV_Br_SNP_1053	GG	GG	AG	AA	AA	AG	GG	AG	GG	AG	AA	AA	AA	NN	NN	AA	AA
PV_Br_SNP_1054	GG	GG	CG	CC	CC	CG	CG	CG	GG	CG	CC	CC	CC	NN	NN	CC	CG
