# Supplementary material for: Morphology, Carbohydrate Composition and Vernalization Response in a Genetically Diverse Collection of Asian and European Turnips (Brassica rapa subsp. rapa)
Source: PLoS One. 2014 Dec 4;9(12):e114241. doi: 10.1371/journal.pone.0114241 (PMC4256417; doi:10.1371/journal.pone.0114241)
Supplement: Table S5 — Descriptive of phenotypic value of traits that were evaluated in each experiment. The presented traits are leaf color (LC), leaf length (LL), lamina blade length (LBL), lamina blade width (LBW), leaf index (LI), petiole length (PL), petiole width (PW), leaf lobe (LB), leaf lobelets (LBs), leaf edge shape (LES), leaf blade shape outline (LS), leaf division (LD), leaf apex shape (LAS), leaf hairiness (LH), leaf lamina attitude (LAT), leaf and stem weight (Lwe), flowering time (FT), tuber length (TL), tuber width (Twi), tuber index (TI), tuber shape (TS), tuber color (TC), tuber shoots number (Tsh), tuber weight (Twe), tuber dry weight (TDW), tuber surface smoothness (Tss), tuber swelling onset (Tso), tuber growing depth (Tgd) and tuber dry mass% (TDM). Experiment code “2008F, 2009F, 2011F and 2012F” stand for four field experiments carried out between 2008 and 2012. Code “2010G” means the greenhouse experiment in 2010. (PDF) [file pone.0114241.s012.pdf]

Table S5 Descriptive of phenotypic value of traits that were evaluated in each experiment.

The presented traits are leaf color (LC), leaf length (LL), lamina blade length (LBL), lamina blade width (LBW), leaf index (LI), petiole length (PL), petiole width (PW),leaf lobe (LB), leaf lobelets (LBs), leaf edge shape (LES), leaf blade shape outline (LS), leaf division (LD), leaf apex shape (LAS), leaf hairiness (LH), leaf lamina attitude (LAT), leaf and stem weight (Lwe), flowering time (FT), tuber length (TL), tuber width (Twi), tuber index (TI), tuber shape (TS), tuber color (TC), tuber shoots number (Tsh), tuber weight (Twe), tuber dry weight (TDW), tuber surface smoothness (Tss), tuber swelling onset (Tso), tuber growing depth (Tgd) and tuber dry mass% (TDM). Experiment code "2008F, 2009F,2011F and 2012F" stand for four field experiments carried out between 2008 and 2012. Code "2010G" means the greenhouse experiment in 2010.

Experiment 2008F																
Accession		Twi (mm)	TL (mm)	TI	FT (day)	LL (cm)	LBW (cm)	LLL (cm)	LI	PW (cm)	LPL (cm)	LS	LDM	LDI	LB	LAS
FT_001	Mean	132.0	163.0	.8	150.0	45.3	20.6	39.2	1.9	1.4	6.0	2.3	2.3	3.0	5.3	5.3
	Std. Deviation	16.9	14.1	.1	.0	1.9	3.0	2.3	.3	.0	2.0	.6	.6	.0	1.2	1.2
FT_002	Mean	133.6	143.8	.9	150.0	46.1	22.6	39.3	1.8	1.3	4.1	3.3	3.3	3.0	6.0	4.7
	Std. Deviation	32.8	20.2	.1	.0	1.7	2.8	1.2	.3	.2	.5	1.5	2.3	.0	1.0	1.2
FT_003	Mean	115.5	201.0	.6	150.0	45.4	21.4	42.7	2.1	1.5	2.0	4.7	2.7	3.0	7.3	4.0
	Std. Deviation	5.6	21.7	.0	.0	.7	4.3	1.6	.4	.3	1.2	2.5	1.2	.0	1.2	.0
FT_004	Mean	95.5	147.5	.7	150.0	44.8	17.6	41.1	2.4	1.3	3.0	3.7	2.3	3.0	8.0	4.7
	Std. Deviation	20.6	7.9	.1	.0	2.0	3.5	.8	.5	.5	2.0	.6	.6	.0	4.0	1.2
FT_005	Mean	96.4	202.8	.5	150.0	44.3	21.6	41.6	1.9	1.4	2.1	2.7	2.0	3.0	8.0	5.3
	Std. Deviation	24.0	61.8	.2	.0	1.1	2.5	1.3	.3	.2	.6	1.2	.0	.0	.0	1.2
FT_047	Mean	162.7	91.8	1.8	150.0	44.4	20.6	38.8	1.9	1.4	3.7	3.7	4.0	3.0	7.0	4.0
	Std. Deviation	17.2	11.2	.3	.0	3.4	1.5	3.2	.3	.1	1.3	2.1	1.7	.0	1.7	.0
FT_051	Mean	109.1	51.9	2.1	150.0	43.8	17.2	35.4	2.1	1.0	7.4	4.3	2.0	3.0	3.3	4.0
	Std. Deviation	12.9	4.8	.5	.0	3.7	3.1	2.4	.4	.1	5.3	2.5	.0	.0	.6	.0
FT_088	Mean	109.3	142.3	.9	150.0	45.4	19.0	41.0	2.2	1.1	2.4	3.7	4.3	2.0	.0	5.3
	Std. Deviation	5.8	61.7	.4	.0	4.7	.9	2.8	.0	.2	.5	.6	1.5	.0	.0	1.2
FT_097	Mean	99.1	113.6	.9	150.0	45.9	25.0	41.5	1.7	1.6	3.2	2.7	4.3	1.7	.0	6.0
	Std. Deviation	21.4	4.1	.2	.0	4.5	4.9	5.0	.4	.7	1.8	1.5	2.1	.6	.0	.0
T_1050V	Mean	144.9	121.4	1.2	150.0	44.3	17.0	39.7	2.5	1.3	4.4	3.3	2.3	3.0	6.7	4.7
	Std. Deviation	44.3	21.0	.3	.0	3.0	5.1	2.4	.6	.6	1.4	1.2	.6	.0	2.3	1.2
T_1283V	Mean	147.5	116.4	1.3	150.0	47.7	17.7	40.6	2.3	1.0	5.6	3.3	2.7	3.0	5.0	5.3
	Std. Deviation	23.5	4.5	.2	.0	1.3	2.8	3.8	.3	.1	3.0	1.2	.6	.0	2.6	1.2
T_163V	Mean	-	-	-	150.0	46.5	18.8	26.9	1.4	1.0	19.1	1.0	1.0	1.0	.0	6.7
	Std. Deviation	-	-	-	.0	1.5	1.1	2.4	.2	.3	.9	.0	.0	.0	.0	1.2
T_307V	Mean	83.4	149.0	.6	150.0	43.1	21.2	39.4	1.9	1.3	3.2	3.0	2.7	3.0	7.0	5.3
	Std. Deviation	8.9	14.6	.1	.0	3.7	4.2	2.1	.3	.6	2.2	1.7	.6	.0	1.7	1.2
T_385V	Mean	133.0	170.9	.8	150.0	45.9	20.7	40.1	2.0	1.1	6.0	2.0	3.0	3.0	7.0	4.7
	Std. Deviation	14.3	56.0	.2	.0	1.6	3.5	.6	.3	.2	2.6	.0	.0	.0	1.0	1.2
T_738V	Mean	154.8	128.4	1.2	150.0	47.2	19.9	42.2	2.1	1.6	3.9	4.0	2.7	2.7	5.0	5.3
	Std. Deviation	51.5	9.1	.3	.0	1.3	.7	3.0	.2	.4	1.7	.0	.6	.6	2.0	1.2
T_821V	Mean	122.4	66.5	1.9	150.0	-	-	-	-	-	-	-	-	-	-	-
	Std. Deviation	6.2	11.3	.4	.0	-	-	-	-	-	-	-	-	-	-	-
T_826V	Mean	153.9	102.9	1.5	150.0	41.7	18.2	38.0	2.1	1.3	2.9	3.0	3.0	2.0	1.0	6.0
	Std. Deviation	38.2	28.0	.1	.0	3.8	2.5	4.4	.4	.3	2.0	1.0	1.7	.0	1.7	.0
T_830V	Mean	158.4	100.6	1.7	150.0	20.4	10.8	18.0	1.1	.5	1.3	2.3	1.7	1.7	3.0	2.7
	Std. Deviation	20.0	25.0	.5	.0	20.2	10.9	19.1	.9	.5	1.6	2.5	1.5	1.5	3.0	2.3
TG_129	Mean	130.6	87.0	1.5	150.0	42.8	20.6	36.8	1.8	1.1	4.8	2.3	2.3	3.0	6.0	5.3
	Std. Deviation	44.0	10.8	.3	.0	2.9	1.9	2.5	.1	.3	2.5	.6	.6	.0	2.0	1.2
VT_006	Mean	121.1	102.4	1.2	150.0	47.9	22.2	42.4	1.9	1.5	5.3	3.3	3.0	2.7	3.0	5.3
	Std. Deviation	19.6	10.2	.3	.0	1.8	2.9	1.9	.3	.2	1.1	1.5	1.7	.6	1.0	1.2
VT_007	Mean	90.0	50.4	1.8	150.0	45.4	17.7	40.1	2.3	1.0	4.4	2.0	4.3	3.0	4.7	5.3
	Std. Deviation	11.9	8.3	.4	.0	2.2	4.3	3.8	.3	.1	2.5	.0	2.1	.0	1.2	1.2
VT_008	Mean	148.3	123.8	1.2	150.0	46.8	20.6	38.8	1.9	1.4	3.9	3.0	1.7	3.0	4.0	5.3
	Std. Deviation	27.1	5.5	.2	.0	2.4	1.7	2.0	.1	.2	.9	1.0	.6	.0	1.0	1.2
VT_009	Mean	111.3	85.2	1.3	150.0	44.5	15.1	35.3	2.5	1.2	8.7	3.3	1.3	2.3	2.7	5.3
	Std. Deviation	19.0	10.2	.2	.0	2.1	4.9	3.0	.7	.2	1.7	1.2	.6	1.2	3.1	1.2
VT_010	Mean	144.2	89.7	1.6	150.0	44.4	17.7	41.6	2.6	1.4	2.5	2.0	3.3	3.0	6.3	5.3
	Std. Deviation	32.2	5.1	.3	.0	3.1	5.4	2.8	1.0	.4	.6	.0	2.3	.0	1.5	1.2
VT_011	Mean	125.8	91.0	1.4	150.0	39.6	15.8	36.0	2.3	1.0	2.5	3.0	2.7	3.0	5.3	5.3
	Std. Deviation	9.1	20.4	.4	.0	3.3	1.5	1.7	.2	.2	.4	1.7	.6	.0	3.1	1.2
VT_012	Mean	-	-	-	79.0	47.5	15.0	37.7	2.5	1.2	5.5	4.3	3.0	2.3	.0	4.0
	Std. Deviation	-	-	-	8.0	.7	.7	4.1	.3	.4	2.5	2.5	.0	.6	.0	.0
VT_013	Mean	129.7	97.2	1.3	150.0	32.3	11.8	28.4	1.6	.8	3.6	2.7	2.3	1.0	.0	3.3
	Std. Deviation	20.8	8.2	.1	.0	28.0	10.3	24.6	1.4	.7	3.2	3.1	2.5	1.0	.0	3.1
VT_014	Mean	136.5	70.6	1.9	150.0	39.2	17.4	36.9	2.2	.9	3.0	1.7	1.7	1.3	.0	6.0
	Std. Deviation	5.0	8.0	.2	.0	2.5	4.1	1.9	.5	.1	2.1	.6	.6	.6	.0	2.0
VT_015	Mean	119.7	58.3	2.1	150.0	45.7	19.1	41.6	2.2	1.0	4.3	2.3	2.3	2.3	4.0	5.3
	Std. Deviation	16.5	10.0	.4	.0	3.6	2.4	4.9	.4	.2	3.5	.6	.6	1.2	3.5	1.2
VT_017	Mean	141.9	72.9	1.9	62.0	42.9	16.0	37.1	2.4	1.0	5.6	3.0	2.3	3.0	4.7	5.3
	Std. Deviation	23.9	9.4	.1	.0	4.8	3.2	7.6	.8	.3	3.2	1.7	.6	.0	2.1	1.2
VT_018	Mean	135.3	99.5	1.4	150.0	46.0	20.2	42.1	2.1	1.0	3.2	4.3	3.3	2.7	3.7	4.0
	Std. Deviation	8.3	4.8	.1	.0	.3	3.4	.6	.4	.1	.5	2.5	2.3	.6	3.2	.0
VT_044	Mean	-	-	-	150.0	11.5	4.8	9.6	.7	.3	1.1	.7	.7	1.0	1.3	1.3
	Std. Deviation	-	-	-	.0	20.0	8.3	16.7	1.2	.5	1.8	1.2	1.2	1.7	2.3	2.3
VT_045	Mean	137.9	69.9	2.0	150.0	43.5	19.2	40.3	2.1	1.0	2.4	3.0	2.0	2.3	2.7	6.0
	Std. Deviation	18.1	1.7	.2	.0	3.0	3.3	4.5	.4	.6	1.5	1.0	.0	.6	4.6	.0
VT_052	Mean	117.9	189.8	.6	150.0	43.3	20.0	33.3	1.7	.8	10.6	3.0	2.3	3.0	3.3	5.3
	Std. Deviation	29.3	57.2	.0	.0	4.7	2.4	8.4	.7	.1	3.9	1.7	.6	.0	1.5	1.2
VT_053	Mean	-	-	-	123.0	38.3	15.5	34.2	2.2	.6	4.1	3.7	2.7	3.0	5.7	4.7
	Std. Deviation	-	-	-	54.0	2.8	2.0	3.9	.4	.2	1.6	2.9	.6	.0	2.5	1.2
VT_089	Mean	141.0	88.7	1.6	150.0	47.9	18.3	43.3	2.4	1.3	4.5	2.7	2.3	3.0	6.7	6.0
	Std. Deviation	15.5	16.7	.4	.0	.8	2.5	1.5	.4	.3	2.2	1.2	.6	.0	1.2	.0
VT_090	Mean	128.5	175.5	.9	150.0	43.4	19.1	38.6	2.0	1.1	4.6	3.7	2.7	3.0	3.7	3.3
	Std. Deviation	34.3	61.0	.5	.0	3.2	1.8	3.0	.1	.1	1.2	2.9	.6	.0	2.5	1.2
VT_091	Mean	154.3	129.9	1.2	113.0	48.7	19.6	40.0	2.0	1.2	6.7	2.3	2.7	2.7	4.7	4.0
	Std. Deviation	33.2	8.4	.3	52.0	1.4	1.8	3.8	.2	.2	.9	.6	.6	.6	2.5	.0
VT_092	Mean	151.0	76.3	2.0	150.0	47.4	16.8	45.5	2.8	.9	1.3	4.0	3.7	1.7	.0	4.7
	Std. Deviation	16.6	4.0	.2	.0	2.8	2.1	2.4	.5	.2	.6	2.6	1.5	.6	.0	1.2
VT_115	Mean	74.6	156.1	.5	134.0	47.5	14.3	32.5	2.3	1.1	14.6	5.0	3.3	2.0	2.0	4.7
	Std. Deviation	14.7	25.0	.1	32.0	2.0	1.6	3.5	.5	.3	5.2	2.6	2.3	1.0	3.5	1.2
VT_116	Mean	-	-	-	63.0	47.6	17.2	34.9	2.0	1.1	13.3	2.0	2.7	3.0	3.0	5.3
	Std. Deviation	-	-	-	5.2	.7	.8	6.1	.3	.1	4.7	.0	.6	.0	1.0	1.2
VT_117	Mean	126.7	67.3	1.9	58.0	47.1	16.5	35.5	2.2	1.0	11.8	3.0	2.0	2.7	2.3	4.0
	Std. Deviation	10.2	8.2	.1	3.0	2.2	2.5	5.2	.4	.1	3.9	1.7	.0	.6	.6	.0
VT_119	Mean	98.3	220.4	.4	150.0	43.1	19.0	40.2	2.1	1.1	3.3	2.0	2.3	3.3	6.0	4.0
	Std. Deviation	17.6	9.8	.1	.0	5.2	2.8	4.5	.1	.2	1.5	.0	.6	.6	1.0	.0
VT_120	Mean	123.2	84.0	1.5	150.0	44.6	24.8	42.5	1.7	1.2	2.0	2.3	2.3	3.3	5.3	4.7
	Std. Deviation	27.9	2.8	.3	.0	2.5	2.4	.8	.2	.2	.9	.6	.6	.6	1.2	1.2
VT_123	Mean	125.7	137.1	.9	150.0	46.4	16.9	40.8	2.4	1.3	4.5	3.7	2.7	2.0	.0	6.0
	Std. Deviation	4.1	16.9	.1	.0	2.1	1.8	3.7	.4	.4	1.9	.6	2.1	.0	.0	.0
VT_137	Mean	141.7	76.9	1.9	150.0	45.8	20.2	40.1	2.1	1.0	5.5	2.7	2.0	3.0	4.0	5.3
	Std. Deviation	16.9	13.9	.5	.0	.7	5.0	1.8	.4	.2	3.0	.6	.0	.0	.0	1.2

Experiment 2009F

Accession		FT (day)	LC	Twe (mm)	TL (mm)	Twi (mm)	Tsh	Tl
FT_001	Mean	150	37	1319	214	119	9	1
	Std. Deviation	0	3	114	1	4	2	0
FT_002	Mean	150	41	1926	148	212	8	1
	Std. Deviation	0	4	60	21	6	1	0
FT_003	Mean	150	42	1506	205	111	9	1
	Std. Deviation	0	3	231	16	12	1	0
FT_004	Mean	150	40	2041	187	151	8	1
	Std. Deviation	0	2	551	4	13	3	0
FT_005	Mean	150	41	1723	198	101	10	1
	Std. Deviation	0	4	363	24	1	2	0
FT_047	Mean	150	40	1354	124	147	4	1
	Std. Deviation	0	4	93	1	6	1	0
FT_051	Mean	150	43	894	80	161	5	2
	Std. Deviation	0	3	83	11	6	2	0
FT_086	Mean	150	42		114	114	12	1
	Std. Deviation	0	5		.	.	.	.
FT_088	Mean	150	38	1805	205	147	11	1
	Std. Deviation	0	3	356	8	29	3	0
FT_097	Mean	150	43	553	112	96	12	1
	Std. Deviation	0	4	166	6	4	2	0
T_1050V	Mean	150	42	2029	165	172	10	1
	Std. Deviation	0	7	596	12	38	1	0
T_1283V	Mean	150	38	886	102	156	11	2
	Std. Deviation	0	2	361	3	31	4	0
T_307V	Mean	150	43	2024	210	152	9	1
	Std. Deviation	0	3	957	8	2	1	0
T_385V	Mean	150	41	1191	151	127	9	1
	Std. Deviation	0	4	689	26	42	3	0
T_738V	Mean	150	51	957	141	115	6	1
	Std. Deviation	0	5	510	27	27	2	0
T_830V	Mean	150	45	948	95	144	8	2
	Std. Deviation	0	5	59	7	0	1	0
T_pancai	Mean	81	42	680	84	147	5	2
	Std. Deviation	2	3	136	13	21	2	0
T_RR	Mean	94	41					
	Std. Deviation	0	5					
VT_007	Mean	150	40	1128	105	156	10	1
	Std. Deviation	0	4	202	1	6	5	0
VT_008	Mean	95	39	1666	95	178	12	2
	Std. Deviation	.	4	.	.	.	3	.
VT_009	Mean	98	46	1106	111	143	19	1
	Std. Deviation	1	3	118	8	16	2	0
VT_010	Mean	150	41	1868	111	208	8	2
	Std. Deviation	0	3	240	1	18	0	0
VT_011	Mean	150	40	2450	155	184	14	1
	Std. Deviation	0	4	163	18	25	0	0
VT_012	Mean	95	43	1227	132	148	11	1
	Std. Deviation	0	4	303	30	25	4	0
VT_013	Mean	95	40	449	101	95	6	1
	Std. Deviation	1	3	137	4	5	1	0
VT_014	Mean	150	38	1475	99	192	4	2
	Std. Deviation	0	3	763	26	43	2	0
VT_015	Mean	150	39	1493	106	196	8	2
	Std. Deviation	0	3	.	.	.	.	.
VT_017	Mean	150	40	1033	100	151	2	2
	Std. Deviation	0	3	241	4	37	1	0
VT_018	Mean	150	42	1432	150	174	13	1
	Std. Deviation	0	2	478	11	18	2	0
VT_044	Mean	150	38	2773	137	182	8	1
	Std. Deviation	0	2	1814	44	26	1	0
VT_045	Mean	150	41	2357	107	239	9	2
	Std. Deviation	0	4	17	17	1	2	0
VT_052	Mean	150	44	1700	273	119	9	0
	Std. Deviation	0	3	441	19	6	2	0
VT_053	Mean	150	39	896	93	159	8	2
	Std. Deviation	0	2	162	6	1	3	0
VT_089	Mean	150	40	1231	125	159	9	1
	Std. Deviation	0	2	160	26	19	4	0
VT_090	Mean	150	44	561	197	78	11	0
	Std. Deviation	0	3	.	.	.	.	.
VT_091	Mean	150	42	1750	152	169	6	1
	Std. Deviation	0	2	687	16	29	1	0
VT_092	Mean	150	49	1199	96	163	9	2
	Std. Deviation	0	10	23	4	10	4	0
VT_116	Mean	56	42		77	107	12	1
	Std. Deviation	0	4		14	10	2	0
VT_117	Mean	48	42		85	105	15	1
	Std. Deviation	2	5		.	.	1	.
VT_119	Mean	150	41	2329	295	128	7	0
	Std. Deviation	0	7	563	64	25	1	0
VT_120	Mean	150	43	1020	126	152	6	1
	Std. Deviation	0	4	227	6	22	5	0
VT_123	Mean	93	46	685	136	96	23	1
	Std. Deviation	2	2	158	21	25	2	0
VT_137	Mean	150	42	1399	107	167	9	2
	Std. Deviation	0	3	342	3	8	2	0

Experiment 2010G

Accession		Twe (gram)	Twi (mm)	Tsh	Tgd	Tss	Tso	FT (day)	LBL (mm)	LBW (mm)	LI	Lwe (gram)	LC	PW (mm)	LES	LAT
FT_002	Mean	442	110	2	2	2	27	150	214	162	1	158	33	3	3	2
	Std. Deviation	118	12	1	1	1	4		19	15		61	2	2	1	1
FT_004	Mean	477	76	5	2	2	33	150	197	135	1	492	31	6	4	2
	Std. Deviation	117	10	1		1	7		20	7		184	1	2	1	1
FT_047	Mean	265	89	2	3	2	34	150	201	146	1	149	29	4	3	2
	Std. Deviation	79	8			1	4		24	10		22	5	1	1	1
FT_051	Mean	232	91	1	2	2	32	150	189	120	2	122	32	6	2	3
	Std. Deviation	141	19	1	1	1	6		22	10		38	4	1	1	1
FT_056	Mean	19	20	5	1	2	47	150	166	150	1	460	27	2	2	3
	Std. Deviation	9	1	1		1	10		25	16		43	4	2	1	1
FT_086	Mean	320	98	2	3	3	29	150	180	120	2	128	36	5	3	3
	Std. Deviation	139	14			1	3		15	22		63	1	1	1	2
FT_097	Mean	126	50	4	2	3	37	150	203	155	1	1059	34	3	3	2
	Std. Deviation	80	13	1		1	5		10	29		340	2	1	1	1
SM_14	Mean	238	85	3	3	1	27	150	265	110	2	155	33	1	2	3
	Std. Deviation	71	12			1	2		9	3		26	1	1	2	1
SM_15	Mean	411	105	2	3	2	31	150	242	139	2	211	26	4	3	2
	Std. Deviation	332	32	1		1	3		23	8		83	1		1	
SM_16	Mean	233	72	4	3	2	25	120	250	133	2	191	30	5	2	2
	Std. Deviation	180	19	1	1		1	35	24	15		97	3	2	1	1
SM_17	Mean	299	79	4	3	1	36	150	230	133	2	117	37	7	2	3
	Std. Deviation	110	6	1		1	3		32	13		66	3	3	1	1
SM_18	Mean	554	120	3	3	2	33	150	261	126	2	284	30	3	2	2
	Std. Deviation	146	19	1		1	4		60	17		53	3	1	1	1
SM_19	Mean	194	85	1	3	2	29	150	225	145	2	211	31	6	3	3
	Std. Deviation	161	25			1	4		31	5		29	1	2		1
SM_20	Mean	516	89	4	3	1	33	150	285	146	2	270	33	7	2	3
	Std. Deviation	94	12	1	1	1			#VALUE!	#VALUE!	.	42	.	.	.	.
T_1050V	Mean	295	80	4	3	2	45	150	214	117	2	153	29	7	3	2
	Std. Deviation	128	16	1			4		18	9		14	2	1	1	1
T_1283V	Mean	341	89	3	3	2	33	150	213	162	1	211	30	9	3	3
	Std. Deviation	218	48	1	1	1	3		8	15		49	1	2		1
T_307V	Mean	443	77	5	2	2	36	150	203	150	1	177	31	8	3	3
	Std. Deviation	202	18	1		1			31	16		27	1	2	1	1
T_385V	Mean						42	150	258	155	2		35	5	1	1
	Std. Deviation						.	.	#VALUE!	#VALUE!	.		.	.	.	.
T_738V	Mean	264	85	4	2	3	45	150	216	154	1	180	41	6	3	4
	Std. Deviation	99	2			1	5		6	28		183	4	6	1	1
T_821V	Mean	22	18	5	3	3	32	80	259	174	1	291	26	4	1	3
	Std. Deviation	11	6		1		7	10	16	11		134	5	1		1
T_826V	Mean	573	125	2	3	1	29	150	225	125	2	128	33	3	3	3
	Std. Deviation	48	7	2			3		24	4		49	1	1	2	2
T_830V	Mean	437	97	2	3	2	34	123	194	131	1	163	27	5	3	2
	Std. Deviation	318	59	1		1	4	55	25	7		61	5	3	1	1
VT_007	Mean	245	85	3	3	2	35	150	197	136	1	194	29	5	3	2
	Std. Deviation	94	30	2			6		37	15		113	3	3	1	1
VT_008	Mean	699	100	4	3	2	27	150	199	133	2	307	31	6	3	3
	Std. Deviation	129	5	1		1	2		16	11		26	2	2	1	1
VT_009	Mean	264	70	3	3	3	24	150	227	134	2	610	36	6	4	2
	Std. Deviation	158	7	1	1	1			25	11		136	2	2		1
VT_010	Mean	552	136	1	3	1	31	150	225	139	2	181	30	2	3	3
	Std. Deviation	68	5	1			2		48	18		12	3	1	1	1
VT_012	Mean	412	96	3	3	2	25	150	285	112	3	163	30	4	1	2
	Std. Deviation	94	14	1	1		1		17	17		42	4	1	1	1
VT_013	Mean	251	90	2	2	2	27	150	283	145	2	589	26	3	1	2
	Std. Deviation	55	13	1	1	1	7		23	28		160	5	1	1	1
VT_014	Mean	264	91	2	3	2	29	150	206	134	2	41	33	2	2	4
	Std. Deviation	192	34	1		1	8		6	7		20	2	1	1	1
VT_017	Mean	444	132	1	3	1	31	150	199	146	1	155	30	4	3	3
	Std. Deviation	170	28			1	2		13	16		41	2	2	1	1
VT_018	Mean	391	108	2	3	2	28	150	184	152	1	138	30	7	3	3
	Std. Deviation	147	15	1	1	1	2		8	17		39	2	2		
VT_044	Mean	217	88	1	3	1	34	150	185	135	1	203	32	6	4	2
	Std. Deviation	97	16	1		1	4		2	21		58	2	1	1	1
VT_052	Mean	309	70	6	2	2	30	124	201	117	2	144	32	4	3	2
	Std. Deviation	131	24	1	1	1	2	52	17	18		16	5	2	1	2
VT_053	Mean	196	80	3	2	3	32	150	179	145	1	117	29	6	3	3
	Std. Deviation	29	8	1	1		3		33	28		26	1	3	1	1
VT_089	Mean	698	149	2	3	1	25	150	218	153	1	438	34	5	3	3
	Std. Deviation	215	33	2					26	9		90	4	3	1	
VT_091	Mean	647	116	3	3	2	32	150	196	155	1	285	33	3	3	3
	Std. Deviation	133	24	1		1			9	23		74	3	2	1	1
VT_115	Mean	308	100	2	3	2	23	59	232	122	2	193	33	2	1	2
	Std. Deviation	87	12	1		1		34	22	15		27	3	1		1
VT_117	Mean	335	105	2	3	1	23	51	215	119	2	263	32	5	3	3
	Std. Deviation	41	9	1			1	25	15	16		51	6	3	1	1
VT_120	Mean	494	111	3	3	2	31	150	202	155	1	216	34	4	3	2
	Std. Deviation	143	18	1			4		22	9		123	2	3	1	1
VT_123	Mean	327	53	6	2	2	24	118	245	127	2	609	34	2	2	3
	Std. Deviation	124	2		1		1	64	23	24	1	236	2	1	1	1
VT_137	Mean	333	105	2	3	2	30	150	178	133	1	146	34	5	3	3
	Std. Deviation	138	9	1		1	9		43	22		64	6	4	1	1

Experiment 2011F					
Accession		Twi (mm)	TL (mm)	Tsh	Tl
FT_001	Mean	97.5	132.6	4.3	.7
	Std. Deviation	30.9	31.0	2.5	.2
FT_002	Mean	96.8	132.7	2.5	.7
	Std. Deviation	23.6	32.6	1.9	.2
FT_004	Mean	92.5	160.2	1.3	.6
	Std. Deviation	16.3	22.8	.7	.1
FT_005	Mean	71.7	172.2	4.7	.4
	Std. Deviation	20.9	62.0	2.0	.2
FT_047	Mean	100.3	81.3	1.6	1.2
	Std. Deviation	22.9	14.4	.9	.2
FT_056	Mean	33.1	48.3	1.9	.7
	Std. Deviation	5.7	12.5	1.5	.1
FT_097	Mean	58.7	67.9	3.6	.9
	Std. Deviation	15.1	13.0	2.2	.2
VT_006	Mean	96.6	82.0	2.1	1.2
	Std. Deviation	21.0	11.8	1.1	.3
VT_007	Mean	103.1	70.1	5.3	1.5
	Std. Deviation	26.8	10.6	1.8	.3
VT_008	Mean	93.9	105.9	6.0	.9
	Std. Deviation	20.0	31.5	1.1	.2
VT_009	Mean	44.8	64.3	8.3	.7
	Std. Deviation	9.2	23.8	3.9	.2
VT_010	Mean	106.2	71.3	3.6	1.5
	Std. Deviation	33.2	17.7	2.1	.3
VT_011	Mean	76.6	80.8	1.8	1.0
	Std. Deviation	28.9	20.5	1.6	.3
VT_012	Mean	50.0	77.5	2.5	.6
	Std. Deviation	17.2	12.5	1.4	.2
VT_013	Mean	58.4	61.6	7.4	.9
	Std. Deviation	13.1	5.1	2.3	.2
VT_014	Mean	117.6	66.6	1.1	1.8
	Std. Deviation	15.4	5.2	.4	.3
VT_017	Mean	113.1	56.4	1.1	2.0
	Std. Deviation	24.3	11.6	.3	.4
VT_044	Mean	65.0	47.0	2.1	1.4
	Std. Deviation	20.8	7.9	2.4	.2
VT_045	Mean	115.0	73.6	4.3	1.6
	Std. Deviation	26.5	12.3	2.6	.4
VT_052	Mean	62.3	167.0	3.2	.4
	Std. Deviation	5.6	24.4	1.5	.1
VT_053	Mean	52.8	71.1	2.8	.7
	Std. Deviation	20.2	13.1	2.0	.2
VT_089	Mean	122.4	72.8	5.4	1.7
	Std. Deviation	27.3	9.7	1.9	.3
VT_091	Mean	104.0	106.0	3.6	1.0
	Std. Deviation	28.0	21.0	1.8	.2
VT_092	Mean	108.3	57.7	2.8	1.9
	Std. Deviation	23.6	12.3	1.7	.3
VT_115	Mean	77.8	55.8	9.6	1.4
	Std. Deviation	25.9	11.7	2.9	.3
VT_117	Mean	67.3	164.4	3.2	.4
	Std. Deviation	17.2	39.7	1.7	.1
VT_120	Mean	94.6	86.0	2.3	1.1
	Std. Deviation	26.4	28.2	1.0	.3
VT_123	Mean	50.1	119.3	8.9	.4
	Std. Deviation	16.7	19.2	2.9	.2
VT_137	Mean	82.9	64.3	5.4	1.3
	Std. Deviation	22.0	9.7	3.2	.3
Total	Mean	86.5	91.7	3.7	1.1
	Std. Deviation	32.0	43.4	2.8	.5

Experiment 2012F								
Accession		Twi (mm)	TL (mm)	TI	Brix	TDM (%)	Twe (gram)	TDW (gram)
FT_001	Mean	53.2	90.4	.6	4.6	5.5	283.3	22.7
	Std. Deviation	8.5	11.4	.0	4.0	4.9	264.1	20.0
FT_002	Mean	58.4	86.1	.7	5.0	7.1	259.9	18.2
	Std. Deviation	11.2	13.5	.1	.5	.4	64.1	4.1
FT_003	Mean	31.4	77.2	.4	6.0	8.7	144.4	12.2
	Std. Deviation	5.8	3.0	.1	.3	.6	81.4	6.2
FT_004	Mean	29.6	70.6	.4	5.8	8.7	95.3	7.9
	Std. Deviation	5.5	6.8	.1	.2	1.0	70.6	5.1
FT_005	Mean	35.6	91.7	.4	5.7	8.0	168.8	13.4
	Std. Deviation	8.1	.3	.1	.6	.2	31.2	2.1
FT_047	Mean	40.4	42.8	.9	7.4	8.5	86.9	7.1
	Std. Deviation	20.3	8.1	.5	.9	.6	80.9	6.1
FT_051	Mean	56.5	37.4	1.5	6.3	11.7	54.9	6.2
	Std. Deviation	18.1	9.5	.5	.8	1.0	30.3	2.9
FT_056	Mean	31.2	46.5	.7	6.9	14.1	14.7	2.1
	Std. Deviation	13.1	16.3	.2	.4	.5	7.2	1.1
FT_086	Mean	29.0	34.4	.6	5.1	9.2	64.6	5.9
	Std. Deviation	25.1	29.8	.5	.4	.6	26.4	2.3
FT_088	Mean	39.7	67.7	.6	5.8	9.7	149.0	15.5
	Std. Deviation	13.4	2.6	.2	.5	3.4	68.2	11.6
FT_097	Mean	33.3	59.4	.6	6.0	10.5	68.9	7.1
	Std. Deviation	13.3	1.0	.2	1.1	.8	32.4	2.7
SM_14	Mean	48.9	46.0	.7	5.1	6.3	335.6	21.0
	Std. Deviation	42.4	39.8	.6	.4	.2	162.6	10.2
SM_15	Mean	39.9	26.4	1.0	6.0	6.6	318.5	19.0
	Std. Deviation	34.6	22.8	.9	.3	3.3	139.8	11.1
SM_16	Mean	55.8	79.6	.7	4.6	7.5	256.9	19.1
	Std. Deviation	8.9	17.3	.1	.3	.6	24.4	1.5
SM_17	Mean	45.7	70.9	.6	5.9	9.0	189.9	17.0
	Std. Deviation	3.3	4.5	.1	.8	.6	9.6	.3
SM_18	Mean	41.3	32.5	.8	6.2	8.3	207.8	17.2
	Std. Deviation	35.8	28.1	.7	.7	.2	9.9	.7
SM_19	Mean	31.7	33.3	.9	6.5	11.9	22.4	2.6
	Std. Deviation	10.7	3.1	.3	1.8	1.2	12.1	1.2
SM_20	Mean	26.7	46.3	.4	5.8	8.8	113.8	9.9
	Std. Deviation	23.1	40.1	.3	.8	.7	31.8	2.3
T_1050V	Mean	32.2	46.8	.5	5.8	10.5	70.3	7.4
	Std. Deviation	29.8	40.5	.4	.4	.4	57.3	6.1
T_1283V	Mean	36.0	48.4	.5	5.8	8.3	227.8	18.8
	Std. Deviation	31.3	41.9	.4	.8	.2	39.2	2.8
T_163V	Mean	24.4	36.6	.4	5.5	15.2	62.5	9.5
	Std. Deviation	22.4	31.7	.4	1.0	.7	9.8	1.7
T_307V	Mean	25.2	53.3	.3	5.1	8.0	186.2	14.9
	Std. Deviation	22.0	46.2	.3	.9	.3	3.7	.9
T_385V	Mean	19.4	37.0	.4	5.9	9.7	81.2	7.8
	Std. Deviation	18.6	32.0	.3	.7	.5	35.8	3.1
T_738V	Mean	29.9	47.0	.4	5.6	10.0	121.3	11.5
	Std. Deviation	26.3	40.7	.4	1.6	1.2	120.1	10.8
T_826V	Mean	42.8	41.6	.7	5.9	7.8	231.6	18.0
	Std. Deviation	39.7	36.0	.6	1.0	1.4	67.7	6.3
T_830V	Mean	21.0	.0	.0	2.2	3.4	18.5	1.9
	Std. Deviation	36.4	.0	.0	3.8	5.9	32.1	3.3
T_pancai	Mean	38.6	31.4	.8	6.1	8.6	69.4	5.9
	Std. Deviation	38.7	27.2	.8	.2	.5	21.6	1.5
T_RR	Mean	67.1	64.7	1.0	6.4	9.6	294.8	27.6
	Std. Deviation	11.5	7.2	.2	1.2	1.0	71.9	3.5
VT_006	Mean	58.1	76.0	.8	5.2	7.9	168.8	13.4
	Std. Deviation	11.4	12.0	.2	.1	.0	42.0	3.3
VT_007	Mean	48.8	61.3	.8	5.6	9.1	94.5	8.5
	Std. Deviation	20.2	8.3	.3	.6	.5	61.1	5.2
VT_008	Mean	41.7	55.6	.5	5.3	7.1	330.4	23.4
	Std. Deviation	38.3	48.1	.5	.2	.4	105.9	7.1
VT_009	Mean	52.5	63.7	.8	4.8	9.6	127.1	12.1
	Std. Deviation	10.3	4.7	.2	.9	.8	32.6	2.6
VT_010	Mean	69.3	57.2	1.2	5.7	8.1	193.4	15.1
	Std. Deviation	11.0	8.2	.2	.6	.6	120.5	8.8
VT_011	Mean	41.0	62.3	.7	5.9	9.4	40.6	3.7
	Std. Deviation	16.8	17.3	.3	.9	1.0	18.3	1.2
VT_012	Mean	59.4	61.0	1.0	5.5	9.0	217.7	20.5
	Std. Deviation	13.6	1.9	.2	.8	2.0	80.0	12.1
VT_013	Mean	42.9	42.8	.7	3.5	4.8	232.7	16.5
	Std. Deviation	37.2	37.1	.6	3.0	4.2	226.4	15.4
VT_014	Mean	55.5	30.2	1.8	4.3	6.5	104.6	6.5
	Std. Deviation	16.0	.0	.5	.6	.4	76.5	4.4
VT_015	Mean	67.2	39.7	1.7	5.9	8.8	218.0	18.9
	Std. Deviation	1.9	2.2	.1	.2	.4	123.5	10.1
VT_017	Mean	47.1	20.9	1.5	6.2	7.6	134.5	9.9
	Std. Deviation	41.2	18.1	1.3	.3	.3	116.5	8.2
VT_018	Mean	56.4	50.6	1.1	5.4	8.6	155.6	13.3
	Std. Deviation	6.2	5.6	.0	1.2	.4	41.2	3.3
VT_044	Mean	.0	.0	.0	.0	.0	.0	.0
	Std. Deviation	.0	.0	.0	.0	.0	.0	.0
VT_045	Mean	60.1	49.3	1.2	6.6	9.1	260.9	22.1
	Std. Deviation	11.6	.4	.2	.9	1.3	180.5	13.4
VT_052	Mean	43.6	76.2	.6	5.8	7.2	237.8	16.4
	Std. Deviation	8.8	20.4	.1	.2	.7	201.3	12.5
VT_053	Mean	45.2	62.8	.7	8.2	19.5	49.8	9.8
	Std. Deviation	2.9	6.6	.1	.6	6.3	21.1	4.7
VT_089	Mean	65.6	59.6	1.1	4.8	8.2	201.3	16.5
	Std. Deviation	5.5	1.6	.1	.5	.4	55.6	4.3
VT_090	Mean	33.4	88.6	.4	4.5	6.6	144.9	9.6
	Std. Deviation	2.4	4.2	.0	.6	.1	19.4	1.2
VT_091	Mean	44.6	55.9	.8	5.6	8.2	172.6	13.9
	Std. Deviation	4.5	7.4	.2	.3	.3	114.5	8.8
VT_092	Mean	70.0	45.0	1.6	6.0	8.0	349.4	27.8
	Std. Deviation	3.5	8.9	.4	.6	.1	69.3	5.4
VT_115	Mean	34.6	27.0	.9	5.3	6.6	131.0	8.6
	Std. Deviation	30.5	23.4	.8	.5	.3	27.2	2.0
VT_116	Mean	70.2	49.2	1.4	4.9	6.2	351.7	21.9
	Std. Deviation	6.2	2.6	.2	.5	.2	97.4	6.3
VT_117	Mean	72.0	49.7	1.5	5.3	8.2	234.0	18.8
	Std. Deviation	4.5	5.8	.1	.5	.8	94.8	7.0
VT_119	Mean	31.3	77.9	.4	6.1	8.5	146.0	12.4
	Std. Deviation	5.0	6.2	.0	.7	.6	11.0	1.5
VT_120	Mean	53.8	58.2	.9	5.9	8.8	188.5	16.3
	Std. Deviation	3.1	6.8	.2	1.0	.8	62.8	3.9
VT_123	Mean	29.4	76.4	.4	5.8	9.8	144.9	14.1
	Std. Deviation	8.1	1.6	.1	1.4	.5	20.0	1.2
VT_137	Mean	37.9	27.5	.9	4.1	6.2	442.2	26.7
	Std. Deviation	33.2	23.9	.8	.6	.5	175.8	8.5
