# Supplementary material for: Morphology, Carbohydrate Composition and Vernalization Response in a Genetically Diverse Collection of Asian and European Turnips (Brassica rapa subsp. rapa)
Source: PLoS One. 2014 Dec 4;9(12):e114241. doi: 10.1371/journal.pone.0114241 (PMC4256417; doi:10.1371/journal.pone.0114241)
Supplement: Table S6 — Restults of analysis of variance (ANOVA) for each trait in five experiments. The presented traits are leaf color (LC), leaf length (LL), lamina blade length (LBL), lamina blade width (LBW), leaf index (LI), petiole length (PL), petiole width (PW), leaf lobe (LB), leaf lobelets (LBs), leaf edge shape (LES), leaf blade shape outline (LS), leaf division (LD), leaf apex shape (LAS), leaf hairiness (LH), leaf lamina attitude (LAT), leaf and stem weight (Lwe), flowering time (FT), tuber length (TL), tuber width (Twi), tuber index (TI), tuber shape (TS), tuber color (TC), tuber shoots number (Tsh), tuber weight (Twe), tuber dry weight (TDW), tuber surface smoothness (Tss), tuber swelling onset (Tso), tuber growing depth (Tgd) and tuber dry mass% (TDM). (PDF) [file pone.0114241.s013.pdf]

Table S6 Restults of analysis of variance (ANOVA) for each trait in five experiments.

The presented traits are leaf color (LC), leaf length (LL), lamina blade length (LBL), lamina blade width (LBW), leaf index (LI), petiole petiole width (PW), leaf lobe (LB), leaf lobelets (LBs), leaf edge shape (LES), leaf blade shape outline (LS), leaf division (LD), leaf a leaf hairiness (LH), leaf lamina attitude (LAT), leaf and stem weight (Lwe), flowering time (FT), tuber length (TL), tuber width (Twi), tu tuber shape (TS), tuber color (TC), tuber shoots number (Tsh), tuber weight (Twe), tuber dry weight (TDW), tuber surface smoothnes tuber swelling onset (Tso), tuber growing depth (Tgd) and tuber dry mass% (TDM).

Experiment	Traits			Sum of Squares	df	Mean Square	F	Sig.
Experiment 2008F*	LL * Accession	Between Groups	(Combined)	6468.105	40	119.780	3.150	.000
		Within Groups		4183.136	87	38.029		
		Total		10651.241	127			
	LBW * Accession	Between Groups	(Combined)	1843.758	40	34.144	2.527	.000
		Within Groups		1486.076	87	13.510		
		Total		3329.834	127			
	LLL * Accession	Between Groups	(Combined)	5861.047	40	108.538	3.116	.000
		Within Groups		3831.896	87	34.835		
		Total		9692.943	127			
	LI * Accession	Between Groups	(Combined)	22.228	40	.412	1.821	.004
		Within Groups		24.868	87	.226		
		Total		47.097	127			
	PW * Accession	Between Groups	(Combined)	10.024	40	.186	1.741	.007
		Within Groups		11.732	87	.107		
		Total		21.755	127			
	LPL * Accession	Between Groups	(Combined)	1981.014	40	36.685	6.717	.000
		Within Groups		600.767	87	5.462		
		Total		2581.781	127			
	LS * Accession	Between Groups	(Combined)	140.509	42	2.602	1.101	.331
		Within Groups		260.000	123	2.364		
		Total		400.509	165			
	LDM * Accession	Between Groups	(Combined)	112.945	40	2.092	1.613	.018
		Within Groups		142.667	87	1.297		
		Total		255.612	127			
	LDI * Accession	Between Groups	(Combined)	59.782	40	1.107	3.971	.000
		Within Groups		30.667	87	.279		
		Total		90.448	127			
	LB * Accession	Between Groups	(Combined)	997.903	42	18.480	5.082	.000
		Within Groups		400.000	123	3.636		
		Total		1397.903	165			
	LAS * Accession	Between Groups	(Combined)	135.903	42	2.517	1.821	.004
		Within Groups		152.000	123	1.382		
		Total		287.903	165			
	Twi * Accession	Between Groups	(Combined)	55702.9	40	1392.6	2.428	.000
		Within Groups		49907.8	87	573.7		
		Total		105610.7	127			
	TL * Accession	Between Groups	(Combined)	247958.9	40	6199.0	9.516	.000
		Within Groups		56677.0	87	651.5		
		Total		304635.9	127			
	TI * Accession	Between Groups	(Combined)	32.7	40	.8	10.121	.000
		Within Groups		7.0	87	.1		
		Total		39.7	127			
	FT * Accession	Between Groups	(Combined)	84003.7	42	2000.1	16.954	.000
		Within Groups		14510.3	123	118.0		
		Total		98514.0	165			

Experiment	Traits		Sum of Squares	df	Mean Square	F	Sig.
Experiment 2009F*	FT * Accession	Between Groups (Combined)	66610.4	42	1586.0	4163.2	.000
		Within Groups	16.0	42	.4		
		Total	66626.4	84			
	LC * Accession	Between Groups (Combined)	1887.2	42	44.9	2.8	.000
		Within Groups	3506.2	215	16.3		
		Total	5393.5	257			
	Twe * Accession	Between Groups (Combined)	26897269.3	38	707822.9	2.4	.002
		Within Groups	14376644.5	49	293400.9		
		Total	41273913.8	87			
	TL * Accession	Between Groups (Combined)	239586.1	41	5843.6	15.1	.000
		Within Groups	19728.5	51	386.8		
		Total	259314.6	92			
	Twi * Accession	Between Groups (Combined)	104434.1	41	2547.2	5.4	.000
		Within Groups	24277.2	51	476.0		
		Total	128711.3	92			
	Tsh * Accession	Between Groups (Combined)	1406.4	41	34.3	5.6	.000
		Within Groups	413.9	67	6.2		
		Total	1820.3	108			
	TI * Accession	Between Groups (Combined)	20.4	41	.5	12.5	.000
		Within Groups	2.0	51	.0		
		Total	22.4	92			
Experiment 2010G*	Twe * Accession	Between Groups (Combined)	3465986	39	88871.4	3.954	.000
		Within Groups	2247674	100	22476.7		
		Total	5713660	139			
	Twi * Accession	Between Groups (Combined)	91813	39	2354.2	4.982	.000
		Within Groups	47257	100	472.6		
		Total	139070	139			
	Tsh * Accession	Between Groups (Combined)	247	39	6.3	10.517	.000
		Within Groups	61	101	.6		
		Total	308	140			
	Tgd * Accession	Between Groups (Combined)	33	39	.8	7.061	.000
		Within Groups	12	99	.1		
		Total	44	138			
	Tss * Accession	Between Groups (Combined)	39	39	1.0	3.545	.000
		Within Groups	28	99	.3		
		Total	67	138			
	Tso * Accession	Between Groups (Combined)	4163	40	104.1	6.077	.000
		Within Groups	1884	110	17.1		
		Total	6047	150			
	FT * Accession	Between Groups (Combined)	91305	40	2282.6	6.777	.000
		Within Groups	38397	114	336.8		
		Total	129702	154			
	LBL * Accession	Between Groups (Combined)	983	40	24.6	3.863	.000
		Within Groups	458	72	6.4		
		Total	1441	112			
	LBW * Accession	Between Groups (Combined)	251	40	6.3	2.267	.001
		Within Groups	199	72	2.8		
		Total	450	112			
	LI * Accession	Between Groups (Combined)	11	40	.3	5.876	.000
		Within Groups	3	72	.0		
		Total	14	112			
	Lwe * Accession	Between Groups (Combined)	5124263	39	131391.4	13.128	.000
		Within Groups	980860	98	10008.8		
		Total	6105123	137			
	LC * Accession	Between Groups (Combined)	1084	40	27.1	2.655	.000
		Within Groups	766	75	10.2		
		Total	1849	115			
	PW * Accession	Between Groups (Combined)	366	40	9.1	2.089	.003
		Within Groups	324	74	4.4		
		Total	690	114			
	LES * Accession	Between Groups (Combined)	68	40	1.7	2.533	.000
		Within Groups	50	75	.7		
		Total	118	115			
	LAT * Accession	Between Groups (Combined)	33	40	.8	1.011	.473
		Within Groups	61	75	.8		
		Total	94	115			

Experiment	Traits			Sum of Squares	df	Mean Square	F	Sig.
Experiment 2011F*	Turnip width (mm) * Accession	Between Groups	(Combined)	144622.795	28	5165.100	9.889	.000
		Within Groups		120127.352	230	522.293		
		Total		264750.147	258			
	Turnip length (mm) * Accession	Between Groups	(Combined)	362676.486	28	12952.732	24.141	.000
		Within Groups		123405.290	230	536.545		
		Total		486081.776	258			
	Nr.shoots (tillers) * Accession	Between Groups	(Combined)	1078.256	28	38.509	10.094	.000
		Within Groups		877.443	230	3.815		
		Total		1955.699	258			
	shape index (width/length) * Accession	Between Groups	(Combined)	57.043	28	2.037	33.637	.000
		Within Groups		13.930	230	.061		
		Total		70.972	258			
Experiment 2012F*	Twi * Accession	Between Groups	(Combined)	37302.413	54	690.785	1.569	.024
		Within Groups		48432.497	110	440.295		
		Total		85734.910	164			
	TL * Accession	Between Groups	(Combined)	68296.485	54	1264.750	2.858	.000
		Within Groups		48682.900	110	442.572		
		Total		116979.384	164			
	TI * Accession	Between Groups	(Combined)	26.937	54	.499	2.985	.000
		Within Groups		18.383	110	.167		
		Total		45.320	164			
	Brix * Accession	Between Groups	(Combined)	223.392	54	4.137	3.344	.000
		Within Groups		136.100	110	1.237		
		Total		359.492	164			
	Drymass * Accession	Between Groups	(Combined)	1197.114	54	22.169	7.450	.000
		Within Groups		327.327	110	2.976		
		Total		1524.441	164			
	Twe * Accession	Between Groups	(Combined)	1601339.539	54	29654.436	3.574	.000
		Within Groups		912701.340	110	8297.285		
		Total		2514040.879	164			
	TDW * Accession	Between Groups	(Combined)	7376.599	54	136.604	2.914	.000
		Within Groups		5157.193	110	46.884		
		Total		12533.792	164			

*Experiment code “2008F, 2009F, 2011F and 2012F” stand for four field experiments carried out between 2008 and 201
Code “2010G” means the greenhouse experiment in 2010.
