# Supplementary material for: Morphology, Carbohydrate Composition and Vernalization Response in a Genetically Diverse Collection of Asian and European Turnips (Brassica rapa subsp. rapa)
Source: PLoS One. 2014 Dec 4;9(12):e114241. doi: 10.1371/journal.pone.0114241 (PMC4256417; doi:10.1371/journal.pone.0114241)
Supplement: Table S7 — Heritability of eleven traits that were evaluated in multiple experiments. (PDF) [file pone.0114241.s014.pdf]

Table S7 Heritability of eleven traits in different experiments.

Trait	Random term	Estimated variance components					
		overall experiments	per experiment*				
			2008F	2009F	2010G	2011F	2012F
Twi (Tuber width)	Accession	235.7	288.7	970.4	551.4	549	133.8
	Experiment	1429.2	-	-	-	-	-
	Accession.Experiment	189.3	-	-	-	-	-
	Residual	451.8	542.7	481.9	473.6	523.4	113.5
	**Heritability (h^2)**	**0.27**	**0.35**	**0.67**	**0.54**	**0.51**	**0.54**
Twe (Tuber weight)	Accession	18952	-	185353	19107	-	73726
	Experiment	325514	-	-	-	-	-
	Accession.Experiment	76065	-	-	-	-	-
	Residual	94104	-	291720	22490	-	64259
	**Heritability (h^2)**	**0.10**	**-**	**0.39**	**0.46**	**-**	**0.53**
TL (Tuber length)	Accession	826.2	1770.5	2437.3	-	1375.5	230.2
	Experiment	1069.4	-	-	-	-	-
	Accession.Experiment	441.6	-	-	-	-	-
	Residual	450.5	663.8	386.1	-	534.7	58.25
	**Heritability (h^2)**	**0.48**	**0.73**	**0.86**	**-**	**0.72**	**0.80**
TI (Tuber index)	Accession	0.15169	0.2393	0.21523	-	0.21171	0.1558
	Experiment	0.0256	-	-	-	-	-
	Accession.Experiment	0.03532	-	-	-	-	-
	Residual	0.0594	0.0801	0.04	-	0.0605	0.0482
	**Heritability (h^2)**	**0.62**	**0.75**	**0.84**	**-**	**0.78**	**0.76**
Tsh (Tuber shoot number)	Accession	1.88	-	11.594	1.6738	4.747	-
	Experiment	11.805	-	-	-	-	-
	Accession.Experiment	3.944	-	-	-	-	-
	Residual	3.436	-	6.198	0.603	3.827	-
	**Heritability (h^2)**	**0.20**	**-**	**0.65**	**0.74**	**0.55**	**-**
FT (Flowering time)	Accession	358.1	594.6	768.8598	498.2	-	-
	Experiment	2.7	-	-	-	-	-
	Accession.Experiment	225.3	-	-	-	-	-
	Residual	190.9	118.5	0.381	335.3	-	-
	**Heritability (h^2)**	**0.46**	**0.83**	**1.00**	**0.60**	**-**	**-**
LC (Leaf color)	Accession	4.33	5.65	4.77	6.05	-	-
	Experiment	26.35	-	-	-	-	-
	Accession.Experiment	0.5	-	-	-	-	-
	Residual	14.78	18.28	16.31	10.26	-	-
	**Heritability (h^2)**	**0.22**	**0.24**	**0.23**	**0.37**	**-**	**-**
LBL (Leaf blade length)	Accession	0	3.319	-	6.638	-	-
	Experiment	4.726	-	-	-	-	-
	Accession.Experiment	4.621	-	-	-	-	-
	Residual	8.027	9.081	-	6.42	-	-
	**Heritability (h^2)**	**0.00**	**0.27**	**-**	**0.51**	**-**	**-**
LBW (Leaf blade width)	Accession	3.52	3.319	-	1.285	-	-
	Experiment	305.16	-	-	-	-	-
	Accession.Experiment	2.53	-	-	-	-	-
	Residual	10.16	9.081	-	2.753	-	-
	**Heritability (h^2)**	**0.22**	**0.27**	**-**	**0.32**	**-**	**-**
LI (Leaf index)	Accession	0.0457	0.0276	-	0.0826	-	-
	Experiment	0.1523	-	-	-	-	-
	Accession.Experiment	0.0032	-	-	-	-	-
	Residual	0.1111	0.156	-	0.0463	-	-
	**Heritability (h^2)**	**0.29**	**0.15**	**-**	**0.64**	**-**	**-**
PW (Petiole)	Accession	0	0.0105	-	1.719	-	-
	Experiment	6.046	-	-	-	-	-
	Accession.Experiment	0.732	-	-	-	-	-
	Residual	1.862	0.0922	-	4.361	-	-
	**Heritability (h^2)**	**0.00**	**0.10**	**-**	**0.28**	**-**	**-**

*Experiment code “2008F, 2009F,2011F and 2012F” stand for four field experiments carried out between 2008 and Code “2010G” means the greenhouse experiment in 2010.
