# Supplementary material for: Morphology, Carbohydrate Composition and Vernalization Response in a Genetically Diverse Collection of Asian and European Turnips (Brassica rapa subsp. rapa)
Source: PLoS One. 2014 Dec 4;9(12):e114241. doi: 10.1371/journal.pone.0114241 (PMC4256417; doi:10.1371/journal.pone.0114241)
Supplement: Table S8 — Descriptive of phenotypic variation between Asian and European subpopulations without vernalization for each trait from five independent experiments. (PDF) [file pone.0114241.s015.pdf]

Table S8 **Descriptives of phenotypic variation between Asian and European subpopulations without vernalization for each trait from five independent experiment.**

Trait code	Trait	Sub-population	2008F*		2009F*		2010G*		2011F*		2012F*	
			Median	Range	Median	Range	Median	Range	Median	Range	Median	Range
FT (day)	flowering time	Asian	150	60-150	94	48-150	134	51-150	-	-	-	-
		European	150	43-150	150	95-150	150	123-150	-	-	-	-
LAS	Leaf apex shape	Asian	2	2-3	-	-	-	-	-	-	-	-
		European	2	2-4	-	-	-	-	-	-	-	-
LAT	Leaf lamina attitude	Asian	2	2-3	-	-	-	-	-	-	-	-
		European	3	2-4	-	-	-	-	-	-	-	-
LB	Leaf Lobe	Asian	5	1-9	-	-	-	-	-	-	-	-
		European	6	1-12	-	-	-	-	-	-	-	-
LBs	Leaf Lobelets	Asian	5	0-7	-	-	-	-	-	-	-	-
		European	6	0-15	-	-	-	-	-	-	-	-
LD	Leaf division (incision)	Asian	3	1-3	-	-	-	-	-	-	-	-
		European	3	1-4	-	-	-	-	-	-	-	-
LH	Leaf hairinness	Asian	1	1-2	-	-	-	-	-	-	-	-
		European	2	1-4	-	-	-	-	-	-	-	-
LS	Leaf blade shape outline	Asian	3	1-5	-	-	-	-	-	-	-	-
		European	3	1-7	-	-	-	-	-	-	-	-
LL (cm)	Leaf length	Asian	47.6	31.1-56.0	-	-	-	-	-	-	-	-
		European	47.8	20.3-58.8	-	-	-	-	-	-	-	-
PL (cm)	Petiole length	Asian	7.4	0-27.6	-	-	-	-	-	-	-	-
		European	2.1	0-12.9	-	-	-	-	-	-	-	-
LES	Leaf edge shape	Asian	1	1-1	-	-	2	1-4	-	-	-	-
		European	1	1-3	-	-	3	2-4	-	-	-	-
PW (cm)	Petiole width	Asian	1.2	0.5-2.8	-	-	0.4	0.1-0.6	-	-	-	-
		European	1.1	0.7-4.9	-	-	0.5	0.2-1.0	-	-	-	-
LBL (cm)	Lamina blade length	Asian	19.9	7.7-48.6	-	-	24.8	19.7-28.5	-	-	-	-
		European	13.9	9.7-51.6	-	-	20.2	16.6-28.5	-	-	-	-
LBW (cm)	Lamina blade width	Asian	12.1	9.2-20.7	-	-	13	11.0-17.4	-	-	-	-
		European	15.9	5.7-26.1	-	-	14.2	11.7-16.2	-	-	-	-
LI	Leaf index	Asian	3.9	1.9-4.7	-	-	1.7	1.3-2.1	-	-	-	-
		European	2.9	1.5-6.9	-	-	1.4	1.1-2.6	-	-	-	-
LC	Leaf color	Asian	38.2	34.4-45.9	42.3	39.6-46.4	30.9	26.2-36	-	-	-	-
		European	37.5	30.3-52.3	41.2	36.9-50.7	31.4	26.3-41.3	-	-	-	-
Lwe (gram)	Leaf and stem weight	Asian	-	-	-	-	228.3	154.8-609.7	-	-	-	-
		European	-	-	-	-	178.4	40.0-1058.8	-	-	-	-

Trait code	Trait	Sub-population	2008F*		2009F*		2010G*		2011F*		2012F*	
			Median	Range	Median	Range	Median	Range	Median	Range	Median	Range
Twi (cm)	Tuber width	Asian	13.4	9.2-15.8	14.3	9.5-15.6	8.5	1.8-10.5	6.6	4.5-10.2	5.8	2.9-7.3
		European	13.1	8.0-18.8	15.4	7.8-23.9	9	2.0-14.9	9.4	3.2-12.2	4.7	2.9-7.1
Twe (gram)	Tuber weight	Asian	-	-	1105.5	449.0-1580.	257.7	22.0-412.3	-	-	202.7	62.3-441.7
		European	-	-	1474.7	553-3-2773.	366	18.7-699.3	-	-	147	14.3-424.5
TL (cm)	Tuber length	Asian	10.6	6.0-17.6	10.5	7.7-17.5	-	-	7.3	5.5-16.5	6.8	4.3-7.8
		European	13.5	6.4-22.8	13.2	8.0-29.5	-	-	7.3	4.6-16.8	6.2	3.7-9.7
TI	Tuber index	Asian	0.8	0.5-1.2	1.2	0.7-1.7	-	-	0.4	0.2-1.3	0.9	0.4-1.4
		European	1	0.5-2.7	1.2	0.4-2.2	-	-	0.7	0.2-1.9	0.8	0.4-1.6
Tsh	Tuber shoots number	Asian	-	-	12	5-23	3	2-6	7	2-10	-	-
		European	-	-	9	2-14	2	1-6	3	1-6	-	-
TC	Tuber color	Asian	3	2-5	4	2-8	-	-	-	-	-	-
		European	2	1-5	2	2-6	-	-	-	-	-	-
TDM (%)	tuber dry mass%	Asian	-	-	-	-	-	-	-	-	8.3	6.2-15.2
		European	-	-	-	-	-	-	-	-	8.5	6.5-19.5
TDW (gram)	Tuber dry weight	Asian	-	-	-	-	-	-	-	-	17.2	5.7-27.3
		European	-	-	-	-	-	-	-	-	12.8	2.0-33.5
Tgd	Tuber growing depth	Asian	-	-	-	-	3	2-3	-	-	-	-
		European	-	-	-	-	3	1-3	-	-	-	-
TS	Tuber shape	Asian	1	1-5	-	-	-	-	-	-	-	-
		European	2	1-5	-	-	-	-	-	-	-	-
Tso (day)	Tuber swelling onset	Asian	-	-	-	-	25	23-35	-	-	-	-
		European	-	-	-	-	32	25-47	-	-	-	-
Tss	Tuber surface smoothness	Asian	-	-	-	-	2	1-3	-	-	-	-
		European	-	-	-	-	2	1-3	-	-	-	-

*Experiment code “2008F, 2009F, 2011F and 2012F” stand for four field experiments carried out between 2008 and 2012. Code “2010G” means the greenhouse experime
