# Supplementary material for: Morphology, Carbohydrate Composition and Vernalization Response in a Genetically Diverse Collection of Asian and European Turnips (Brassica rapa subsp. rapa)
Source: PLoS One. 2014 Dec 4;9(12):e114241. doi: 10.1371/journal.pone.0114241 (PMC4256417; doi:10.1371/journal.pone.0114241)
Supplement: Table S9 — Descriptive of phenotypic variation between Asian and European subpopulations with and without vernalization for each trait from five independent experiments. Within the same trait, same accession, the values accompanied by the common letter show no significant difference between the treatments. The presented traits are flowering time (FT), tuber weight (Twe), tuber width (Twi), tuber shoots number (Tsh) tuber growing depth (Tgd), tuber swelling onset (Tso), tuber surface smoothness (Tss), tuber color (TC), leaf and stem weight (Lwe), leaf length (LL), lamina blade width (LBW), leaf index (LI), leaf color (LC), petiole width (PW), leaf blade shape outline (LS), leaf lamina attitude (LAT) and leaf edge shape (LES). (PDF) [file pone.0114241.s016.pdf]

Table S9 **Descriptives of phenotypic variation between Asian and European subpopulations with and without vernalization for each trait from five independent experiment.**

Within the same trait, same accession, the values accompanied by the common letter show no significant difference between the treatments

The presented traits are flowering time (FT), tuber weight (Twe), tuber width (Twi), tuber shoots number (Tsh) tuber growing depth (Tgd), tuber swelling onset (Tso), tuber surface smoothness (Tss), tuber color (TC), leaf and stem weight (Lwe), leaf length (LL), lamina blade width (LBW), leaf index (LI), leaf color (LC), petiole width (PW), leaf blade shape outline (LS), leaf lamina attitude (LAT) and leaf edge shape (LES).

Accession	Subpopulation	Vernalization (week)	FT (days)	Twe (gram)	Twi (mm)	TSh	Tgd	Tso	Tss	TC	Lwe (gram)	LL (cm)	LBW (cm)	LI	LC	PW (mm)	LS	LAT	LES
T-1050V	European	0	150 a	294.5 a	79.5 a	4 a	3 a	45 a	2 a	2 -	152.8 a	21 a	12 a	2 a	29 a	7 a	2 a	3 a	3 ab
T-1050V		4	130 a	339.7 a	79.3 a	4 a	3 a	34 b	3 a	2 -	190.0 a	21 a	14 a	2 ab	30 a	6 a	2 a	2 a	4 b
T-1050V		8	44 b	37.0 b	38.0 a	4 a	3 a	37 ab	3 a	2 -	233.5 a	18 a	14 a	1 b	26 a	7 a	2 a	2 a	3 a
T-1283V	European	0	150 a	341.3 ab	88.5 a	3 ab	3 a	33 a	2 a	2 -	210.5 a	21 a	16 a	1 a	30 a	9 a	2 a	2 a	3 ab
T-1283V		4	150 a	417.3 a	108.5 a	2 a	3 a	30 a	2 a	2 -	166.3 a	19 a	14 a	1 a	29 a	6 a	3 a	3 a	4 a
T-1283V		8	124 a	105.3 b	58.7 a	3 b	1 b	31 a	3 b	2 -	292.7 a	22 a	16 a	1 a	31 a	6 a	2 a	3 a	3 b
T-307V	European	0	150 a	442.7 a	77.3 a	5 a	2 a	36 a	2 a	3 a	177.3 a	20 a	15 a	1 a	31 a	8 a	2 ab	3 a	3 a
T-307V		4	150 a	503.8 a	84.8 a	5 a	2 a	30 b	2 a	5 a	405.0 a	21 a	16 a	1 a	34 a	9 a	2 a	3 a	3 a
T-307V		8	132 a	477.8 a	79.3 a	5 a	2 a	32 b	2 a	4 a	288.5 a	19 a	14 a	1 a	35 a	10 a	3 b	3 a	4 a
T-821V	European	0	80 a	22.0 a	18.3 a	5 a	3 a	32 a	3 a	2 a	291.3 a	26 a	17 a	1 a	26 a	4 a	1 a	3 a	1 -
T-821V		4	40 b	10.3 b	15.8 a	5 a	1 b	31 a	2 a	2 a	260.5 a	23 a	15 a	2 a	30 a	3 a	2 a	3 a	1 -
T-821V		8	42 b	8.5 b	15.3 a	5 a	2 b	50 b	3 a	2 a	231.8 a	25 a	14 a	2 a	31 a	3 a	1 a	2 a	1 -
T-830V	European	0	123 a	436.8 a	97.3 a	2 a	3 a	34 a	2 a	2 -	163.3 a	19 a	13 a	1 a	27 a	5 a	2 a	2 a	3 a
T-830V		4	150 a	442.7 a	113.3 a	1 a	3 a	31 a	2 a	2 -	190.7 a	21 a	14 a	1 a	26 a	6 a	2 a	3 a	4 a
T-830V		8	50 b	110.0 a	66.3 a	2 a	3 a	37 a	3 a	2 -	299.3 a	20 a	15 a	1 a	24 a	7 a	2 a	3 a	4 a
W-FT-002	European	0	150 -	441.7 a	110.0 a	2 a	2 a	27 a	2 a	5 a	158.0 a	21 a	16 a	1 a	33 a	3 ab	2 -	3 a	3 a
W-FT-002		4	150 -	417.0 a	106.8 a	3 b	3 a	28 a	2 a	5 a	113.0 a	21 a	16 a	1 a	31 a	7 a	2 -	4 a	3 a
W-FT-002		8	150 -	163.7 a	99.7 a	4 b	2 a	32 a	2 a	5 a	103.3 a	22 a	17 a	1 a	35 a	1 b	2 -	2 a	3 a
W-FT-004	European	0	150 a	477.3 a	76.0 a	5 a	2 a	33 a	2 a	2 a	492.3 a	20 a	14 a	1 a	31 a	6 a	2 a	2 a	4 a
W-FT-004		4	150 a	307.3 ab	69.8 a	4 a	2 a	32 a	2 a	2 a	395.8 a	19 a	15 a	1 a	31 a	5 a	2 a	3 a	3 a
W-FT-004		8	91 a	183.7 b	60.5 a	4 a	2 a	33 a	2 a	3 b	431.7 a	19 a	14 a	1 a	31 a	7 a	2 a	3 a	3 a
W-FT-047	European	0	150 -	265.0 a	88.7 a	2 a	3 -	34 a	2 a	4 a	149.0 a	20 a	15 a	1 a	29 a	4 a	2 -	2 a	3 a
W-FT-047		4	150 -	281.5 a	80.5 a	3 a	3 -	43 a	3 a	4 a	309.5 a	19 a	12 a	2 a	29 a	4 a	2 -	3 a	3 a
W-FT-047		8	150 -	418.0 a	103.0 a	2 a	3 -	35 a	2 a	4 a	252.0 a	21 a	16 a	1 a	31 a	7 a	2 -	2 a	3 a
W-FT-051	European	0	150 a	232.3 a	90.8 a	1 a	2 a	32 a	2 a	7 a	122.0 a	19 a	12 a	2 a	32 a	6 a	2 a	3 a	2 a
W-FT-051		4	108 a	190.5 a	68.0 ab	4 ab	2 a	33 a	2 a	8 a	328.5 a	19 a	13 a	1 a	34 a	4 a	2 a	2 a	2 a
W-FT-051		8	30 b	18.8 b	25.8 b	5 b	2 a	29 a	3 a	7 a	272.0 a	20 a	13 a	2 a	38 a	3 a	2 a	2 a	2 a
W-FT-056	European	0	150 a	18.7 a	20.0 a	5 a	1 a	47 a	2 a	2 -	460.0 a	17 a	15 a	1 a	27 a	2 a	2 a	3 a	2 a
W-FT-056		4	118 a	13.5 a	18.5 a	5 a	2 a	48 a	3 a	2 -	538.3 a	18 a	15 a	1 a	29 a	6 ab	2 a	3 a	3 a
W-FT-056		8	31 b	9.3 a	20.3 a	4 a	2 a	45 a	2 a	2 -	282.5 a	19 a	16 a	1 a	28 a	7 b	3 a	3 a	4 a
W-FT-086	European	0	150 -	320.0 a	98.0 a	2 a	3 a	29 a	3 a	6 a	128.3 a	18 a	12 a	2 a	36 a	5 a	2 a	3 a	3 a
W-FT-086		4	150 -	295.8 a	102.3 a	1 a	3 a	30 a	3 a	6 a	130.3 a	20 a	12 a	2 a	29 b	4 a	2 a	2 a	4 a
W-FT-086		8	150 -	304.0 a	99.7 a	2 a	3 a	29 a	2 a	7 a	183.3 a	20 a	15 a	1 a	31 ab	4 a	1 a	2 a	3 a
W-FT-097	European	0	150 -	126.0 a	50.3 a	4 a	2 a	37 a	3 a	2 -	##### a	20 a	16 a	1 a	34 a	3 a	2 a	4 a	3 a
W-FT-097		4	150 -	109.0 a	54.3 a	4 a	1 a	37 a	2 a	2 -	845.8 a	21 a	16 a	1 a	31 a	4 a	2 a	3 a	2 a
W-FT-097		8	150 -	75.3 a	41.7 a	4 a	2 a	37 a	2 a	2 -	919.3 a	19 a	16 a	1 a	33 a	4 a	2 a	3 a	2 a
W-VT-007	Asian	0	150 -	245.0 a	85.3 a	3 a	3 a	35 a	2 a	2 -	193.5 a	20 a	14 a	1 a	29 a	5 a	2 -	3 a	3 a
W-VT-007		4	150 -	382.5 a	112.5 a	1 a	3 a	35 a	2 a	2 -	203.8 a	19 a	15 a	1 ab	30 a	7 a	2 -	3 a	4 a
W-VT-007		8	150 -	419.3 a	112.0 a	1 a	3 a	32 a	2 a	2 -	380.0 b	18 a	15 a	1 b	29 a	5 a	2 -	3 a	3 a
W-VT-008	European	0	150 a	699.3 a	99.8 a	4 a	3 a	27 a	2 a	2 a	307.0 a	20 a	13 a	2 a	31 a	6 a	2 a	3 a	3 a
W-VT-008		4	73 b	179.3 b	67.5 a	4 a	2 b	25 a	3 a	2 a	507.5 b	19 a	14 a	1 a	31 a	7 a	2 a	3 a	3 a
W-VT-008		8	72 b	254.3 b	61.3 a	4 a	2 b	27 a	2 a	2 a	214.5 a	21 a	14 a	2 a	28 a	6 a	2 a	2 a	3 a
W-VT-012	Asian	0	150 a	412.3 a	96.3 a	3 a	3 a	25 a	2 a	8 a	162.7 a	29 a	11 a	3 a	30 a	4 a	2 a	2 a	1 a
W-VT-012		4	47 b	291.0 a	82.7 a	3 a	3 a	25 a	2 a	8 a	247.3 a	30 a	13 a	2 a	26 a	4 a	3 a	2 a	1 a
W-VT-012		8	36 b	36.5 b	35.3 b	4 a	2 a	25 a	2 a	7 a	170.3 a	28 a	11 a	3 a	26 a	3 b	3 a	3 a	1 a
W-VT-013	Asian	0	150 a	251.3 a	89.7 a	2 a	2 a	27 a	2 a	7 a	589.0 a	28 a	15 a	2 a	26 a	3 a	3 a	2 a	1 a
W-VT-013		4	34 b	39.3 b	38.8 b	4 b	2 a	23 a	2 a	7 a	243.0 b	25 a	11 a	2 a	37 a	3 a	2 a	3 a	1 a
W-VT-013		8	56 b	64.8 b	45.5 b	4 b	2 a	23 a	2 a	8 a	293.0 b	25 a	15 a	2 a	28 a	4 a	3 a	4 a	1 a
W-VT-014	European	0	150 a	264.0 a	91.0 a	2 a	3 -	29 a	2 a	4 a	40.8 a	21 a	13 -	2 -	33 -	2 -	2 -	3 -	2 -
W-VT-014		4	150 a	385.3 a	126.0 a	1 a	3 -	25 a	1 a	5 a	69.0 a	21 a	14 -	2 -	34 -	4 -	2 -	3 -	1 -
W-VT-014		8	86 a	149.0 a	83.0 a	2 a	3 -	23 a	2 a	5 a	201.0 a	23 a	17 -	1 -	27 -	5 -	1 -	3 -	1 -
W-VT-017	European	0	150 a	444.3 a	131.8 a	1 a	3 a	31 a	1 a	2 -	154.5 a	20 a	15 a	1 a	30 a	4 a	2 a	2 a	3 a
W-VT-017		4	118 a	627.3 a	151.7 a	1 a	3 a	28 a	1 a	2 -	272.3 a	21 a	14 a	1 a	32 a	5 a	2 a	3 b	3 a
W-VT-017		8	118 a	365.3 a	110.3 a	2 a	2 a	30 a	2 a	2 -	232.0 a	21 a	15 a	1 a	29 a	6 a	2 a	3 b	3 a
W-VT-018	European	0	150 -	390.7 a	108.0 a	2 a	3 a	28 a	2 a	2 a	138.3 a	18 a	15 a	1 a	30 a	7 a	2 a	3 a	3 a
W-VT-018		4	150 -	267.3 a	96.7 a	2 a	3 a	30 a	2 a	2 a	160.3 a	18 a	15 a	1 a	32 a	8 a	2 a	3 a	3 a
W-VT-018		8	150 -	294.8 a	102.5 a	3 a	3 a	31 a	2 a	2 a	111.0 a	19 a	15 a	1 a	32 a	7 a	2 a	3 a	3 a
W-VT-044	European	0	150 a	217.3 a	88.3 a	1 a	3 a	34 a	1 a	2 a	203.0 a	19 a	14 a	1 a	32 a	6 a	2 a	2 a	4 a
W-VT-044		4	118 a	404.3 a	109.3 a	1 a	3 a	35 a	1 a	3 a	208.3 a	19 a	12 a	2 a	29 a	5 a	2 a	3 a	4 a
W-VT-044		8	118 a	151.8 a	67.0 a	3 a	3 a	39 a	2 a	2 a	238.3 a	20 a	12 a	2 a	29 a	6 a	2 a	2 a	4 a

Accession	Subpopulation	Vernalization (week)	FT (days)	Twe (gram)	Twi (mm)	TSh	Tgd	Tso	Tss	TC	Lwe (gram)	LL (cm)	LBW (cm)	LI	LC	PW (mm)	LS	LAT	LES
W-VT-052	European	0	124 a	308.8 a	70.0 a	6 a	2 a	30 a	2 a	2 a	144.3 a	20 a	12 a	2 a	32 a	4 a	2 a	3 a	3 a
W-VT-052		4	66 ab	171.3 ab	62.3 a	6 a	2 a	31 a	2 a	2 a	244.0 b	19 a	13 a	1 a	39 a	6 a	2 a	3 a	3 a
W-VT-052		8	22 b	54.0 b	43.5 a	6 a	3 a	30 a	2 a	5 a	210.5 b	18 a	11 a	2 a	30 a	5 a	2 a	2 b	4 a
W-VT-053	European	0	150 a	195.5 a	80.0 a	3 a	2 a	32 a	3 a	2 a	116.5 a	18 a	14 a	1 a	29 a	6 a	2 a	3 a	3 a
W-VT-053		4	150 a	128.3 ab	57.3 a	4 a	2 a	32 a	3 a	2 a	154.7 a	21 a	15 a	1 a	29 a	6 a	2 a	2 a	4 a
W-VT-053		8	101 a	83.0 b	52.3 a	4 a	2 a	31 a	3 a	2 a	255.5 a	19 a	15 a	1 a	27 a	9 a	2 a	2 a	4 a
W-VT-089	European	0	150 a	698.0 a	149.3 a	2 a	3 -	25 a	1 a	5 a	438.3 a	22 a	15 a	1 a	34 a	5 a	2 a	3 a	3 a
W-VT-089		4	118 a	578.5 a	147.5 a	1 a	3 -	26 a	2 a	5 a	446.0 a	22 a	16 a	1 a	32 a	6 a	3 a	3 a	4 a
W-VT-089		8	32 b	30.3 b	32.8 b	4 b	2 -	30 a	3 b	5 a	328.3 a	22 a	16 a	1 a	31 a	5 a	2 a	2 a	4 a
W-VT-091	European	0	150 a	646.5 a	116.0 a	3 a	3 a	32 a	2 a	2 a	285.0 a	20 a	15 a	1 a	33 a	3 a	2 a	2 a	3 a
W-VT-091		4	150 a	378.5 b	93.0 a	4 a	3 a	28 a	2 a	2 a	188.5 a	19 a	14 a	1 a	31 a	3 a	2 a	3 a	3 a
W-VT-091		8	106 a	213.0 b	70.3 a	4 a	3 a	30 a	2 a	2 a	350.0 a	19 a	14 a	1 a	32 a	1 a	2 a	2 a	2 a
W-VT-115	Asian	0	59 a	308.3 a	100.0 a	2 a	3 a	23 a	2 a	2 -	193.3 a	23 a	12 a	2 a	33 a	2 a	1 a	3 a	1 -
W-VT-115		4	33 a	39.5 b	40.0 b	4 b	2 b	23 a	3 b	2 -	179.0 a	19 ab	11 ab	2 a	33 a	3 a	2 a	3 a	1 -
W-VT-115		8	49 a	22.0 b	26.8 b	5 c	2 b	25 a	3 ab	2 -	124.8 a	16 b	8 b	2 a	27 a	2 a	2 a	1 a	1 -
W-VT-117	Asian	0	51 a	335.0 a	105.3 a	2 a	3 a	23 a	1 a	6 a	263.0 a	22 a	12 a	2 a	32 a	5 a	2 a	2 a	3 a
W-VT-117		4	27 b	10.8 b	22.3 b	5 b	1 b	24 a	2 b	3 b	136.8 b	19 a	12 a	2 a	27 a	3 a	1 a	1 a	3 a
W-VT-117		8	23 b	4.0 b	14.3 b	4 b	1 b	30 b	3 b	3 ab	60.8 c	12 b	7 b	2 a	28 a	4 a	1 a	1 a	1 b
W-VT-120	European	0	150 -	493.8 a	110.8 a	3 a	3 -	31 a	2 a	2 -	216.0 a	20 a	15 a	1 a	34 a	4 a	2 a	3 a	3 a
W-VT-120		4	150 -	349.3 a	112.0 a	2 a	3 -	24 b	2 a	2 -	219.0 a	20 a	15 a	1 a	33 a	6 a	3 a	3 a	4 a
W-VT-120		8	150 -	296.3 a	96.3 a	2 a	3 -	29 a	2 a	2 -	191.0 a	18 a	13 a	1 a	30 a	2 a	2 a	4 a	3 a
W-VT-123	Asian	0	118 a	327.3 a	52.7 a	6 a	2 a	24 a	2 a	6 a	608.7 a	25 a	13 a	2 a	34 a	2 a	2 a	3 a	2 a
W-VT-123		4	46 b	9.5 b	16.0 b	5 a	2 a	28 ab	2 a	6 a	280.0 b	18 ab	12 a	2 a	31 a	2 a	2 a	2 a	1 a
W-VT-123		8	30 b	52.0 b	25.5 b	5 a	2 a	31 b	2 a	5 a	288.5 b	20 b	13 a	2 a	34 a	5 b	3 a	3 a	1 a
W-VT-137	European	0	150 -	333.0 a	104.5 a	2 a	3 a	30 a	2 a	2 a	146.3 a	18 a	13 a	1 a	34 a	5 a	1 a	3 a	3 a
W-VT-137		4	150 -	356.0 a	112.3 a	1 a	3 a	28 a	2 a	3 a	275.0 b	21 a	15 a	1 a	32 a	3 a	1 a	2 b	3 a
W-VT-137		8	150 -	421.3 a	109.8 a	2 a	3 a	33 a	2 a	2 a	322.5 b	19 a	14 a	1 a	30 a	5 a	2 a	3 a	4 a
